# Access to pyrrolines and fused diaziridines by selective radical addition to homoallylic diazirines†‡

**DOI:** 10.1039/d3sc04886a

**Published:** 2023-12-29

**Authors:** Zhigang Ma, Xinxin Wu, Haotian Li, Zhu Cao, Chen Zhu

**Affiliations:** a Key Laboratory of Organic Synthesis of Jiangsu Province, College of Chemistry, Chemical Engineering and Materials Science, Soochow University 199 Ren-Ai Road Suzhou Jiangsu 215123 China chzhu@suda.edu.cn; b Frontiers Science Center for Transformative Molecules, Shanghai Key Laboratory for Molecular Engineering of Chiral Drugs, Shanghai Jiao Tong University 800 Dongchuan Road Shanghai 200240 China chzhu@sjtu.edu.cn

## Abstract

Pyrroline derivatives are common in bioactive natural products and therapeutic agents. We report here a synthesis of pyrrolines and fused diaziridines by divergent radical cyclization of homoallylic diazirines, which can serve as an internal radical trap and a nitrogen source. This reaction proceeds by selective radical addition to C=C or N=N bonds followed by intramolecular cyclization. Frontier molecular orbital analysis provides a deep insight into the origin of the selectivity. The reaction demonstrates a new cyclization mode, broad functional group compatibility and high product diversity, and reveals a much broader chemical space for diazirine studies.

## Abstract 2

A novel, divergent radical cyclization of homoallylic diazirines for the synthesis of pyrrolines and fused diaziridines is described.

## Graphical abstract

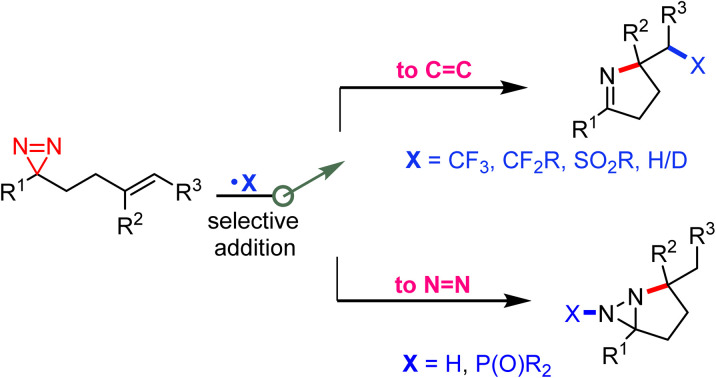

Pyrroline and pyrrolidine structural motifs are widely encountered in bioactive natural products in plants, insects such as myrmicine ants and animals, including poison frogs.^1,2^ Consequently, new strategies to access pyrrolines and analogues address a long-standing interest of chemists. Notwithstanding the interest in transition-metal catalyzed synthesis of pyrroline derivatives over the past few decades,^3^ radical approaches have also received remarkable attention owing to their innately attractive features which include mild reaction conditions and good tolerance of susceptible functional groups.^4^ The conventional radical approaches proceed through the generation of an iminyl radical by homolysis or single-electron transfer (SET) in labile precursors and the subsequent intramolecular N → C cyclization of the iminyl radical with C=C bonds. External radical acceptors are sought which can terminate the cascade reaction and furnish pyrroline products (Fig. 1a).^5^ We have conceived a reverse route which can access pyrrolines by C → N cyclization using external radical donors rather than acceptors (Fig. 1b). This process is intended to enrich product diversity and lead to distinct pyrrolines, such as CF_3_-substituted pyrrolines, which are difficult to produce by conventional methods. This is because there are no SOMOphiles that lead to the introduction of, for example, CF_3_ groups.

**Fig. 1:**
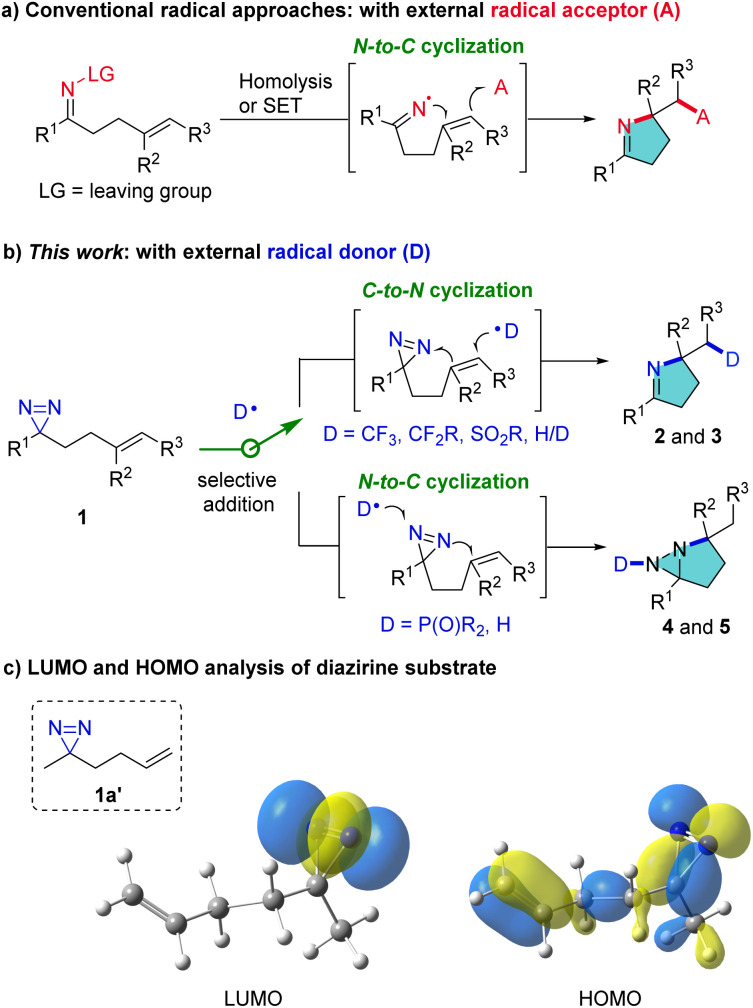
Radical synthetic approaches of pyrroline derivatives.

Diazirines, which consist of one carbon and two nitrogen atoms in an unsaturated three-membered ring, are often exploited in chemical biology as carbene precursors.^6^ They have been reported only rarely in radical reactions. The use of specific perfluoroalkyl-substituted diazirines to trap carbon-centered radicals was pioneered by Barton,^7^ and recently extended by Lopchuk *et al.*^8^ and Liao *et al.*^9^ Despite this elegant research, the use of diazirines as a nitrogen source for construction of valuable N-heterocycles remains underexplored. Herein, we report novel, divergent radical cyclization reactions of homoallylic diazirines (1) for the synthesis of pyrrolines and fused diaziridines (Fig. 1b). The selectivity of addition of an external radical to the C=C or N=N bond is dictated by the inherent polarity of the radical. The transformation of 1 can proceed through serial radical addition to C=C and N=N bonds, leading to diverse multi-functionalized pyrrolines (2 and 3). Alternatively, the reaction can proceed by first adding the external radical to the N=N bond, followed by cyclization with the C=C bond, resulting in unusual fused diaziridines (4 and 5). The products may define a much broader chemical space for diazirine studies, and in particular they can probe the reactivity and synthetic potential of diazirines.

At the outset, frontier molecular orbital (FMO) analysis of the simplified model (1a′) was conducted. The aim was to gain an insight into the discrepancy in the energy levels of alkenes and diazirines in the same molecule and to examine the feasibility of the hypothesis from a theoretical point of view. The results of the analysis demonstrated that the lowest unoccupied molecular orbital (LUMO) of 1a′ is dominated by the LUMO of the diazirine moiety (Fig. 1c, left), while the highest occupied molecular orbital (HOMO) of 1a′ is located more in the alkenyl moiety than in the diazirine (Fig. 1c, right). It was postulated that selective radical addition to the C=C or N=N bond might be controlled with an appropriate external radical. Experimental investigations began with the addition of a CF_3_ radical to the dialiphatic diazirine (1a). The reactivity in radical reactions of dialiphatic diazirines without aryl or perfluoroalkyl substituents has not been explored. The CF_3_ radical was generated from the Ruppert–Prakash reagent (TMSCF_3_) which was oxidized by (diacetoxyiodo)benzene (PIDA) at 0 °C (for details, see the ESI‡). The transformation was initiated by adding a CF_3_ radical to the C=C bond of 1a and ended with N=N interception, leading as expected, to the pyrroline (2a) (Scheme 1). This product is unlikely to be formed by the previously described N → C cyclization in Fig. 1b, due to the lack of a radical acceptor that can transfer the CF_3_ group. The use of CsF as an additive is crucial to the reaction outcome, and other bases such as Cs_2_CO_3_ or KF were ineffective in this reaction.

**Scheme 1:**
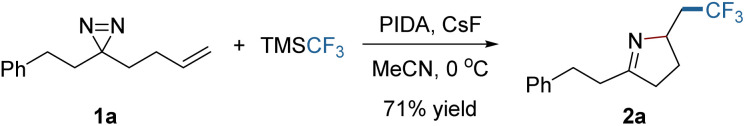
Optimal conditions for the formation of pyrroline by radical trifluoromethylation.

With the optimized reaction conditions established, the scope of aliphatic diazirines was explored (Fig. 2). An array of diazirines bearing diverse skeletons were tested and easily converted into the corresponding pyrrolines in synthetically useful yields. The benzyl-substituted diazirine (1h) gave rise to a mixture of a product (2h) with an over-oxidized product (2h′), resulting from the spontaneous oxidation of the methylene between the phenyl and the pyrroline in air during the work-up procedure.^3*f*,10^ Diazirines bearing 1,1-disubstituted alkenes tended to construct pyrrolines containing quaternary centers regardless of steric hindrance. The substituents could contain alkyl, alkenyl, aryl, or heteroaryl groups (2i–2t). The electronic properties of aryl substituents had only a modest impact on the reaction outcomes (2m–2r). In those cases, the undesired oxidation of the tertiary benzylic radical intermediates to cations, which can occur under oxidative conditions, was not observed, indicating the priority of the intramolecular trapping of the benzylic radical by the N=N bond. The reaction of a diazirine (1u) bearing an internal alkene also proceeded smoothly, leading to an aza-spiro product (2u) with exclusive diastereoselectivity (dr > 20 : 1). Aryl-substituted diazirines (2v) are also suitable substrates for the reaction. In addition to pyrrolines, tetrahydropyridine as a six-membered analogue could also be produced under the same conditions (2w, 2x and 2y), enriching the product library. Construction of other six-membered N-containing heterocycles could also be anticipated by placing various heteroatoms on the aliphatic chain. Attempts to construct seven- or eight-membered or even larger N-containing heterocycles by extending the aliphatic chain were unsuccessful. Remarkably, it was found however that this protocol can be applied to the dearomative 2,3-difunctionalization of indoles, providing spiro-indoline products (2z–2ab) with excellent stereo-selectivity. These three-dimensional indolines are widely distributed in various bioactive molecules.^11^

**Fig. 2:**
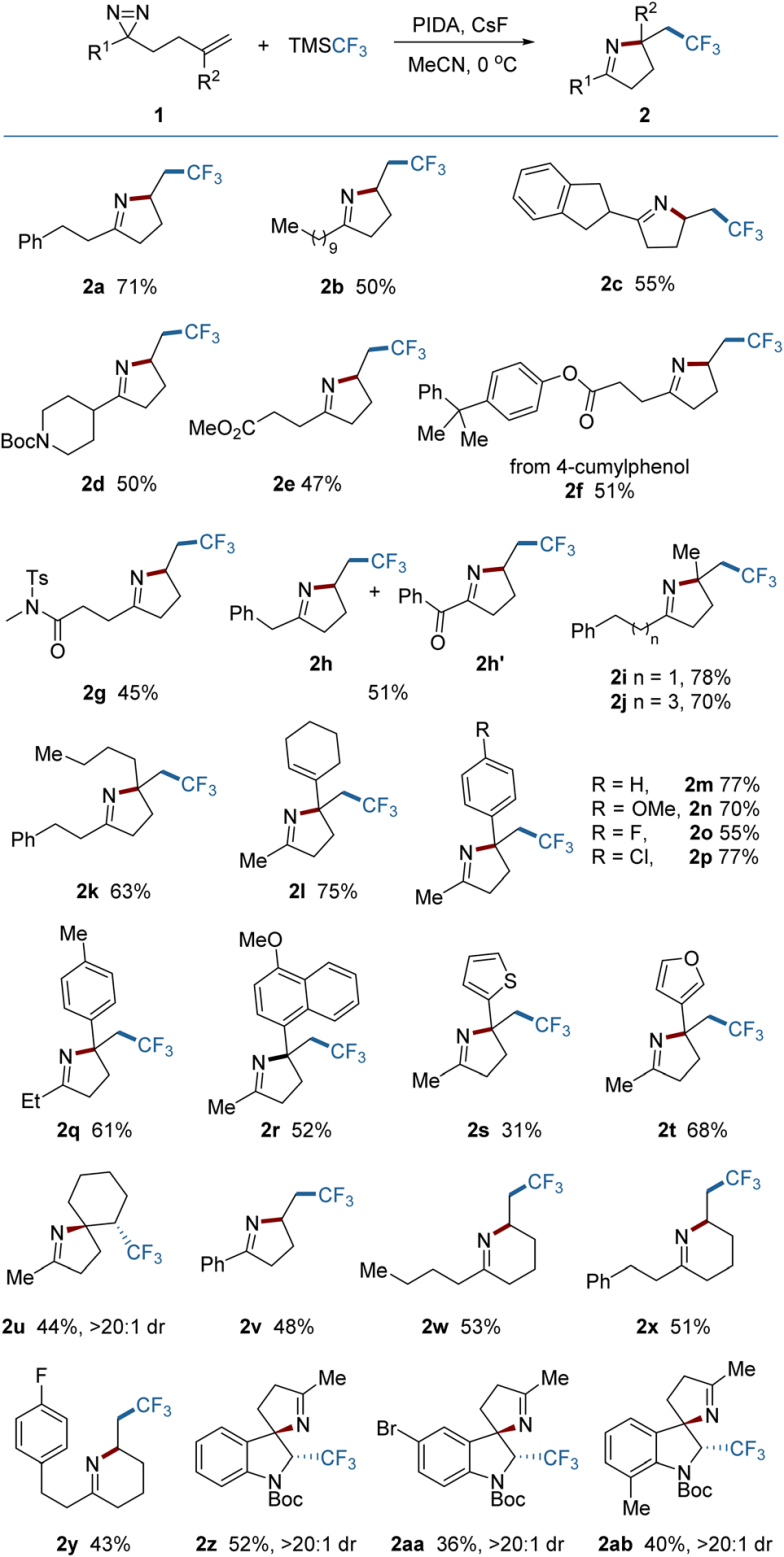
Scope of diazirines. Reaction conditions: 1 (0.2 mmol), TMSCF_3_ (0.6 mmol), PIDA (0.3 mmol), and CsF (0.3 mmol) in 2.0 mL MeCN under Ar at 0 °C for 1 h. Yields of isolated products are given.

Using electrophilic radicals other than the CF_3_ radical further enriched the diversity of products from the reaction. As shown in Fig. 3, a portfolio of external radicals, such as di- or perfluoroalkyl radicals, electron-deficient alkyl radicals and sulfonyl radicals proved to be amenable to photoredox catalytic conditions, leading to the corresponding pyrrolines (3a–3m) in acceptable yields. The formation of 3e and 3f from the enyne-substituted diazirine precursors, is noteworthy, as the nascent propargyl radical was retained and engaged in the cyclization without conversion to the tautomeric allenyl radical.^12^ A set of (hetero)aryl, alkyl and styryl substituted sulfonyl radicals were added readily to the substrates, but the strong electron-deficient radicals delivered a lower yield (3k). A sulfonyl radical could also be generated under thermal conditions using copper(ii) acetate as the oxidant and sodium *p*-toluenesulfinate as the radical source (3g). Combining this process with a metal-hydride hydrogen atom transfer (MHAT) led to the products containing incorporated hydrogen or deuterium (3n, 3o) in good yields, demonstrating the breadth of the method.

**Fig. 3:**
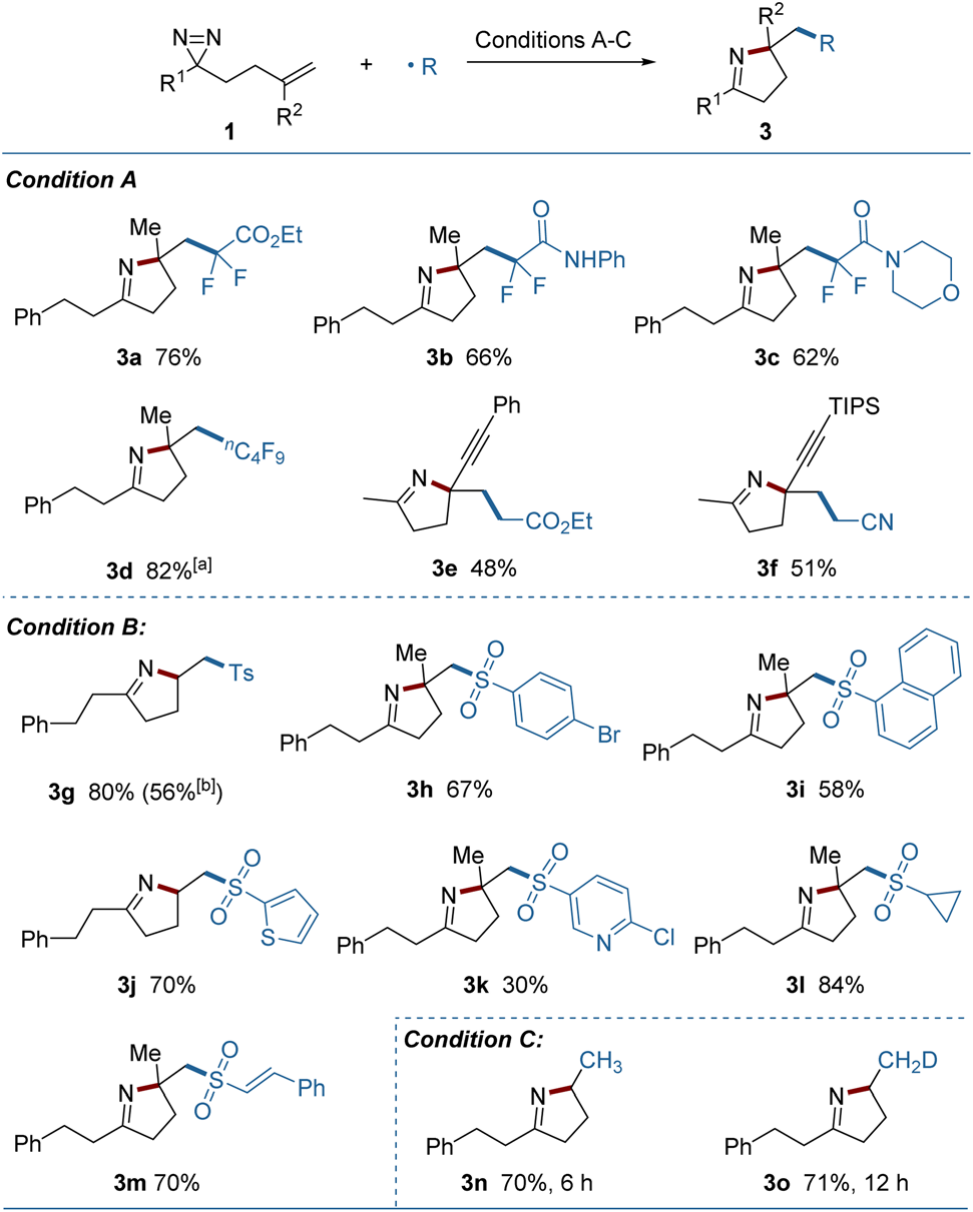
Investigation of external radicals. Condition A: 1 (0.2 mmol), (fluoro)alkyl bromide (0.4 mmol), *fac*-Ir(ppy)_3_ (0.004 mmol), Hantzsch ester (0.4 mmol) and 2,6-lutidine (0.4 mmol) in dry MeCN (2.0 mL) under Ar irradiated with 5 W × 2 blue LEDs at rt for 12 h. Condition B: 1 (0.2 mmol), sulfonyl chloride (0.4 mmol), *fac*-Ir(ppy)_3_ (0.004 mmol), DIPEA (0.4 mmol) and K_2_HPO_4_ (0.4 mmol) in MeCN/H_2_O (2.0 mL/0.2 mL) under Ar irradiated with 5 W × 2 blue LEDs at rt for 12 h. Condition C: 1 (0.2 mmol), PhSiH_3_ or PhSiD_3_ (0.4 mmol), Fe(acac)_3_ (0.06 mmol) and EtOH (0.4 mmol) in THF (2.0 mL) under air at 60 °C. Yields of isolated products are given. ^*a*^Perfluoroalkyl iodide was used. ^*b*^With sodium *p*-toluenesulfinate (0.3 mmol) and Cu(OAc)_2_ (0.2 mmol) in MeCN (2.0 mL) at 60 °C for 13 h.

It was found that the addition of P-centered radicals to diazirine (1) proceeded through a distinct pathway and resulted in fused diaziridine products (Fig. 4). Diarylphosphinoyl radicals were generated from diarylphosphine oxides in the presence of silver nitrate as a catalyst, and were selectively added to the N=N rather than the C=C bond. The N → C cyclization generated an alkyl radical reaction which was terminated by HAT from diarylphosphine oxide. Meanwhile, the diarylphosphinoyl radical was regenerated, perpetuating the radical chain process. Compared to the diazirines (4a, 4b) which bear a 1,2-dialkyl alkene, the diazirine bearing a trisubstituted alkene delivered a lower yield (4c), probably because of the weak H-atom transfer (HAT) ability of the tertiary alkyl radical and the steric congestion that impeded the HAT. The engagement of the highly reactive primary alkyl radicals from HAT was less controllable, and also led to a decreased yield (4d). Skeletal complex polycyclic diaziridines (4e, 4f) are otherwise difficult to prepare, but were furnished in useful yields by this method. A quaternary carbon center can be readily constructed in this reaction.

**Fig. 4:**
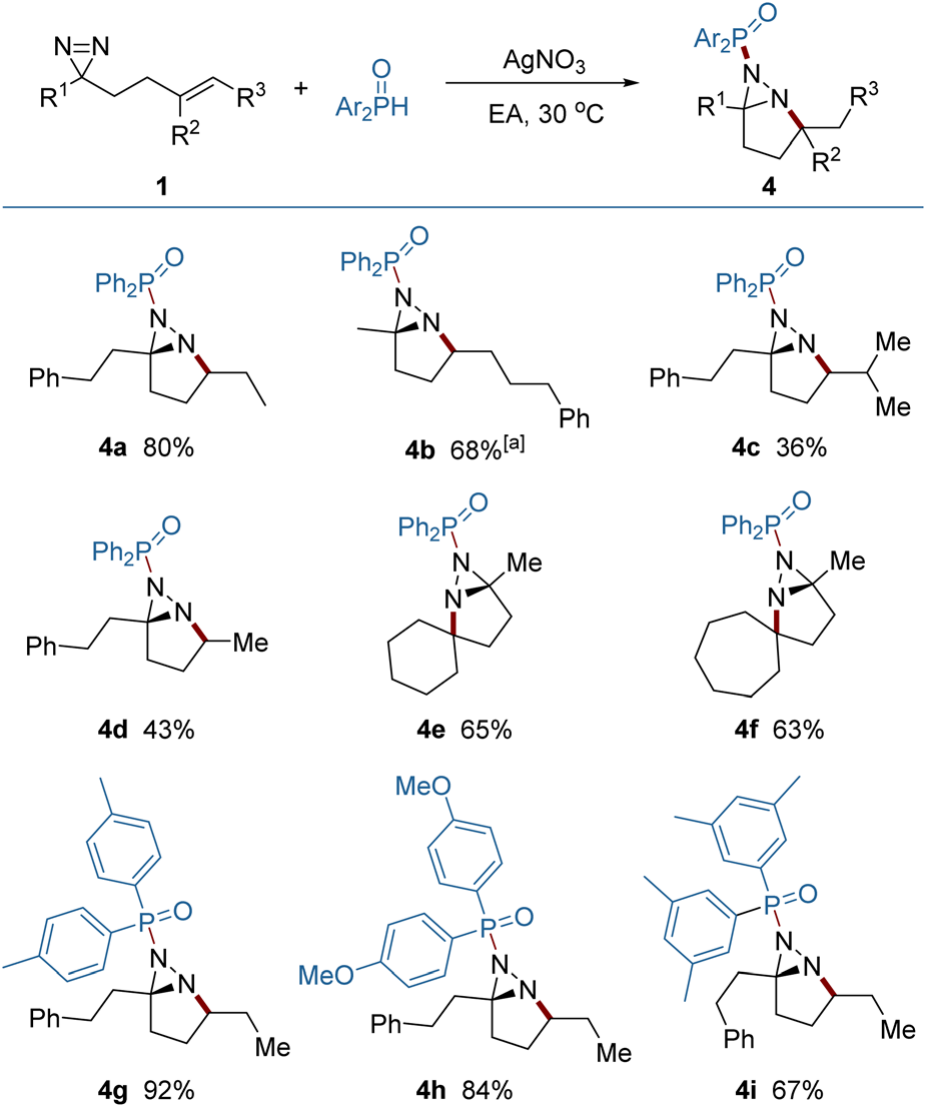
Synthesis of fused diaziridines by P-radical addition. Reaction conditions: 1 (0.2 mmol), diarylphosphine oxide 6 (0.4 mmol) and AgNO_3_ (0.02 mmol) in EA (2.0 mL) under Ar at 30 °C. Yields of isolated products are given. For 4a–4i, dr > 20 : 1. ^*a*^At 40 °C.

The reductive cyclization of 1 was accomplished to generate a new type of fused diaziridine (5) under photochemical conditions using a Hantzsch ester (HE) as a reducing agent (Fig. 5). These fused diaziridines might serve as versatile precursors for various N-heterocycles according to published approaches.^13^ The reaction leading to fused diaziridines was initiated by the reductive quenching of excited *fac*-Ir(ppy)_3_ with the Hantzsch ester to form an Ir(ii) species. Its strong reduction potential (*E*^III/II^_1/2_ = −2.19 V *vs.* SCE) enables single-electron reduction of the N=N bond of diazirine (*E*^RE^_1/2_ = −2.06 V *vs.* SCE) to an N-centered radical anion (for details, see the ESI‡), and this is followed by a sequence of intramolecular cyclization, HAT and protonation. It should be noted that excited *fac*-Ir(ppy)_3_
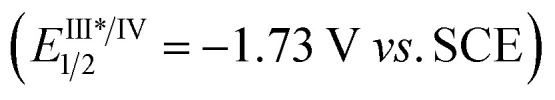
 is not sufficient to reduce diazirines to trigger the reaction.

**Fig. 5:**
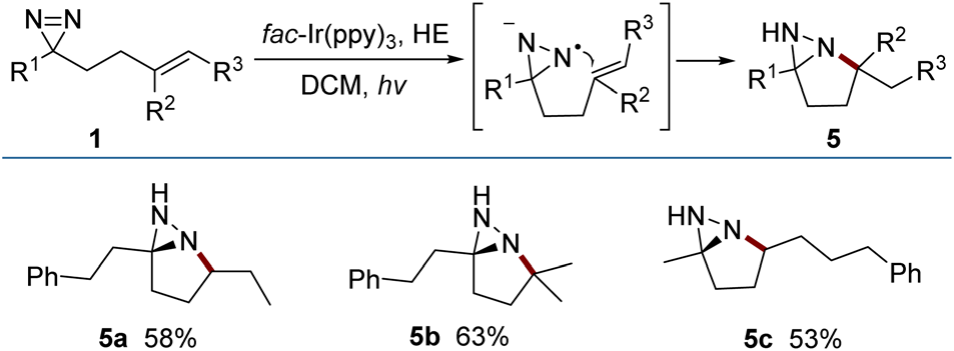
Reductive cyclization generating fused diaziridines. Reaction conditions: 1 (0.2 mmol), *fac*-Ir(ppy)_3_ (0.004 mmol) and Hantzsch ester (0.4 mmol) in dry DCM (4.0 mL) under Ar irradiated with 5 W × 2 blue LEDs at rt for 4 h. Yields of isolated products are given. For 5a–5c, dr > 20 : 1.

A scaled-up preparation of pyrroline (2m) from 2 mmol of 1m was achieved with 82% isolated yield, illustrating the practicality of the protocol. The products could be further converted to other valuable molecules (Fig. 6). For example, in the presence of diisobutyl aluminum-hydride (DIBAL-H), 2m was readily reduced to a pyrrolidine (6). The treatment of 2m with *m*-CPBA gave rise to a fused oxaziridine (7). The [3 + 2] cycloaddition of 2a with *N*-hydroxybenzimidoyl chloride generated the biologically relevant, CF_3_-substituted 1,2,4-oxadiazoline (8).^14^ Addition of an allyl Grignard reagent to the C=N bond of 2m in the presence of BF_3_·Et_2_O afforded 2,2,5,5-tetrasubstituted pyrrolidine (9). Simple treatment of 5a with acetyl chloride resulted in *N*-acetyl-tetrahydropyridazine (10) by a ring-opening process.

**Fig. 6:**
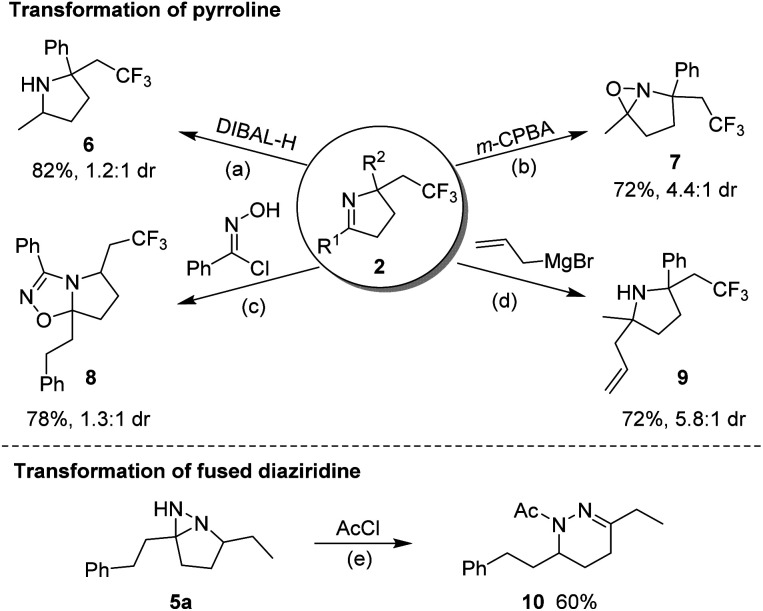
Product transformations. Reaction conditions: (a) 2m (0.2 mmol), DIBAL-H (0.6 mmol) in DCM (2.0 mL) at −78 °C for 4 h; (b) 2m (0.2 mmol), *m*-CPBA (0.3 mmol) and Na_2_HPO_4_ (0.3 mmol) in DCM at rt for 2 h; (c) 2a (0.1 mmol), *N*-hydroxybenzimidoyl chloride (0.15 mmol) and Et_3_N (0.2 mmol) in DCM (2.0 mL) at rt for 3 h; (d) 2m (0.2 mmol), Et_2_O·BF_3_ (0.6 mmol) and allyl magnesium bromide (0.6 mmol) in THF (5.0 mL) at −78 °C for 4 h; (e) 5a (0.2 mmol), AcCl (0.6 mmol) and Et_3_N (0.6 mmol) in DCM (2.0 mL) for 3 h at rt.

Based on the above experimental and computational results, a plausible mechanism is depicted in Fig. 7. For electrophilic radicals such as the fluoroalkyl and sulfonyl radicals, the selective addition to the C=C bond of 1, followed by intramolecular trapping of a radical (a) by the N=N bond gives rise to an N-centered radical (b), which can form a dimer (c). Intermediate c is unstable, and simultaneously undergoes N_2_ extrusion to afford two molecules of the pyrroline product.^7,15^ The formation of radical b was confirmed by the detection by HRMS of the fused diaziridine (d) which is gained from competitive HAT. In contrast, the relatively nucleophilic phosphinoyl radical prefers addition to the N=N bond of 1, resulting in radical e. The subsequent intramolecular cyclization to the C=C bond and HAT affords the fused diaziridine products. The formation of intermediate e was confirmed by the isolation of byproduct g derived from HAT.

**Fig. 7:**
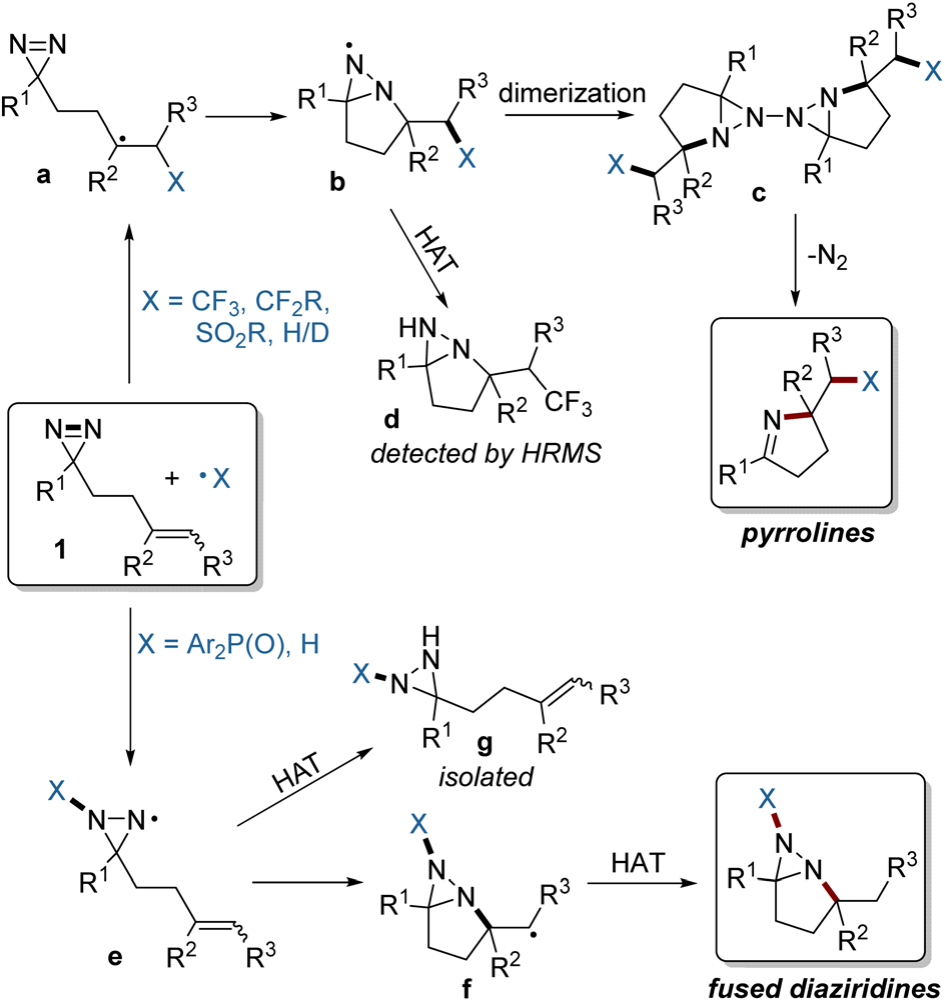
Proposed reaction mechanism.

## Conclusions

In summary, a novel radical-mediated divergent synthesis of pyrrolines and fused diaziridines from rationally designed alkene-substituted diazirines is described. The key to the success of the reaction is the selective addition of an external radical to the C=C or N=N bond, which is controlled by the inherent electronic characteristics of the external radical. FMO analysis sheds light on the underlying reason for the chemoselectivity. The protocol features good functional group tolerance and high product diversity, and creates a much broader chemical space for diazirine studies.

## Data availability

All relevant experimental and computational data and characterization details are provided in the ESI.‡

## Author contributions

C. Z. conceived the idea and designed the experiments, Z. M. performed most of the experiments, X. W. performed the DFT calculations, H. L. synthesized some of the starting materials, Z. C. helped with analysis of the data, and C. Z. supervised the research and co-wrote the manuscript.

## Conflicts of interest

There are no conflicts to declare.

## Supplementary Material

SC-015-D3SC04886A-s001
