# Supplementary material for: Access to pyrrolines and fused diaziridines by selective radical addition to homoallylic diazirines
Source: Chem Sci. 2023 Dec 29;15(5):1879–84. doi: 10.1039/d3sc04886a (PMC10829008; doi:10.1039/d3sc04886a)

Electronic Supplementary Material (ESI) for Chemical Science.
This journal is © The Royal Society of Chemistry 2023

Supporting Information

Access to Pyrrolines and Fused Diaziridines by Selective Radical Addition to Homoallylic Diazirines

Zhigang Ma,[a] # Xinxin Wu,[a] # Haotian Li,[a] Zhu Cao,[b] and Chen Zhu*[a,b]

Content

1. General experimental details 3

2. Reaction parameters survey and general procedures 3

2.1 For radical-mediated trifluoromethylation (Fig 2) 3

2.2 For radical-mediated difluoroalkylation (Fig 3) 4

2.3 For radical-mediated sulfonylation (Fig 3) 5

2.4 For radical-mediated hydrogenation (Fig 3) 6

2.5 For radical-mediated phosphinoylation (Fig 4) 6

2.6 For radical-mediated reductive cyclization (Fig 5) 7

3. Preparation of starting materials 7

3.1 General method A 7

3.2 General method B 8

4. Product transformations 9

5. Mechanistic studies 11

5.1 Identification of intermediates (Fig 7) 11

5.2 Cyclic voltammogram experiment 12

5.3 DFT calculations 13

6. References 14

7. Characterization of starting materials and products 14

8. NMR Spectra 35

1. General experimental details

All reactions were maintained under a nitrogen atmosphere unless otherwise stated. Commercially available reagents were used without further purification. DMF was distilled from NaH under reduced pressure, and THF was distilled from sodium. Infrared (FT-IR) spectra were recorded on a BRUKER VERTEX 70, ν_{max} in cm^{-1}. ^{1}H-NMR spectra were recorded on a BRUKER AVANCE III HD (400 MHz) spectrometer. Chemical shifts are reported in ppm from tetramethylsilane with the solvent resonance as internal standard ($CDCl_3$: δ 7.26). Data are reported as follows: chemical shift, multiplicity (s = singlet, d = doublet, t = triplet, q = quadruplet, br = broad, m = multiplet), coupling constants (Hz) and integration. ^{13}C-NMR spectra were recorded on a BRUKER AVANCE III HD (100 MHz) spectrometer with complete proton decoupling. Chemical shifts are reported in ppm from tetramethylsilane with the solvent resonance as the internal standard ($CDCl_3$: δ 77.16). ^{19}F-NMR spectra were recorded on a BRUKER AVANCE III HD (376 MHz) spectrometer, ^{31}P-NMR spectra were recorded on a BRUKER AVANCE III HD (162 MHz) spectrometer. High resolution mass spectrometry (HRMS) was measured with a GCT Premier™ and BRUKER micrOTF-Q III. Melting points were measured using INESA WRR and values are uncorrected.

2. Reaction parameters survey and general procedures

2.1 For radical-mediated trifluoromethylation (Fig 2)

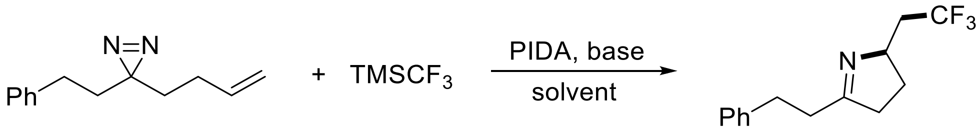

1a 2a

Entry	Base	Solvent	T (°C)	Yield[a]
1	CsF	MeCN	0	71%
2	CsF	THF	0	9%
3	CsF	DCM	0	trace
4	CsF	MeOH	0	nr
5	CsF	acetone	0	trace
6	CsF	EA	0	15%
7	CsF	DMF	0	40%
8	CsF	MeCN	-10	62%
9[b]	CsF	MeCN	0	70%
10[c]	KF	MeCN	rt	52%
11	Cs_2CO_3	MeCN	rt	15%

12	CsF	MeCN	0	trace[d]
13	CsF	MeCN	0	27%[e]

Table S1. Reaction conditions: **1a** (0.2 mmol), $TMSCF_3$ (0.6 mmol), (diacetoxyiodo)benzene (PIDA, 0.3 mmol) and base (0.3 mmol) in dry solvent (2.0 mL) were stirred vigorously (1300 rpm) under Ar atmosphere at indicated temperature for 1 h. [a] Yields of isolated product are given. [b] 3.0 mL MeCN. [c] Reaction for 12 h. [d] With 0.2 mmol of $TMSCF_3$. [e] With 0.4 mmol of $TMSCF_3$.

General procedure for the synthesis of 2

To a suspension of diazirine **1** (0.2 mmol), CsF (1.5 equiv., 45.6 mg) and PIDA (1.5 equiv., 96.6 mg) in dry acetonitrile (2.0 mL) was added $TMSCF_3$ (3.0 equiv, 88.6 uL) dropwise at 0 ℃. After the reaction mixture was stirred vigorously (1200 rpm) for 1 h at the same temperature, the reaction was quenched with water. The aqueous layer was extracted with EtOAc. The combined organic extracts were washed with brine and dried over anhydrous Na_2SO_4. After removal of solvents in vacuo, pyrroline **2** was isolated by flash column chromatography on silica gel.

2.2 For radical-mediated difluoroalkylation (Fig 3)

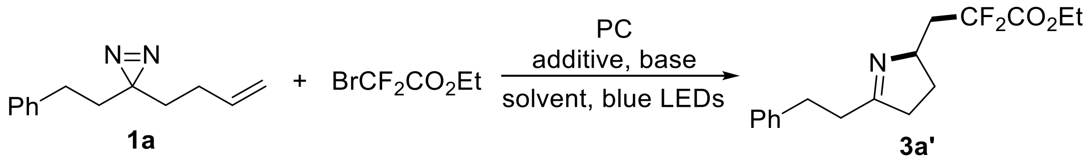

Entry	[Ir]	Base	Additive	Solvent	Yield[a]
1[b]	*fac*-Ir(ppy)$_3$	-	DIPEA	MeCN	13%
2[b]	*fac*-Ir(ppy)$_3$	-	HE	MeCN	33%
3[b]	*fac*-Ir(ppy)$_3$	K_2HPO_4	HE	MeCN/H_2O (10:1)	20%
4	*fac*-Ir(ppy)$_3$	K_2HPO_4	HE	MeCN	48%
5	[Ir(ppy)$_2$(dtbbpy)]PF_6	K_2HPO_4	HE	MeCN	60%
6	Eosin Y	K_2HPO_4	HE	MeCN	trace
7	Eosin Y-Na$_2$	K_2HPO_4	HE	MeCN	trace
8	*fac*-Ir(ppy)$_3$	K_2CO_3	HE	MeCN	41%
9	*fac*-Ir(ppy)$_3$	K_3PO_4	HE	MeCN	34%
10	*fac*-Ir(ppy)$_3$	KH_2PO_4	HE	MeCN	24%

Table S2. Reaction conditions: **1a** (0.2 mmol), ethyl bromodifluoroacetate (0.4 mmol), PC (2 mol %), additive (0.4 mmol) and base (0.4 mmol) in dry solvent (2.0 mL) under Ar atmosphere were irradiated under 5 W × 2 blue LEDs for 12 h. [a] Yields of isolated products are given. [b] 1 mol % PC was used.

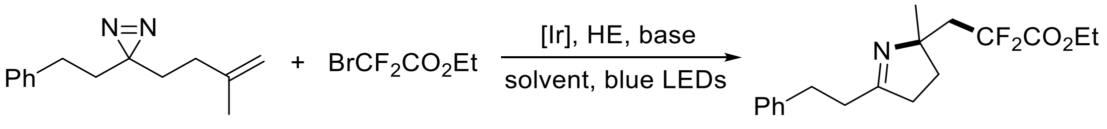

Entry	[Ir]	Base	Solvent	Yield[b]
1	*fac*-Ir(ppy)$_3$	K_2HPO_4	MeCN/H_2O (10:1)	58%
2	*fac*-Ir(ppy)$_3$	K_2HPO_4	MeCN	71%
3	[Ir(ppy)$_2$(dtbbpy)]PF$_6$	K_2HPO_4	MeCN	67%
4	*fac*-Ir(ppy)$_3$	Na_2HPO_4	MeCN	63%
5	*fac*-Ir(ppy)$_3$	2,6-Lutidine	MeCN	76%

Table S3. Reaction conditions: **1i** (0.2 mmol), ethyl bromodifluoroacetate (0.4 mmol), PC (2 mol %), Hantzsch ester (0.4 mmol) and base (0.4 mmol) in dry solvent (2.0 mL) under Ar atmosphere were irradiated under 5 W × 2 blue LEDs for 12 h. [b] Yields of isolated product are given.

General procedure for the synthesis of 3a-3f

To a 4-mL oven-dried reaction vial was charged with **1** (0.2 mmol), *fac*-Ir(ppy)$_3$ (2 mol %) and Hantzsch ester (HE, 2 equiv.). The reaction vial was back-flushed with argon three times. Then dry MeCN (2.0 mL), alkylbromide (2 equiv.) and 2,6-lutidine (2 equiv.) was added to the reaction vial via syringe. The reaction mixture was vigorously stirred at rt for 12 h under the irradiation with 5 W × 2 blue LEDs (approximately 5 cm away from the light sources). After removal of solvents in vacuo, pyrroline **3** was isolated by flash column chromatography on silica gel.

2.3 For radical-mediated sulfonylation (Fig 3)

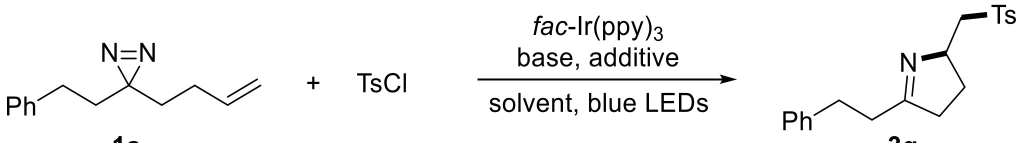

Entry	Base	Additive	Solvent	Yield[a]
1	Na_2HPO_4	HE	MeCN	31%
2	Na_2HPO_4	DIPEA	MeCN	45%
3	K_2HPO_4	DIPEA	MeCN	44%
4	K_2HPO_4	DIPEA	MeCN/H_2O (10:1)	79%
5	K_2HPO_4	DABCO	MeCN/H_2O (10:1)	trace
6	K_2HPO_4	Quinuclidine	MeCN/H_2O (10:1)	trace

Table S4. Reaction conditions: **1a** (0.2 mmol), TsCl (0.4 mmol), *fac*-Ir(ppy)$_3$ (2 mol %), additive (0.4 mmol) and base (0.4 mmol) in solvent (2.0 mL) under Ar atmosphere were irradiated under 5 W × 2 blue LEDs for 12 h. [a] Yields of isolated products are given.

General procedure for the synthesis of 3g-3m

To a 4-mL reaction vial was charged with **1** (0.2 mmol), *fac*-Ir(ppy)$_3$ (2 mol %), sulfonyl chloride (2 equiv.) and K_2HPO_4 (2 equiv.). The reaction vial was back-flushed with argon three times. Then MeCN (2.0 mL), H_2O (0.2 mL) and DIPEA (2 equiv.) were added to the reaction vial via syringe. The reaction mixture was vigorously stirred at rt for 12 h under the irradiation with 5 W × 2 blue LEDs (approximately 5 cm away from the light sources). After removal of solvents in vacuo, pyrroline **3** was isolated by flash column chromatography on silica gel.

2.4 For radical-mediated hydrogenation (Fig 3)

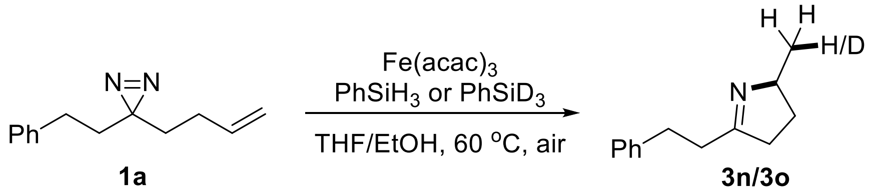

1a **3n/3o**

Reaction conditions: **1a** (0.2 mmol), $PhSiH_3$ or $PhSiD_3$ (0.4 mmol), Fe(acac)$_3$ (0.06 mmol) and EtOH (0.4 mmol) in THF (2.0 mL) under air at 60 °C.

General procedure for the synthesis of 3n and 3o

To a 4-mL oven-dried reaction vial was charged with **1a** (0.2 mmol), $PhSiH_3$ or $PhSiD_3$ (2 equiv.), Fe(acac)$_3$ (3 equiv.), EtOH (2 equiv.) and THF (2.0 mL). The reaction mixture was stirred at 60 °C under air. After removal of solvents in vacuo, pyrroline **3** was isolated by flash column chromatography on silica gel.

2.5 For radical-mediated phosphinoylation (Fig 4)

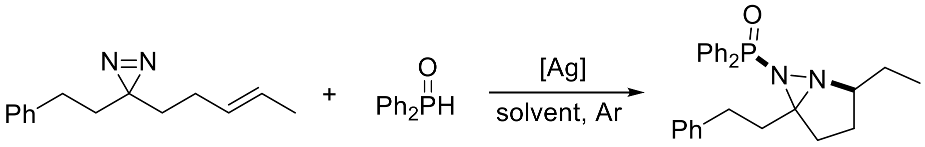

1ac **4a**

Entry	[Ag] (mol%)	Solvent	T (°C)	Time (h)	Yield[a]
1	$AgNO_3$ (100)	MeCN	50	1	66%
2	$AgNO_3$ (20)	MeCN	50	6	64%
3	AgOAc (20)	MeCN	50	72	11%
4	AgTFA (20)	MeCN	50	72	23%
5	AgOTf (20)	MeCN	50	72	44%
6	$AgBF_4$ (20)	MeCN	50	72	40%
7	$AgSbF_6$ (20)	MeCN	50	72	38%
8	Ag_2O (10)	MeCN	50	72	trace
9	Ag_2CO_3 (10)	MeCN	50	72	trace
10	$AgNO_3$ (20)	DMF	50	6	57%
11	$AgNO_3$ (20)	DCE	50	6	61%

12	$AgNO_3$ (20)	$PhCF_3$	50	6	71%
13	$AgNO_3$ (20)	EA	50	6	69%
14	$AgNO_3$ (20)	DMSO	50	24	trace
15	$AgNO_3$ (20)	EA	40	4	72%
16	$AgNO_3$ (20)	EA	30	4	75%
17	$AgNO_3$ (10)	EA	30	5	79%
18	$AgNO_3$ (5)	EA	30	52	53%

Table S5. Reaction conditions：**1ad** (0.2 mmol), diphenylphosphine oxide (0.4 mmol) and silver catalyst in dry solvent (2.0 mL) under Ar atmosphere were stirred at indicated temperature. [a] Yields of isolated products are given.

General procedure for the synthesis of 4

To a 4-mL oven-dried reaction vial was charged with **1** (0.2 mmol), diarylphosphine oxide (2 equiv.), and $AgNO_3$ (10 mol %). The reaction vial was back-flushed with argon three times. Then dry EtOAc (2.0 mL) was added to the reaction vial via syringe, and the reaction mixture was stirred vigorously at 30 °C. After removal of solvents in vacuo, fused diaziridine **4** was isolated by flash column chromatography on silica gel.

2.6 For radical-mediated reductive cyclization (Fig 5)

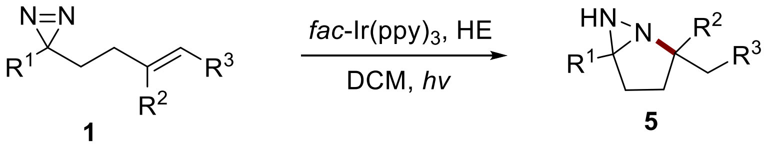

Reaction conditions: **1** (0.2 mmol), *fac*-Ir(ppy)$_3$ (0.004 mmol) and Hantzsch ester (0.4 mmol) in dry DCM (4.0 mL) under Ar irradiated with 5 W × 2 blue LEDs at rt for 4 h. Yields of isolated products are given.

General procedure for the synthesis of 5

To a 8-mL reaction vial was charged with **1** (0.2 mmol), *fac*-Ir(ppy)$_3$ (2 mol %), and Hantzsch ester (2 equiv.). The reaction vial was back-flushed with argon three times. Then DCM (4.0 mL) was added to the reaction vial via syringe. The reaction mixture was vigorously stirred at rt for 4 h under the irradiation of 5 W × 2 blue LEDs (approximately 5 cm away from the light sources). After removal of solvents in vacuo, fused diaziridine **5** was isolated by flash column chromatography on silica gel.

3. Preparation of starting materials

3.1 General method A

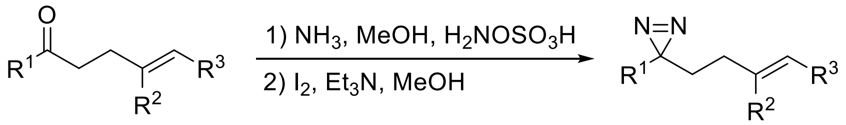

According to the reference,[1] to a flask containing corresponding acetone (10 mmol, 1.0 equiv.) was added NH_3 (7 M in MeOH, 15 equiv., 21.4 mL) at −10 °C. The flask was sealed and the mixture was stirred at −10 ℃ for 4.5 h. Then a solution of hydroxylamine-*O*-sulfonic acid (H_2NOSO_3H, 1.3 equiv., 1.47 g) in anhydrous MeOH (7.4 mL) was added dropwise at −10 ℃ and the reaction mixture was stirred at −10 ℃ for 1 h in a sealed flask. Subsequently, the reaction mixture was allowed to warm to room temperature and stirred for 16 h. NH_3 was removed by gently blowing Ar through the suspension. The precipitate was removed by filtration through celite silica gel and the precipitate was washed with several portions of anhydrous MeOH (10 mL). The organic phase was treated with Et_3N (7.4 equiv., 10.3 mL) and cooled to 0 ℃. I_2 was then added in small portions until a dark brown color persisted in the solution. And the reaction mixture was stirred at 0 ℃ for another 1 h to complete the oxidation of the diaziridine intermediate. The solution was then diluted with Et_2O and the mixture was washed with brine. The aqueous phase was extracted with Et_2O, the combined organic layer was dried over anhydrous Na_2SO_4, filtered and the solvent was removed under reduced pressure. The residue was purified by flash column chromatography on silica to afford the corresponding diazirine.

Substrate **1a**-**1d**, **1h**-**1af** were synthesized according to this method.

3.2 General method B

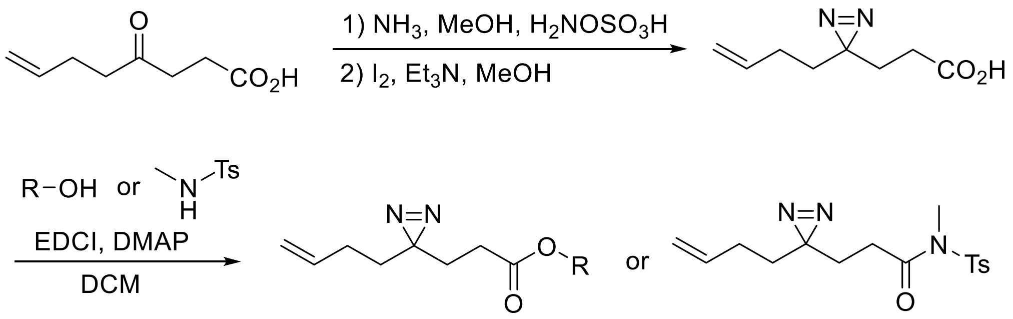

According to the references,[2] to a flask containing 4-oxooct-7-enoic acid (10 mmol, 1.00 equiv.) was added NH_3 (7 M in MeOH, 15 equiv., 21.4 mL) at −10 °C. The flask was sealed and the mixture was stirred at −10 °C for 4.5 h. Then a solution of hydroxylamine-*O*-sulfonic acid (H_2NOSO_3H, 1.3 equiv., 1.47 g) in anhydrous MeOH (7.4 mL) was added dropwise at −10 °C and the reaction mixture was stirred at −10 °C for 1 h in a sealed flask. Subsequently, the reaction mixture was allowed to warm to room temperature and stirred for 16 h. NH_3 was removed by gently blowing Ar through the suspension. The precipitate was removed by filtration through celite silica gel and the precipitate was washed with several portions of anhydrous MeOH (10 mL). The organic phase was treated with Et_3N (7.4 equiv., 10.3 mL) and cooled to 0 ℃. I_2 was then added in small portions until a dark brown color persisted in the solution. And the reaction mixture was stirred at 0 ℃ for another 1 h to complete the oxidation of the diaziridine intermediate. The solution was then diluted with Et_2O and the mixture was washed with 1 M HCl and then brine. The aqueous phase was extracted with Et_2O, the combined organic layer was dried over anhydrous Na_2SO_4, filtered and the solvent was

removed in vacuo. The residue was purified by flash column chromatography on silica gel to afford 3-(3-(but-3-en-1-yl)-3*H*-diazirin-3-yl)propanoic acid.

Next, to a flask containing the above 3-(3-(but-3-en-1-yl)-3H-diazirin-3-yl)propanoic acid (1.0 equiv.), DMAP (10 mol %), alcohol or amine (1.5 equiv.), DCM (0.5 M) was added 1-(3-dimethylaminopropyl)-3-ethylcarbodiimide hydrochloride (EDCI, 1.5 equiv.) at 0 ^{o}C. The reaction mixture was stirred vigorously at room temperature overnight. The solution was then diluted with DCM and the mixture was washed with water. The aqueous phase was extracted with DCM, the combined organic layer was dried over anhydrous Na_2SO_4, filtered and the solvent was removed under reduced pressure. The residue was purified by flash column chromatography on silica gel to afford the corresponding diazirine.

Substrates **1e**-**1g** were synthesized according to this method.

4. Product transformations

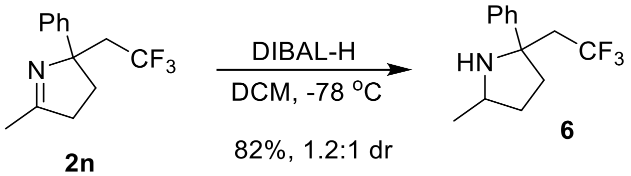

2n **6**

To a solution of **2n** (0.2 mmol, 48.3 mg) in DCM (2.0 mL) was added dropwise DIBAL-H (0.8 mmol, 4.0 equiv., 0.53 mL, 1.5 M in toluene) at -78 ^{o}C under N_2 atmosphere. The mixture was stirred at -78 ^{o}C for 4 h. The reaction was then quenched by adding a saturated NH_4Cl aqueous solution and diluted with Roche salt aqueous solution. The aqueous phase was extracted with DCM and combined organic layer was dried over anhydrous Na_2SO_4, filtered. After removal of the solvent in vacuo, compound **6** was isolated by flash column chromatography on silica gel.

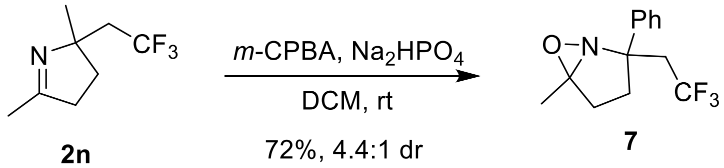

2n **7**

To a screw-cap vial equipped with a magnetic stirring bar were added **2n** (0.2 mmol, 48.3 mg), *m*-CPBA (0.3 mmol, 60.9 mg, 1.5 equiv.), Na_2HPO_4 (0.3 mmol, 42.6 mg, 1.5 equiv.) and DCM (2.0 mL). The mixture was stirred at rt for 2 h. The reaction was then diluted with water, extracted with DCM, and combined organic layer was dried over anhydrous Na_2SO_4, filtered. The solvent was removed in vacuo, and the crude product was purified by flash column chromatography on silica gel to generate product **7**.

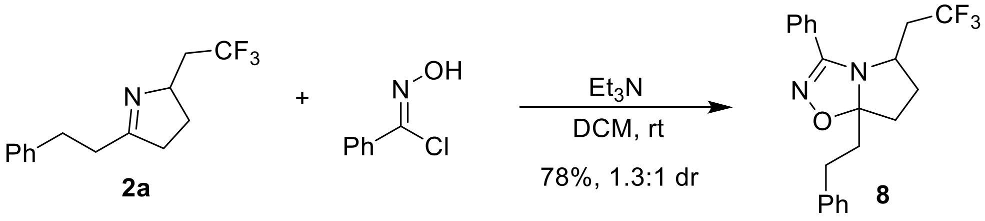

To a screw-cap vial equipped with a magnetic stirring bar were added **2a** (0.1 mmol, 25.5 mg), *N*-hydroxybenzimidoyl chloride (0.15 mmol, 23.3 mg, 1.5 equiv.), E_3N (0.2 mmol, 28.0 μL, 2.0 equiv.) and DCM (2.0 mL). The mixture was stirred at rt for 3 h. The solvent was removed in vacuo, and the crude product was purified by flash column chromatography on silica gel to give product **8**.

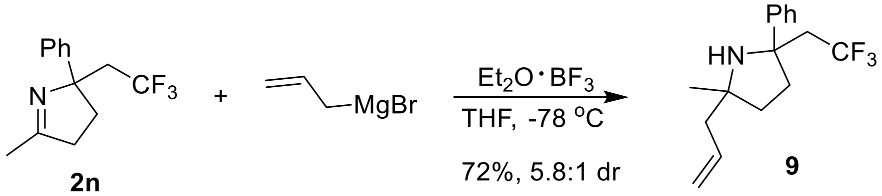

To a solution of **2n** (0.15 mmol, 48.3 mg) in THF (5.0 mL) at -78 °C under N_2 atmosphere, $Et_2O\cdot BF_3$ (0.45 mmol, 56.8 μL, 3.0 equiv.) was added followed by the dropwise addition of allyl magnesium bromide (0.6 mmol, 3.0 equiv., 0.45 mL, 1.0 M in Et_2O). The reaction mixture was stirred at -78 °C for 4 h and then quenched by adding a saturated NH_4Cl aqueous solution. The aqueous phase was extracted with Et_2O and combined organic layer was dried over anhydrous Na_2SO_4, filtered. After removal of the solvent in vacuo, compound **9** was isolated by flash column chromatography on silica gel.

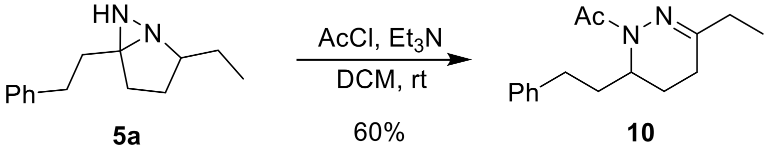

To a screw-cap vial equipped with a magnetic stirring bar were added **5a** (0.2 mmol, 43.3 mg), E_3N (0.6 mmol, 41.6 μL, 3.0 equiv.), AcCl (0.6 mmol, 21.3 μL, 3.0 equiv.), and DCM (2.0 mL). The reaction mixture was stirred at rt for 3 h. The reaction was then diluted with water, extracted with DCM, and combined organic layer was dried over anhydrous Na_2SO_4, filtered. The solvent was removed in vacuo, and the crude product was purified by flash column chromatography on silica gel to give product **10**.

5. Mechanistic studies

5.1 Identification of intermediates (Fig 7)

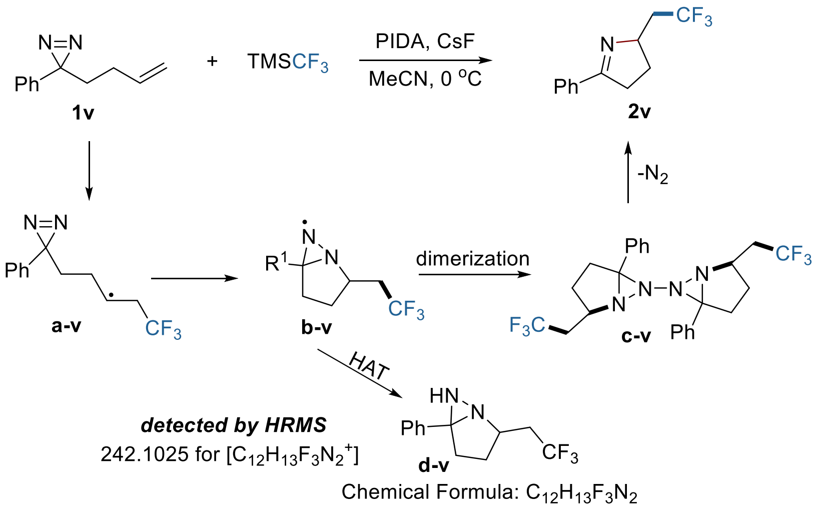

Fig. S1. Identification of intermediate.

Substrate **1v** was treated under standard conditions. After stirred under 0 °C for 5 min, the reaction mixture was used for the HRMS detection directly. Intermediate **d-v** calculated for [$C_{12}H_{13}F_3N_2^+$] 242.1025, found 242.1036.

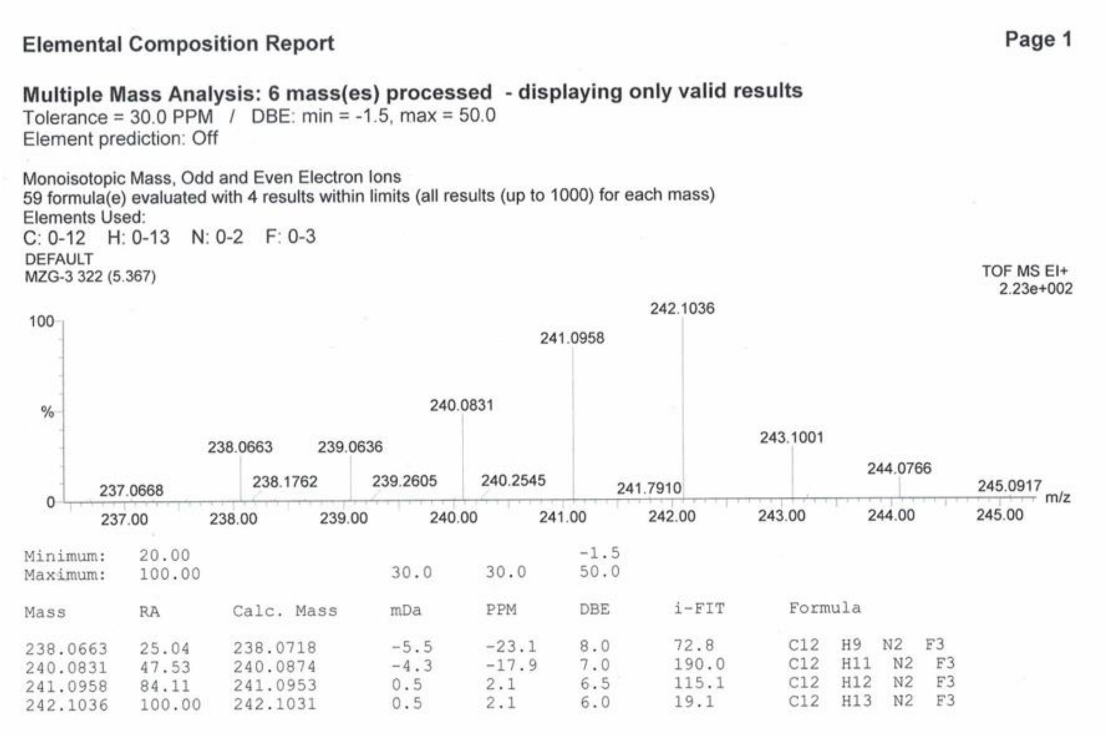

Elemental Composition Report

Page 1

Multiple Mass Analysis: 6 mass(es) processed - displaying only valid results

Tolerance = 30.0 PPM / DBE: min = -1.5, max = 50.0

Element prediction: Off

Monoisotopic Mass, Odd and Even Electron Ions

59 formula(e) evaluated with 4 results within limits (all results (up to 1000) for each mass)

Elements Used:

C: 0-12 H: 0-13 N: 0-2 F: 0-3

DEFAULT

MZG-3 322 (5.367)

TOF MS EI+

2.23e+002

Minimum:	20.00				-1.5		
Maximum:	100.00		30.0	30.0	50.0		
Mass	RA	Calc. Mass	mDa	PPM	DBE	i-FIT	Formula
238.0663	25.04	238.0718	-5.5	-23.1	8.0	72.8	C12 H9 N2 F3
240.0831	47.53	240.0874	-4.3	-17.9	7.0	190.0	C12 H11 N2 F3
241.0958	84.11	241.0953	0.5	2.1	6.5	115.1	C12 H12 N2 F3
242.1036	100.00	242.1031	0.5	2.1	6.0	19.1	C12 H13 N2 F3

Fig. S2. HRMS of **d-v**.

Intermediate **5a-g** was isolated in the following reaction (standard conditions in Fig. 5).

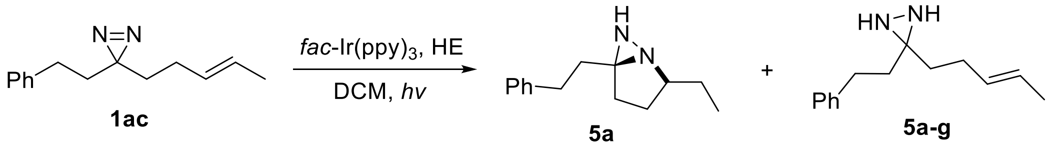

5a-g, colorless oil. **^{1}H NMR** (400 MHz, $CDCl_3$) δ 7.29-7.25 (m, 2H), 7.20-7.16 (m, 3H), 5.52-5.38 (m, 2H), 2.73-2.69 (m, 2H), 2.12 (q, *J* = 7.2 Hz, 2H), 1.94-1.88 (m, 2H), 1.71 (q, *J* = 8.4 Hz, 2H), 1.65 (d, *J* = 6.0 Hz, 3H). **^{13}C NMR** (100 MHz, $CDCl_3$) δ 141.1, 129.9, 128.5, 128.1, 126.1, 125.9, 57.3, 38.1, 36.0, 30.9, 27.8, 17.8. **FT-IR**: ν (cm^{-1}) 2931, 1453, 1133, 965, 747, 698. **HRMS** [EI] calcd for $C_{14}H_{20}N_2[M]^+$ 216.1621, found 216.1630.

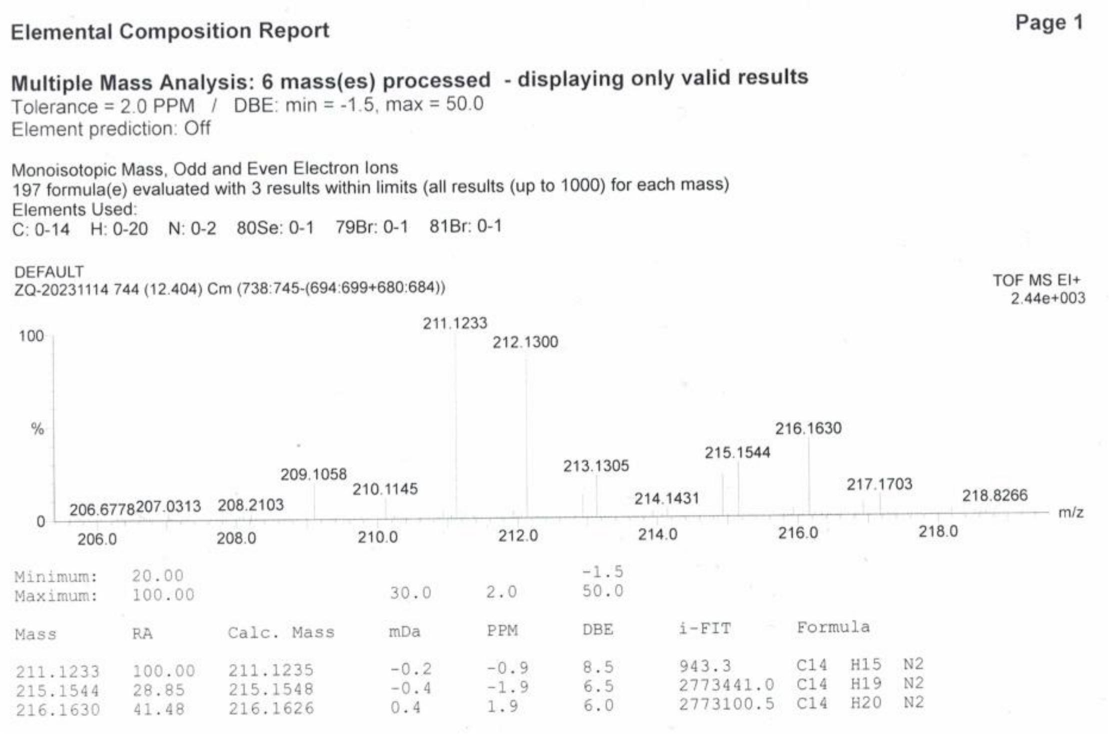

Fig. S3. HRMS of **5a-g**.

5.2 Cyclic voltammogram experiment

All voltammograms were taken at room temperature using a mesh platinum (Pt) counter electrode, a glassy carbon working electrode, and a saturated calomel (SCE) reference electrode. The conditions of the experiments were the following: an acetonitrile solution of 0.1 M tetrabutylammonium tetrafluoroborate (Bu_4NBF_4) and 0.01 M **1ac**, a scan rate of 0.1 V/s, and a negative initial scan direction. The reported potentials were averages over segments, and were taken at half-height of the cathodic peaks (Ep/2) of **1ac**, since all reductions were nonreversible.

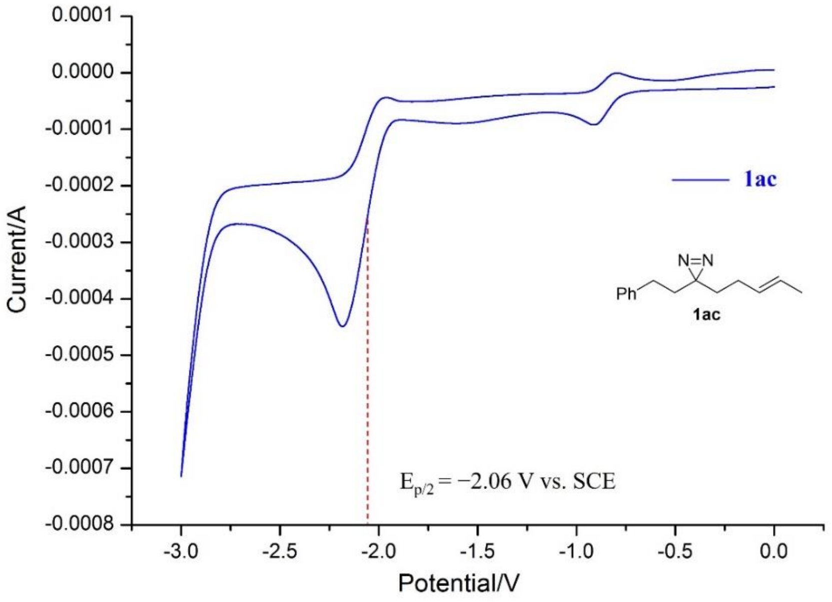

Fig. S4. cyclic voltammogram experiment.

We can conclude from the cyclic voltammogram experiment that $E_{p/2}$ of **1ac** is -2.06 V vs. SCE.

5.3 **DFT calculations**

Computational methods

All theoretical calculations were performed with Gaussian 09. Geometry optimizations and frequency computations were calculated by B3LYP functional together with the 6-31G(d) basis set.

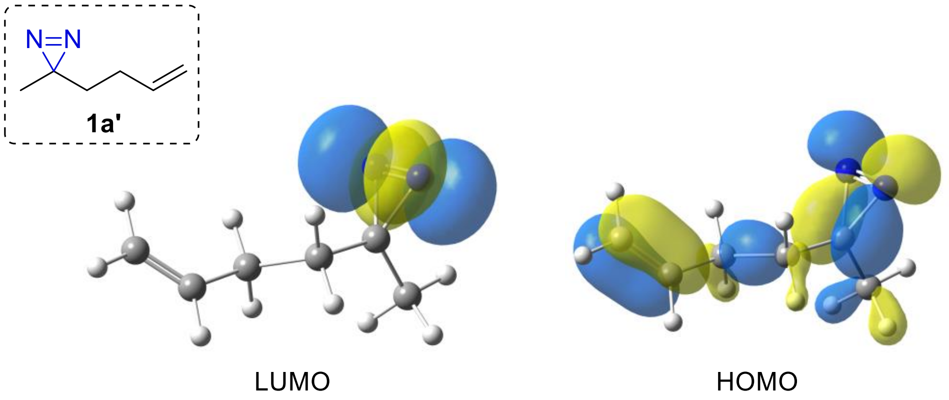

Fig. S5. LUMO and HOMO analysis of diazirine substrate

DFT-calculated Cartesian Coordinates.

1a’

C	-0.03150400	0.07909300	-0.66827500

H	-0.24006000	-0.86786400	-1.17950800
H	-0.10042600	0.86890400	-1.42975900
C	-1.10734800	0.31265500	0.41814700
H	-0.88173000	1.25038900	0.94724100
H	-1.05210600	-0.49041900	1.16312600
C	-2.49580300	0.38621100	-0.15644400
H	-2.67160600	1.19145300	-0.87257400
C	-3.49140800	-0.45222400	0.13259300
H	-4.47435500	-0.35233100	-0.31998100
H	-3.36230400	-1.27080700	0.83811500
C	1.37997700	0.04965300	-0.11845000
N	1.78686000	-1.16215600	0.63546500
N	2.24124500	-1.09137700	-0.50960400
C	2.05968200	1.35258600	0.22913200
H	2.17628100	1.98003500	-0.66377300
H	1.47576500	1.92191600	0.96267800
H	3.05223500	1.17560700	0.65319700

6. References

1. Pan, S., Jang, S.-Y., Wang, D., Liew, S. S., Li, Z., Lee, J.-S. & Yao, S. Q. A suite of "Minimalist" photo-crosslinkers for live-cell imaging and chemical proteomics: case study with BRD4 inhibitors. *Angew. Chem. Int. Ed.* 2017, **56**, 11816-11821.
2. (a) Kambe, T., Correia, B. E., Niphakis, M. J. & Cravatt, B. F. Mapping the protein interaction landscape for fully functionalized small-molecule probes in human cells. *J. Am. Chem. Soc.* **136**, 10777−10782 (2014); (b) Kleiner, P., Heydenreuter, W., Stahl, M., Korotkov, V. S. & Siebe, S. A. A Whole proteome inventory of background photocrosslinker binding. *Angew. Chem. Int. Ed.* 2017, **56**, 1396–1401.

7. Characterization of starting materials and products

7.1. Starting materials

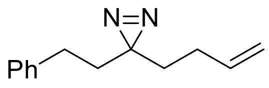

1a, colorless oil. **^{1}H NMR** (400 MHz, $CDCl_3$) δ 7.29-7.24 (m, 2H), 7.20-7.13 (m, 3H), 5.77-5.67 (m, 1H), 5.02-4.96 (m, 2H), 2.40 (t, *J* = 8.4 Hz, 2H), 1.87-1.82 (m, 2H), 1.72-1.68 (m, 2H), 1.46 (t, *J* = 8.0 Hz, 2H); **^{13}C NMR** (100 MHz, $CDCl_3$) δ 140.8, 137.1, 128.5, 128.3, 126.2, 115.4, 35.0, 32.4, 30.0, 28.5, 28.0. **FT-IR**: ν (cm^{-1}) 2923, 2855, 2360, 1583, 1497, 1453, 995, 914, 739, 697. **HRMS** [ESI] calcd for $C_{13}H_{16}N_2Na$ $[M+Na]^+$ 223.1206, found 223.1204.

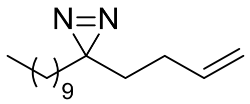

1b, colorless oil. **^{1}H NMR** (400 MHz, $CDCl_3$) δ 5.79-5.69 (m, 1H), 5.04-4.96 (m, 2H), 1.88-1.83 (m, 2H), 1.46 (t, *J* = 7.8 Hz, 2H), 1.36 (t, *J* = 8.0 Hz, 2H), 1.31-1.24 (m, 15H), 1.09-1.07 (m,

2H), 0.88 (t, *J* = 6.6 Hz, 2H); **^{13}C NMR** (100 MHz, $CDCl_3$) δ 137.2, 115.2, 32.9, 32.4, 31.9, 29.5, 29.5, 29.4, 29.3, 29.2, 28.6, 28.0, 23.8, 22.7, 14.1. **FT-IR**: ν (cm^{-1}) 2924, 2854, 2360, 2341, 1584, 1465, 993, 913, 721. **HRMS** [ESI] calcd for $C_{15}H_{29}N_2$ $[M+H]^+$ 237.2325, found 237.2335.

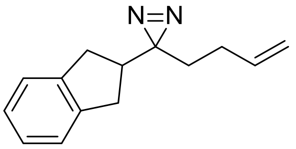

1c, colorless oil. **^{1}H NMR** (400 MHz, $CDCl_3$) δ 7.16-7.11 (m, 4H), 5.79-5.69 (m, 1H), 5.05-4.98 (m, 2H), 2.84 (dd, *J* =14.8, 8.0 Hz, 2H), 2.72-2.64 (m, 1H), 2.58 (dd, *J* = 14.8, 8.0 Hz, 2H), 1.87-1.81 (m, 2H), 1.62-1.57 (m, 2H); **^{13}C NMR** (100 MHz, $CDCl_3$) δ 141.7, 137.0, 126.5, 124.3, 115.4, 42.5, 34.2, 31.4, 30.3, 27.8. **FT-IR**: ν (cm^{-1}) 2937, 2849, 2360, 2342, 1642, 1577, 1483, 1459, 996, 913, 742. **HRMS** [ESI] calcd for $C_{14}H_{16}N_2Na$ $[M+Na]^+$ 235.1206, found 235.1205.

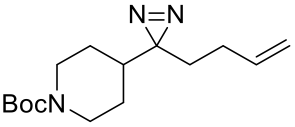

1d, colorless oil. **^{1}H NMR** (400 MHz, $CDCl_3$) δ 5.74-5.64 (m, 1H), 5.02-4.96 (m, 2H), 4.14-3.97 (m, 2H), 2.57 (t, *J* = 12.0 Hz, 2H), 1.77-1.71 (m, 2H), 1.61-1.44 (m, 5H), 1.42 (s, 9H), 0.97-0.85(m, 2H); **^{13}C NMR** (100 MHz, $CDCl_3$) δ 154.6, 136.9, 115.4, 79.5, 38.8, 30.6, 30.2, 28.4, 27.6, 27.1. **FT-IR**: ν (cm^{-1}) 2977, 2933, 2855, 1689, 1419, 1365, 1239, 1154, 1011, 914. **HRMS** [ESI] calcd for $C_{15}H_{25}N_3O_2Na$ $[M+Na]^+$ 302.1839, found 302.1831.

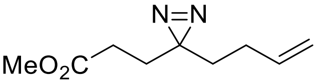

1e, colorless oil. **^{1}H NMR** (400 MHz, $CDCl_3$) δ 5.78-5.67 (m, 1H), 5.04-4.97 (m, 2H), 3.67 (s, 3H), 2.11 (t, *J* = 7.6 Hz, 2H), 1.89-1.83 (m, 2H), 1.75 (t, *J* = 7.6 Hz, 2H), 1.52-1.48 (m, 2H); **^{13}C NMR** (100 MHz, $CDCl_3$) δ 172.7, 136.8, 115.5, 51.8, 32.2, 28.3, 28.2, 27.9, 27.8. **FT-IR**: ν (cm^{-1}) 2953, 2361, 2342, 1738, 1584, 1437, 1362, 1314, 1257, 1197, 1172, 995, 915. **HRMS** [ESI] calcd for $C_9H_{14}N_2NaO_2$ $[M+Na]^+$ 205.0947, found 205.0949.

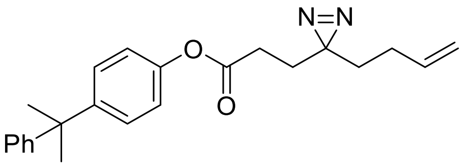

1f, colorless oil. **^{1}H NMR** (400 MHz, $CDCl_3$) δ 7.29-7.15 (m, 7H), 7.00-6.96 (m, 2H), 5.79-5.69 (m, 1H), 5.06-4.98 (m, 2H), 2.35 (t, *J* = 7.6 Hz, 2H), 1.91-1.84 (m, 4H), 1.67 (s, 6H), 1.57-1.53 (m, 2H); **^{13}C NMR** (100 MHz, $CDCl_3$) δ 170.9, 150.2, 148.3, 136.8, 128.0, 127.8, 126.7, 125.7, 120.8, 120.7, 115.6, 42.7, 32.2, 30.8, 28.7, 28.2, 27.9, 27.8. **FT-IR**: ν (cm^{-1}) 2954, 2363, 2343, 1757, 1505, 1204, 1170, 1136, 1017, 914, 763, 700. **HRMS** [ESI] calcd for $C_{23}H_{26}N_2NaO_2$ $[M+Na]^+$ 385.1886, found 385.1885.

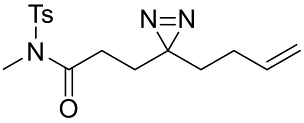

1g, colorless oil. **^{1}H NMR** (400 MHz, $CDCl_3$) δ 7.74 (d, *J* = 8.2 Hz, 2H), 7.34 (d, *J* = 8.2 Hz, 2H), 5.73-5.63 (m, 1H), 5.00-4.94 (m, 2H), 3.25 (s, 3H), 2.46-2.42 (m, 5H), 1.83-1.77 (m, 2H), 1.71 (t, *J* = 7.6 Hz, 2H), 1.42 (t, *J* = 7.6 Hz, 2H); **^{13}C NMR** (100 MHz, $CDCl_3$) δ 171.6, 145.0, 136.8, 135.9, 129.9, 127.3, 115.4, 33.0, 32.2,

30.7, 27.8, 27.7, 21.5. **FT-IR**: ν (cm^{-1}) 2926, 2361, 2342, 1698, 1354, 1160, 1085, 989, 917, 813, 713, 665. **HRMS** [ESI] calcd for $C_{16}H_{21}N_3NaO_3S$ $[M+Na]^+$ 358.1196, found 358.1210.

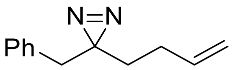

1h, colorless oil. **^{1}H NMR** (400 MHz, $CDCl_3$) δ 7.33-7.17 (m, 5H), 5.75-5.64 (m, 1H), 5.01-4.95 (m, 2H), 2.65 (s, 2H), 1.86-1.80 (m, 2H), 1.53-1.49 (m, 2H); **^{13}C NMR** (100 MHz, $CDCl_3$) δ 137.0, 135.4, 129.4, 128.6, 126.9, 115.4, 40.1, 31.7, 29.1, 27.8. **FT-IR**: ν (cm^{-1}) 2916, 2360, 1642, 1583, 1496, 1454, 1437, 995, 913, 730, 698. **HRMS** [ESI] calcd for $C_{12}H_{14}N_2Na$ $[M+Na]^+$ 209.1049, found 209.1058.

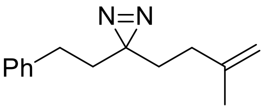

1i, colorless oil. **^{1}H NMR** (400 MHz, $CDCl_3$) δ 7.31-7.15 (m, 5H), 4.73 (s, 1H), 4.65 (s, 1H), 2.44-2.40 (m, 2H), 1.81-1.70 (m, 4H), 1.67 (s, 3H), 1.54-1.50 (m, 2H); **^{13}C NMR** (100 MHz, $CDCl_3$) δ 144.2, 140.8, 128.5, 128.2, 126.1, 110.5, 34.9, 31.6, 31.2, 30.0, 28.6, 22.4. **FT-IR**: ν (cm^{-1}) 2921, 2857, 2361, 2341, 1650, 1580, 1497, 1453, 889, 739, 697. **HRMS** [ESI] calcd for $C_{14}H_{18}N_2Na$ $[M+Na]^+$ 237.1362, found 237.1364.

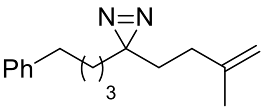

1j, colorless oil. **^{1}H NMR** (400 MHz, $CDCl_3$) δ 7.29-7.25 (m, 2H), 7.19-7.14 (m, 3H), 4.72 (s, 1H), 4.64 (s, 1H), 2.56 (t, *J* = 7.8 Hz, 2H), 1.80-1.76 (m, 2H), 1.66 (s, 3H), 1.61-1.53 (m, 2H), 1.51-1.47 (m, 2H), 1.40 (t, *J* = 7.4 Hz, 2H), 1.18-1.10 (m, 2H); **^{13}C NMR** (100 MHz, $CDCl_3$) δ 144.3, 142.2, 128.3, 128.3, 125.7, 110.4, 35.7, 32.7, 31.6, 31.2, 30.9, 28.6, 23.5, 22.4. **FT-IR**: ν (cm^{-1}) 2934, 2858, 1650, 1582, 1496, 1453, 1375, 888, 744, 698. **HRMS** [ESI] calcd for $C_{16}H_{22}N_2Na$ $[M+Na]^+$ 265.1675, found 265.1683.

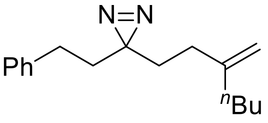

1k, colorless oil. **^{1}H NMR** (400 MHz, $CDCl_3$) δ 7.31-7.27 (m, 2H), 7.22-7.15 (m, 3H), 4.73 (s, 1H), 4.66 (s, 1H), 2.44-2.40 (m, 2H), 1.94 (t, *J* = 7.4 Hz, 2H), 1.79-1.69 (m, 4H), 1.55-1.19 (m, 2H), 1.38-1.26 (m, 4H), 0.90 (t, *J* = 7.0 Hz, 3H); **^{13}C NMR** (100 MHz, $CDCl_3$) δ 148.3, 140.8, 128.5, 128.2, 126.1, 109.3, 35.8, 35.0, 31.3, 30.0, 29.9, 29.8, 28.6, 22.4, 13.9. **FT-IR**: ν (cm^{-1}) 2955, 2928, 2859, 2361, 2342, 1646, 1582, 1454, 889, 739, 697. **HRMS** [ESI] calcd for $C_{17}H_{25}N_2$ $[M+H]^+$ 257.2012, found 257.2017.

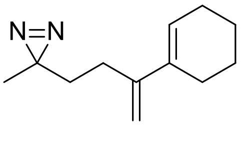

1l, colorless oil. **^{1}H NMR** (400 MHz, $CDCl_3$) δ 5.80 (t, *J* = 3.8 Hz, 1H), 4.98 (s, 1H), 4.80 (s, 1H), 2.14-2.09 (m, 6H), 1.69-1.63 (m, 2H), 1.59-1.55 (m, 2H), 1.53-1.49 (m, 2H), 1.02 (s, 3H); **^{13}C NMR** (100 MHz, $CDCl_3$) δ 147.1, 135.3, 124.4, 109.2, 34.0, 27.8, 26.0, 25.9, 25.8, 22.9, 22.1, 19.9. **FT-IR**: ν (cm^{-1}) 2927, 2859, 1604, 1448, 1384, 919, 886, 851, 803. **HRMS** [ESI] calcd for $C_{12}H_{18}KN_2$ $[M+K]^+$ 229.1102, found 229.1092.

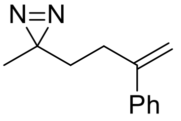

1m, colorless oil. **^{1}H NMR** (400 MHz, $CDCl_3$) δ 7.37-7.25 (m, 5H), 5.29 (s, 1H), 5.07 (q, *J* = 1.2 Hz, 1H), 2.39-2.35 (m, 2H), 1.54-1.50 (m, 2H), 1.02 (s, 3H); **^{13}C NMR** (100 MHz, $CDCl_3$) δ 147.1, 140.6, 128.4,

127.5, 126.0, 112.9, 33.2, 29.7, 25.7, 19.9. **FT-IR**: ν (cm^{-1}) 2924, 2859, 2360, 1628, 1575, 1494, 1444, 1385, 1028, 897, 777, 700. **HRMS** [ESI] calcd for $C_{12}H_{14}N_2Na$ $[M+Na]^+$ 209.1049, found 209.1058.

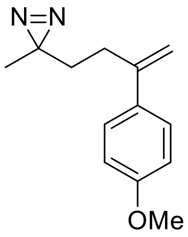

1n, colorless oil. **^{1}H NMR** (400 MHz, $CDCl_3$) δ 7.31-7.28 (m, 2H), 6.87-6.84 (m, 2H), 5.23 (s, 1H), 4.99 (d, *J* = 0.8 Hz, 1H), 3.81 (s, 3H), 2.34 (t, *J* = 8.0 Hz, 2H), 1.53-1.49 (m, 2H), 1.01 (s, 3H); **^{13}C NMR** (100 MHz, $CDCl_3$) δ 159.1, 146.3, 133.0, 127.1, 113.7, 111.3, 55.2, 33.2, 29.7, 25.7, 19.9. **FT-IR**: ν (cm^{-1}) 2952, 2837, 2361, 2342, 1608, 1511, 1456, 1287, 1246, 1179, 1033, 891, 834, 810. **HRMS** [ESI] calcd for $C_{13}H_{16}N_2NaO$ $[M+Na]^+$ 239.1155, found 239.1153.

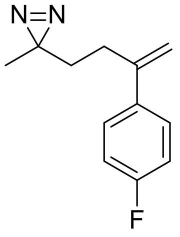

1o, colorless oil. **^{1}H NMR** (400 MHz, $CDCl_3$) δ 7.34-7.28 (m, 2H), 7.03-6.97 (m, 2H), 5.23 (s, 1H), 5.05 (d, *J* = 1.2 Hz, 1H), 2.35-2.31 (m, 2H), 1.52-1.48 (m, 2H), 1.00 (s, 3H); **^{13}C NMR** (100 MHz, $CDCl_3$) δ 162.3 (d, $J_{C\text{-}F}$ = 244.9 Hz), 146.1, 136.7 (d, $J_{C\text{-}F}$ = 3.4 Hz), 127.6 (d, $J_{C\text{-}F}$ = 7.9 Hz), 115.2 (d, $J_{C\text{-}F}$ = 21.2 Hz), 112.9, 33.1, 29.8, 25.6, 19.8; **^{19}F NMR** (376 MHz, $CDCl_3$) δ −115.0 (s). **FT-IR**: ν (cm^{-1}) 2925, 1602, 1509, 1452, 1229, 1161, 898, 838. **HRMS** [EI] calcd for $C_{12}H_{13}FN_2$ $[M]^+$ 204.1057, found 204.1059.

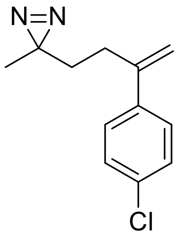

1p, colorless oil. **^{1}H NMR** (400 MHz, $CDCl_3$) δ 7.31-7.27 (m, 4H), 5.28 (s, 1H), 5.08 (d, *J* = 1.2 Hz, 1H), 2.33 (td, *J* = 8.0, 1.2 Hz, 2H), 1.52-1.48 (m, 2H), 1.01 (s, 3H); **^{13}C NMR** (100 MHz, $CDCl_3$) δ 146.0, 139.0, 133.3, 128.5, 127.3, 113.5, 33.1, 29.6, 25.6, 19.9. **FT-IR**: ν (cm^{-1}) 2949, 2924, 2360, 2342, 1492, 1096, 1012, 899, 834, 736. **HRMS** [EI] calcd for $C_{12}H_{13}ClN_2$ $[M]^+$ 220.0762, found 220.0769.

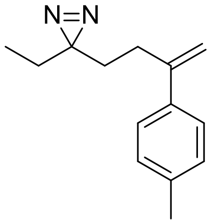

1q, colorless oil. **^{1}H NMR** (400 MHz, $CDCl_3$) δ 7.25-7.23 (m, 2H), 7.12 (d, *J* = 8.0 Hz, 2H), 5.25 (s, 1H), 5.00 (s, 1H), 2.34 (s, 3H), 2.31-2.25 (m, 2H), 1.56-1.50 (m, 2H), 1.42 (q, *J* = 7.6 Hz, 2H), 0.66 (t, *J* = 7.6 Hz, 3H); **^{13}C NMR** (100 MHz, $CDCl_3$) δ 146.9, 137.6, 137.3, 129.0, 125.8, 112.0, 31.6, 29.5, 29.4, 25.9, 21.1, 8.2. **FT-IR**: ν (cm^{-1}) 2970, 2923, 2360, 2342, 1626, 1576, 1514, 1457, 894, 824, 734. **HRMS** [ESI] calcd for $C_{14}H_{18}N_2Na$ $[M+Na]^+$ 237.1362, found 237.1370.

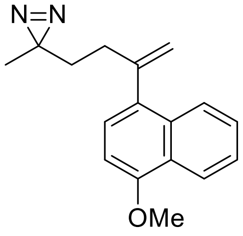

1r, colorless oil. **^{1}H NMR** (400 MHz, $CDCl_3$) δ 8.31-8.28 (m, 1H), 7.94-7.90 (m, 1H), 7.52-7.45 (m, 2H), 7.16 (d, *J* = 7.6 Hz, 1H), 6.77 (d, *J* = 7.6 Hz, 1H), 5.36 (d, *J* = 1.6 Hz, 1H), 5.08 (d, *J* = 1.6 Hz, 1H), 4.01 (s, 3H), 2.35 (t, *J* = 8.0 Hz, 2H), 1.52-1.48 (m, 2H), 0.98 (s, 3H); **^{13}C NMR** (100 MHz, $CDCl_3$) δ 154.7, 147.3, 132.7, 132.1, 126.4, 125.6, 125.3, 125.0, 125.0, 122.2, 115.9, 103.0, 55.5, 33.0, 32.9, 25.7, 19.8. **FT-IR**: ν (cm^{-1}) 2938, 2839, 1585, 1462, 1236, 1088, 903, 819, 765. **HRMS** [EI] calcd for $C_{17}H_{18}N_2O$ [M] 266.1419, found 266.1423.

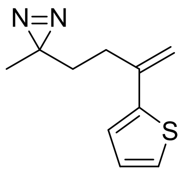

1s, colorless oil. **^{1}H NMR** (400 MHz, $CDCl_3$) δ 7.17 (d, *J* = 4.8 Hz, 1H), 6.98-6.96 (m, 2H), 5.41 (s, 1H), 4.97 (s, 1H), 2.35 (t, *J* = 8.0 Hz, 2H), 1.66-1.62 (m, 2H), 1.05 (s, 3H); **^{13}C NMR** (100 MHz, $CDCl_3$) δ 144.6, 140.3, 127.4, 124.3, 123.3, 111.4, 33.5, 29.7, 25.6, 19.9. **FT-IR**: ν (cm^{-1}) 2924, 1619, 1440, 1227, 884, 829, 695. **HRMS** [EI] calcd for $C_{10}H_{12}N_2S$ $[M]^+$ 192.0716, found 192.0724.

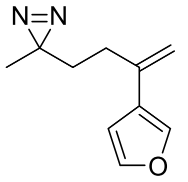

1t, colorless oil. **^{1}H NMR** (400 MHz, $CDCl_3$) δ 7.40 (s, 1H), 7.36 (t, *J* = 1.8 Hz, 1H), 6.50-6.49 (m, 1H), 5.25 (s, 1H), 4.93 (d, *J* = 1.2 Hz, 1H), 2.21-2.17 (m, 2H), 1.63-1.59 (m, 2H), 1.04 (s, 3H); **^{13}C NMR** (100 MHz, $CDCl_3$) δ 143.3, 138.6, 138.1, 126.3, 110.6, 108.1, 33.2, 29.0, 25.7, 19.9. **FT-IR**: ν (cm^{-1}) 2925, 1637, 1450, 1165, 1070, 1025, 887, 872, 790, 734. **HRMS** [ESI] calcd for $C_{10}H_{12}KN_2O$ $[M+K]^+$ 215.0581, found 215.0586.

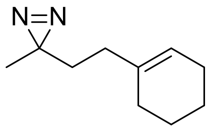

1u, colorless oil. **^{1}H NMR** (400 MHz, $CDCl_3$) δ 5.39-5.39 (m, 1H), 1.97-1.96 (m, 2H), 1.84-1.77 (m, 4H), 1.62-1.50 (m, 4H), 1.44-1.40 (m, 2H), 0.99 (s, 3H); **^{13}C NMR** (100 MHz, $CDCl_3$) δ 136.2, 121.5, 32.7, 32.1, 28.2, 25.9, 25.2, 22.9, 22.4, 19.8. **FT-IR**: ν (cm^{-1}) 2925, 2857, 2836, 1590, 1448, 1439, 1384, 919, 801. **HRMS** [EI] calcd for $C_{10}H_{16}N_2$ $[M]^+$ 164.1308, found 164.1311.

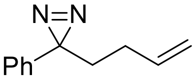

1v, colorless oil. **^{1}H NMR** (400 MHz, $CDCl_3$) δ 7.34-7.24 (m, 3H), 6.96-6.93 (m, 2H), 5.84-5.74 (m, 1H), 5.07-4.99 (m, 2H), 2.09-1.98 (m, 4H); **^{13}C NMR** (100 MHz, $CDCl_3$) δ 138.8, 136.8, 128.3, 127.4, 125.6, 115.5, 29.6, 29.0, 28.1. **FT-IR**: ν (cm^{-1}) 2928, 1596, 1498, 1450, 991, 912, 749, 694. **HRMS** [EI] calcd for $C_{11}H_{12}N_2$ $[M]^+$ 172.0995, found 172.0996.

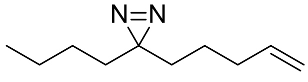

1w, colorless oil. **^{1}H NMR** (400 MHz, $CDCl_3$) δ 5.76-5.66 (m, 1H), 5.00-4.92 (m, 2H), 2.01-1.95 (m, 2H), 1.37-1.32 (m, 4H), 1.27-1.14 (m, 4H), 1.09-1.01 (m, 2H), 0.84 (t, *J* = 7.2 Hz, 3H); **^{13}C NMR** (100 MHz, $CDCl_3$) δ 137.9, 115.0, 33.1, 32.6, 32.3, 28.7, 25.9, 23.1, 22.3, 13.8. **FT-IR**: ν (cm^{-1}) 2956, 2861, 1711, 1457, 1252, 1115, 911, 746. **HRMS** [ESI] calcd for $C_{10}H_{19}N_2$ $[M+H]^+$ 167.1543, found 167.1549.

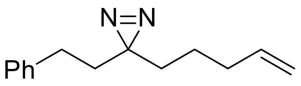

1x, colorless oil. **^{1}H NMR** (400 MHz, $CDCl_3$) δ 7.31-7.28 (m, 2H), 7.23-7.15 (m, 3H), 5.78-5.68 (m, 1H), 5.03-4.96 (m, 2H), 2.43 (t, *J* = 8.4 Hz, 2H), 2.00 (q, *J* = 7.1 Hz, 2H), 1.73-1.69 (m, 2H), 1.39 (t, *J* = 7.6 Hz, 2H), 1.25-1.17 (m, 2H); **^{13}C NMR** (100 MHz, $CDCl_3$) δ 140.7, 137.8, 128.4, 128.2, 126.1, 115.1, 35.0, 33.0, 32.2, 29.9, 28.6, 23.0. **FT-IR**: ν (cm^{-1}) 2934, 2860, 1583, 1454, 992, 912, 736, 697. **HRMS** [ESI] calcd for $C_{14}H_{18}N_2Na$ $[M+Na]^+$ 237.1362, found 237.1365.

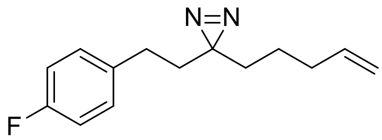

1y, colorless oil. **^{1}H NMR** (400 MHz, $CDCl_3$) δ 7.12-7.09 (m, 2H), 6.99-6.94 (m, 2H), 5.77-5.67 (m, 1H), 5.02-4.95 (m, 2H), 2.40-2.36 (m, 2H), 2.02-1.96 (m, 2H), 1.69-1.65 (m, 2H), 1.39-1.35 (m, 2H), 1.23-1.15 (m, 2H); **^{13}C NMR** (100 MHz, $CDCl_3$) δ 161.3 (d, $J_{C\text{-}F}$ = 242.6 Hz), 137.7, 136.3 (d, $J_{C\text{-}F}$ = 3.3 Hz), 129.6 (d, $J_{C\text{-}F}$ = 7.8 Hz), 115.2 (d, $J_{C\text{-}F}$ = 20.1 Hz), 115.1, 35.0, 33.0, 32.2, 29.1, 28.4, 23.0; **^{19}F NMR** (376 MHz, $CDCl_3$) δ −117.1 (s). **FT-IR**: ν (cm^{-1}) 2930, 1509, 1222, 1158, 913, 825. **HRMS** [ESI] calcd for $C_{14}H_{18}FN_2$ $[M+H]^+$ 233.1449, found 233.1450.

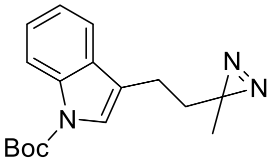

1z, colorless oil. **^{1}H NMR** (400 MHz, $CDCl_3$) δ 8.11 (d, J = 6.4 Hz, 1H), 7.46 (d, J = 8.0 Hz, 1H), 7.36-7.28 (m, 2H), 7.24-7.20 (m, 1H), 2.56-2.52 (m, 2H), 1.80-1.76 (m, 2H), 1.66 (s, 9H), 1.06 (s, 3H); **^{13}C NMR** (100 MHz, $CDCl_3$) δ 149.7, 135.5, 130.2, 124.4, 122.4, 122.3, 119.5, 118.7, 115.3, 83.4, 33.9, 28.2, 25.7, 19.9, 19.4. **FT-IR**: ν (cm^{-1}) 2979, 1728, 1452, 1369, 1253, 1154, 1082, 766, 743. **HRMS** [ESI] calcd for $C_{17}H_{21}N_3NaO_2$ $[M+Na]^+$ 322.1526, found 322.1516.

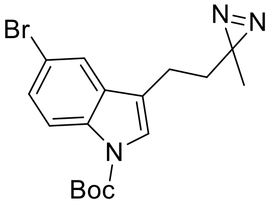

1aa, white solid, mp. 75-76 °C. **^{1}H NMR** (400 MHz, $CDCl_3$) δ 7.98 (d, J = 6.4 Hz, 1H), 7.57 (d, J = 1.6 Hz, 1H), 7.40-7.35 (m, 2H), 2.50-2.46 (m, 2H), 1.79-1.75 (m, 2H), 1.66 (s, 9H), 1.06 (s, 3H); **^{13}C NMR** (100 MHz, $CDCl_3$) δ 149.3, 134.2, 131.9, 127.2, 123.5, 121.4, 118.8, 116.7, 115.8, 83.9, 33.8, 28.1, 25.6, 19.8, 19.3. **FT-IR**: ν (cm^{-1}) 2983, 1722, 1449, 1372, 1258, 1151, 1091, 1053, 857, 800, 766, 644. **HRMS** [ESI] calcd for $C_{17}H_{20}BrN_3NaO_2$ $[M+Na]^+$ 400.0631, found 400.0639.

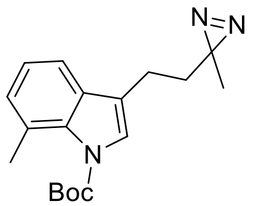

1ab, light brown oil. **^{1}H NMR** (400 MHz, $CDCl_3$) δ 7.30 (d, J = 6.0 Hz, 2H), 7.19-7.11 (m, 2H), 2.64 (s, 3H), 2.56-2.52 (m, 2H), 1.80-1.76 (m, 2H), 1.65 (s, 9H), 1.08 (s, 3H); **^{13}C NMR** (100 MHz, $CDCl_3$) δ 149.5, 135.1, 131.6, 127.8, 125.6, 124.7, 122.9, 119.2, 116.2, 83.0, 34.0, 28.1, 25.7, 22.1, 19.8, 19.4. **FT-IR**: ν (cm^{-1}) 2977, 2930, 1741, 1343, 1252, 1220, 1153, 1045, 1028, 789, 756. **HRMS** [ESI] calcd for $C_{18}H_{23}N_3NaO_2$ $[M+Na]^+$ 336.1682, found 336.1694.

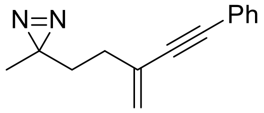

1ac, colorless oil. **^{1}H NMR** (400 MHz, $CDCl_3$) δ 7.44-7.41 (m, 2H), 7.33-7.30 (m, 3H), 5.45 (s, 1H), 5.34 (s, 1H), 2.14 (t, J = 8.0 Hz, 2H), 1.68-1.64 (m, 2H), 1.06 (s, 3H); **^{13}C NMR** (100 MHz, $CDCl_3$) δ 131.6, 130.2, 128.3, 123.0, 121.9, 89.8, 88.9, 33.3, 31.7, 25.5, 19.9. **FT-IR**: ν (cm^{-1}) 2922, 1609, 1489, 1443, 1069, 900, 754, 690. **HRMS** [EI] calcd for $C_{14}H_{14}N_2$ [M] 210.1157, found 210.1160.

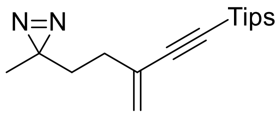

1ad, colorless oil. **^{1}H NMR** (400 MHz, $CDCl_3$) δ 5.39 (s, 1H), 5.29 (s, 1H), 2.07 (t, *J* = 8.0 Hz, 2H), 1.62-1.58 (m, 2H), 1.08 (s, 21H), 1.02 (s, 3H); **^{13}C NMR** (100 MHz, $CDCl_3$) δ 130.5, 122.3, 106.6, 91.0, 33.2, 31.7, 25.5, 19.9, 18.6, 11.2. **FT-IR**: ν (cm^{-1}) 2943, 2866, 2143, 1462, 1385, 996, 901, 881, 675, 659. **HRMS** [ESI] calcd for $C_{17}H_{30}KN_2Si$ $[M+K]^+$ 329.1810, found 329.1809.

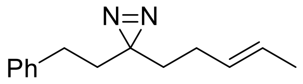

1ae, colorless oil. **^{1}H NMR** (400 MHz, $CDCl_3$) δ 7.29-7.24 (m, 2H), 7.20-7.12 (m, 3H), 5.46-5.38 (m, 1H), 5.34-5.28 (m, 1H), 2.40 (t, *J* = 8.2 Hz, 2H), 1.80-1.74 (m, 2H), 1.70-1.62 (m, 5H), 1.41 (t, *J* = 8.0 Hz, 2H); **^{13}C NMR** (100 MHz, $CDCl_3$) δ 140.8, 129.5, 128.4, 128.2, 126.1, 125.9, 35.0, 33.0, 29.9, 28.5, 26.8, 17.9. **FT-IR**: ν (cm^{-1}) 2919, 2855, 1582, 1496, 1453, 965, 746, 697. **HRMS** [EI] calcd for $C_{14}H_{18}N_2$ [M] 214.1470, found 214.1471.

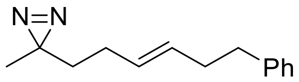

1af, colorless oil. **^{1}H NMR** (400 MHz, $CDCl_3$) δ 7.28-7.24 (m, 2H), 7.18-7.15 (m, 3H), 5.50-5.43 (m, 1H), 5.39-5.32 (m, 1H), 2.66 (t, *J* = 7.8 Hz, 2H), 2.33-2.27 (m, 2H), 1.89-1.83 (m, 2H), 1.39-1.35 (m, 2H), 0.98 (s, 3H); **^{13}C NMR** (100 MHz, $CDCl_3$) δ 141.9, 130.5, 129.2, 128.4, 128.2, 125.7, 35.9, 34.4, 34.3, 27.0, 25.6, 19.9. **FT-IR**: ν (cm^{-1}) 2924, 2853, 1602, 1496, 1452, 968, 745, 698. **HRMS** [ESI] calcd for $C_{14}H_{18}N_2Na$ $[M+Na]^+$ 237.1362, found 237.1355.

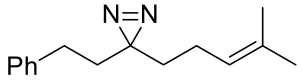

1ag, colorless oil. **^{1}H NMR** (400 MHz, $CDCl_3$) δ 7.30-7.26 (m, 2H), 7.21-7.13 (m, 3H), 5.01-4.97 (m, 1H), 2.42-2.38 (m, 2H), 1.79-1.75 (m, 2H), 1.73-1.67 (m, 5H), 1.57 (s, 3H), 1.42-1.38 (m, 2H); **^{13}C NMR** (100 MHz, $CDCl_3$) δ 140.8, 132.6, 128.5, 128.2, 126.1, 122.7, 35.1, 33.1, 30.0, 28.7, 25.7, 22.4, 17.6. **FT-IR**: ν (cm^{-1}) 2916, 2855, 1584, 1454, 747, 697. **HRMS** [EI] calcd for $C_{15}H_{20}N_2$ [M] 228.1626, found 228.1621.

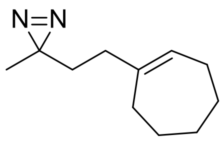

1ah, colorless oil. **^{1}H NMR** (400 MHz, $CDCl_3$) δ 5.54 (t, *J* = 6.4 Hz, 1H), 2.08-2.01 (m, 4H), 1.84 (t, *J* = 8.0 Hz, 2H), 1.74-1.68 (m, 2H), 1.48-1.39 (m, 6H), 1.00 (s, 3H); **^{13}C NMR** (100 MHz, $CDCl_3$) δ 143.1, 126.6, 34.2, 33.1, 32.7, 32.6, 28.2, 27.2, 26.7, 25.8, 19.8. **FT-IR**: ν (cm^{-1}) 2919, 2849, 1590, 1447, 1384, 1220, 965, 845. **HRMS** [EI] calcd for $C_{11}H_{18}N_2$ [M] 178.1470, found 178.1472.

7.2. Products

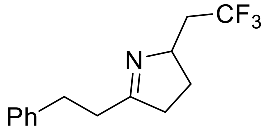

2a, colorless oil. **^{1}H NMR** (400 MHz, $CDCl_3$) δ 7.31-7.27 (m, 2H), 7.21-7.18 (m, 3H), 4.20-4.17 (m, 1H), 2.95-2.91 (m, 2H), 2.77-2.64 (m, 3H), 2.61-2.43 (m, 2H), 2.24-2.16 (m, 1H), 2.10-2.02 (m, 1H), 1.61-1.52 (m, 1H); **^{13}C NMR** (100 MHz, $CDCl_3$) δ 178.6, 141.0, 128.4, 128.2, 126.4 (q, J_{C-F} = 275.2 Hz), 126.1, 66.4 (q, J_{C-F} = 2.7 Hz), 40.1 (q, J_{C-F} = 26.8 Hz), 38.0, 35.2, 32.6, 29.2; **^{19}F NMR** (376 MHz, $CDCl_3$) δ −64.0

(s). **FT-IR**: ν (cm^{-1}) 2945, 2360, 2342, 1644, 1377, 1249, 1130, 1088, 1020, 840, 750, 699, 648. **HRMS** [ESI] calcd for $C_{14}H_{17}F_3N$ [M+H]$^+$ 256.1308, found 256.1318.

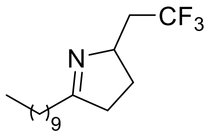

2b, colorless oil. **^{1}H NMR** (400 MHz, $CDCl_3$) δ 4.19-4.12 (m, 1H), 2.79-2.68 (m, 1H), 2.62-2.44 (m, 2H), 2.35-2.30 (m, 2H), 2.24-2.16 (m, 1H), 2.14-2.00 (m, 1H), 1.61-1.52 (m, 3H), 1.29-1.25 (m, 14H), 0.87 (t, *J* = 7.0 Hz, 3H); **^{13}C NMR** (100 MHz, $CDCl_3$) δ 179.6, 126.4 (q, J_{C-F} = 275.4 Hz), 66.3 (q, J_{C-F} = 2.5 Hz), 40.1 (q, J_{C-F} = 26.9 Hz), 37.5, 33.8, 31.9, 29.5, 29.5, 29.4, 29.3, 29.3, 29.2, 26.4, 22.7, 14.1; **^{19}F NMR** (376 MHz, $CDCl_3$) δ −64.0 (s). **FT-IR**: ν (cm^{-1}) 2925, 2855, 2360, 2342, 1644, 1377, 1249, 1136, 1090, 841, 649. **HRMS** [ESI] calcd for $C_{16}H_{29}F_3N$ [M+H]$^+$ 292.2247, found 292.2235.

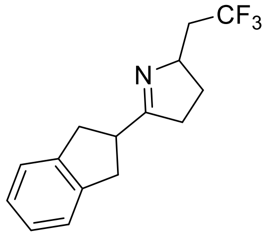

2c, colorless oil. **^{1}H NMR** (400 MHz, $CDCl_3$) δ 7.23-7.20 (m, 2H), 7.18-7.15 (m, 2H), 4.23-4.16 (m, 1H), 3.48-3.40 (m, 1H), 3.24-3.17 (m, 2H), 3.11-3.04 (m, 2H), 2.84-2.70 (m, 1H), 2.66-2.47 (m, 2H), 2.28-2.20 (m, 1H), 2.15-2.03 (m, 1H), 1.65-1.56 (m, 1H); **^{13}C NMR** (100 MHz, $CDCl_3$) δ 180.9, 142.1, 142.1, 126.5, 126.4 (q, J_{C-F} = 275.3 Hz), 124.4, 66.2 (q, J_{C-F} = 2.6 Hz), 43.3, 40.1 (q, J_{C-F} = 26.9 Hz), 36.8, 36.8, 35.9, 29.3; **^{19}F NMR** (376 MHz, $CDCl_3$) δ −64.0 (s). **FT-IR**: ν (cm^{-1}) 2946, 2361, 2342, 1637, 1377, 1249, 1136, 1089, 743, 648. **HRMS** [ESI] calcd for $C_{15}H_{17}F_3N$ [M+H]$^+$ 268.1308, found 268.1295.

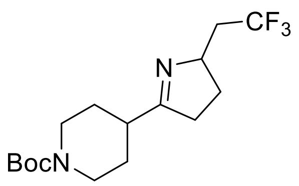

2d, colorless oil. **^{1}H NMR** (400 MHz, $CDCl_3$) δ 4.19-4.13 (m, 3H), 2.78-2.56 (m, 4H), 2.52-2.43 (m, 2H), 2.24-2.16 (m, 1H), 2.11-2.02 (m, 1H), 1.81-1.77 (m, 2H), 1.60-1.47 (m, 3H), 1.44 (s, 9H); **^{13}C NMR** (100 MHz, $CDCl_3$) δ 181.0, 154.7, 126.3 (q, J_{C-F} = 275.4 Hz), 79.5, 66.2 (q, J_{C-F} = 2.6 Hz), 43.6, 40.5, 40.0 (q, J_{C-F} = 27.0 Hz), 35.4, 29.3, 29.2, 28.9, 28.4; **^{19}F NMR** (376 MHz, $CDCl_3$) δ −64.0 (s). **FT-IR**: ν (cm^{-1}) 2976, 2934, 2361, 2342, 1688, 1423, 1366, 1250, 1165, 1138, 1090, 1012. **HRMS** [ESI] calcd for $C_{16}H_{25}F_3N_2O_2$ [M+Na]$^+$ 357.1760, found 357.1760.

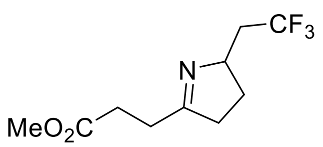

2e, colorless oil. **^{1}H NMR** (400 MHz, $CDCl_3$) δ 4.21-4.14 (m, 1H), 3.68 (s, 3H), 2.75-2.48 (m, 7H), 2.25-2.17 (m, 1H), 2.11-2.02 (m, 1H), 1.65-1.56 (m, 1H); **^{13}C NMR** (100 MHz, $CDCl_3$) δ 177.1, 173.3, 126.3 (q, J_{C-F} = 275.2 Hz), 66.4 (q, J_{C-F} = 2.5 Hz), 51.6, 40.0 (q, J_{C-F} = 26.9 Hz), 38.1, 30.3, 29.1, 28.3; **^{19}F NMR** (376 MHz, $CDCl_3$) δ −64.1 (s). **FT-IR**: ν (cm^{-1}) 2955, 1736, 1249, 1130, 1097, 1019, 840, 644. **HRMS** [ESI] calcd for $C_{10}H_{15}F_3NO_2$ [M+H]$^+$ 238.1049, found 238.1044.

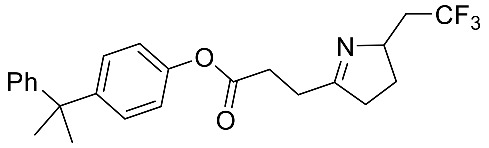

2f, colorless oil. **^{1}H NMR** (400 MHz, $CDCl_3$) δ 7.28-7.15 (m, 7H), 6.96 (d, *J* = 8.4 Hz, 2H), 4.21-4.18 (m, 1H), 2.98-2.85 (m, 2H), 2.77-2.50 (m, 5H), 2.27-2.18 (m, 1H), 2.15-2.01 (m, 1H), 1.67 (s, 6H), 1.64-1.56 (m, 1H); **^{13}C NMR** (100 MHz, $CDCl_3$) δ 177.0, 171.6,

150.3, 148.5, 148.2, 128.0, 127.8, 126.7, 126.3 (q, J_{C-F} = 275.3 Hz), 125.7, 120.7, 66.4 (q, J_{C-F} = 2.6 Hz), 42.7, 40.1 (q, J_{C-F} = 26.9 Hz), 38.3, 30.8, 30.7, 29.2, 28.2; **^{19}F NMR** (376 MHz, $CDCl_3$) δ −64.0 (s). **FT-IR**: ν (cm^{-1}) 2970, 2360, 2342, 1756, 1505, 1250, 1205, 1170, 1133, 1017, 840, 764, 700. **HRMS** [ESI] calcd for $C_{24}H_{26}F_3NO_2$ $[M+Na]^+$ 440.1808, found 440.1800.

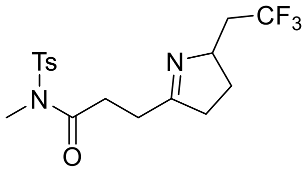

2g, colorless oil. **^{1}H NMR** (400 MHz, $CDCl_3$) δ 7.83 (d, J = 8.2 Hz, 2H), 7.33 (d, J = 8.2 Hz, 2H), 4.08-4.01 (m, 1H), 3.31 (s, 3H), 3.13-2.95 (m, 2H), 2.65-2.41 (m, 8H), 2.19-2.10 (m, 1H), 1.99-1.90 (m, 1H), 1.57-1.48 (m, 1H); **^{13}C NMR** (100 MHz, $CDCl_3$) δ 177.1, 172.5, 144.7, 136.2, 129.7, 127.6, 126.3 (q, J_{C-F} = 275.3 Hz), 66.1 (q, J_{C-F} = 2.6 Hz), 40.0 (q, J_{C-F} = 26.8 Hz), 38.2, 33.0, 32.7, 29.1, 27.9, 21.5; **^{19}F NMR** (376 MHz, $CDCl_3$) δ −64.0 (s). **FT-IR**: ν (cm^{-1}) 2952, 2361, 2342, 1699, 1354, 1249, 1160, 1131, 1085, 814, 711, 665. **HRMS** [ESI] calcd for $C_{17}H_{21}F_3N_2O_3S$ $[M+Na]^+$ 413.1117, found 413.1114.

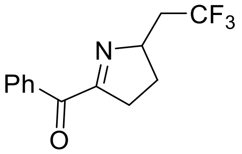

2h′, colorless oil (*Note*: The isolated imine **2h** was unstable, and underwent spontaneous oxidation under air during work-up to furnish **2h′**). **^{1}H NMR** (400 MHz, $CDCl_3$) δ 8.21-8.19 (m, 2H), 7.62-7.58 (m, 1H), 7.49-7.45 (m, 2H), 4.60-4.52 (m, 1H), 3.15-3.06 (m, 1H), 3.01-2.83 (m, 2H), 2.40-2.27 (m, 2H), 1.78-1.68 (m, 1H); **^{13}C NMR** (100 MHz, $CDCl_3$) δ 190.4, 174.2, 135.1, 133.7, 130.5, 128.4, 126.1 (q, J_{C-F} = 275.2 Hz), 68.9 (q, J_{C-F} = 2.6 Hz), 39.7 (q, J_{C-F} = 27.6 Hz), 36.3, 28.3; **^{19}F NMR** (376 MHz, $CDCl_3$) δ −64.0 (s). **FT-IR**: ν (cm^{-1}) 2953, 1659, 1249, 1144, 1130, 1089, 917, 702, 644. **HRMS** [ESI] calcd for $C_{13}H_{13}F_3NNaO$ $[M+H]^+$ 278.0763, found 278.0770.

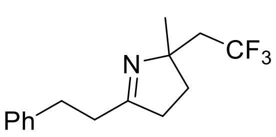

2i, colorless oil. **^{1}H NMR** (400 MHz, $CDCl_3$) δ 7.30-7.26 (m, 2H), 7.21-7.17 (m, 3H), 2.91 (t, J = 8.0 Hz, 2H), 2.65-2.61 (m, 2H), 2.58-2.43 (m, 3H), 2.30-2.18 (m, 1H), 2.01-1.93 (m, 1H), 1.78-1.72 (m, 1H), 1.26 (s, 3H); **^{13}C NMR** (100 MHz, $CDCl_3$) δ 175.9, 141.0, 128.4, 128.3, 126.3 (q, J_{C-F} = 276.3 Hz), 126.1, 72.4 (q, J_{C-F} = 1.9 Hz), 44.1 (q, J_{C-F} = 26.0 Hz), 37.8, 35.1, 34.1 (q, J_{C-F} = 1.4 Hz), 32.7, 26.8; **^{19}F NMR** (376 MHz, $CDCl_3$) δ −60.5 (s). **FT-IR**: ν (cm^{-1}) 2969, 2938, 1645, 1364, 1258, 1151, 1089, 1044, 748, 698, 652. **HRMS** [ESI] calcd for $C_{15}H_{19}F_3N$ $[M+H]^+$ 270.1464, found 270.1463.

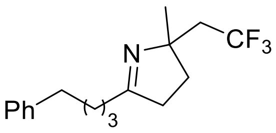

2j, colorless oil. **^{1}H NMR** (400 MHz, $CDCl_3$) δ 7.29-7.25 (m, 2H), 7.19-7.15 (m, 3H), 2.63 (t, J = 7.6 Hz, 2H), 2.58-2.44 (m, 3H), 2.35-2.21 (m, 3H), 2.02-1.94 (m, 1H), 1.79-1.72 (m, 1H), 1.70-1.57 (m, 4H), 1.27 (s, 3H); **^{13}C NMR** (100 MHz, $CDCl_3$) δ 176.6, 142.3, 128.3, 128.3, 126.3 (q, J_{C-F} = 276.3 Hz), 125.7, 72.2 (q, J_{C-F} = 1.7 Hz), 44.2 (q, J_{C-F} = 25.9 Hz), 37.3, 35.6, 34.0 (q, J_{C-F} = 1.6 Hz), 33.5, 31.1, 26.9 (q, J_{C-F} = 1.3 Hz), 26.1; **^{19}F NMR** (376 MHz, $CDCl_3$) δ −60.5 (s). **FT-IR**: ν (cm^{-1}) 2936, 2860, 1644, 1454, 1364, 1258, 1150, 1090, 839, 747, 698, 652. **HRMS** [ESI] calcd for

$C_{17}H_{23}F_3N$ [M+H]$^+$ 298.1777, found 298.1781.

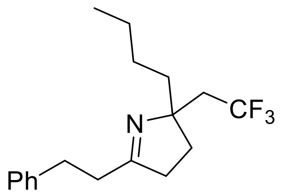

2k, colorless oil. **^{1}H NMR** (400 MHz, $CDCl_3$) δ 7.29-7.26 (m, 2H), 7.22-7.16 (m, 3H), 2.97-2.85 (m, 2H), 2.65 (t, *J* = 7.8 Hz, 2H), 2.56-2.42 (m, 3H), 2.35-2.22 (m, 1H), 1.96-1.89 (m, 1H), 1.82-1.75 (m, 1H), 1.64-1.49 (m, 2H), 1.32-1.23 (m, 2H), 1.21-1.10 (m, 2H), 0.88 (t, *J* = 7.2 Hz, 3H); **^{13}C NMR** (100 MHz, $CDCl_3$) δ 176.1, 141.0, 128.4, 128.2, 126.4 (q, $J_{C\text{-}F}$ = 276.6 Hz), 126.1, 75.5 (q, $J_{C\text{-}F}$ = 1.5 Hz), 42.9 (q, $J_{C\text{-}F}$ = 25.6 Hz), 40.3, 38.3, 35.0, 32.7, 31.3 (q, $J_{C\text{-}F}$ = 1.5 Hz), 25.7, 23.0, 14.0; **^{19}F NMR** (376 MHz, $CDCl_3$) δ −60.0 (s). **FT-IR**: ν (cm^{-1}) 2957, 2932, 2863, 2361, 2342, 1648, 1456, 1366, 1256, 1143, 1109, 748, 698, 654. **HRMS** [ESI] calcd for $C_{18}H_{25}F_3N$ [M+H]$^+$ 312.1934, found 312.1937.

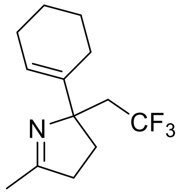

2l, colorless oil. **^{1}H NMR** (400 MHz, $CDCl_3$) δ 5.64-5.62 (m, 1H), 2.60-2.40 (m, 4H), 2.06-1.96 (m, 9H), 1.68-1.50 (m, 4H); **^{13}C NMR** (100 MHz, $CDCl_3$) δ 174.3, 139.7, 126.2 (q, $J_{C\text{-}F}$ = 276.9 Hz), 120.9, 78.8 (q, $J_{C\text{-}F}$ = 1.8 Hz), 41.8 (q, $J_{C\text{-}F}$ = 25.4 Hz), 38.8, 32.7 (q, $J_{C\text{-}F}$ = 1.4 Hz), 25.2, 25.1, 23.0, 22.3, 19.7; **^{19}F NMR** (376 MHz, $CDCl_3$) δ −60.6 (s). **FT-IR**: ν (cm^{-1}) 2929, 1650, 1433, 1363, 1258, 1130, 1111. **HRMS** [ESI] calcd for $C_{13}H_{19}F_3N$ [M+H]$^+$ 246.1464, found 246.1463.

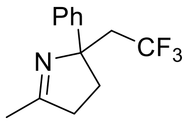

2m, colorless oil. **^{1}H NMR** (400 MHz, $CDCl_3$) δ 7.45-7.42 (m, 2H), 7.34-7.30 (m, 2H), 7.26-7.21 (m, 1H), 2.76-2.63 (m, 3H), 2.58-2.40 (m, 2H), 2.33-2.25 (m, 1H), 2.12 (s, 3H); **^{13}C NMR** (100 MHz, $CDCl_3$) δ 175.2, 146.3, 128.3, 126.9, 125.8 (q, $J_{C\text{-}F}$ = 277.0 Hz), 125.6, 77.8 (q, $J_{C\text{-}F}$ = 1.8 Hz), 45.6 (q, $J_{C\text{-}F}$ = 25.6 Hz), 39.2, 34.9 (q, $J_{C\text{-}F}$ = 1.6 Hz), 19.8; **^{19}F NMR** (376 MHz, $CDCl_3$) δ −60.4 (s). **FT-IR**: ν (cm^{-1}) 2952, 2361, 2342, 1652, 1432, 1365, 1257, 1120, 1077, 1030, 762, 702. **HRMS** [ESI] calcd for $C_{13}H_{15}F_3N$ [M+H]$^+$ 242.1151, found 242.1149.

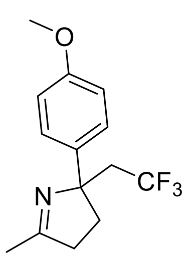

2n, colorless oil. **^{1}H NMR** (400 MHz, $CDCl_3$) δ 7.36 (d, *J* = 8.8 Hz, 2H), 6.86 (d, *J* = 8.8 Hz, 2H), 3.79 (s, 3H), 2.73-2.60 (m, 3H), 2.57-2.48 (m, 1H), 2.43-2.36 (m, 1H), 2.32-2.24 (m, 1H), 2.10 (s, 3H); **^{13}C NMR** (100 MHz, $CDCl_3$) δ 174.8, 158.3, 138.3, 126.7, 125.8 (q, $J_{C\text{-}F}$ = 277.0 Hz), 113.5, 77.3 55.2, 45.7 (q, $J_{C\text{-}F}$ = 25.4 Hz), 39.2, 34.9 (q, $J_{C\text{-}F}$ = 1.6 Hz), 19.8; **^{19}F NMR** (376 MHz, $CDCl_3$) δ −60.4 (s). **FT-IR**: ν (cm^{-1}) 2956, 2361, 2342, 1652, 1510, 1365, 1244, 1179, 1120, 1033, 830, 653. **HRMS** [ESI] calcd for $C_{14}H_{17}F_3NO$ [M+H]$^+$ 272.1257, found 272.1254.

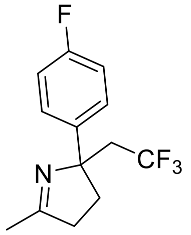

2o, colorless oil. **^{1}H NMR** (400 MHz, $CDCl_3$) δ 7.44-7.39 (m, 2H), 7.04-6.98 (m, 2H), 2.72-2.50 (m, 4H), 2.46-2.39 (m, 1H), 2.29-2.22 (m, 1H), 2.12 (s, 3H); **^{13}C NMR** (100 MHz, $CDCl_3$) δ 175.3, 161.7 (d, $J_{C\text{-}F}$ = 244.0 Hz), 141.9 (d, $J_{C\text{-}F}$ = 3.0 Hz), 127.4 (d, $J_{C\text{-}F}$ = 7.9 Hz), 125.7 (q, $J_{C\text{-}F}$ = 277.0 Hz), 115.0 (d, $J_{C\text{-}F}$ = 7.9 Hz), 77.3, 45.7 (q, $J_{C\text{-}F}$ = 25.7 Hz), 39.2, 35.1, 19.8; **^{19}F NMR** (376 MHz, $CDCl_3$) δ −60.4 (s, 3F), −116.1 (s, 1F). **FT-IR**: ν (cm^{-1}) 2950, 2361, 1651, 1508, 1366, 1258, 1224, 1120, 1097, 834. **HRMS** [ESI] calcd for $C_{13}H_{14}F_4N$ $[M+H]^+$ 260.1057, found 260.1061.

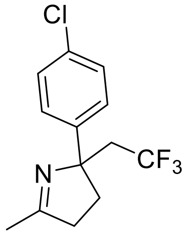

2p, colorless oil. **^{1}H NMR** (400 MHz, $CDCl_3$) δ 7.38 (d, *J* = 8.6 Hz, 2H), 7.29 (d, *J* = 8.6 Hz, 2H), 2.71-2.49 (m, 4H), 2.45-2.38 (m, 1H), 2.27-2.19 (m, 1H), 2.11 (s, 3H); **^{13}C NMR** (100 MHz, $CDCl_3$) δ 175.5, 144.7, 132.7, 128.4, 127.1, 125.6 (q, $J_{C\text{-}F}$ = 277.0 Hz), 77.4 (q, $J_{C\text{-}F}$ = 1.4 Hz), 45.5 (q, $J_{C\text{-}F}$ = 25.8 Hz), 39.2, 35.0 (q, $J_{C\text{-}F}$ = 1.2 Hz), 19.8; **^{19}F NMR** (376 MHz, $CDCl_3$) δ −60.3 (s). **FT-IR**: ν (cm^{-1}) 2954, 2361, 2342, 1650, 1492, 1257, 1120, 1103, 1014, 829. **HRMS** [ESI] calcd for $C_{13}H_{14}ClF_3N$ $[M+H]^+$ 276.0761, found 276.0766.

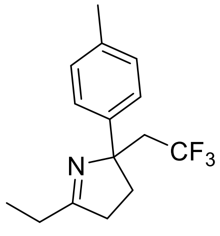

2q, colorless oil, **^{1}H NMR** (400 MHz, $CDCl_3$) δ 7.32 (d, *J* = 8.0 Hz, 2H), 7.13 (d, *J* = 8.0 Hz, 2H), 2.73-2.62 (m, 3H), 2.55-2.37 (m, 4H), 2.32 (s, 3H), 2.28-2.20 (m, 1H), 1.19 (t, *J* = 7.6 Hz, 3H); **^{13}C NMR** (100 MHz, $CDCl_3$) δ 179.2, 143.8, 136.4, 128.9, 125.9 (q, $J_{C\text{-}F}$ = 277.1 Hz), 125.4, 45.6 (q, $J_{C\text{-}F}$ = 25.5 Hz), 37.1, 34.6 (q, $J_{C\text{-}F}$ = 1.4 Hz), 27.0, 20.9, 10.8; **^{19}F NMR** (376 MHz, $CDCl_3$) δ −60.2 (s). **FT-IR**: ν (cm^{-1}) 2975, 2361, 2342, 1650, 1364, 1257, 1118, 812, 650. **HRMS** [ESI] calcd for $C_{15}H_{19}F_3N$ $[M+H]^+$ 270.1464, found 270.1460.

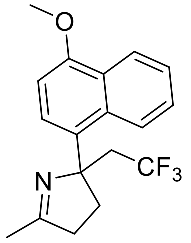

2r, white solid, mp. 96-97 ^{o}C. **^{1}H NMR** (400 MHz, $CDCl_3$) δ 8.38 (dd, *J* = 8.2, 1.0 Hz, 1H), 8.06 (d, *J* = 8.4 Hz, 1H), 7.77 (d, *J* = 8.0 Hz, 1H), 7.58-7.46 (m, 2H), 6.73 (d, *J* = 8.4 Hz, 1H), 3.99 (s, 3H), 3.22-3.10 (m, 1H), 2.95-2.74 (m, 3H), 2.64-2.56 (m, 1H), 2.43-2.35 (m, 1H), 2.20 (s, 3H); **^{13}C NMR** (100 MHz, $CDCl_3$) δ 174.2, 155.0, 135.1, 130.4, 126.8, 126.3 (q, $J_{C\text{-}F}$ = 277.4 Hz), 126.3, 124.6, 124.5, 123.9, 123.4, 102.7, 78.0 (q, $J_{C\text{-}F}$ = 1.7 Hz), 55.4, 43.9 (q, $J_{C\text{-}F}$ = 25.2 Hz), 40.1, 34.4 (q, $J_{C\text{-}F}$ = 1.4 Hz), 19.7; **^{19}F NMR** (376 MHz, $CDCl_3$) δ −60.5 (s). **FT-IR**: ν (cm^{-1}) 2988, 2901, 2361, 2342, 1521, 1066, 669. **HRMS** [ESI] calcd for $C_{18}H_{19}F_3NO$ $[M+H]^+$ 322.1413, found 322.1414.

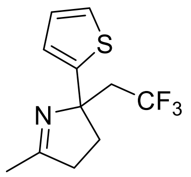

2s, colorless oil. **^{1}H NMR** (400 MHz, $CDCl_3$) δ 7.21-7.19 (m, 1H), 6.96-6.93 (m, 2H), 2.91-2.65 (m, 4H), 2.44-2.40 (m, 2H), 2.09 (s, 3H); **^{13}C NMR** (100 MHz, $CDCl_3$) δ 175.7, 149.7, 126.6, 125.5 (q, $J_{C\text{-}F}$ = 276.8 Hz), 124.3, 122.8, 76.2 (q, $J_{C\text{-}F}$ = 1.8 Hz), 46.3 (q, $J_{C\text{-}F}$ = 25.8 Hz), 39.7, 35.5 (q, $J_{C\text{-}F}$ = 1.7 Hz), 19.5; **^{19}F NMR** (376 MHz, $CDCl_3$) δ −60.5 (s). **FT-IR**: ν (cm^{-1}) 2924, 2342, 1619, 1440, 1385, 1227, 884, 852, 829, 695.

HRMS [ESI] calcd for $C_{11}H_{13}F_3NS$ $[M+H]^+$ 248.0715, found 248.0710.

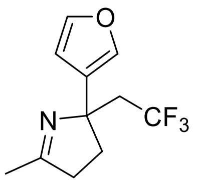

2t, colorless oil. **^{1}H NMR** (400 MHz, $CDCl_3$) δ 7.37 (t, J = 1.4 Hz, 1H), 7.30 (s, 1H), 6.39 (s, 1H), 2.83-2.71 (m, 1H), 2.68-2.55 (m, 3H), 2.27-2.19 (m, 2H), 2.06 (s, 3H); **^{13}C NMR** (100 MHz, $CDCl_3$) δ 175.0, 143.3, 138.2, 130.1, 125.8 (q, $J_{C\text{-}F}$ = 276.6 Hz), 109.0, 73.1 (q, $J_{C\text{-}F}$ = 1.8Hz), 44.9 (q, $J_{C\text{-}F}$ = 25.8 Hz), 39.3, 34.3, 19.6; **^{19}F NMR** (376 MHz, $CDCl_3$) δ −60.4 (s). **FT-IR**: ν (cm^{-1}) 2954, 1649, 1365, 1258, 1126, 1070, 874, 792. **HRMS** [ESI] calcd for $C_{11}H_{13}F_3NO$ $[M+H]^+$ 232.0944, found 232.0945.

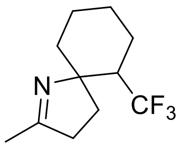

2u, light yellow oil, **^{1}H NMR** (400 MHz, $CDCl_3$) δ 2.59-2.45 (m, 3H), 2.16-2.09 (m, 1H), 2.01-1.97 (m, 4H), 1.84-1.59 (m, 4H), 1.41-1.29 (m, 4H); **^{13}C NMR** (100 MHz, $CDCl_3$) δ 172.4, 127.7 (q, $J_{C\text{-}F}$ = 280.6 Hz), 48.5 (q, $J_{C\text{-}F}$ = 22.9 Hz), 40.3, 38.8, 27.6 (q, $J_{C\text{-}F}$ = 1.8 Hz), 24.6, 23.6 (q, $J_{C\text{-}F}$ = 2.8 Hz), 22.6, 19.5; **^{19}F NMR** (376 MHz, $CDCl_3$) δ −65.7 (s). **FT-IR**: ν (cm^{-1}) 2934, 2862, 1452, 1378, 1182, 1129, 1089, 1073, 732. **HRMS** [ESI] calcd for $C_{11}H_{17}F_3N$ $[M+H]^+$ 220.1308, found 220.1304.

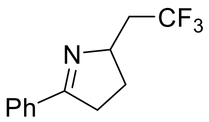

2v, colorless oil. **^{1}H NMR** (400 MHz, $CDCl_3$) δ 7.84-7.82 (m, 2H), 7.47-7.36 (m, 3H), 4.45-4.38 (m, 1H), 3.15-3.07 (m, 1H), 2.98-2.86 (m, 2H), 2.43-2.35 (m, 1H), 2.27-2.12 (m, 1H), 1.80-1.70 (m, 1H); **^{13}C NMR** (100 MHz, $CDCl_3$) δ 173.7, 134.0, 130.8, 128.5, 127.7, 126.4 (q, $J_{C\text{-}F}$ = 275.2 Hz), 67.0 (q, $J_{C\text{-}F}$ = 2.7 Hz), 40.2 (q, $J_{C\text{-}F}$ = 26.9 Hz), 35.4, 29.3; **^{19}F NMR** (376 MHz, $CDCl_3$) δ −64.0 (s). **FT-IR**: ν (cm^{-1}) 2947, 2360, 1616, 1379, 1343, 1249, 1135, 1090, 1012, 761, 692, 647. **HRMS** [ESI] calcd for $C_{12}H_{13}F_3N$ $[M+H]^+$ 228.0995, found 228.0986.

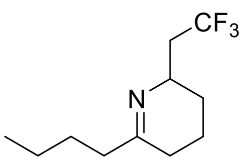

2w, colorless oil. **^{1}H NMR** (400 MHz, $CDCl_3$) 3.70-3.58 (m, 1H), 2.75-2.61 (m, 1H), 2.20-2.09 (m, 4H), 1.96-1.91 (m, 1H), 1.81-1.73 (m, 1H), 1.65-1.59 (m, 1H), 1.54-1.46 (m, 2H), 1.37-1.28 (m, 4H), 0.91 (t, J = 7.2 Hz, 3H); **^{13}C NMR** (100 MHz, $CDCl_3$) δ 172.3, 126.6 (q, $J_{C\text{-}F}$ = 275.5 Hz), 52.6 (q, $J_{C\text{-}F}$ = 2.9 Hz), 41.6 (q, $J_{C\text{-}F}$ = 26.6 Hz), 40.7, 28.8, 28.5, 27.1, 22.5, 18.4, 13.9; **^{19}F NMR** (376 MHz, $CDCl_3$) δ −63.1 (s). **FT-IR**: ν (cm^{-1}) 2958, 2933, 1660, 1374, 1249, 1142, 1037. **HRMS** [ESI] calcd for $C_{11}H_{19}F_3N$ $[M+H]^+$ 222.1464, found 222.1462.

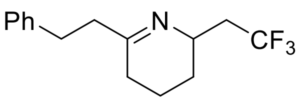

2x, colorless oil. **^{1}H NMR** (400 MHz, $CDCl_3$) δ 7.29-7.25 (m, 2H), 7.20-7.16 (m, 3H), 3.70-3.60 (m, 1H), 2.86 (t, J = 8.0 Hz, 2H), 2.69-2.55 (m, 1H), 2.49-2.45 (m, 2H), 2.15-1.98 (m, 3H), 1.95-1.88 (m, 1H), 1.79-1.70 (m, 1H), 1.63-1.52 (m, 1H), 1.26-1.15 (m, 1H); **^{13}C NMR** (100 MHz, $CDCl_3$) δ 171.0, 141.7, 128.4, 128.3, 126.6 (q, $J_{C\text{-}F}$ = 275.5 Hz), 125.9, 52.6 (q, $J_{C\text{-}F}$ = 2.8 Hz), 42.1, 41.5 (q, $J_{C\text{-}F}$ = 26.6 Hz), 32.5, 29.2, 27.1, 18.4; **^{19}F NMR** (376 MHz, $CDCl_3$) δ −63.1 (s). **FT-IR**: ν (cm^{-1}) 2932, 1659, 1374, 1250, 1137, 1039,

749, 699. **HRMS** [ESI] calcd for $C_{15}H_{19}F_3N$ $[M+H]^+$ 270.1464, found 270.1469.

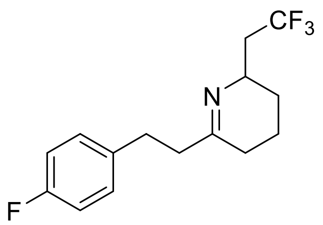

2y, colorless oil. **^{1}H NMR** (400 MHz, $CDCl_3$) δ 7.16-7.13 (m, 2H), 6.97-6.93 (m, 2H), 3.70-3.60 (m, 1H), 2.84 (t, J = 8.0 Hz, 2H), 2.69-2.55 (m, 1H), 2.47-2.43 (m, 2H), 2.16-2.02 (m, 3H), 1.94-1.90 (m, 1H), 1.79-1.72 (m, 1H), 1.64-1.53 (m, 1H), 1.29-1.19 (m, 1H); **^{13}C NMR** (100 MHz, $CDCl_3$) δ 178.8, 161.3 (d, $J_{C\text{-}F}$ = 242.1 Hz), 137.3 (d, $J_{C\text{-}F}$ = 3.1 Hz), 129.7 (d, $J_{C\text{-}F}$ = 7.8 Hz), 126.6 (q, $J_{C\text{-}F}$ = 275.5 Hz), 115.0 (d, $J_{C\text{-}F}$ = 20.9 Hz), 52.7 (q, $J_{C\text{-}F}$ = 2.8 Hz), 42.0, 41.5 (q, $J_{C\text{-}F}$ = 26.6 Hz), 31.5, 29.3, 27.1, 18.4; **^{19}F NMR** (376 MHz, $CDCl_3$) δ −63.1 (s), −117.6 (s). **FT-IR**: ν (cm^{-1}) 2930, 1742, 1510, 1221, 1139, 1016, 824. **HRMS** [ESI] calcd for $C_{15}H_{18}F_4N$ $[M+H]^+$ 288.1370, found 288.1372.

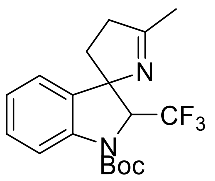

2z, colorless oil, **^{1}H NMR** (400 MHz, $CDCl_3$) δ 7.79-7.67 (m, 1H), 7.27-7.23 (m, 1H), 7.06-6.97 (m, 2H), 4.75 (q, J = 8.0 Hz, 1H), 2.97-2.88 (m, 1H), 2.81-2.73 (m, 1H), 2.61-2.53 (m, 1H), 2.49-2.42 (m, 1H), 2.06 (s, 3H), 1.56 (s, 9H); **^{13}C NMR** (100 MHz, $CDCl_3$) δ 174.2, 151.7, 141.4, 135.3, 129.3, 124.6 (q, $J_{C\text{-}F}$ = 283.2 Hz), 123.5, 121.8, 116.2, 82.4, 82.2, 68.7 (q, $J_{C\text{-}F}$ = 28.0 Hz), 40.4, 28.2 (q, $J_{C\text{-}F}$ = 2.9 Hz), 28.2, 19.6; **^{19}F NMR** (376 MHz, $CDCl_3$) δ −70.2 (s). **FT-IR**: ν (cm^{-1}) 2927, 1709, 1483, 1253, 1164, 1126, 868, 752. **HRMS** [ESI] calcd for $C_{18}H_{22}F_3N_2O_2$ $[M+H]^+$ 355.1628, found 355.1641.

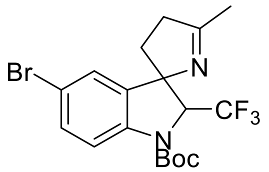

2aa, white solid, mp. 104-105 °C. **^{1}H NMR** (400 MHz, $CDCl_3$) δ 7.62 (d, J = 8.8 Hz, 1H), 7.36 (dd, J = 8.4, 2.0 Hz, 1H), 7.13 (d, J = 2.4 Hz, 1H), 4.72 (q, J = 8.0 Hz, 1H), 2.96-2.88 (m, 1H), 2.82-2.74 (m, 1H), 2.60-2.53 (m, 1H), 2.45-2.38 (m, 1H), 2.08 (s, 3H), 1.55 (s, 9H); **^{13}C NMR** (100 MHz, $CDCl_3$) 175.0, 151.4, 140.7, 137.4, 132.1, 125.1, 124.4 (q, $J_{C\text{-}F}$ = 288.3 Hz), 117.7, 115.8, 82.6, 82.2, 68.9 (q, $J_{C\text{-}F}$ = 29.2 Hz), 40.4, 28.1, 28.1 (q, $J_{C\text{-}F}$ = 3.5 Hz), 19.6; **^{19}F NMR** (376 MHz, $CDCl_3$) δ −70.2 (s). **FT-IR**: ν (cm^{-1}) 2951, 1361, 1248, 1161, 1136, 1121, 891, 840, 755, 700, 688, 661. **HRMS** [ESI] calcd for $C_{18}H_{21}BrF_3N_2O_2$ $[M+H]^+$ 433.0733, found 433.0744.

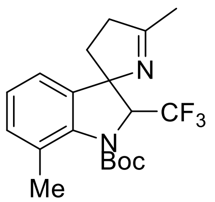

2ab, white solid, mp. 110-111 °C. **^{1}H NMR** (400 MHz, $CDCl_3$) δ 7.09 (d, J = 8.0 Hz, 1H), 7.01 (t, J = 7.6 Hz, 1H), 6.89 (d, J = 7.6 Hz, 1H), 4.77 (q, J = 8.4 Hz, 1H), 2.95-2.86 (m, 1H), 2.78-2.70 (m, 1H), 2.52-2.44 (m, 1H), 2.39-2.34 (m, 4H), 2.04 (s, 3H), 1.54 (s, 9H); **^{13}C NMR** (100 MHz, $CDCl_3$) 173.9, 153.7, 141.2, 137.7, 131.3, 128.9, 125.3, 124.7 (q, $J_{C\text{-}F}$ = 287.1 Hz), 119.0, 83.3, 82.0, 71.8 (q, $J_{C\text{-}F}$ = 29.1 Hz), 40.4, 28.2, 27.8 (q, $J_{C\text{-}F}$ = 2.6 Hz), 19.7, 19.6; **^{19}F NMR** (376 MHz, $CDCl_3$) δ −70.6 (s). **FT-IR**: ν (cm^{-1}) 2926, 1712, 1464, 1370, 1250, 1156, 1126, 878. **HRMS** [ESI] calcd for $C_{19}H_{23}F_3N_2NaO_2$ $[M+Na]^+$ 391.1604, found 391.1613.

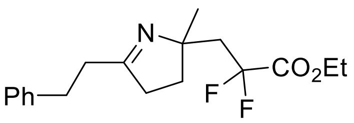

3a, colorless oil. **^{1}H NMR** (400 MHz, $CDCl_3$) δ 7.30-7.26 (m, 2H), 7.21-7.17 (m, 3H), 4.30 (q, *J* = 7.2 Hz, 2H), 2.95-2.82 (m, 2H), 2.60-2.51 (m, 4H), 2.46-2.35 (m, 2H), 1.99-1.92 (m, 1H), 1.75-1.68 (m, 1H), 1.34 (t, *J* = 7.2 Hz, 3H), 1.25 (s, 3H); **^{13}C NMR** (100 MHz, $CDCl_3$) δ 176.1, 164.3 (t, $J_{C\text{-}F}$ = 32.3 Hz), 141.2, 128.4, 128.2, 126.0, 115.9 (t, $J_{C\text{-}F}$ = 248.8 Hz), 77.7 (t, $J_{C\text{-}F}$ = 2.8 Hz), 62.6, 44.9 (t, $J_{C\text{-}F}$ = 21.4 Hz), 37.6, 35.2, 34.9, 32.5, 27.6 (d, $J_{C\text{-}F}$ = 2.0 Hz), 13.9; **^{19}F NMR** (376 MHz, $CDCl_3$) δ −100.8 (d, *J* = 263.2 Hz, 1F), −102.2 (d, *J* = 262.8 Hz, 1F). **FT-IR**: ν (cm^{-1}) 2966, 2933, 1766, 1644, 1179, 1148, 1058, 750, 700. **HRMS** [ESI] calcd for $C_{18}H_{24}F_2NO_2$ $[M+H]^+$ 324.1770, found 324.1766.

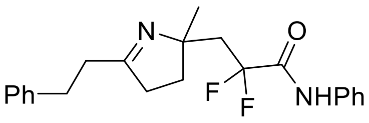

3b, colorless oil. **^{1}H NMR** (400 MHz, $CDCl_3$) δ 8.52 (s, 1H), 7.60 (d, *J* = 7.6 Hz, 2H), 7.34 (t, *J* = 7.8 Hz, 2H), 7.26-7.14 (m, 4H), 7.04 (d, *J* = 7.2 Hz, 2H), 2.87-2.74 (m, 2H), 2.63-2.38 (m, 6H), 2.00-1.93 (m, 1H), 1.77-1.70 (m, 1H), 1.31 (s, 3H); **^{13}C NMR** (100 MHz, $CDCl_3$) δ 176.7, 162.4 (t, $J_{C\text{-}F}$ = 28.2 Hz), 141.1, 136.6, 129.2, 128.4, 128.2, 126.0, 125.2, 120.0, 117.8 (t, $J_{C\text{-}F}$ = 252.4 Hz), 72.5 (t, $J_{C\text{-}F}$ = 2.6 Hz), 43.9 (t, $J_{C\text{-}F}$ = 21.6 Hz), 37.5, 36.2, 35.2, 32.5, 27.8 (d, $J_{C\text{-}F}$ = 2.3 Hz); **^{19}F NMR** (376 MHz, $CDCl_3$) δ −98.3 (d, *J* = 259.1 Hz), −102.3 (d, *J* = 259.1 Hz). **FT-IR**: ν (cm^{-1}) 2929, 2361, 1698, 1602, 1542, 1498, 1447, 1238, 1180, 1149, 1039, 751, 692. **HRMS** [ESI] calcd for $C_{22}H_{24}F_2N_2NaO$ $[M+Na]^+$ 393.1749, found 393.1749.

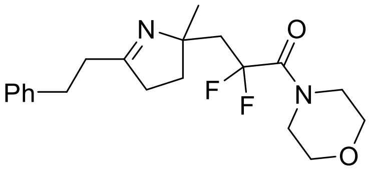

3c, colorless oil. **^{1}H NMR** (400 MHz, $CDCl_3$) δ 7.30-7.26 (m, 2H), 7.21-7.17 (m, 3H), 3.72-3.63 (m, 8H), 2.92-2.88 (m, 2H), 2.63-2.33 (m, 6H), 2.04-1.96 (m, 1H), 1.77-1.70 (m, 1H), 1.28 (s, 3H); **^{13}C NMR** (100 MHz, $CDCl_3$) δ 175.5, 162.5 (t, $J_{C\text{-}F}$ = 29.0 Hz), 141.1, 128.4, 128.2, 126.0, 119.0 (t, $J_{C\text{-}F}$ = 252.5 Hz), 73.2, 66.7, 46.7 (t, $J_{C\text{-}F}$ = 5.8 Hz), 44.2 (t, $J_{C\text{-}F}$ = 21.0 Hz), 43.4, 37.7, 35.2, 34.9, 32.7, 27.5; **^{19}F NMR** (376 MHz, $CDCl_3$) δ −95.6 (d, *J* = 275.6 Hz), −96.7 (d, *J* = 275.6 Hz). **FT-IR**: ν (cm^{-1}) 2964, 2927, 2859, 1667, 1442, 1180, 1116, 1021, 752, 700. **HRMS** [ESI] calcd for $C_{20}H_{27}F_2N_2O_2$ $[M+H]^+$ 365.2035, found 365.2038.

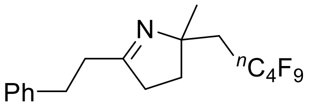

3d, yellow oil. **^{1}H NMR** (400 MHz, $CDCl_3$) δ 7.30-7.26 (m, 2H), 7.21-7.17 (m, 3H), 2.94-2.90 (m, 2H), 2.66-2.62 (m, 2H), 2.60-2.41 (m, 3H), 2.25-2.11 (m, 1H), 2.01-1.93 (m, 1H), 1.85-1.79 (m, 1H), 1.30 (d, $J_{C\text{-}F}$ = 1.6 Hz, 3H); **^{13}C NMR** (100 MHz, $CDCl_3$) δ 175.8, 140.9, 128.4, 128.3, 126.1, 73.1, 40.2 (t, $J_{C\text{-}F}$ = 19.9 Hz), 37.9, 35.0, 34.7, 32.6, 27.0 (d, $J_{C\text{-}F}$ = 2.9 Hz); **^{19}F NMR** (376 MHz, $CDCl_3$) δ −81.1 (t, *J* = 9.6 Hz, 3F), −110.1-−113.5 (m, 2F), −124.7-−124.8 (m, 2F), −125.7-−125.8 (m, 2F). **FT-IR**: ν (cm^{-1}) 2970, 2361, 1646, 1351, 1218, 1132, 879, 737, 699. **HRMS** [ESI] calcd for $C_{18}H_{19}F_9N$ $[M+H]^+$ 420.1368, found 420.1371.

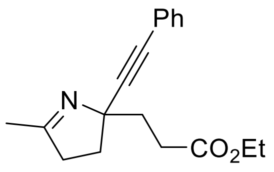

3e, yellow oil. **^{1}H NMR** (400 MHz, $CDCl_3$) δ 7.43-7.40 (m, 2H), 7.28-7.27 (m, 3H), 4.12 (q, *J* = 7.1 Hz, 2H), 2.79-2.56 (m, 4H), 2.36-2.29 (m, 1H), 2.12-1.95 (m, 6H), 1.24 (t, *J* = 7.0 Hz, 3H); **^{13}C NMR** (100 MHz, $CDCl_3$) δ 175.6, 173.6, 131.7, 128.1, 128.0, 123.1, 91.5, 84.0, 73.2, 60.4, 39.0, 36.9, 36.8, 30.8, 19.9, 14.2. **FT-IR**: ν (cm^{-1}) 2979, 2930, 1730, 1644, 1375, 1288, 1177, 1025, 757, 692. **HRMS** [ESI] calcd for $C_{18}H_{22}NO_2$ $[M+H]^+$ 284.1645, found 284.1644.

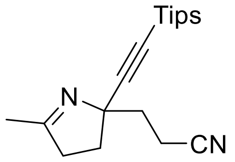

3f, colorless oil. **^{1}H NMR** (400 MHz, $CDCl_3$) δ 2.78-2.68 (m, 3H), 2.62-2.54 (m, 1H), 2.33-2.26 (m, 1H), 2.09-1.87 (m, 6H), 1.06-1.05 (m, 21H); **^{13}C NMR** (100 MHz, $CDCl_3$) δ 176.4, 120.0, 108.5, 85.5, 73.0, 39.0, 37.6, 19.7, 18.6, 13.7, 11.1. **FT-IR**: ν (cm^{-1}) 2942, 2865, 2162, 1647, 1462, 996, 882, 675, 661. **HRMS** [ESI] calcd for $C_{19}H_{32}N_2NaSi$ $[M+Na]^+$ 339.2227, found 339.2231.

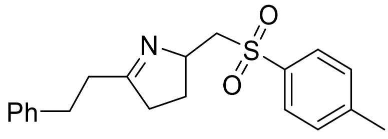

3g, colorless oil. **^{1}H NMR** (400 MHz, $CDCl_3$) δ 7.81 (d, *J* = 8.0 Hz, 2H), 7.35 (d, *J* = 8.0 Hz, 2H), 7.28-7.25 (m, 2H), 7.20-7.14 (m, 3H), 4.32-4.26 (m, 1H), 3.67 (dd, *J* = 14.0, 3.6 Hz, 1H), 3.01 (dd, *J* = 14.0, 10.0 Hz, 1H), 2.84 (t, *J* = 8.0 Hz, 2H), 2.61-2.42 (m, 7H), 2.28-2.18 (m, 1H), 1.82-1.72 (m, 1H); **^{13}C NMR** (100 MHz, $CDCl_3$) δ 179.2, 144.7, 141.0, 136.8, 129.8, 128.4, 128.2, 128.1, 126.1, 66.9, 61.4, 38.1, 35.1, 32.3, 28.7, 21.6. **FT-IR**: ν (cm^{-1}) 2923, 2361, 2342, 1598, 1313, 1301, 1288, 1143, 1086, 816, 755, 700. **HRMS** [ESI] calcd for $C_{20}H_{23}NO_2S$ $[M+Na]^+$ 364.1342, found 364.1352.

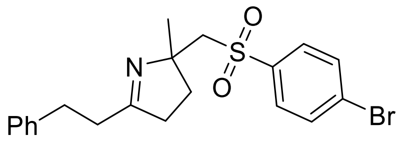

3h, white solid, mp. 64-65 °C. **^{1}H NMR** (400 MHz, $CDCl_3$) δ 7.75-7.67 (m, 4H), 7.28-7.24 (m, 2H), 7.18-7.15 (m, 3H), 3.44 (d, *J* = 14.4 Hz, 1H), 3.28 (d, *J* = 14.4 Hz, 1H), 2.87-2.73 (m, 2H), 2.62-2.52 (m, 4H), 2.46-2.38 (m, 1H), 1.83-1.76 (m, 1H), 1.34 (s, 3H); **^{13}C NMR** (100 MHz, $CDCl_3$) δ 177.0, 140.8, 140.4, 132.4, 129.3, 128.6, 128.4, 128.2, 126.1, 74.0, 65.0, 38.2, 35.0, 33.2, 32.3, 27.7. **FT-IR**: ν (cm^{-1}) 2968, 2923, 1572, 1306, 1274, 1142, 1083, 1065, 789, 722, 701. **HRMS** [ESI] calcd for $C_{20}H_{22}BrNO_2S$ $[M+Na]^+$ 442.0447, found 442.0437.

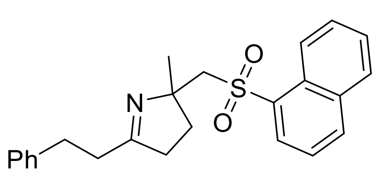

3i, colorless oil, **^{1}H NMR** (400 MHz, $CDCl_3$) δ 8.74 (d, *J* = 8.8 Hz, 1H), 8.28 (dd, *J* = 7.2, 1.2 Hz, 1H), 8.11 (d, *J* = 8.4 Hz, 1H), 7.96 (d, *J* = 8.4 Hz, 1H), 7.73-7.69 (m, 1H), 7.64-7.56 (m, 2H), 7.22-7.19 (m, 2H), 7.14-7.09 (m, 3H), 3.67 (d, *J* = 14.0 Hz, 1H), 3.38 (d, *J* = 14.0 Hz, 1H), 2.84-2.70 (m, 2H), 2.63-2.58 (m, 2H), 2.54-2.45 (m, 3H), 1.88-1.81 (m, 1H), 1.39 (s, 3H); **^{13}C NMR** (100 MHz, $CDCl_3$) δ 176.9, 140.9, 136.3, 135.0, 134.1, 129.9, 129.2, 128.7, 128.3, 128.2, 126.9, 126.0, 124.3, 124.1, 74.2, 64.5, 38.3, 34.9, 33.4, 32.3, 27.4. **FT-IR**: ν (cm^{-1}) 2921, 1308, 1273, 1154, 1115, 828, 804, 771, 759. **HRMS** [ESI] calcd for $C_{24}H_{26}NO_2S$ $[M+H]^+$ 392.1679, found 392.1665.

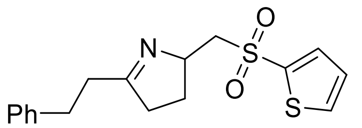

3j, colorless oil, **^{1}H NMR** (400 MHz, $CDCl_3$) δ 7.73-7.70 (m, 2H), 7.29-7.25 (m, 2H), 7.20-7.14 (m, 4H), 4.40-4.34 (m, 1H), 3.79 (dd, *J* = 14.0, 3.8 Hz, 1H), 3.14 (dd, *J* = 14.0, 10.0 Hz, 1H), 2.89-2.85 (m, 2H), 2.63-2.44 (m, 4H), 2.30-2.21 (m, 1H), 1.82-1.73 (m, 1H); **^{13}C NMR** (100 MHz, $CDCl_3$) δ 179.4, 140.9, 140.8, 134.1, 134.0, 128.4, 128.2, 127.9, 126.1, 67.1, 62.8, 38.1, 35.1, 32.3, 28.7; **FT-IR**: ν (cm^{-1}) 2923, 2361, 2341, 1638, 1402, 1312, 1227, 1139, 1015, 725, 700. **HRMS** [ESI] calcd for $C_{17}H_{20}NO_2S_2$ $[M+H]^+$ 334.0930, found 334.0922.

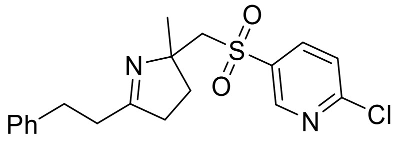

3k, yellow solid, mp. 68-69 °C. **^{1}H NMR** (400 MHz, $CDCl_3$) δ 8.84 (d, *J* = 2.0 Hz, 1H), 8.09 (d, *J* = 8.4, 3.0 Hz, 1H), 7.49 (d, *J* = 8.4 Hz, 1H), 7.29-7.25 (m, 2H), 7.19-7.16 (m, 3H), 3.45 (d, *J* = 14.8 Hz, 1H), 3.39 (d, *J* = 14.8 Hz, 1H), 2.85-2.69 (m, 2H), 2.64-2.51 (m, 4H), 2.42-2.34 (m, 1H), 1.85-1.78 (m, 1H), 1.33 (s, 3H); **^{13}C NMR** (100 MHz, $CDCl_3$) δ 177.4, 156.3, 149.5, 140.7, 138.3, 136.8, 128.4, 128.2, 126.1, 124.5, 73.9, 65.7, 38.1, 35.0, 33.5, 32.3, 27.8. **FT-IR**: ν (cm^{-1}) 2966, 2929, 1571, 1449, 1318, 1276, 1144, 1107, 790, 699. **HRMS** [ESI] calcd for $C_{19}H_{22}ClN_2O_2S$ $[M+H]^+$ 377.1085, found 377.1088.

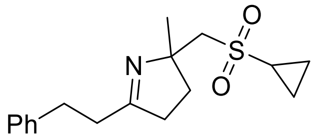

3l, colorless oil. **^{1}H NMR** (400 MHz, $CDCl_3$) δ 7.30-7.26 (m, 2H), 7.21-7.17 (m, 3H), 3.33 (d, *J* = 14.4 Hz, 1H), 3.27 (d, *J* = 14.4 Hz, 1H), 2.95-2.90 (m, 2H), 2.70-2.60 (m, 4H), 2.54-2.48 (m, 1H), 2.43-2.35 (m, 1H), 1.84-1.77 (m, 1H), 1.38 (s, 3H), 1.23-1.19 (m, 2H), 1.01-0.93 (m, 2H); **^{13}C NMR** (100 MHz, $CDCl_3$) δ 177.3, 140.9, 128.4, 128.2, 126.1, 73.6, 62.8, 38.1, 35.1, 33.5, 32.5, 32.1, 27.8, 5.2, 4.9. **FT-IR**: ν (cm^{-1}) 2921, 1309, 1275, 1115, 828, 772, 759, 700. **HRMS** [ESI] calcd for $C_{17}H_{23}NO_2S$ $[M+Na]^+$ 328.1342, found 328.1340.

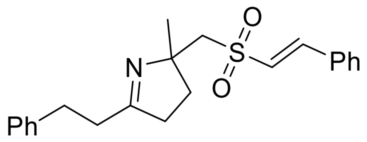

3m, colorless oil. **^{1}H NMR** (400 MHz, $CDCl_3$) δ 7.52-7.48 (m, 3H), 7.42-7.36 (m, 3H), 7.26-7.15 (m, 3H), 7.10 (d, *J* = 7.2 Hz, 2H), 6.96 (d, *J* = 15.2 Hz, 1H), 3.36 (d, *J* = 0.8 Hz, 2H), 2.94-2.81 (m, 2H), 2.66-2.56 (m, 4H), 2.46-2.38 (m, 1H), 1.83-1.76 (m, 1H), 1.38 (s, 3H); **^{13}C NMR** (100 MHz, $CDCl_3$) δ 177.3, 142.2, 140.9, 132.4, 131.0, 129.0, 128.4, 128.3, 128.1, 127.7, 126.0, 73.7, 64.5, 38.2, 35.1, 33.5, 32.4, 28.0. **FT-IR**: ν (cm^{-1}) 2921, 1273, 1178, 1115, 827, 758. **HRMS** [ESI] calcd for $C_{22}H_{26}NO_2S$ $[M+H]^+$ 368.1679, found 368.1682.

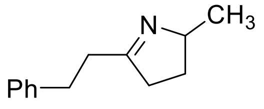

3n, colorless oil. **^{1}H NMR** (400 MHz, $CDCl_3$) δ 7.30-7.26 (m, 2H), 7.22-7.17 (m, 3H), 4.08-4.02 (m, 1H), 2.95-2.91 (m, 2H), 2.65-2.61 (m, 2H), 2.57-2.37 (m, 2H), 2.11-2.02 (m, 1H), 1.41-1.31 (m, 1H), 1.25 (d, *J* = 6.4 Hz, 3H); **^{13}C NMR** (100 MHz, $CDCl_3$) δ 176.2, 141.4, 128.4, 128.2, 125.9, 67.7, 37.7, 35.4, 32.7, 30.6, 22.0. **FT-IR**: ν (cm^{-1}) 2960, 2926, 2361, 2342, 1642, 1454, 1077, 749, 699. **HRMS** [ESI] calcd for $C_{13}H_{18}N$ $[M+H]^+$ 188.1434,

found 188.1444.

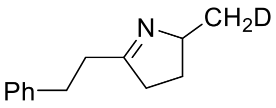

3o, yellow oil. **^{1}H NMR** (400 MHz, $CDCl_3$) δ 7.30-7.26 (m, 2H), 7.22-7.17 (m, 3H), 4.07-4.00 (m, 1H), 2.95-2.90 (m, 2H), 2.64-2.60 (m, 2H), 2.52-2.39 (m, 2H), 2.09-2.04 (m, 1H), 1.41-1.32 (m, 1H), 1.23 (dt, *J* = 6.8, 1.6 Hz, 2H); **^{13}C NMR** (100 MHz, $CDCl_3$) δ 176.2, 141.4, 128.4, 128.2, 125.9, 67.6, 37.7, 35.4, 32.7, 30.6, 21.7 (t, $J_{C\text{-}D}$ = 19.2 Hz). **FT-IR**: ν (cm^{-1}) 2957, 2929, 2360, 1641, 1496, 1454, 1134, 1077, 748, 698. **HRMS** [ESI] calcd for $C_{13}H_{17}DN$ $[M+H]^+$ 189.1497, found 189.1503.

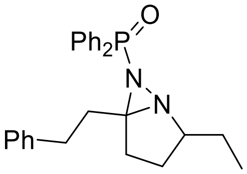

4a, white solid, mp. 110-111 °C. **^{1}H NMR** (400 MHz, $CDCl_3$) δ 8.09-8.04 (m, 2H), 7.96-7.91 (m, 2H), 7.55-7.38 (m, 6H), 7.22-7.18 (m, 2H), 7.13-7.08 (m, 3H), 2.94-2.87 (m, 1H), 2.76-2.69 (m, 1H), 2.58-2.38 (m, 3H), 2.33-2.28 (m, 1H), 1.80-1.71 (m, 1H), 1.67-1.61 (m, 1H), 1.40-1.30 (m, 1H), 1.04-0.86 (m, 2H), 0.55 (t, *J* = 7.4 Hz, 3H); **^{13}C NMR** (100 MHz, $CDCl_3$) δ 141.1, 133.2 (d, $J_{C\text{-}P}$ = 127.7 Hz), 133.2 (d, $J_{C\text{-}P}$ = 124.9 Hz), 132.0 (d, $J_{C\text{-}P}$ = 9.2 Hz), 131.9 (d, $J_{C\text{-}P}$ = 2.9 Hz), 131.8 (d, $J_{C\text{-}P}$ = 8.1 Hz), 131.5 (d, $J_{C\text{-}P}$ = 2.6 Hz), 128.5 (d, $J_{C\text{-}P}$ = 11.6 Hz), 128.3, 128.2, 127.9 (d, $J_{C\text{-}P}$ = 12.8 Hz), 125.8, 78.2 (d, $J_{C\text{-}P}$ = 8.7 Hz), 68.1 (d, $J_{C\text{-}P}$ = 5.1 Hz), 33.7 (d, $J_{C\text{-}P}$ = 3.4 Hz), 32.4, 31.4 (d, $J_{C\text{-}P}$ = 3.5 Hz), 26.4, 25.6, 11.4; **^{31}P NMR** (162 MHz, $CDCl_3$) δ 28.7 (s). **FT-IR**: ν (cm^{-1}) 2970, 2924, 1439, 1208, 1118, 971, 725, 697. **HRMS** [ESI] calcd for $C_{26}H_{29}N_2NaOP$ $[M+Na]^+$ 439.1910, found 439.1908.

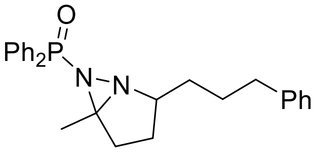

4b, colorless oil. **^{1}H NMR** (400 MHz, $CDCl_3$) δ 7.99-7.91 (m, 4H), 7.55-7.40 (m, 6H), 7.23-7.20 (m, 2H), 7.15-7.11 (m, 1H), 6.98 (d, *J* = 6.8 Hz, 2H), 3.08-3.00 (m, 1H), 2.34-2.29 (m, 1H), 2.15-2.02 (m, 2H), 1.77-1.69 (m, 4H), 1.64-1.57 (m, 1H), 1.47-1.31 (m, 3H), 1.07-0.93 (m, 2H); **^{13}C NMR** (100 MHz, $CDCl_3$) δ 142.2, 133.3 (d, $J_{C\text{-}P}$ = 131.2 Hz), 133.0 (d, $J_{C\text{-}P}$ = 121.2 Hz), 132.1 (d, $J_{C\text{-}P}$ = 9.1 Hz), 131.8 (d, $J_{C\text{-}P}$ = 2.5 Hz), 131.6 (d, $J_{C\text{-}P}$ = 8.0 Hz), 131.6, 128.5 (d, $J_{C\text{-}P}$ = 11.6 Hz), 128.2, 128.1 (d, $J_{C\text{-}P}$ = 12.8 Hz), 128.0, 125.5, 74.6 (d, $J_{C\text{-}P}$ = 8.6 Hz), 66.5 (d, $J_{C\text{-}P}$ = 5.3 Hz), 35.5, 34.1 (d, $J_{C\text{-}P}$ = 3.8 Hz), 32.4, 28.7, 26.9, 17.8 (d, $J_{C\text{-}P}$ = 3.8 Hz); **^{31}P NMR** (162 MHz, $CDCl_3$) δ 29.4 (s). **FT-IR**: ν (cm^{-1}) 2930, 1438, 1211, 1122, 1108, 907, 725, 695. **HRMS** [ESI] calcd for $C_{26}H_{29}N_2NaOP$ $[M+Na]^+$ 439.1910, found 439.1924.

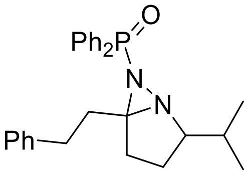

4c, white solid, mp. 163-164 °C. **^{1}H NMR** (400 MHz, $CDCl_3$) δ 8.09-8.07 (m, 2H), 8.00-7.95 (m, 2H), 7.52-7.42 (m, 6H), 7.22-7.18 (m, 2H), 7.14-7.08 (m, 3H), 2.80-2.69 (m, 1H), 2.62-2.51 (m, 2H), 2.48-2.31 (m, 3H), 1.80-1.64 (m, 2H), 1.48-1.38 (m, 1H), 0.99-0.91 (m, 1H), 0.66 (d, *J* = 6.4 Hz, 3H), 0.50 (d, *J* = 6.4 Hz, 3H); **^{13}C NMR** (100 MHz, $CDCl_3$) δ 141.2, 133.7 (d, $J_{C\text{-}P}$ = 122.1 Hz), 133.3 (d, $J_{C\text{-}P}$ = 130.0 Hz), 132.2 (d, $J_{C\text{-}P}$ = 9.1 Hz), 131.9 (d, $J_{C\text{-}P}$ = 2.5 Hz), 131.8 (d, $J_{C\text{-}P}$ = 8.1 Hz), 131.6 (d, $J_{C\text{-}P}$ = 2.7 Hz), 128.6 (d, $J_{C\text{-}P}$ = 11.6 Hz), 128.3, 128.2, 128.0 (d, $J_{C\text{-}P}$ = 12.6

Hz), 125.8, 77.9 (d, J_{C-P} = 8.5 Hz), 73.6 (d, J_{C-P} = 5.1 Hz), 33.7 (d, J_{C-P} = 3.4 Hz), 32.4, 32.0, 31.6 (d, J_{C-P} = 3.5 Hz), 25.7, 20.6, 20.2; **^{31}P NMR** (162 MHz, $CDCl_3$) δ 27.9 (s). **FT-IR**: ν (cm^{-1}) 2952, 2915, 2866, 1437, 1206, 1125, 1069, 1015, 728, 699, 624. **HRMS** [ESI] calcd for $C_{27}H_{32}N_2OP$ $[M+H]^+$ 431.2247, found 431.2245.

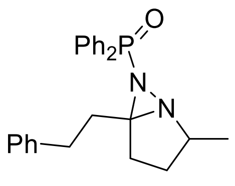

4d, colorless oil. **^{1}H NMR** (400 MHz, $CDCl_3$) δ 8.10-8.05 (m, 2H), 7.94-7.89 (m, 2H), 7.56-7.39 (m, 6H), 7.22-7.18 (m, 2H), 7.14-7.07 (m, 3H), 3.18-3.09 (m, 1H), 2.68-2.53 (m, 2H), 2.50-2.40 (m, 2H), 2.33-2.27 (m, 1H), 1.83-1.74 (m, 1H), 1.65-1.59 (m, 1H), 1.41-1.31 (m, 1H), 0.61 (d, J = 6.4 Hz, 3H); **^{13}C NMR** (100 MHz, $CDCl_3$) δ 141.1, 133.3 (d, J_{C-P} = 131.2 Hz), 132.9 (d, J_{C-P} = 121.6 Hz), 132.0 (d, J_{C-P} = 2.4 Hz), 132.0 (d, J_{C-P} = 9.2 Hz), 131.9 (d, J_{C-P} = 8.1 Hz), 131.6 (d, J_{C-P} = 2.7 Hz), 128.6 (d, J_{C-P} = 11.6 Hz), 128.3, 128.2, 128.0 (d, J_{C-P} = 12.8 Hz), 125.8, 78.5 (d, J_{C-P} = 8.7 Hz), 61.6 (d, J_{C-P} = 5.1 Hz), 33.6 (d, J_{C-P} = 3.5 Hz), 32.5, 31.9 (d, J_{C-P} = 3.6 Hz), 27.9, 16.9; **^{31}P NMR** (162 MHz, $CDCl_3$) δ 29.1 (s). **FT-IR**: ν (cm^{-1}) 2971, 2929, 1735, 1438, 1207, 1122, 962, 726, 696. **HRMS** [ESI] calcd for $C_{25}H_{27}N_2OP$ $[M+Na]^+$ 425.1753, found 425.1757.

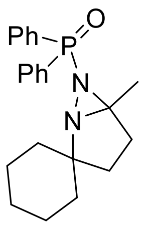

4e, white solid, mp. 145-146 °C. **^{1}H NMR** (400 MHz, $CDCl_3$) δ 7.99-7.94 (m, 2H), 7.92-7.86 (m, 2H), 7.55-7.37 (m, 6H), 2.30-2.25 (m, 1H), 1.84-1.70 (m, 5H), 1.56-1.50 (m, 2H), 1.40-1.14 (m, 6H), 1.02-0.81 (m, 3H); **^{13}C NMR** (100 MHz, $CDCl_3$) δ 133.1 (d, J_{C-P} = 120.9 Hz), 133.0 (d, J_{C-P} = 131.8 Hz), 132.1 (d, J_{C-P} = 9.1 Hz), 131.7 (d, J_{C-P} = 8.0 Hz), 131.5 (d, J_{C-P} = 2.8 Hz), 128.4 (d, J_{C-P} = 11.7 Hz), 127.8 (d, J_{C-P} = 12.8 Hz), 73.6 (d, J_{C-P} = 8.6 Hz), 68.8 (d, J_{C-P} = 4.8 Hz), 35.1, 33.4, 33.2 (d, J_{C-P} = 3.7 Hz), 30.4, 25.6, 22.9, 22.7, 18.4 (d, J_{C-P} = 3.8 Hz); **^{31}P NMR** (162 MHz, $CDCl_3$) δ 29.6 (s). **FT-IR**: ν (cm^{-1}) 2950, 2929, 1438, 1212, 1115, 962, 908, 725, 694, 660. **HRMS** [ESI] calcd for $C_{22}H_{27}N_2NaOP$ $[M+Na]^+$ 389.1753, found 389.1752.

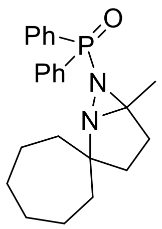

4f, white solid, mp. 139-140 °C. **^{1}H NMR** (400 MHz, $CDCl_3$) δ 7.98-7.93 (m, 2H), 7.86-7.81 (m, 2H), 7.55-7.51 (m, 1H), 7.49-7.42 (m, 3H), 7.41-7.36 (m, 2H), 2.26-2.20 (m, 2H), 1.75 (s, 3H), 1.70-1.51 (m, 4H), 1.43-1.18 (m, 6H), 1.11-1.02 (m, 2H), 0.97-0.91 (m, 1H), 0.72-0.66 (m, 1H); **^{13}C NMR** (100 MHz, $CDCl_3$) δ 133.0 (d, J_{C-P} = 131.1 Hz), 132.7 (d, J_{C-P} = 121.8 Hz), 132.0 (d, J_{C-P} = 9.2 Hz), 131.8 (d, J_{C-P} = 8.1 Hz), 131.8 (d, J_{C-P} = 3.1 Hz), 131.4 (d, J_{C-P} = 2.7 Hz), 128.4 (d, J_{C-P} = 11.7 Hz), 127.9 (d, J_{C-P} = 12.9 Hz), 75.0 (d, J_{C-P} = 8.6 Hz), 72.7 (d, J_{C-P} = 4.6 Hz), 37.3, 36.0, 32.9 (d, J_{C-P} = 3.7 Hz), 32.6, 30.6, 30.2, 23.2, 22.9, 18.4 (d, J_{C-P} = 3.9 Hz); **^{31}P NMR** (162 MHz, $CDCl_3$) δ 30.2 (s). **FT-IR**: ν (cm^{-1}) 2926, 2849, 1437, 1209, 1124, 1030, 930, 729, 698, 654. **HRMS** [ESI] calcd for $C_{23}H_{30}N_2OP$ $[M+H]^+$ 381.2090, found 381.2094.

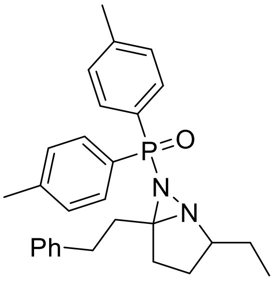

4g, white solid, mp. 109-110 ºC. **^{1}H NMR** (400 MHz, $CDCl_3$) δ 7.93 (dd, *J* = 10.6, 8.0 Hz, 2H), 7.81 (dd, *J* = 12.0, 8.0 Hz, 2H), 7.28-7.25 (m, 2H), 7.22-7.18 (m, 4H), 7.13-7.09 (m, 3H), 2.94-2.86 (m, 1H), 2.74-2.68 (m, 1H), 2.58-2.39 (m, 3H), 2.37 (s, 3H), 2.35 (s, 3H), 2.33-2.27 (m, 1H), 1.79-1.70 (m, 1H), 1.67-1.60 (m, 1H), 1.40-1.32 (m, 1H), 1.08-1.01 (m, 1H), 0.96-0.85 (m, 1H), 0.57 (t, *J* = 7.4 Hz, 3H); **^{13}C NMR** (100 MHz, $CDCl_3$) δ 142.2 (d, J_{C-P} = 2.8 Hz), 141.7 (d, J_{C-P} = 2.8 Hz), 141.3, 132.0 (d, J_{C-P} = 9.5 Hz), 131.7 (d, J_{C-P} = 8.5 Hz), 130.2 (d, J_{C-P} = 127.9 Hz), 130.2 (d, J_{C-P} = 129.3 Hz), 129.2 (d, J_{C-P} = 11.9 Hz), 128.6 (d, J_{C-P} = 13.1 Hz), 128.3, 128.1, 125.7, 77.9 (d, J_{C-P} = 8.6 Hz), 77.2, 68.1 (d, J_{C-P} = 5.1 Hz), 33.7 (d, J_{C-P} = 3.4 Hz), 32.5, 31.4 (d, J_{C-P} = 3.6 Hz), 26.5, 25.7, 21.5 (d, J_{C-P} = 5.8 Hz), 11.4; **^{31}P NMR** (162 MHz, $CDCl_3$) δ 29.2 (s). **FT-IR**: ν (cm^{-1}) 2958, 2919, 2855, 1456, 1199, 1121, 1105, 1033, 972, 806, 713, 702, 661, 627. **HRMS** [ESI] calcd for $C_{28}H_{34}N_2OP$ $[M+H]^+$ 445.2403, found 445.2401.

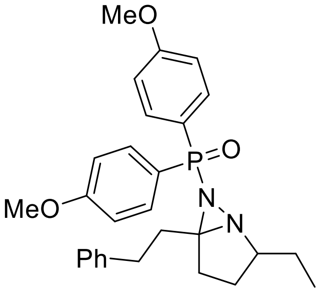

4h, white solid, mp. 122-123 ºC. **^{1}H NMR** (400 MHz, $CDCl_3$) δ 7.99-7.94 (m, 2H), 7.86-7.81 (m, 2H), 7.23-7.19 (m, 2H), 7.14-7.10 (m, 3H), 6.99-6.91 (m, 4H), 3.82 (s, 3H), 3.82 (s, 3H), 2.94-2.87 (m, 1H), 2.74-2.67 (m, 1H), 2.58-2.36 (m, 3H), 2.32-2.26 (m, 1H), 1.79-1.60 (m, 2H), 1.39-1.29 (m, 1H), 1.09-0.88 (m, 2H), 0.60 (t, *J* = 7.4 Hz, 3H); **^{13}C NMR** (100 MHz, $CDCl_3$) δ 162.4, 162.1, 141.3, 133.8 (d, J_{C-P} = 10.4 Hz), 133.5 (d, J_{C-P} = 9.3 Hz), 128.4, 128.2, 125.8, 125.0 (d, J_{C-P} = 137.8 Hz), 124.9 (d, J_{C-P} = 129.0 Hz), 114.0 (d, J_{C-P} = 12.5 Hz), 113.5 (d, J_{C-P} = 13.8Hz), 77.9 (d, J_{C-P} = 8.6 Hz), 68.1 (d, J_{C-P} = 5.1 Hz), 55.2, 33.7 (d, J_{C-P} = 3.4 Hz), 32.5, 31.4 (d, J_{C-P} = 3.5 Hz), 26.5, 25.8, 11.5; **^{31}P NMR** (162 MHz, $CDCl_3$) δ 29.0 (s). **FT-IR**: ν (cm^{-1}) 2960, 2934, 1595, 1500, 1292, 1247, 1124, 1106, 1022, 980, 833, 799, 702. **HRMS** [ESI] calcd for $C_{28}H_{34}N_2O_3P$ $[M+H]^+$ 477.2302, found 477.2295.

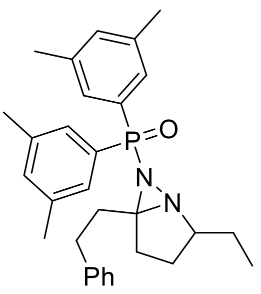

4i, white solid, mp. 147-148 ºC. **^{1}H NMR** (400 MHz, $CDCl_3$) δ 7.65 (d, *J* = 11.2 Hz, 2H), 7.52 (d, *J* = 12.4 Hz, 2H), 7.22-7.18 (m, 2H), 7.14-7.08 (m, 5H), 2.93-2.86 (m, 1H), 2.73-2.67 (m, 1H), 2.58-2.41 (m, 3H), 2.35 (s, 6H), 2.31 (s, 6H), 2.29-2.24 (m, 1H), 1.78-1.60 (m, 2H), 1.42-1.31 (m, 1H), 1.08-0.86 (m, 2H), 0.58 (t, *J* = 7.4 Hz, 3H); **^{13}C NMR** (100 MHz, $CDCl_3$) δ 141.3, 138.1 (d, J_{C-P} = 12.3 Hz), 137.5 (d, J_{C-P} = 13.4 Hz), 133.0 (d, J_{C-P} = 129.1 Hz), 133.0 (d, J_{C-P} = 120.5 Hz), 133.6 (d, J_{C-P} = 2.8 Hz), 133.1 (d, J_{C-P} = 2.8 Hz), 129.6 (d, J_{C-P} = 9.2 Hz), 129.4 (d, J_{C-P} = 8.1 Hz), 128.4, 128.2, 125.7, 78.0 (d, J_{C-P} = 8.6 Hz), 68.1 (d, J_{C-P} = 5.1 Hz), 33.6 (d, J_{C-P} = 3.4 Hz), 32.5, 31.5 (d, J_{C-P} = 3.6 Hz), 26.6, 25.6, 21.3, 21.2, 11.5; **^{31}P NMR** (162 MHz, $CDCl_3$) δ 29.8 (s). **FT-IR**: ν (cm^{-1}) 2955, 2921, 1455, 1216, 1124, 1012, 875, 855, 695. **HRMS** [ESI] calcd for $C_{30}H_{37}N_2NaOP$ $[M+Na]^+$ 495.2536, found 495.2538.

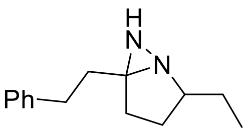

5a, colorless oil. **^{1}H NMR** (400 MHz, $CDCl_3$) δ 7.30-7.26 (m, 2H), 7.20-7.18 (m, 3H), 2.95-2.88 (m, 1H), 2.76 (t, *J* = Hz, 2H), 2.23-2.15 (m, 2H), 2.00-1.92 (m, 1H), 1.80-1.68 (m, 2H), 1.62-1.55 (m, 1H), 1.51-1.40 (m, 1H), 1.27-1.17 (m, 1H), 0.98 (t, *J* = 7.4 Hz, 3H); **^{13}C NMR** (100 MHz, $CDCl_3$) δ 141.1, 128.5, 128.2, 126.1, 67.8, 66.6, 37.5, 31.7, 29.7, 26.4, 25.7, 11.9. **FT-IR**: ν (cm^{-1}) 3207, 2961, 2931, 1454, 1379, 1269, 967, 747, 701. **HRMS** [ESI] calcd for $C_{14}H_{21}N_2$ $[M+H]^+$ 217.1699, found 217.1707.

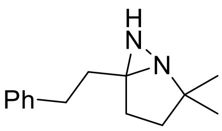

5b, colorless oil. **^{1}H NMR** (400 MHz, $CDCl_3$) δ 7.30-7.26 (m, 2H), 7.21-7.17 (m, 3H), 2.78 (t, *J* = 8.2 Hz, 2H), 2.16-1.98 (m, 3H), 1.88-1.80 (m, 1H), 1.49-1.41 (m, 1H), 1.33-1.28 (m, 1H), 1.23 (s, 3H), 1.06 (s, 3H); **^{13}C NMR** (100 MHz, $CDCl_3$) δ 141.1, 128.5, 128.2, 126.1, 67.9, 63.7, 37.7, 33.2, 31.8, 29.0, 26.0, 25.2. **HRMS** [ESI] calcd for $C_{14}H_{21}N_2$ $[M+H]^+$ 217.1699, found 217.1703.

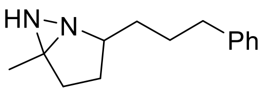

5c, colorless oil. **^{1}H NMR** (400 MHz, $CDCl_3$) δ 7.30-7.26 (m, 2H), 7.21-7.16 (m, 3H), 3.08-3.01 (m, 1H), 2.72-2.61 (m, 2H), 2.17-2.12 (m, 1H), 1.88-1.64 (m, 3H), 1.60-1.48 (m, 5H), 1.29-1.20 (m, 2H); **^{13}C NMR** (100 MHz, $CDCl_3$) δ 142.5, 128.4, 128.2, 125.6, 65.0, 64.9, 36.1, 32.3, 31.7, 29.3, 27.0, 21.6. **FT-IR**: ν (cm^{-1}) 2928, 2859, 1453, 747, 698. **HRMS** [ESI] calcd for $C_{14}H_{20}N_2Na$ $[M+Na]^+$ 239.1519, found 239.1512.

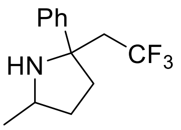

6, dr = 1.2:1, colorless oil. **^{1}H NMR** (400 MHz, $CDCl_3$) δ 7.49-7.42 (m, 2H, two isomers), 7.35-7.30 (m, 2H, two isomers), 7.25-7.21 (m, 1H, two isomers), 3.52-3.43 (m, 0.45H) & 3.25-3.16 (m, 0.55H) (two isomers), 2.77-2.56 (m, 2H, two isomers), 2.33-2.26 (m, 0.67H) & 2.20-2.11 (m, 1.66H) & 1.84-1.76 (m, 0.67H) (two isomers), 2.02-1.97 (m, 1H, two isomers), 1.45-1.35 (m, 1H) (two isomers), 1.18 (t, *J* = 6.4 Hz, 3H, two isomers); **^{13}C NMR** (100 MHz, $CDCl_3$) δ 146.4 & 145.1 (two isomers), 128.2 & 128.1 (two isomers), 126.7 & 126.6 (two isomers), 125.9 (q, J_{C-F} = 277.0 Hz) & 125.9 (q, J_{C-F} = 276.9 Hz) (two isomers), 125.9 & 125.7 (two isomers), 65.0 (q, J_{C-F} = 1.3 Hz) & 64.9 (q, J_{C-F} = 1.4 Hz) (two isomers), 53.0 & 52.5 (two isomers), 46.9 (q, J_{C-F} = 25.3 Hz) & 44.5 (q, J_{C-F} = 24.6 Hz) (two isomers), 39.4 & 39.0 (two isomers), 32.5 & 32.4 (two isomers), 22.3 & 22.2 (two isomers); **^{19}F NMR** (376 MHz, $CDCl_3$) δ −60.2 (s) & −60.3 (s) (two isomers). **FT-IR**: ν (cm^{-1}) 2962, 1364, 1119, 1031, 764, 701, 648. **HRMS** [ESI] calcd for $C_{13}H_{17}F_3N$ $[M+H]^+$ 244.1308, found 244.1304.

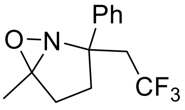

7, dr = 4.4:1, white solid, mp. 66-67 ^{o}C. **^{1}H NMR** (400 MHz, $CDCl_3$) δ 7.46-7.40 (m, 2H, two isomers), 7.32-7.18 (m, 3H, two isomers), 2.95-2.61 (m, 1.63 H) & 2.55-2.41 (m, 0.37 H) (two isomers), 2.39-2.19 (m, 2H, two isomers), 2.12-1.90 (m, 0.37H) & 1.80-1.66 (m, 1.63H) (two isomers), 1.59 (s, 0.55H) & 1.46 (s, 2.45H) (two isomers); **^{13}C NMR** (100 MHz, $CDCl_3$) δ 143.7 & 138.6 (two isomers), 128.5 & 128.3 (two isomers), 127.9 & 127.3 (two isomers), 126.7 & 126.0 (two isomers), 125.6 (q, J_{C-F} = 276.2 Hz) & 125.2 (q, J_{C-F} = 277.1 Hz)

(two isomers), 88.8 & 87.2 (two isomers), 72.2 (q, $J_{C\text{-}F}$ = 1.5 Hz) & 71.3 (q, $J_{C\text{-}F}$ = 1.9 Hz) (two isomers), 43.7 (q, $J_{C\text{-}F}$ = 27.0 Hz) & 30.8 (q, $J_{C\text{-}F}$ = 41.1 Hz) (two isomers), 30.1 & 29.7 (two isomers), 30.1 & 29.6 (two isomers), 18.8 & 18.7 (two isomers); **^{19}F NMR** (376 MHz, $CDCl_3$) δ −59.9 (s) & −60.5 (s) (two isomers). **FT-IR**: ν (cm^{-1}) 2984, 1723, 1449, 1259, 1241, 1151, 1124, 1054, 856, 800, 698, 649. **HRMS** [ESI] calcd for $C_{13}H_{15}F_3NO$ [M+H]$^+$ 258.1100, found 258.1099.

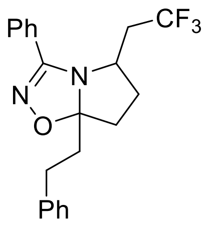

8, dr = 1.3:1, white solid, mp. 64-65 °C. *Major isomer*: **^{1}H NMR** (400 MHz, $CDCl_3$) δ 7.70-7.68 (m, 2H), 7.50-7.42 (m, 3H), 7.29-7.25 (m, 2H), 7.22-7.16 (m, 3H), 3.84-3.79 (m, 1H), 2.89-2.80 (m, 2H), 2.53-2.35 (m, 2H), 2.30-2.13 (m, 3H), 2.10-2.00 (m, 2H), 1.87-1.82 (m, 1H); **^{13}C NMR** (100 MHz, $CDCl_3$) δ 158.8, 141.6, 130.9, 128.9, 128.4, 128.3, 127.8, 126.3, 125.9, 125.5 (q, $J_{C\text{-}F}$ = 275.5 Hz), 109.3, 58.6 (q, $J_{C\text{-}F}$ = 2.8 Hz), 42.4, 38.7 (q, $J_{C\text{-}F}$ = 27.1 Hz), 35.9, 29.9, 28.8; **^{19}F NMR** (376 MHz, $CDCl_3$) δ −63.7 (s). **FT-IR**: ν (cm^{-1}) 2950, 1393, 1248, 1160, 1136, 1120, 906, 755, 700, 688. **HRMS** [ESI] calcd for $C_{21}H_{21}F_3N_2NaO$ [M+Na]$^+$ 397.1498, found 397.1505.

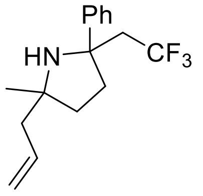

9, dr = 5.8:1, light yellow oil. **^{1}H NMR** (400 MHz, $CDCl_3$) δ 7.44-7.32 (m, 2H, two isomers), 7.26-7.22 (m, 2H, two isomers), 7.18-7.13 (m, 1H, two isomers), 5.88-5.70 (m, 1H, two isomers), 5.06-4.85 (m, 2H, two isomers), 2.65-2.44 (m, 2H, two isomers), 2.29-2.12 (m, 2H, two isomers), 1.98 (d, J = 7.2 Hz, 1.70 H) & 1.74-1.67 (m, 0.30H) (two isomers), 1.60 (t, J = 7.2 Hz, 1.57H) & 1.50-1.43 (m, 0.35 H) (two isomers), 1.15 (s, 2.56H) & 0.97 (s, 0.44H) (two isomers); **^{13}C NMR** (100 MHz, $CDCl_3$) δ 146.8 & 146.8 (two isomers), 135.6 & 135.1 (two isomers), 128.0 (two isomers), 126.5 (two isomers), 125.8 (q, $J_{C\text{-}F}$ = 277.1 Hz) (two isomers), 125.9 (two isomers), 117.9 & 117.3 (two isomers), 64.9 & 64.9 (two isomers), 61.6 (two isomers), 48.3 & 47.9 (two isomers), 47.1 (q, $J_{C\text{-}F}$ = 24.6 Hz) (two isomers), 38.6 & 38.4 (two isomers), 37.2 & 36.6 (two isomers), 28.9 & 28.8 (two isomers); **^{19}F NMR** (376 MHz, $CDCl_3$) δ −60.1 (s) & −60.1 (s) (two isomers). **FT-IR**: ν (cm^{-1}) 2962, 1361, 1258, 1118, 1078, 914, 702. **HRMS** [ESI] calcd for $C_{16}H_{21}F_3N$ [M+H]$^+$ 284.1621, found 284.1612.

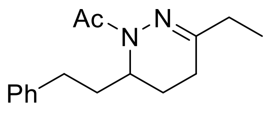

10, light yellow solid, mp. 66-67 °C. **^{1}H NMR** (400 MHz, $CDCl_3$) δ 7.30-7.26 (m, 2H), 7.21-7.17 (m, 3H), 4.61-4.57 (m, 1H), 2.91 (t, 2H, J = 7.8 Hz, 2H), 2.54 (t, J = 7.8 Hz, 2H), 2.25 (s, 3H), 2.16-1.98 (m, 2H), 1.91-1.86 (m, 1H), 1.68-1.58 (m, 1H), 1.54-1.46 (m, 1H), 1.34-1.26 (m, 1H), 0.87 (t, J = 7.4 Hz, 3H); **^{13}C NMR** (100 MHz, $CDCl_3$) δ 171.5, 151.0, 141.3, 128.3, 128.3, 126.0, 47.2, 39.2, 32.3, 23.0, 21.8, 21.4, 19.5, 10.2. **FT-IR**: ν (cm^{-1}) 2930, 1722, 1656, 1371, 1344, 1259, 1210, 1151, 800. **HRMS** [ESI] calcd for $C_{16}H_{22}N_2NaO$ [M+Na]$^+$ 281.1624, found 281.1621.

8. NMR Spectra

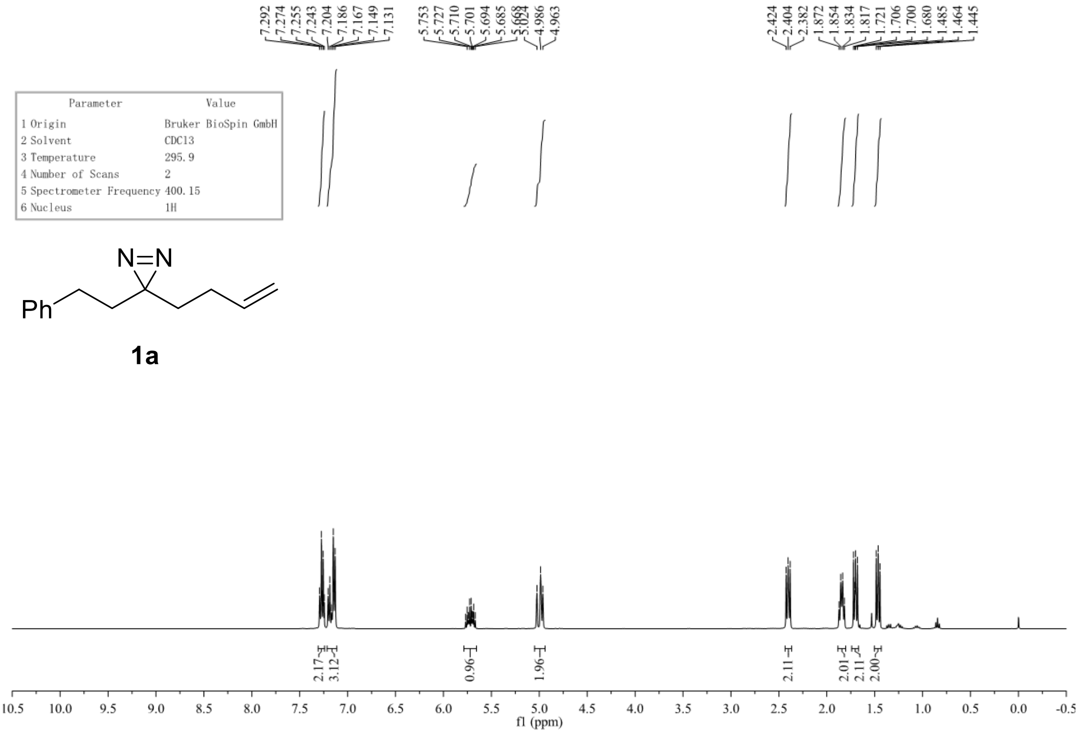

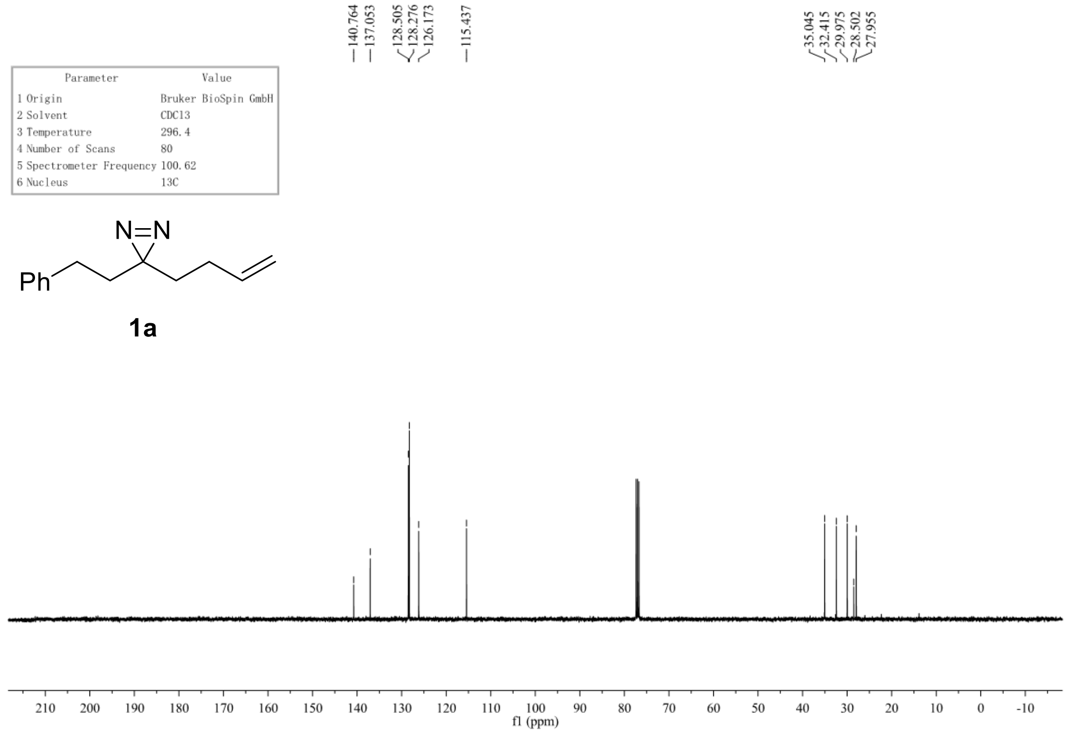

Parameter	Value
1 Origin	Bruker BioSpin GmbH
2 Solvent	CDCl3
3 Temperature	294.9
4 Number of Scans	2
5 Spectrometer Frequency	400.13
6 Nucleus	1H

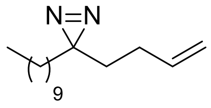

1b

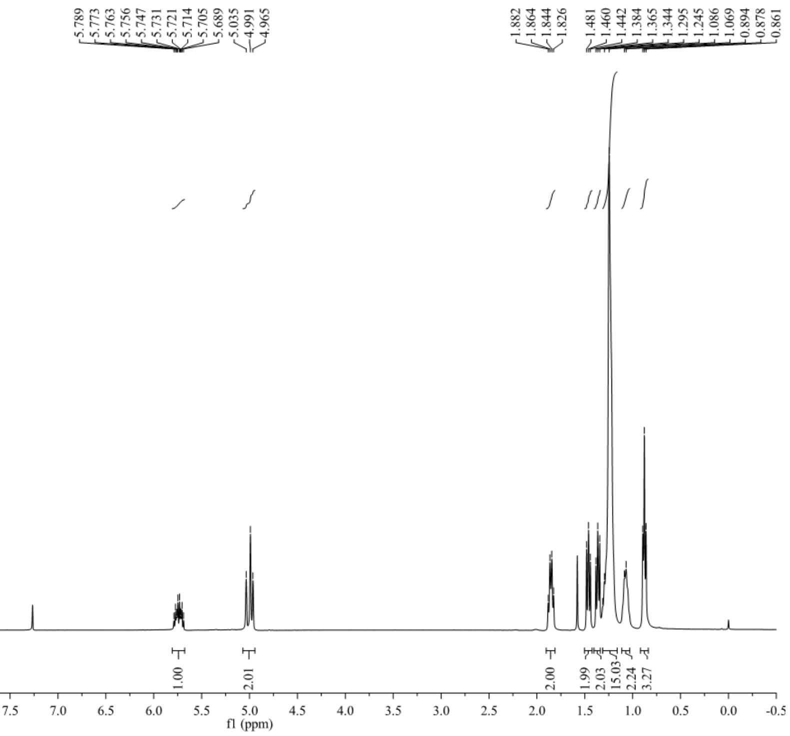

Parameter	Value
1 Origin	Bruker BioSpin GmbH
2 Solvent	CDCl3
3 Temperature	296.2
4 Number of Scans	80
5 Spectrometer Frequency	100.62
6 Nucleus	13C

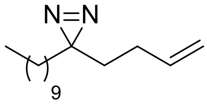

1b

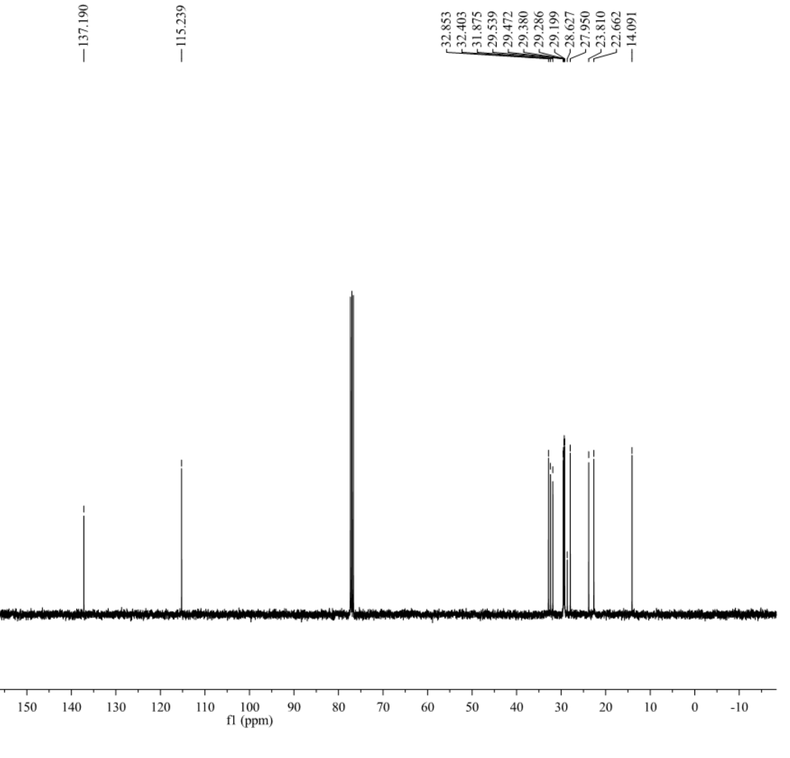

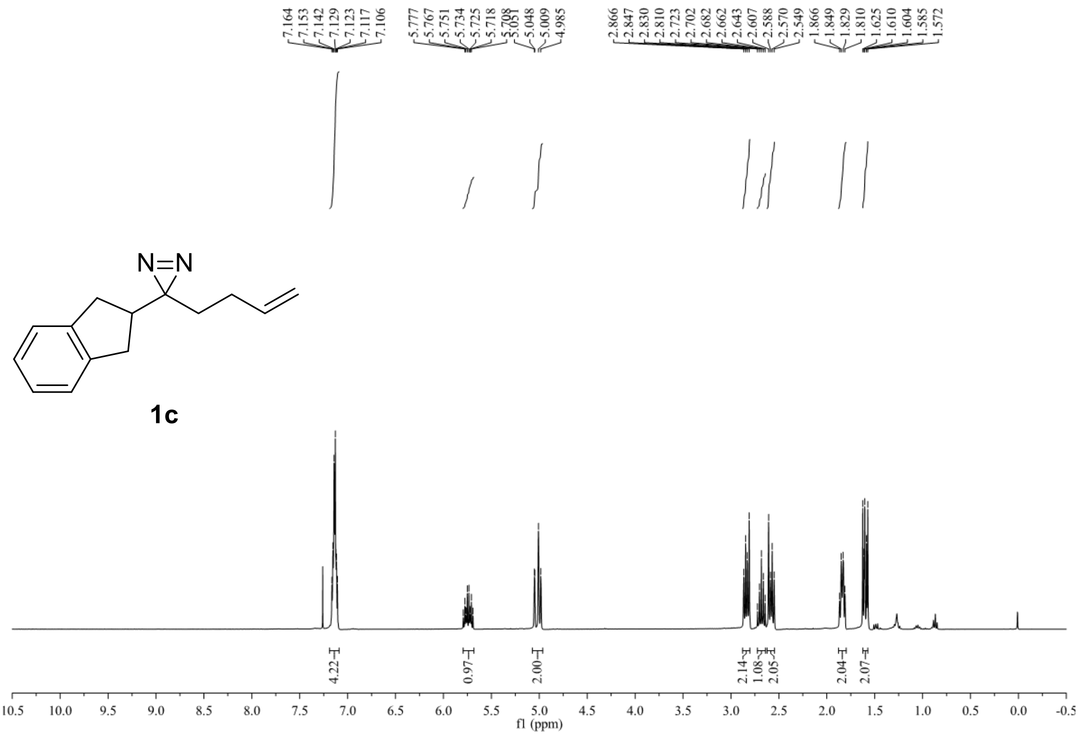
7.164 7.153 7.142 7.129 7.123 7.117 7.106
5.777 5.767 5.751 5.734 5.725 5.718 5.708 5.051 5.048 5.009 4.985
2.866 2.847 2.830 2.810 2.723 2.702 2.682 2.662 2.643 2.607 2.588 2.570 2.549 1.866 1.849 1.829 1.810 1.625 1.610 1.604 1.585 1.572
N=N
1c
4.22
0.97
2.00
2.14
1.08
2.05
2.04
2.07
10.5 10.0 9.5 9.0 8.5 8.0 7.5 7.0 6.5 6.0 5.5 5.0 4.5 4.0 3.5 3.0 2.5 2.0 1.5 1.0 0.5 0.0 -0.5
f1 (ppm)

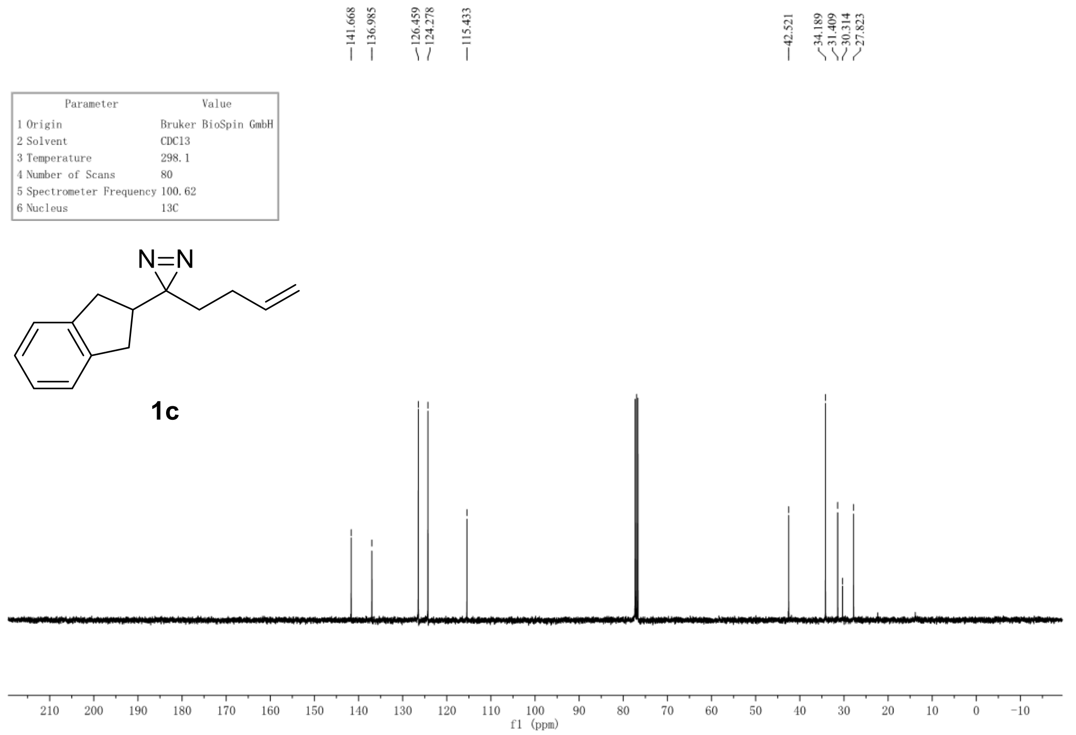
141.668 136.985 126.459 124.278 115.433 42.521 34.189 31.409 30.314 27.823
Parameter Value
1 Origin Bruker BioSpin GmbH
2 Solvent CDCl3
3 Temperature 298.1
4 Number of Scans 80
5 Spectrometer Frequency 100.62
6 Nucleus 13C
N=N
1c
210 200 190 180 170 160 150 140 130 120 110 100 90 80 70 60 50 40 30 20 10 0 -10
f1 (ppm)

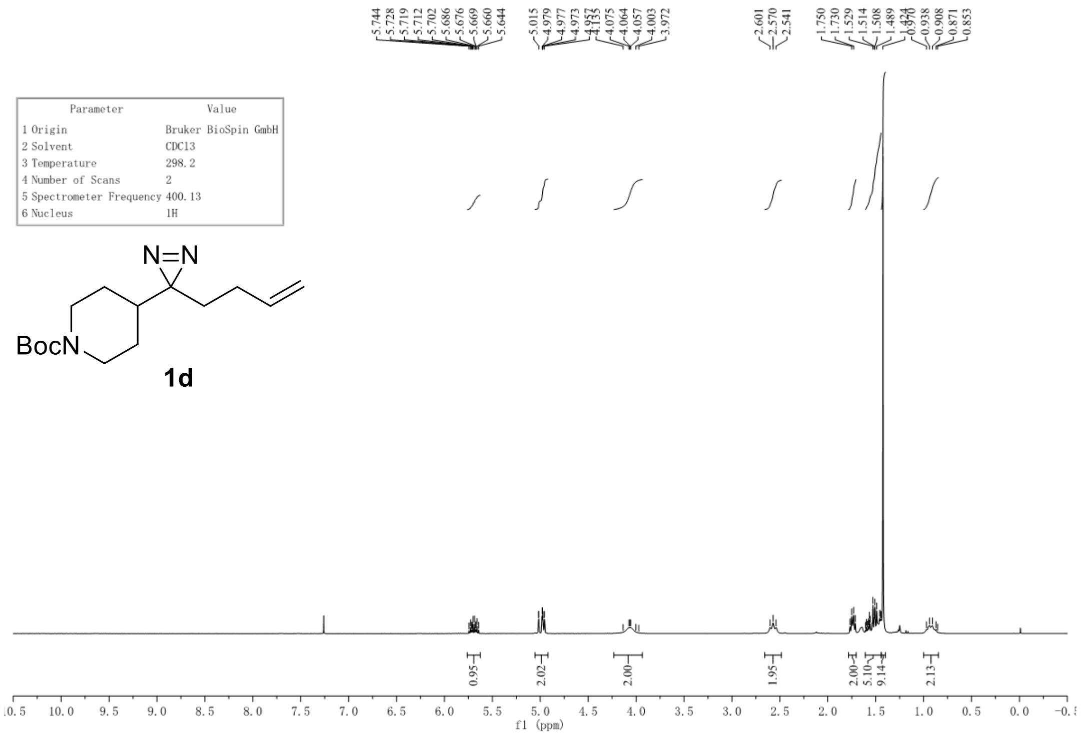
5.744 5.728 5.719 5.712 5.702 5.686 5.676 5.669 5.660 5.644
5.015 4.979 4.977 4.973
4.075 4.064 4.057 4.003 3.972
2.601 2.570 2.541
1.750 1.730 1.529 1.514 1.508 1.489
0.938 0.908 0.871 0.853
Parameter Value
1 Origin Bruker BioSpin GmbH
2 Solvent CDCl3
3 Temperature 298.2
4 Number of Scans 2
5 Spectrometer Frequency 400.13
6 Nucleus 1H
N=N
BocN
1d
0.95
2.02
2.00
1.95
2.00
5.10
9.14
2.13
f1 (ppm)

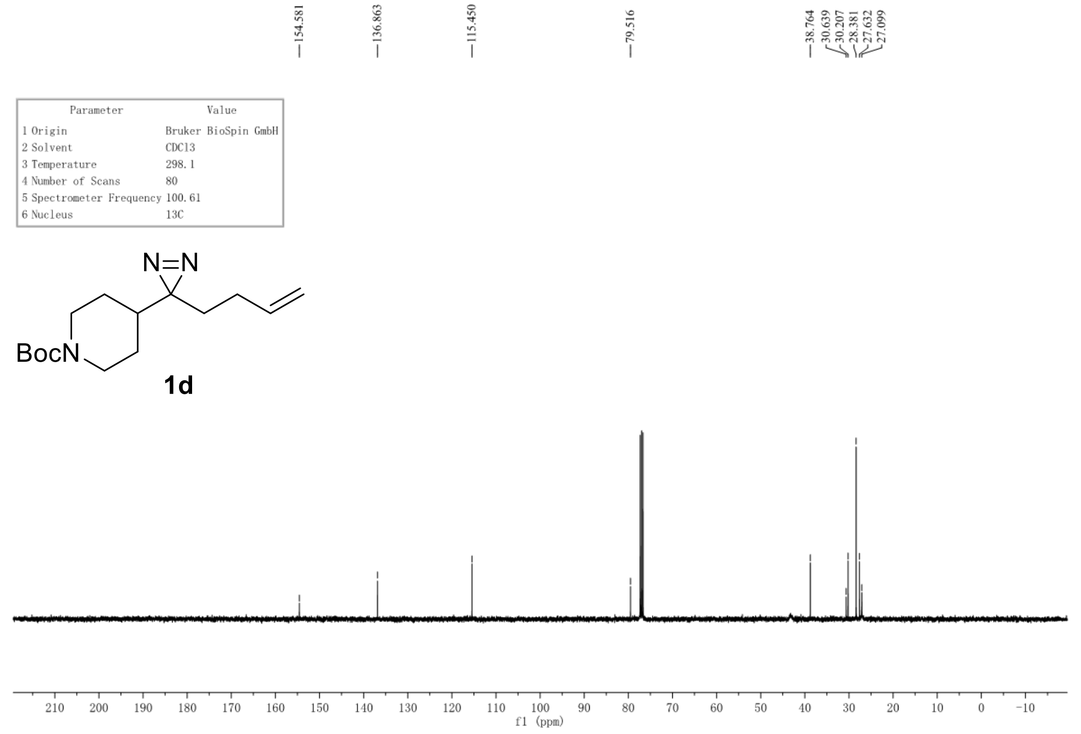
154.581
136.863
115.450
79.516
38.764 30.639 30.207 28.381 27.632 27.099
Parameter Value
1 Origin Bruker BioSpin GmbH
2 Solvent CDCl3
3 Temperature 298.1
4 Number of Scans 80
5 Spectrometer Frequency 100.61
6 Nucleus 13C
N=N
BocN
1d
f1 (ppm)

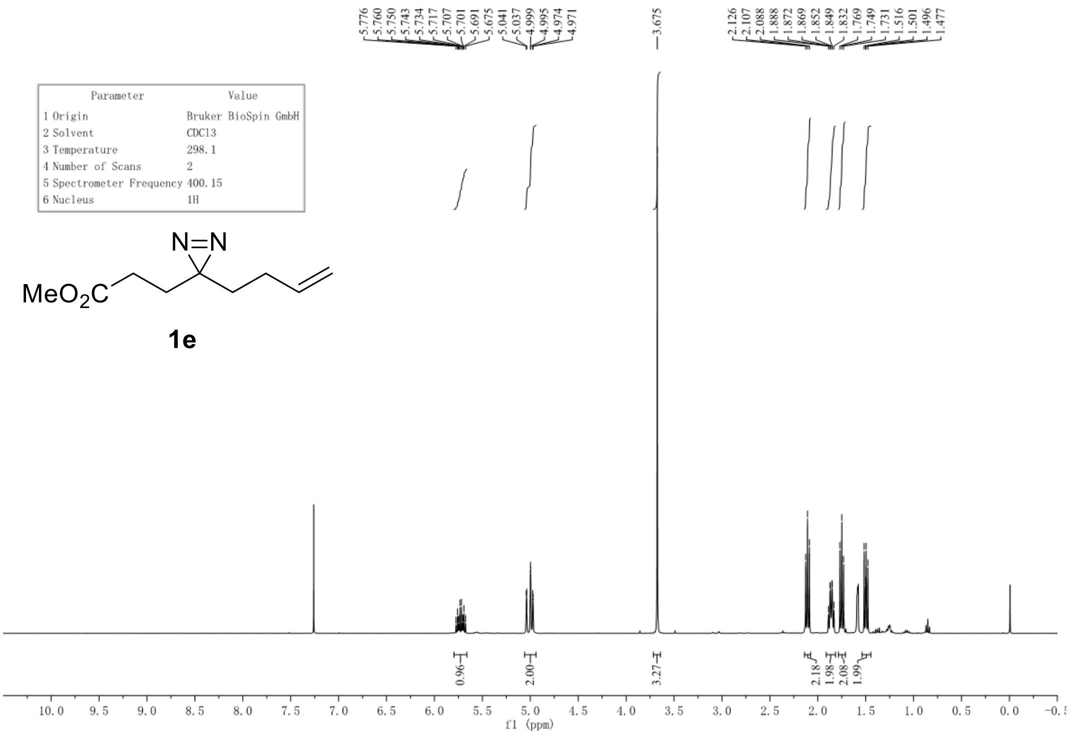
5.776 5.760 5.750 5.743 5.734 5.717 5.707 5.701 5.691 5.675 5.041 5.037 4.999 4.995 4.974 4.971
3.675
2.126 2.107 2.088 1.888 1.872 1.869 1.852 1.849 1.832 1.769 1.749 1.731 1.516 1.501 1.496 1.477
Parameter | Value
1 Origin | Bruker BioSpin GmbH
2 Solvent | CDCl3
3 Temperature | 298.1
4 Number of Scans | 2
5 Spectrometer Frequency | 400.15
6 Nucleus | 1H
N=N
MeO2C
1e
0.96 2.00 3.27 2.18 1.98 2.08 1.99
f1 (ppm)

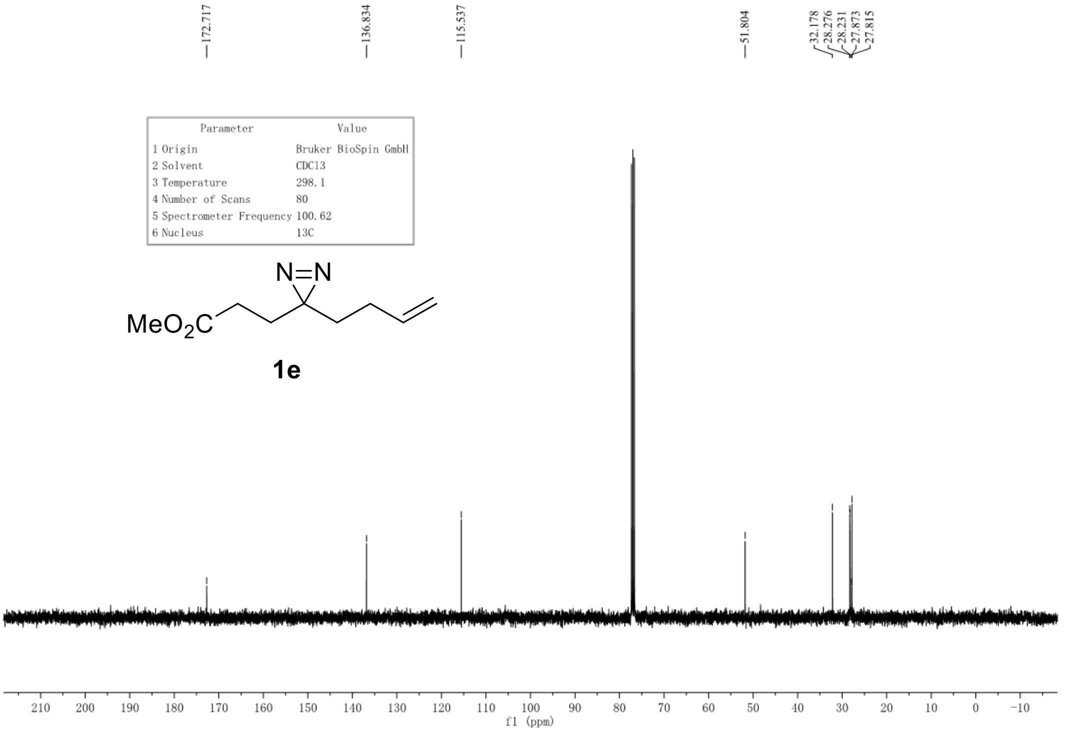
172.717
136.834
115.537
51.804
32.178 28.276 28.231 27.873 27.815
Parameter | Value
1 Origin | Bruker BioSpin GmbH
2 Solvent | CDCl3
3 Temperature | 298.1
4 Number of Scans | 80
5 Spectrometer Frequency | 100.62
6 Nucleus | 13C
N=N
MeO2C
1e
f1 (ppm)

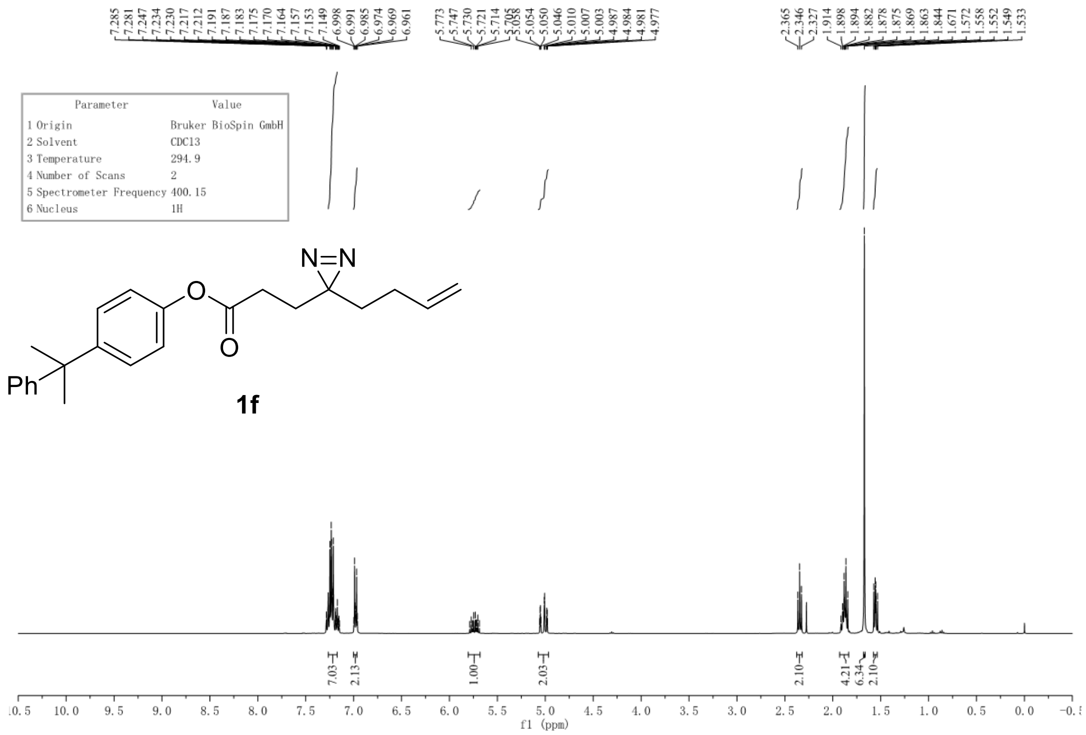

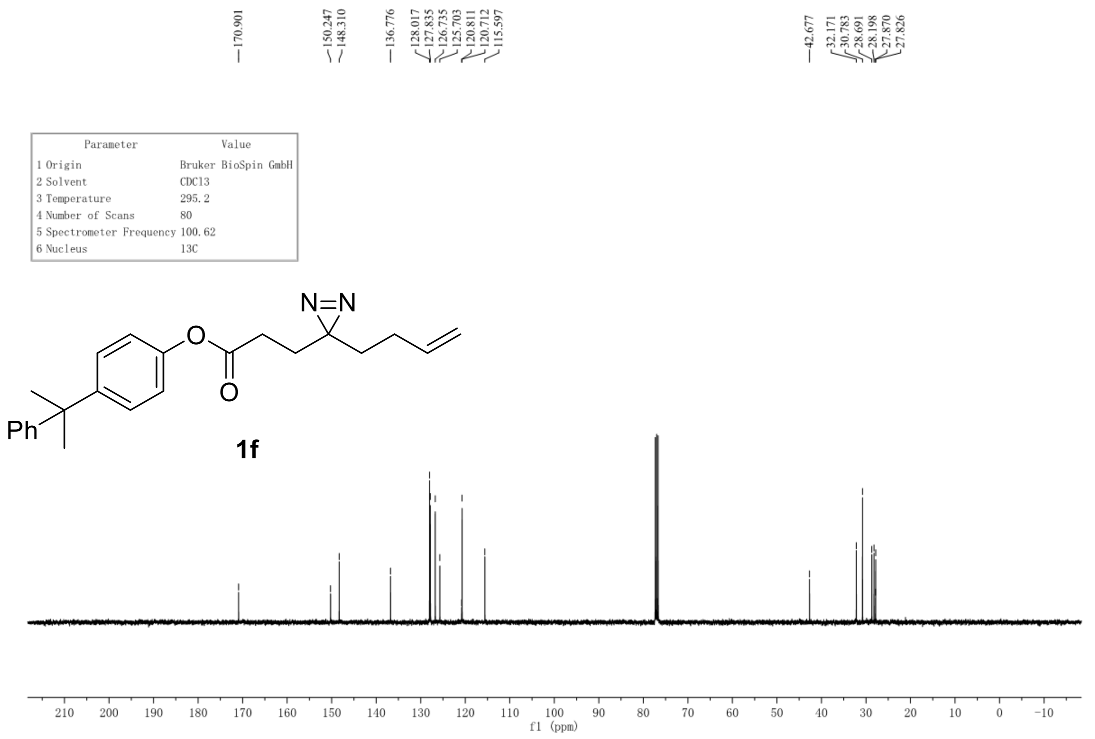

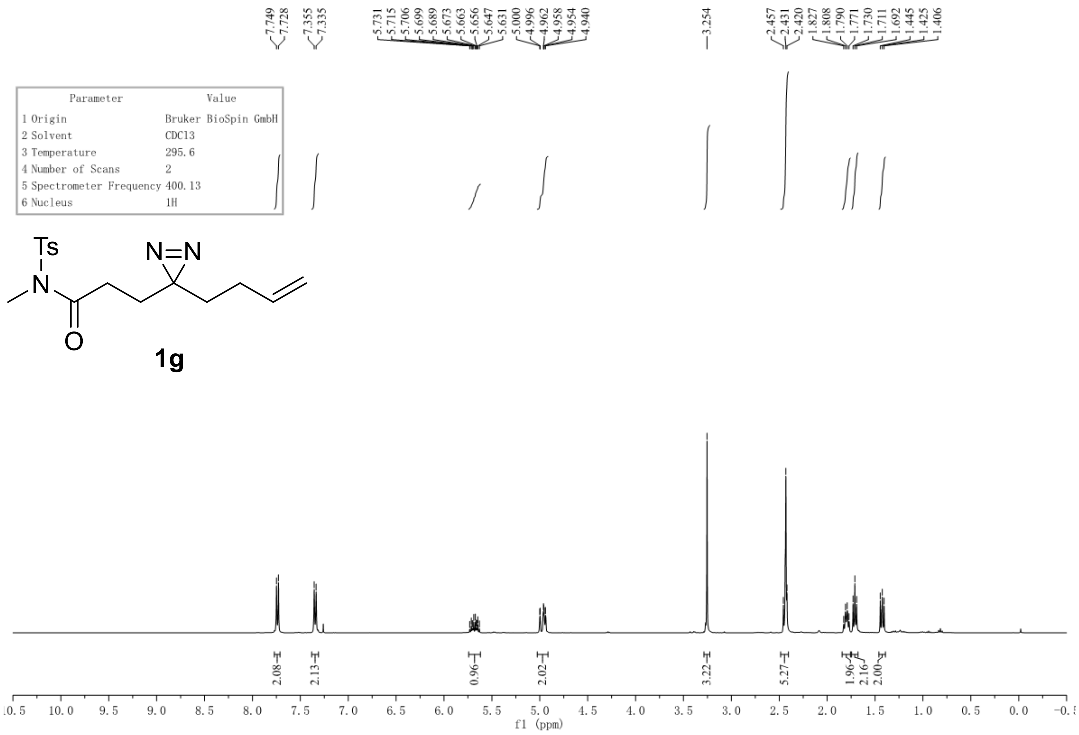
7.749
7.728
7.355
7.335
5.731
5.715
5.706
5.699
5.689
5.673
5.663
5.656
5.647
5.631
5.000
4.996
4.962
4.958
4.954
4.940
3.254
2.457
2.431
2.420
1.827
1.808
1.790
1.771
1.730
1.711
1.692
1.445
1.425
1.406
Parameter Value
1 Origin Bruker BioSpin GmbH
2 Solvent CDCl3
3 Temperature 295.6
4 Number of Scans 2
5 Spectrometer Frequency 400.13
6 Nucleus 1H
Ts
N
N=N
O
1g
2.08
2.13
0.96
2.02
3.22
5.27
1.96
2.16
2.00
f1 (ppm)

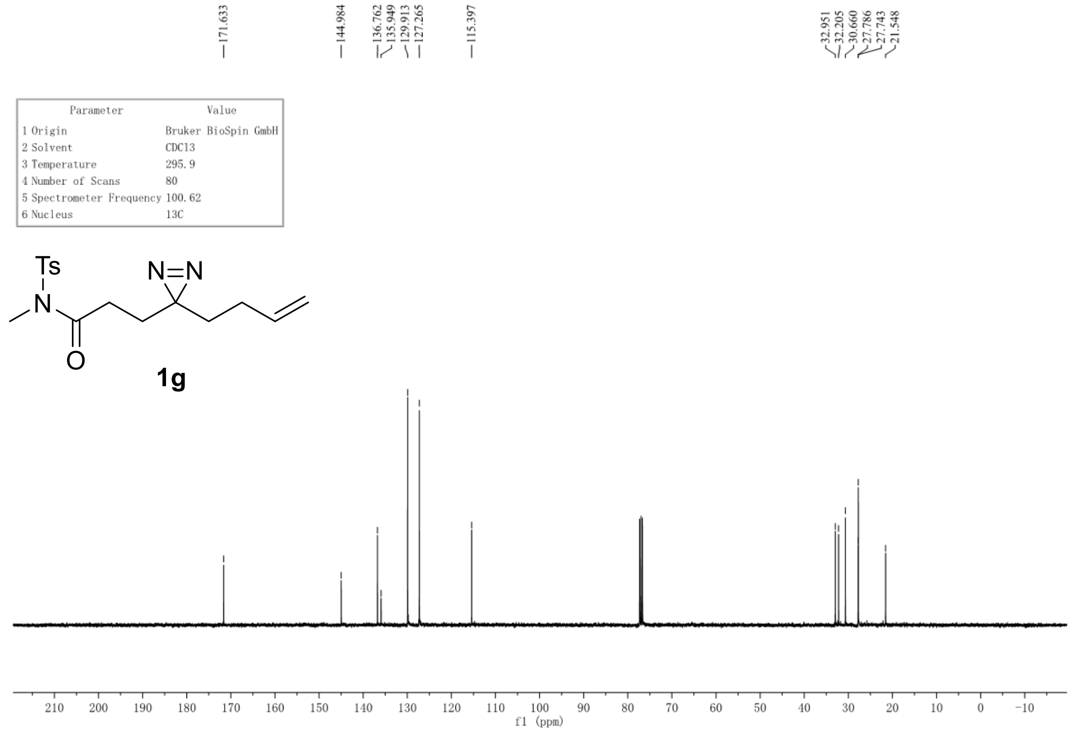
171.633
144.984
136.762
135.949
129.913
127.265
115.397
32.951
32.205
30.660
27.786
27.743
21.548
Parameter Value
1 Origin Bruker BioSpin GmbH
2 Solvent CDCl3
3 Temperature 295.9
4 Number of Scans 80
5 Spectrometer Frequency 100.62
6 Nucleus 13C
Ts
N
N=N
O
1g
f1 (ppm)

Parameter	Value
1 Origin	Bruker BioSpin GmbH
2 Solvent	CDCl3
3 Temperature	298.2
4 Number of Scans	2
5 Spectrometer Frequency	400.15
6 Nucleus	1H

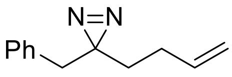

1h

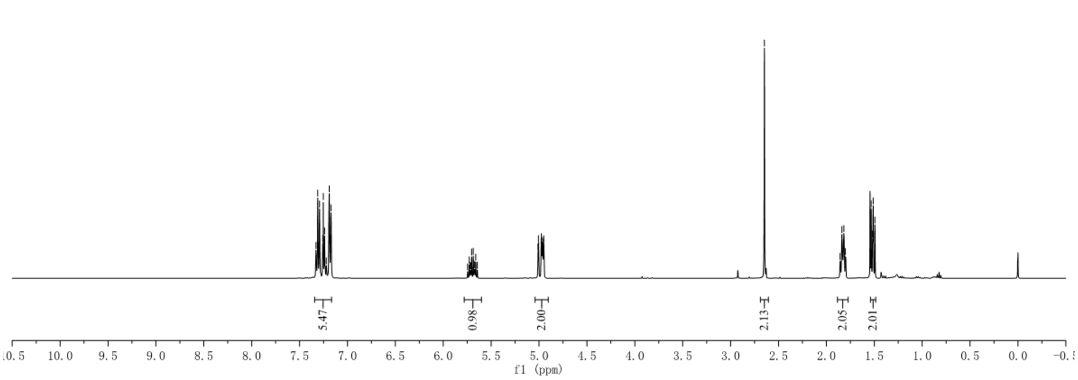

Parameter	Value
1 Origin	Bruker BioSpin GmbH
2 Solvent	CDCl3
3 Temperature	298.1
4 Number of Scans	80
5 Spectrometer Frequency	100.62
6 Nucleus	13C

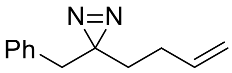

1h

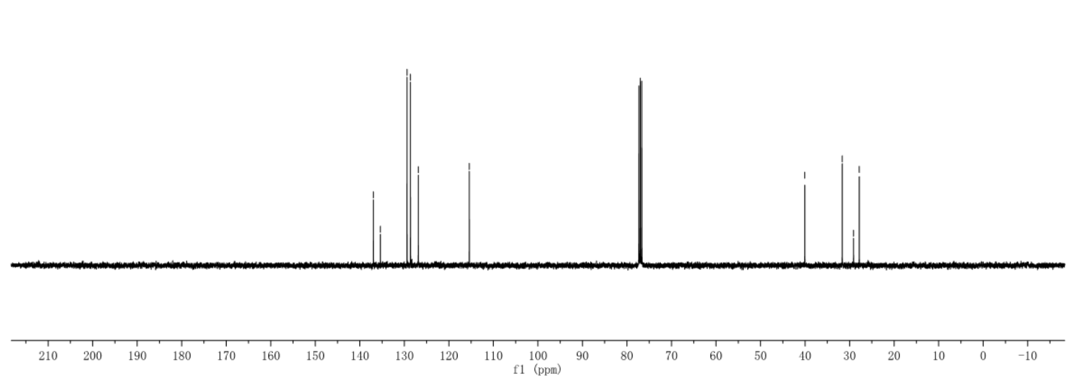

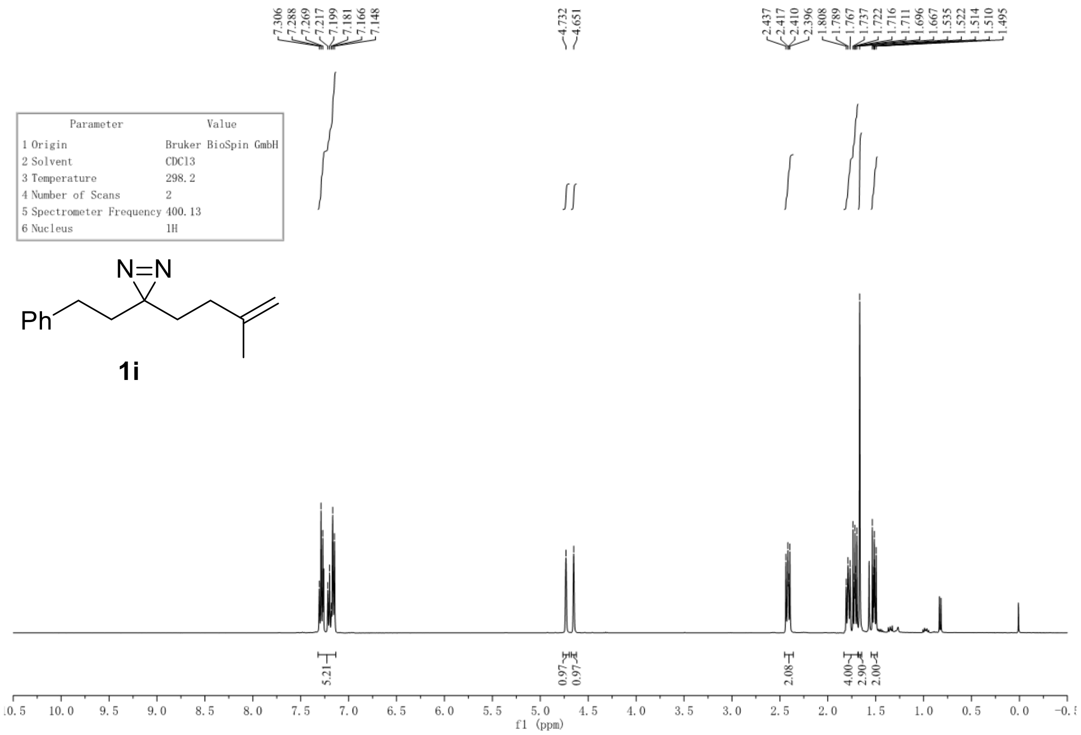
7.306
7.288
7.269
7.217
7.199
7.181
7.166
7.148
4.732
4.651
2.437
2.417
2.410
2.396
1.808
1.789
1.767
1.737
1.722
1.716
1.711
1.696
1.667
1.535
1.522
1.514
1.510
1.495
Parameter Value
1 Origin Bruker BioSpin GmbH
2 Solvent CDCl3
3 Temperature 298.2
4 Number of Scans 2
5 Spectrometer Frequency 400.13
6 Nucleus 1H
N=N
Ph
1i
5.21
0.97
0.97
2.08
4.00
2.90
2.00
f1 (ppm)

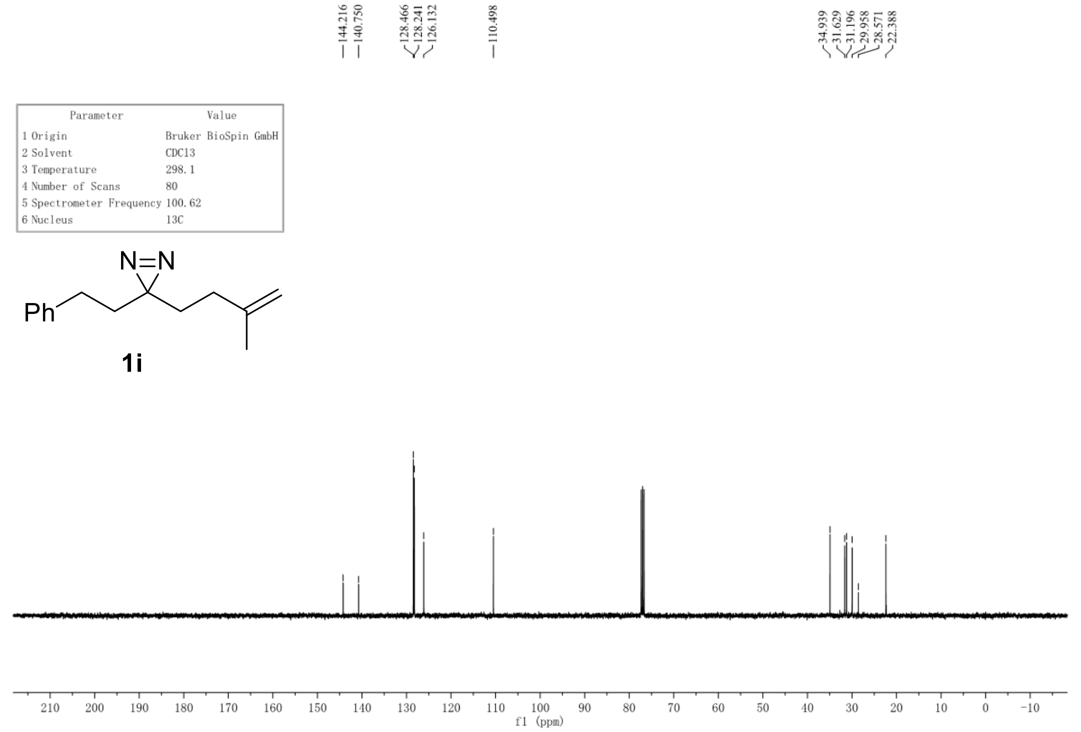
144.216
140.750
128.466
128.241
126.132
110.498
34.939
31.629
31.196
29.958
28.571
22.388
Parameter Value
1 Origin Bruker BioSpin GmbH
2 Solvent CDCl3
3 Temperature 298.1
4 Number of Scans 80
5 Spectrometer Frequency 100.62
6 Nucleus 13C
N=N
Ph
1i
f1 (ppm)

Parameter	Value
1 Origin	Bruker BioSpin GmbH
2 Solvent	CDCl3
3 Temperature	298.2
4 Number of Scans	2
5 Spectrometer Frequency	400.15
6 Nucleus	1H

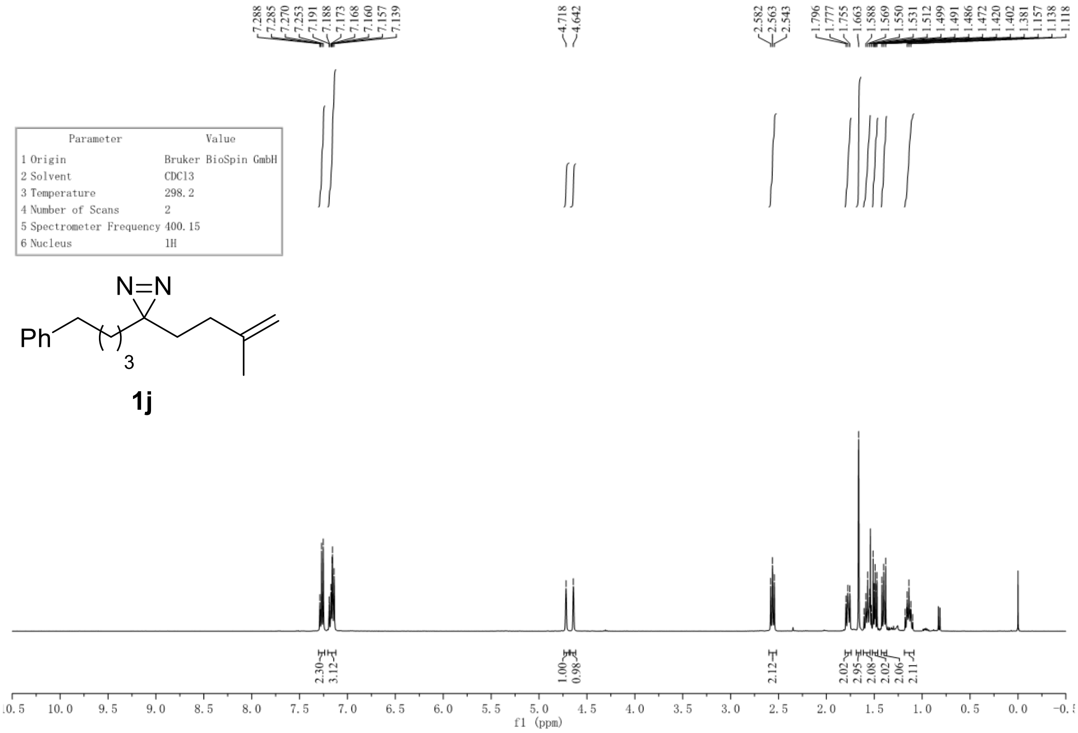

Parameter	Value
1 Origin	Bruker BioSpin GmbH
2 Solvent	CDCl3
3 Temperature	298.2
4 Number of Scans	80
5 Spectrometer Frequency	100.62
6 Nucleus	13C

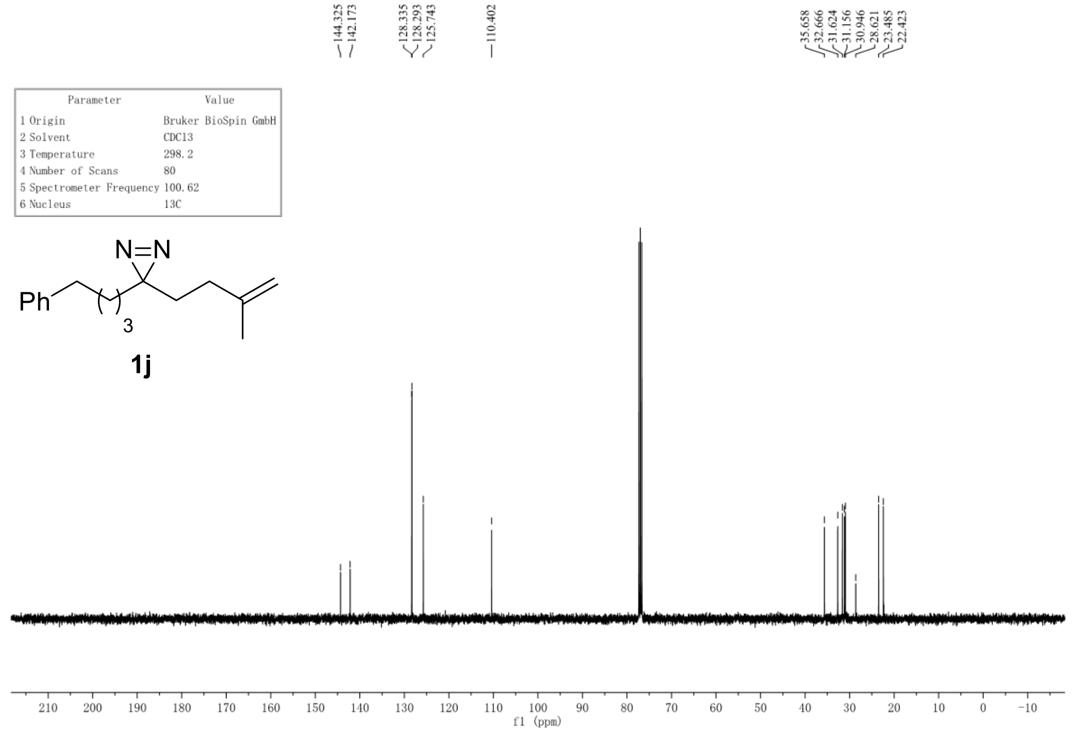

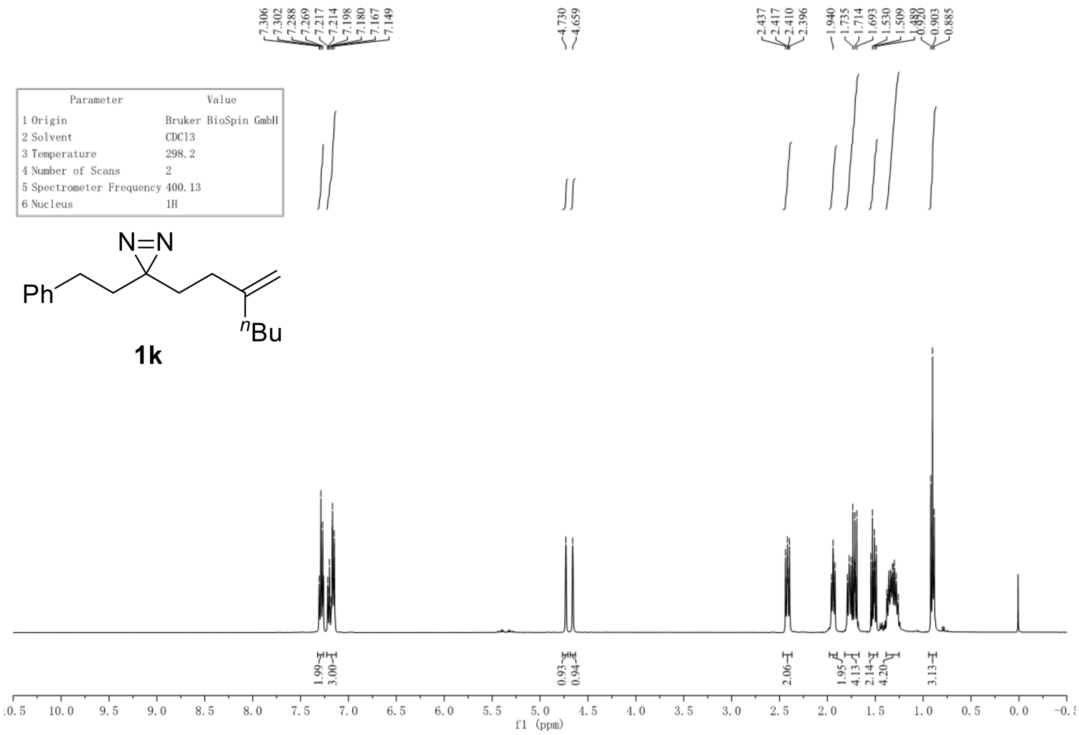

Parameter Value
1 Origin Bruker BioSpin GmbH
2 Solvent CDCl3
3 Temperature 298.2
4 Number of Scans 2
5 Spectrometer Frequency 400.13
6 Nucleus 1H
N=N
Ph
nBu
1k
f1 (ppm)

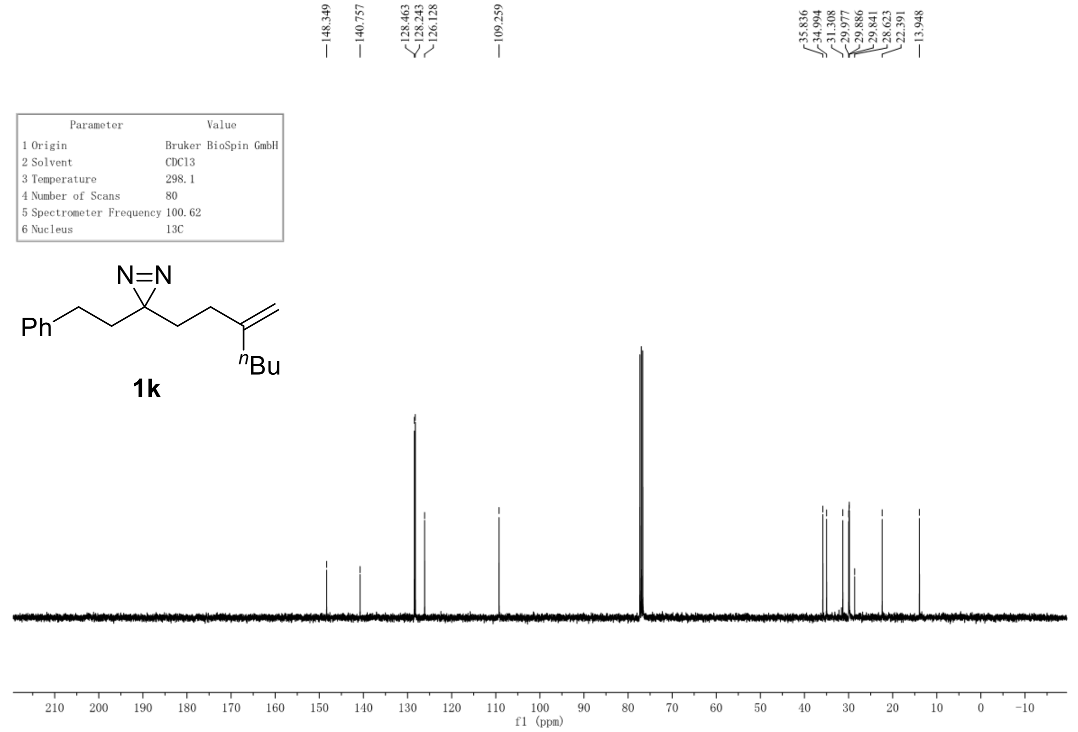

Parameter Value
1 Origin Bruker BioSpin GmbH
2 Solvent CDCl3
3 Temperature 298.1
4 Number of Scans 80
5 Spectrometer Frequency 100.62
6 Nucleus 13C
N=N
Ph
nBu
1k
f1 (ppm)

Parameter	Value
1 Origin	Bruker BioSpin GmbH
2 Solvent	CDCl3
3 Temperature	298.1
4 Number of Scans	2
5 Spectrometer Frequency	400.13
6 Nucleus	1H

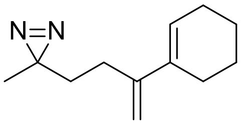

1l

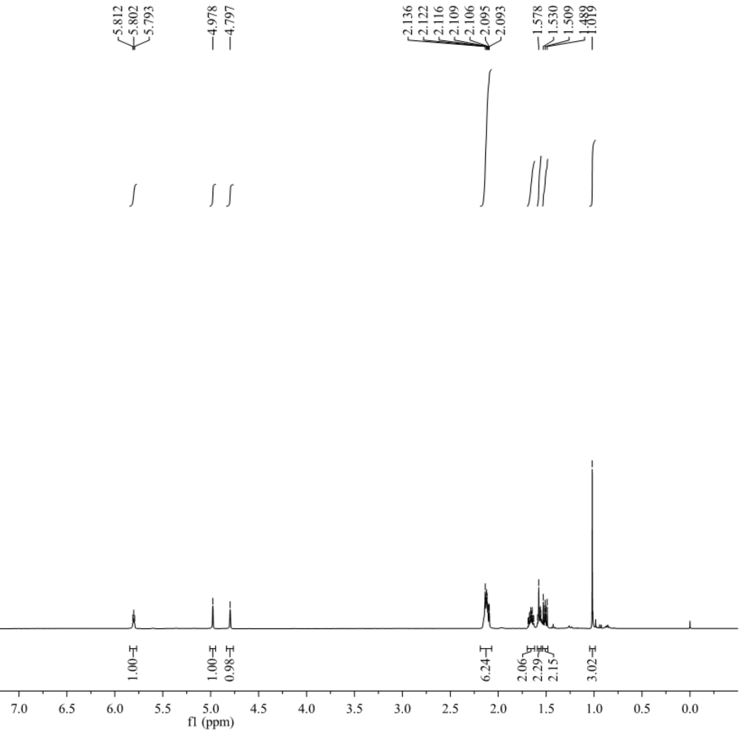

Parameter	Value
1 Origin	Bruker BioSpin GmbH
2 Solvent	CDCl3
3 Temperature	298.1
4 Number of Scans	80
5 Spectrometer Frequency	100.62
6 Nucleus	13C

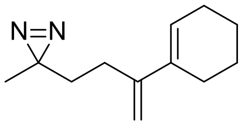

1l

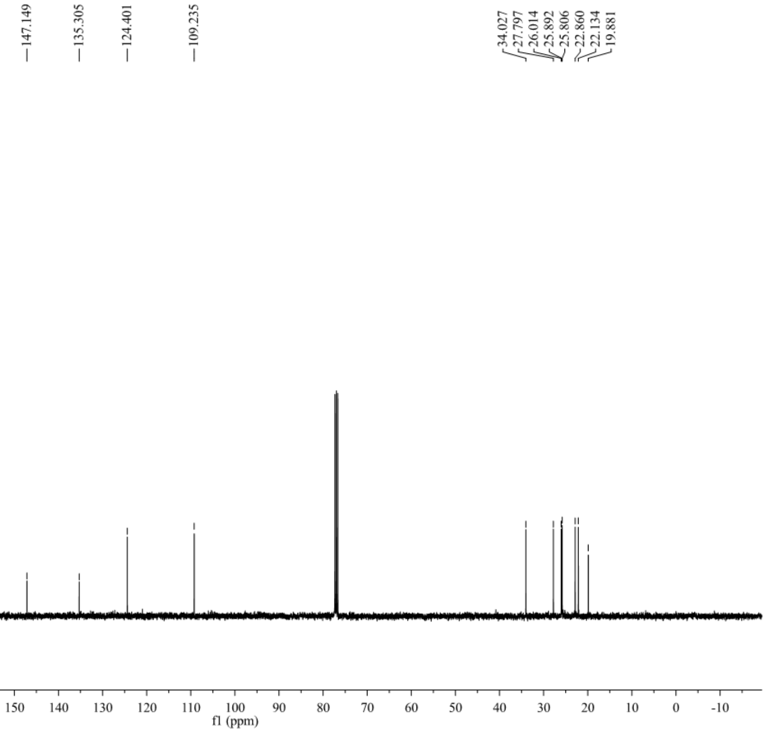

Parameter	Value
1 Origin	Bruker BioSpin GmbH
2 Solvent	CDCl3
3 Temperature	298.2
4 Number of Scans	2
5 Spectrometer Frequency	400.13
6 Nucleus	1H

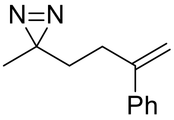

1m

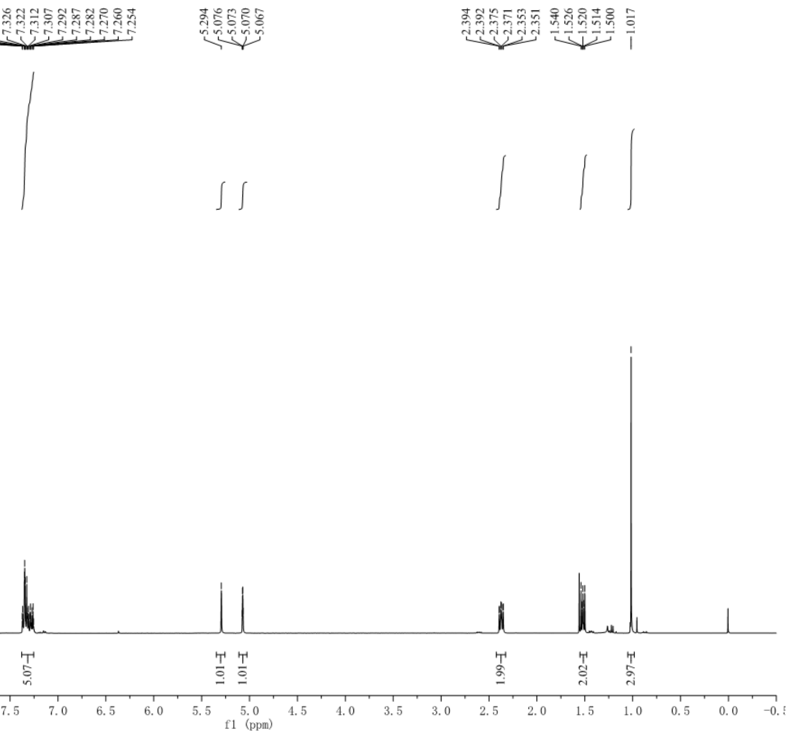

Parameter	Value
1 Origin	Bruker BioSpin GmbH
2 Solvent	CDCl3
3 Temperature	298.1
4 Number of Scans	80
5 Spectrometer Frequency	100.62
6 Nucleus	13C

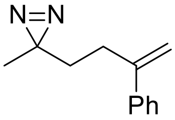

1m

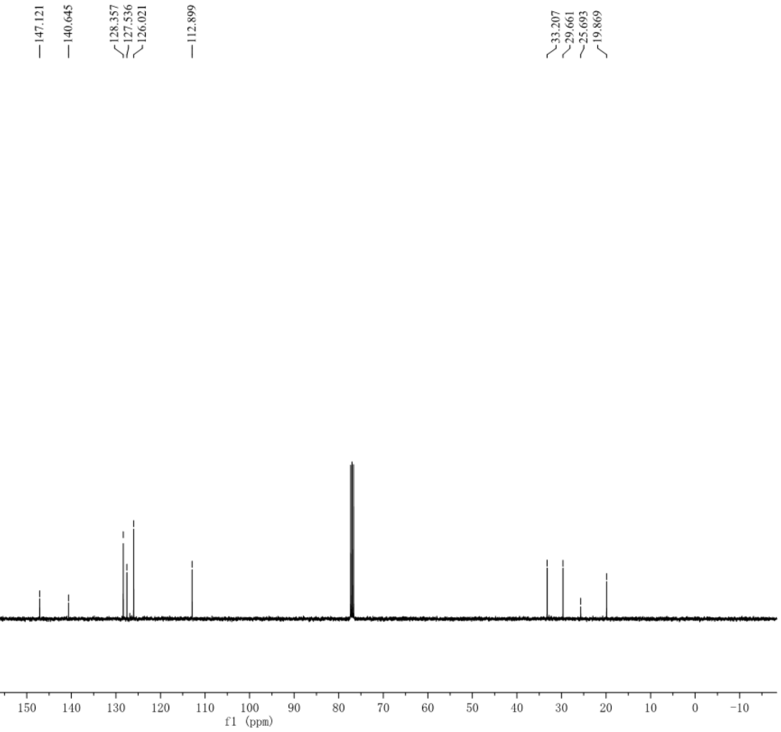

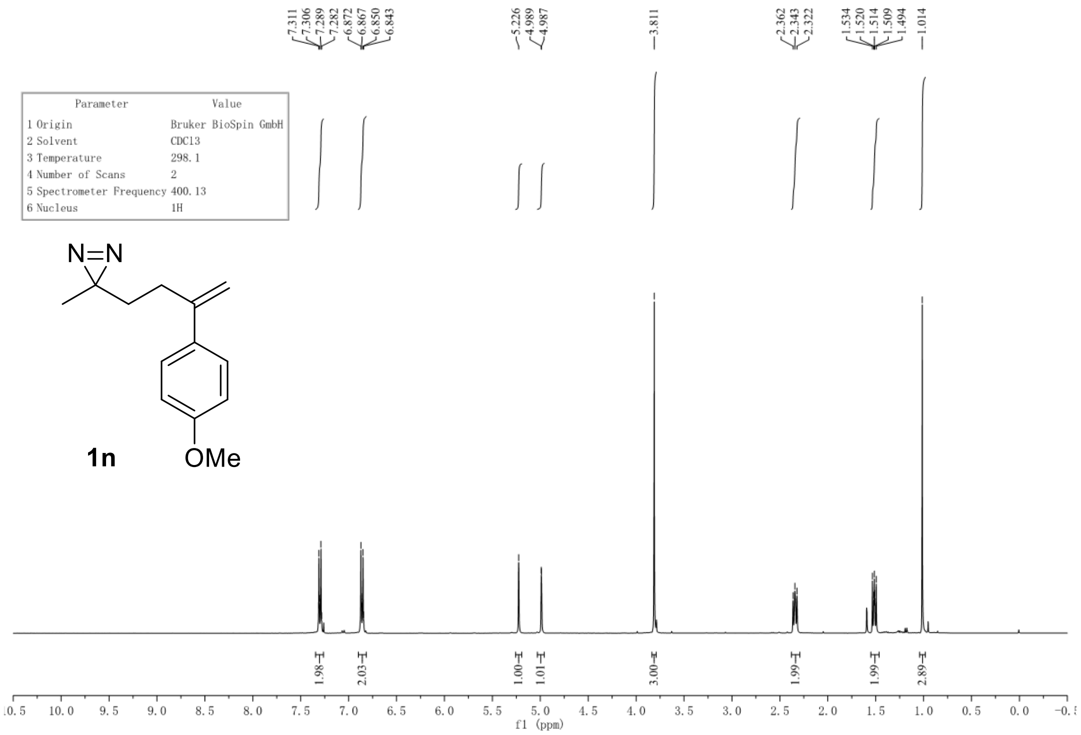
7.311
7.306
7.289
7.282
6.872
6.867
6.850
6.843
5.226
4.989
4.987
3.811
2.362
2.343
2.322
1.534
1.520
1.514
1.509
1.494
1.014
Parameter Value
1 Origin Bruker BioSpin GmbH
2 Solvent CDCl3
3 Temperature 298.1
4 Number of Scans 2
5 Spectrometer Frequency 400.13
6 Nucleus 1H
N=N
OMe
1n
1.98
2.03
1.00
1.01
3.00
1.99
1.99
2.89
f1 (ppm)

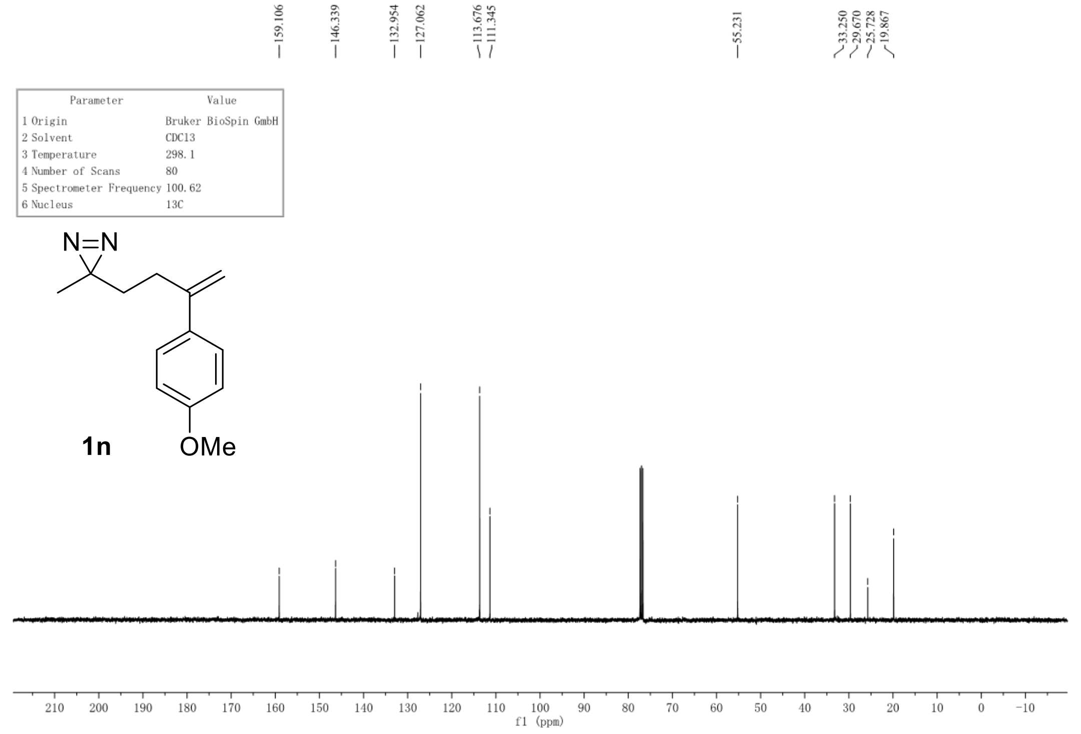
159.106
146.339
132.954
127.062
113.676
111.345
55.231
33.250
29.670
25.728
19.867
Parameter Value
1 Origin Bruker BioSpin GmbH
2 Solvent CDCl3
3 Temperature 298.1
4 Number of Scans 80
5 Spectrometer Frequency 100.62
6 Nucleus 13C
N=N
OMe
1n
f1 (ppm)

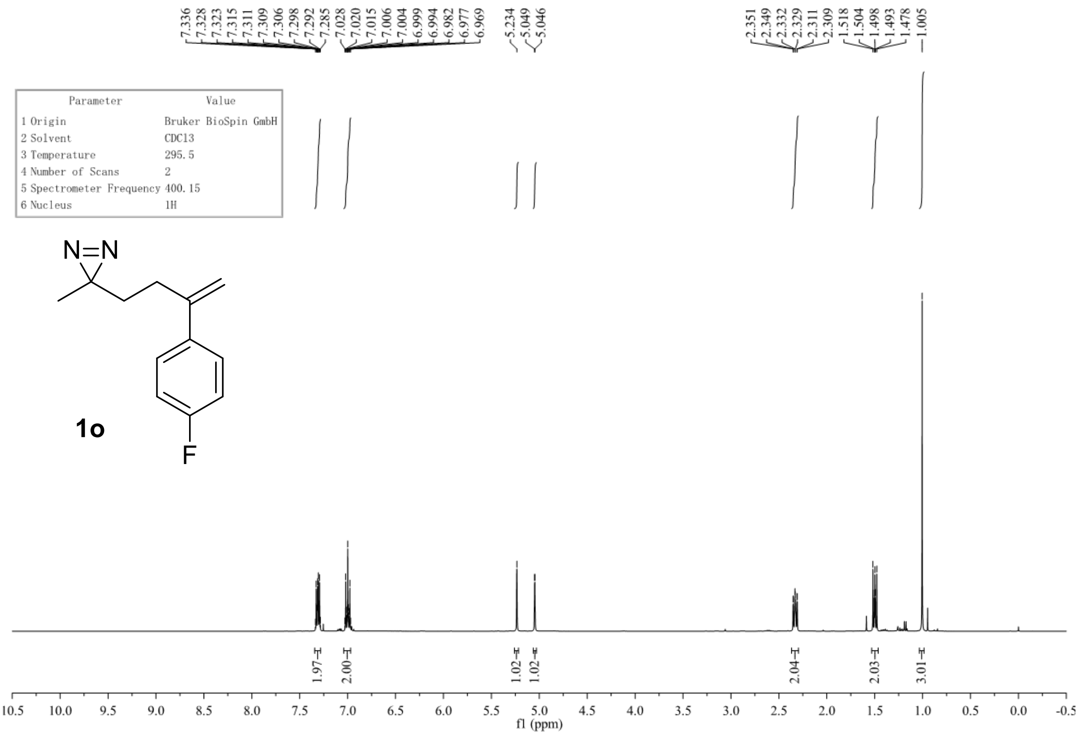

Parameter	Value
1 Origin	Bruker BioSpin GmbH
2 Solvent	CDCl3
3 Temperature	295.5
4 Number of Scans	2
5 Spectrometer Frequency	400.15
6 Nucleus	1H

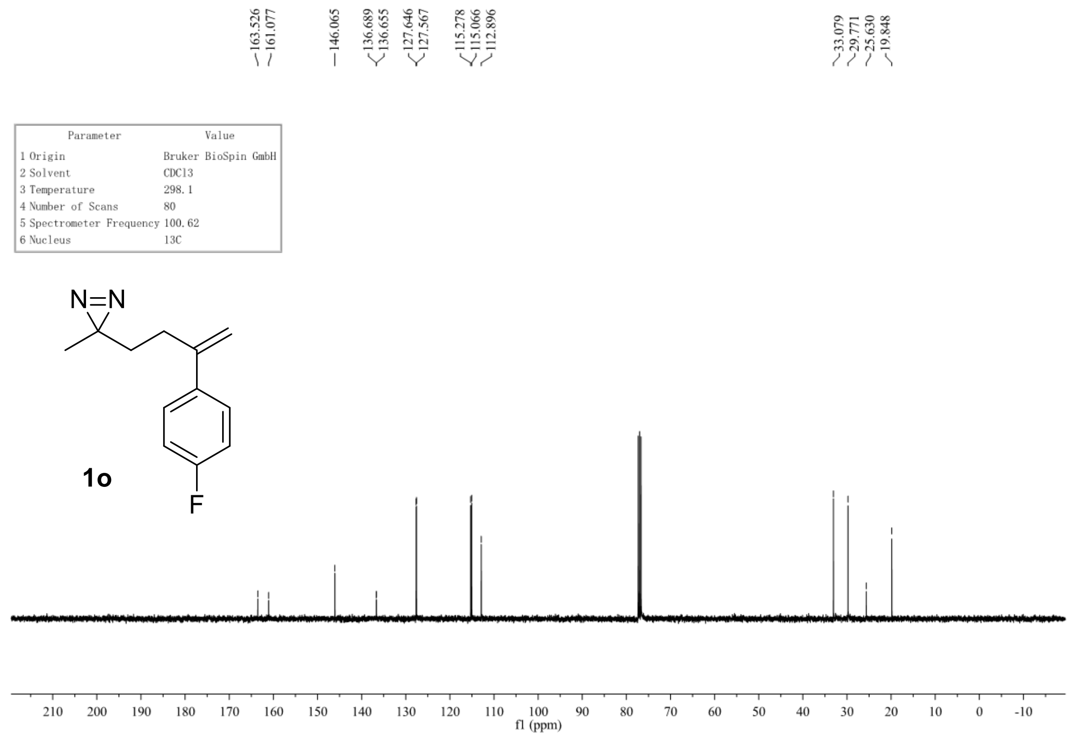

Parameter	Value
1 Origin	Bruker BioSpin GmbH
2 Solvent	CDCl3
3 Temperature	298.1
4 Number of Scans	80
5 Spectrometer Frequency	100.62
6 Nucleus	13C

Parameter	Value
1 Origin	Bruker BioSpin GmbH
2 Solvent	CDCl3
3 Temperature	295.5
4 Number of Scans	2
5 Spectrometer Frequency	376.52
6 Nucleus	19F

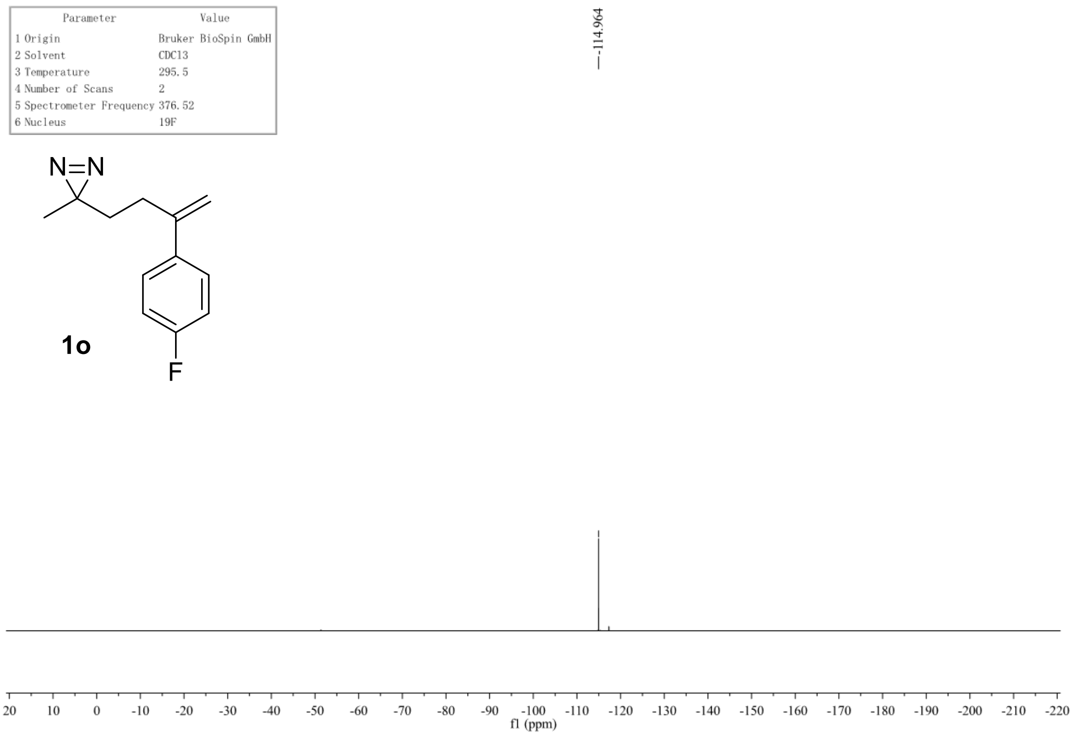

Parameter	Value
1 Origin	Bruker BioSpin GmbH
2 Solvent	CDCl3
3 Temperature	298.2
4 Number of Scans	2
5 Spectrometer Frequency	400.13
6 Nucleus	1H

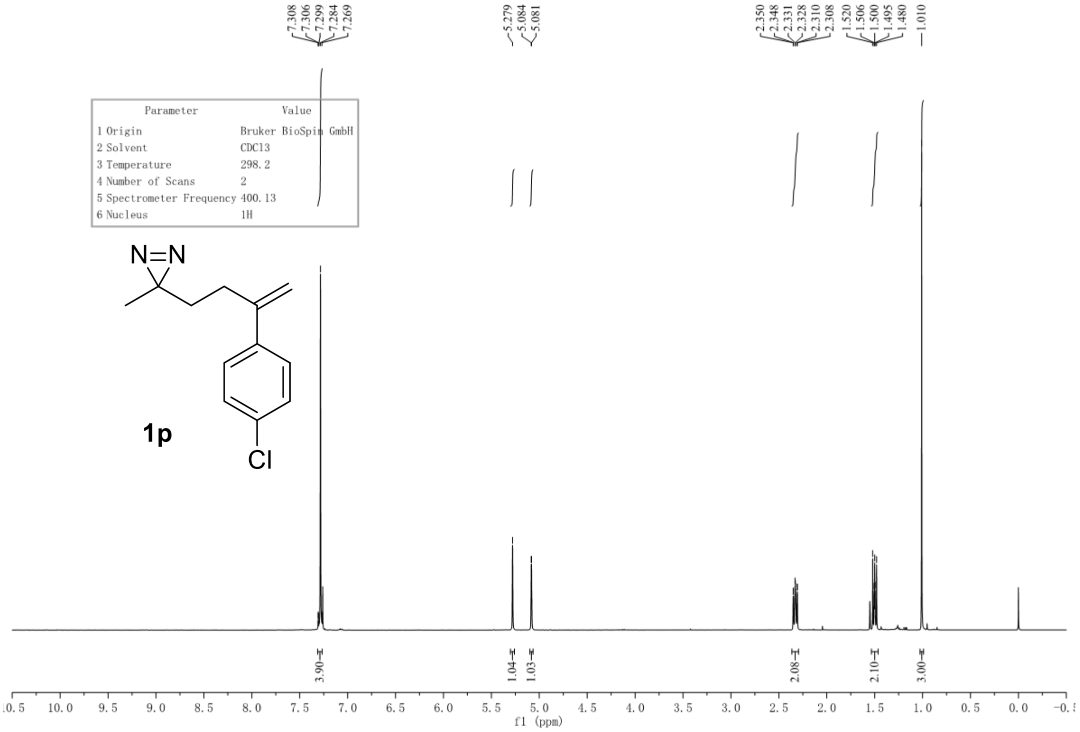

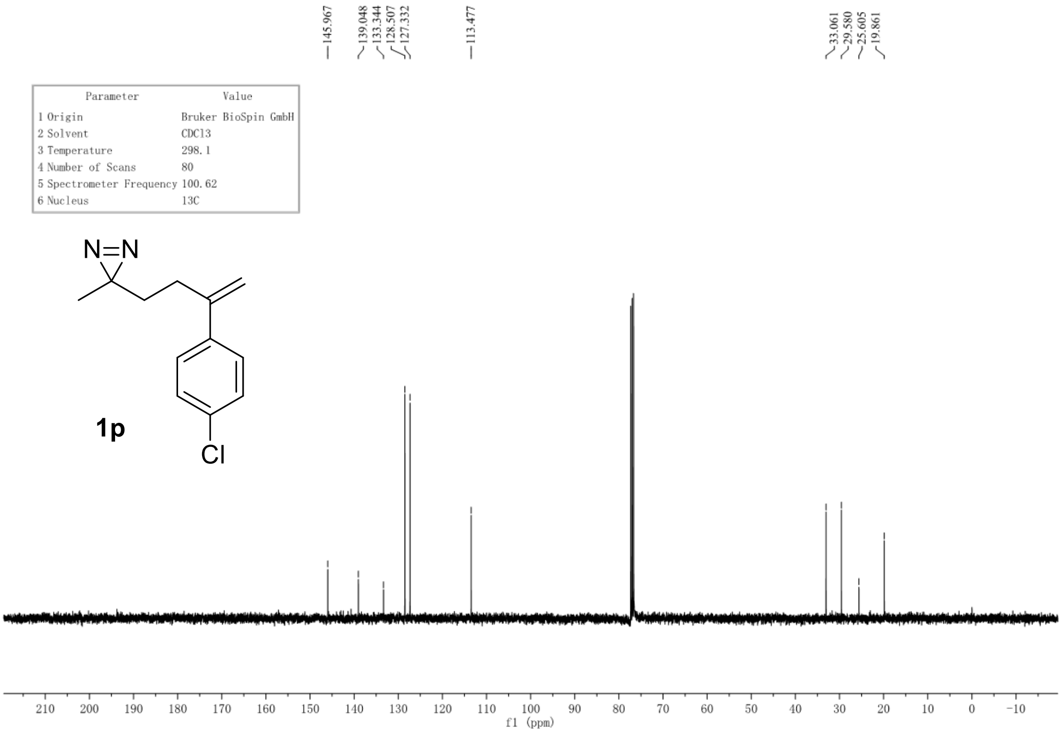

Parameter	Value
1 Origin	Bruker BioSpin GmbH
2 Solvent	CDCl3
3 Temperature	298.1
4 Number of Scans	80
5 Spectrometer Frequency	100.62
6 Nucleus	13C

1p

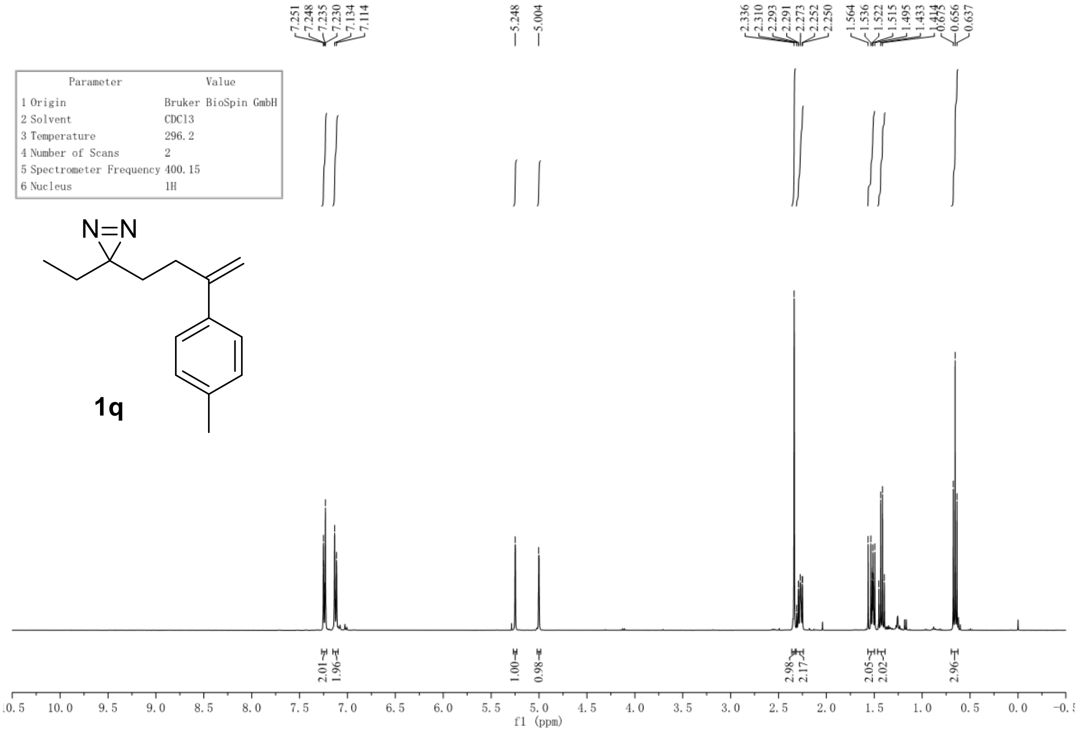

Parameter	Value
1 Origin	Bruker BioSpin GmbH
2 Solvent	CDCl3
3 Temperature	296.2
4 Number of Scans	2
5 Spectrometer Frequency	400.15
6 Nucleus	1H

1q

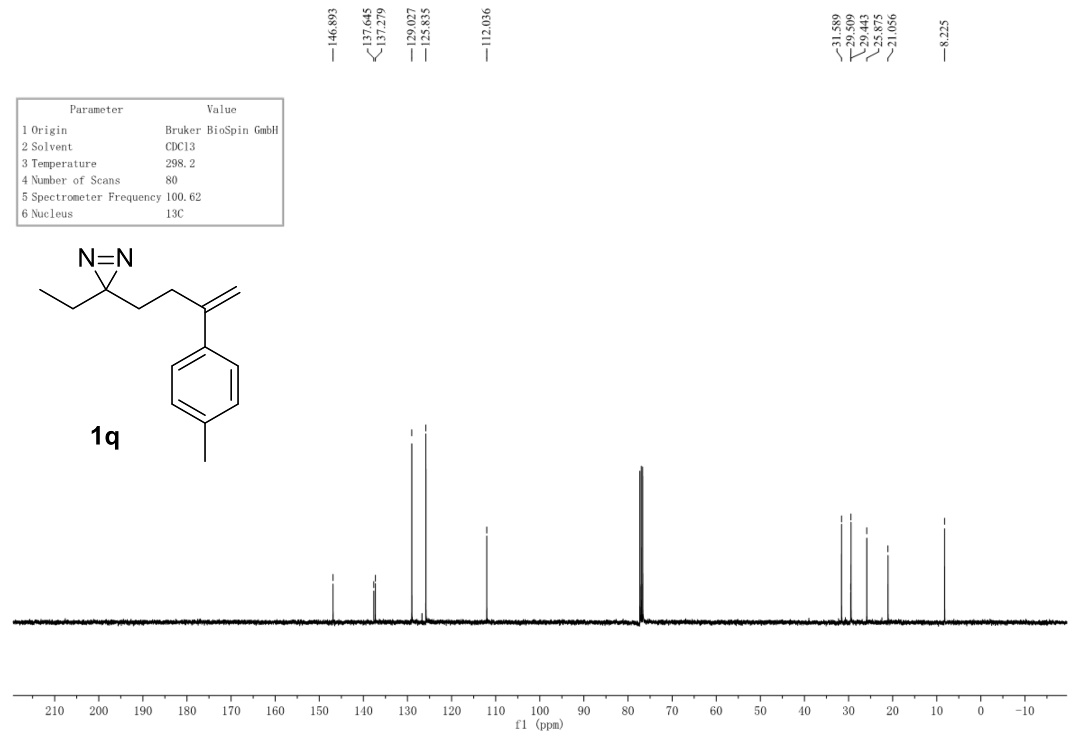
146.893
137.645
137.279
129.027
125.835
112.036
31.589
29.509
29.443
25.875
21.056
8.225
Parameter Value
1 Origin Bruker BioSpin GmbH
2 Solvent CDCl3
3 Temperature 298.2
4 Number of Scans 80
5 Spectrometer Frequency 100.62
6 Nucleus 13C
N=N
1q
f1 (ppm)

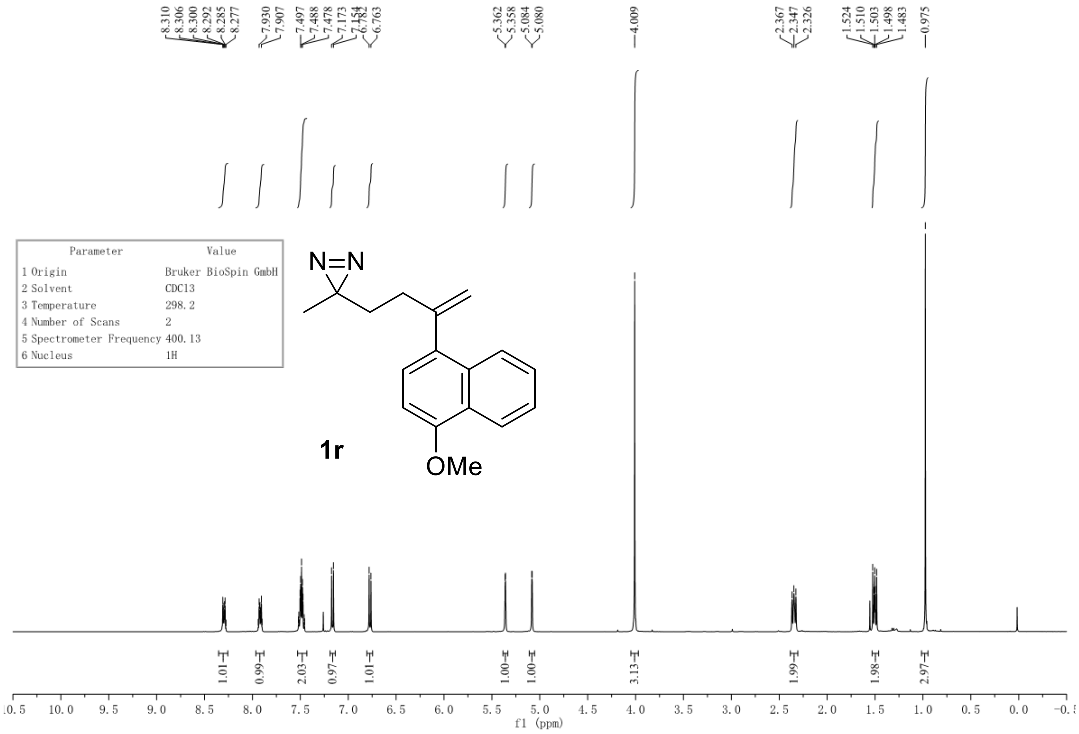
8.310
8.306
8.300
8.292
8.285
8.277
7.930
7.907
7.497
7.488
7.478
7.173
7.154
6.782
6.763
5.362
5.358
5.084
5.080
4.009
2.367
2.347
2.326
1.524
1.510
1.503
1.498
1.483
0.975
Parameter Value
1 Origin Bruker BioSpin GmbH
2 Solvent CDCl3
3 Temperature 298.2
4 Number of Scans 2
5 Spectrometer Frequency 400.13
6 Nucleus 1H
N=N
1r
OMe
1.01
0.99
2.03
0.97
1.01
1.00
1.00
3.13
1.99
1.98
2.97
f1 (ppm)

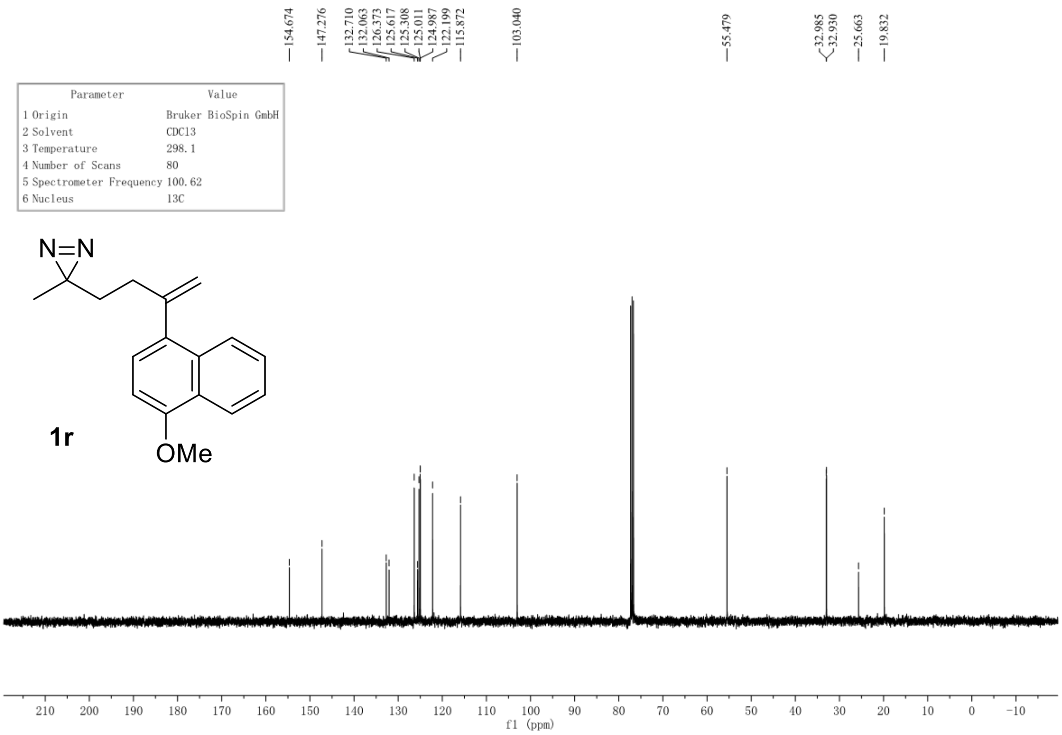
154.674
147.276
132.710
132.063
126.373
125.617
125.308
125.011
124.987
122.199
115.872
103.040
55.479
32.985
32.930
25.663
19.832
Parameter Value
1 Origin Bruker BioSpin GmbH
2 Solvent CDCl3
3 Temperature 298.1
4 Number of Scans 80
5 Spectrometer Frequency 100.62
6 Nucleus 13C
N=N
OMe
1r
210 200 190 180 170 160 150 140 130 120 110 100 90 80 70 60 50 40 30 20 10 0 −10
f1 (ppm)

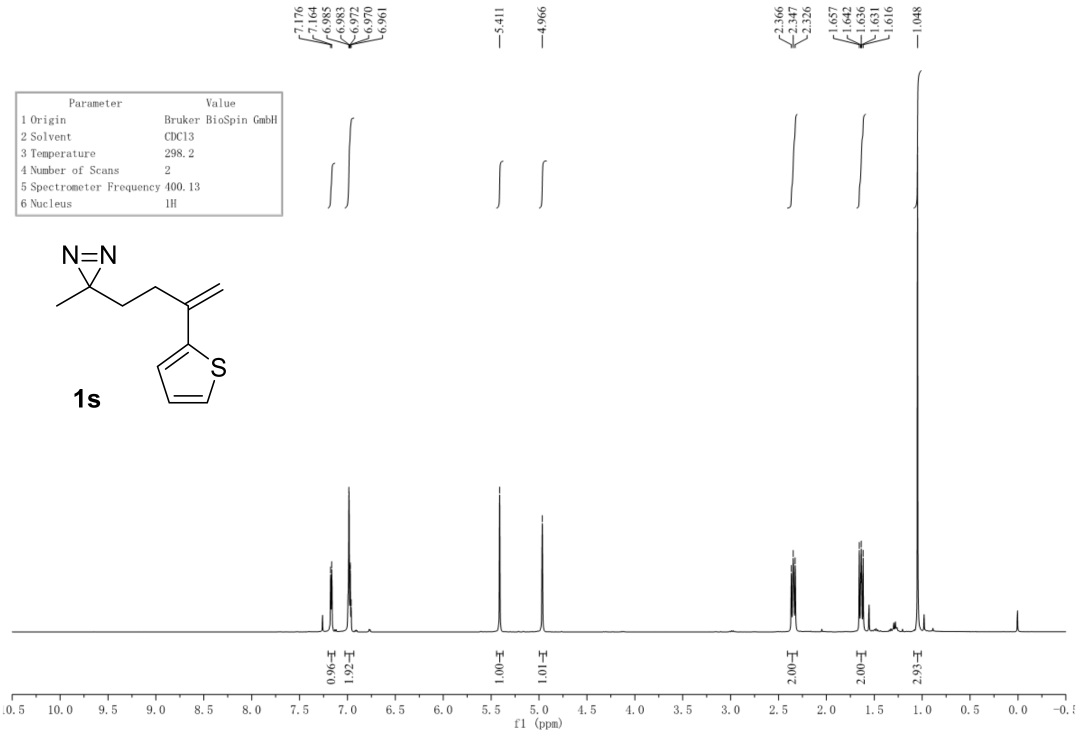
7.176
7.164
6.985
6.983
6.972
6.970
6.961
5.411
4.966
2.366
2.347
2.326
1.657
1.642
1.636
1.631
1.616
1.048
Parameter Value
1 Origin Bruker BioSpin GmbH
2 Solvent CDCl3
3 Temperature 298.2
4 Number of Scans 2
5 Spectrometer Frequency 400.13
6 Nucleus 1H
N=N
S
1s
0.96
1.92
1.00
1.01
2.00
2.00
2.93
10.5 10.0 9.5 9.0 8.5 8.0 7.5 7.0 6.5 6.0 5.5 5.0 4.5 4.0 3.5 3.0 2.5 2.0 1.5 1.0 0.5 0.0 −0.5
f1 (ppm)

Parameter	Value
1 Origin	Bruker BioSpin GmbH
2 Solvent	CDCl3
3 Temperature	298.1
4 Number of Scans	80
5 Spectrometer Frequency	100.61
6 Nucleus	13C

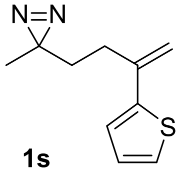

1s

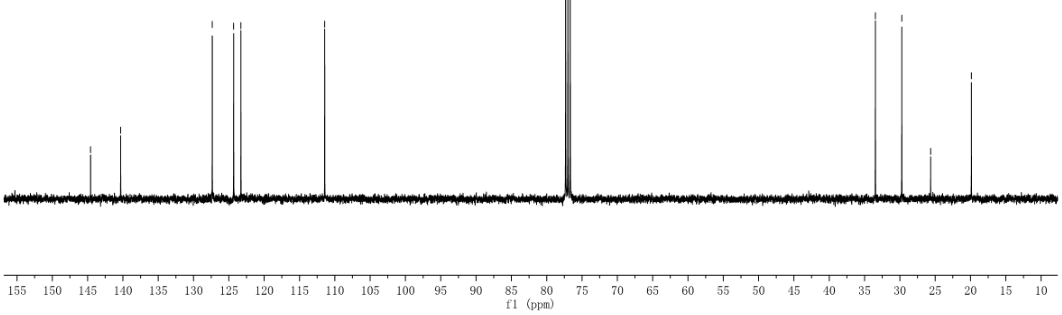

Parameter	Value
1 Origin	Bruker BioSpin GmbH
2 Solvent	CDCl3
3 Temperature	298.1
4 Number of Scans	2
5 Spectrometer Frequency	400.13
6 Nucleus	1H

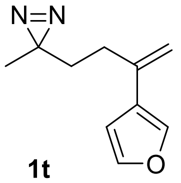

1t

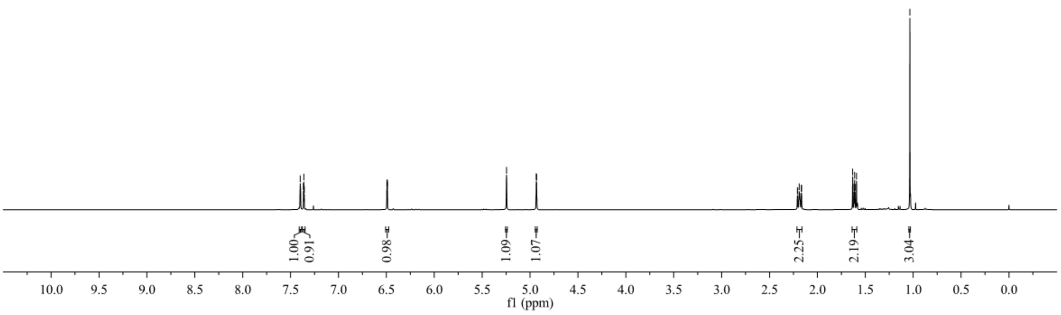

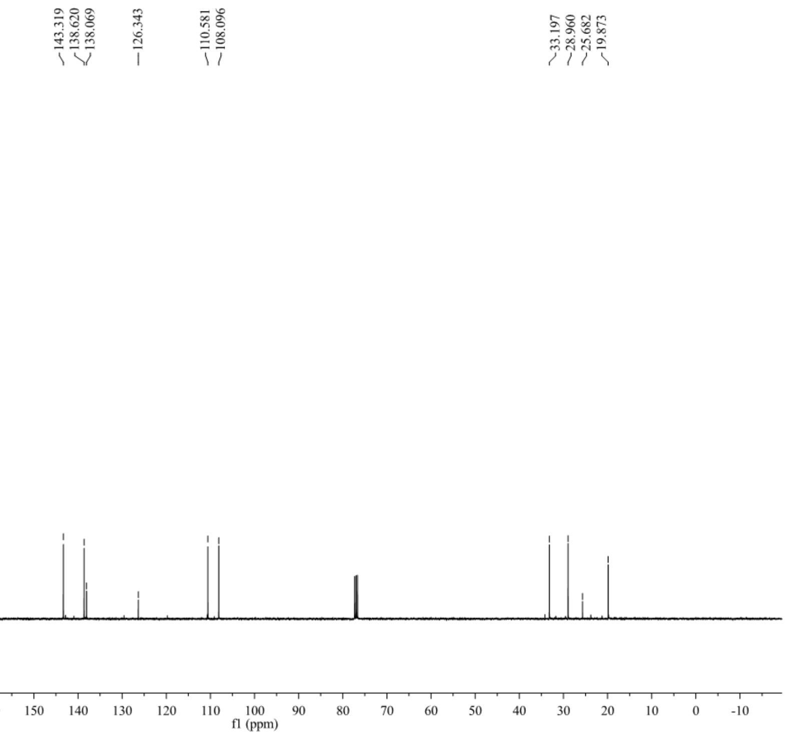
Parameter | Value
1 Origin | Bruker BioSpin GmbH
2 Solvent | CDCl3
3 Temperature | 298.1
4 Number of Scans | 80
5 Spectrometer Frequency | 100.62
6 Nucleus | 13C
143.319
138.620
138.069
126.343
110.581
108.096
33.197
28.960
25.682
19.873
210 200 190 180 170 160 150 140 130 120 110 100 90 80 70 60 50 40 30 20 10 0 -10
f1 (ppm)

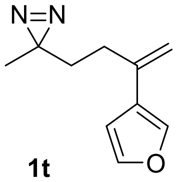
N=N
O

1t

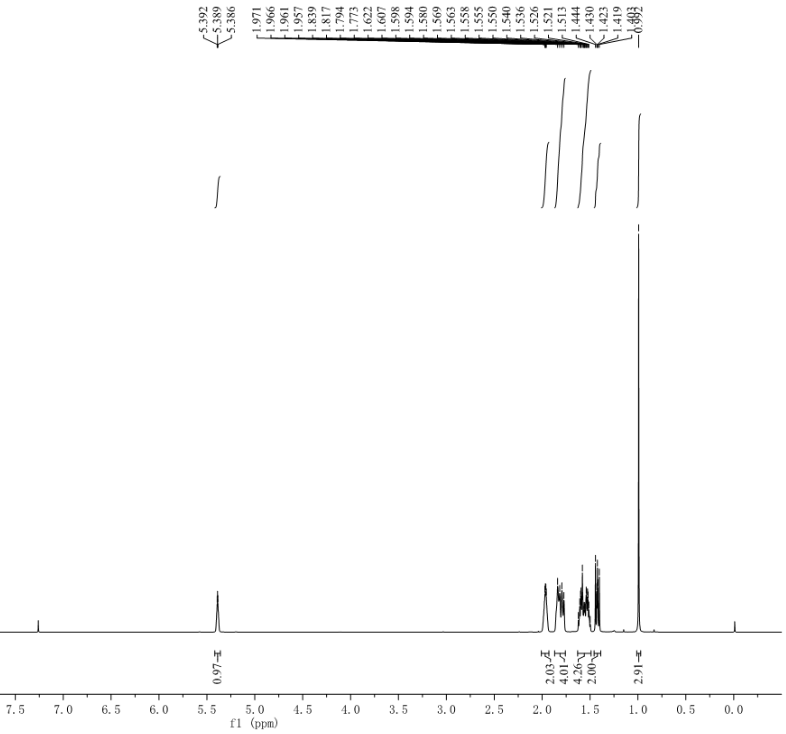
Parameter | Value
1 Origin | Bruker BioSpin GmbH
2 Solvent | CDCl3
3 Temperature | 292.7
4 Number of Scans | 16
5 Spectrometer Frequency | 400.15
6 Nucleus | 1H
5.392
5.389
5.386
1.971
1.966
1.961
1.957
1.839
1.817
1.794
1.773
1.622
1.607
1.598
1.594
1.580
1.569
1.563
1.558
1.555
1.550
1.540
1.536
1.526
1.521
1.513
1.444
1.430
1.423
1.419
1.403
0.992
0.97
2.03
4.01
4.26
2.00
2.91
10.5 10.0 9.5 9.0 8.5 8.0 7.5 7.0 6.5 6.0 5.5 5.0 4.5 4.0 3.5 3.0 2.5 2.0 1.5 1.0 0.5 0.0
f1 (ppm)

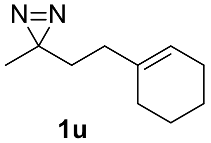
N=N

1u

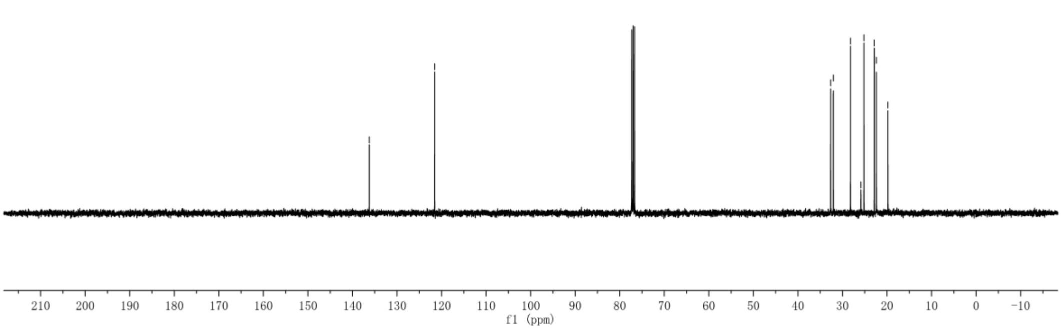

Parameter	Value
1 Origin	Bruker BioSpin GmbH
2 Solvent	CDCl3
3 Temperature	293.1
4 Number of Scans	80
5 Spectrometer Frequency	100.62
6 Nucleus	13C

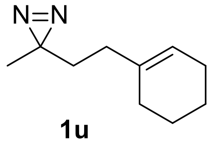

1u

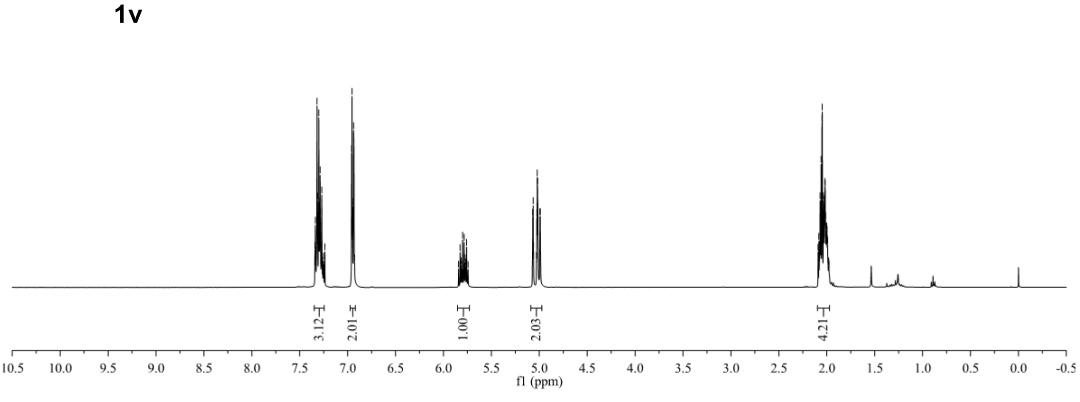

Parameter	Value
1 Origin	Bruker BioSpin GmbH
2 Solvent	CDCl3
3 Temperature	298.1
4 Number of Scans	2
5 Spectrometer Frequency	400.13
6 Nucleus	1H

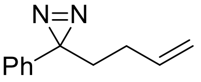

1v

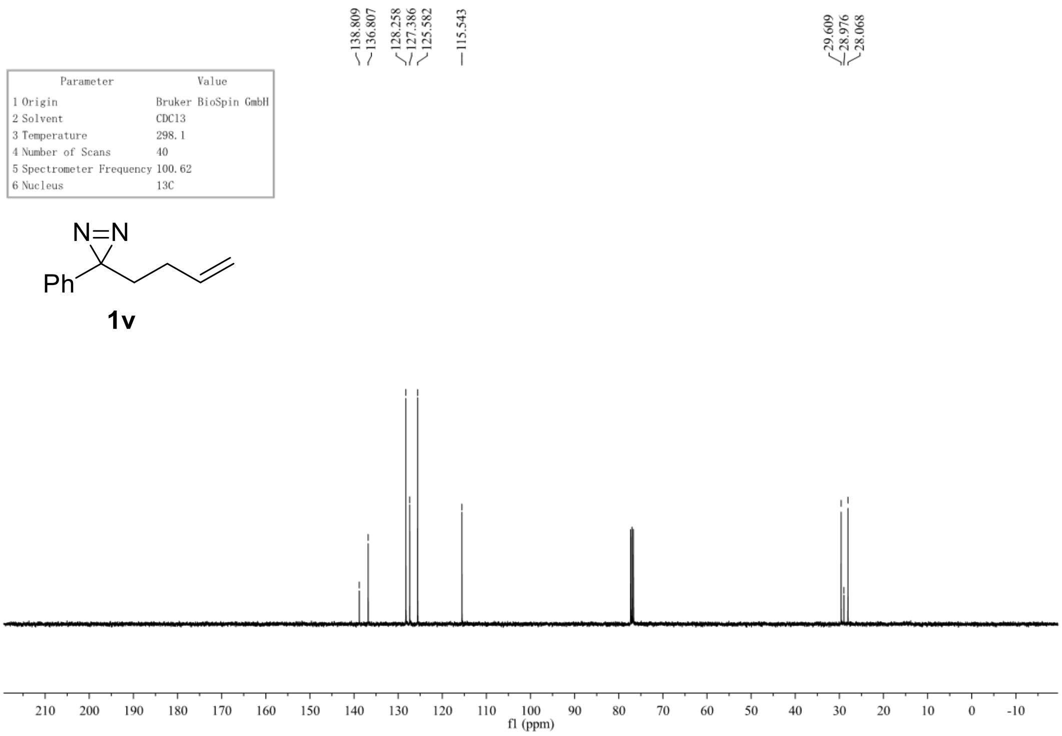
138.809
136.807
128.258
127.386
125.582
115.543
29.609
28.976
28.068
Parameter | Value
1 Origin | Bruker BioSpin GmbH
2 Solvent | CDCl3
3 Temperature | 298.1
4 Number of Scans | 40
5 Spectrometer Frequency | 100.62
6 Nucleus | 13C
N=N
Ph
1v
210 200 190 180 170 160 150 140 130 120 110 100 90 80 70 60 50 40 30 20 10 0 -10
f1 (ppm)

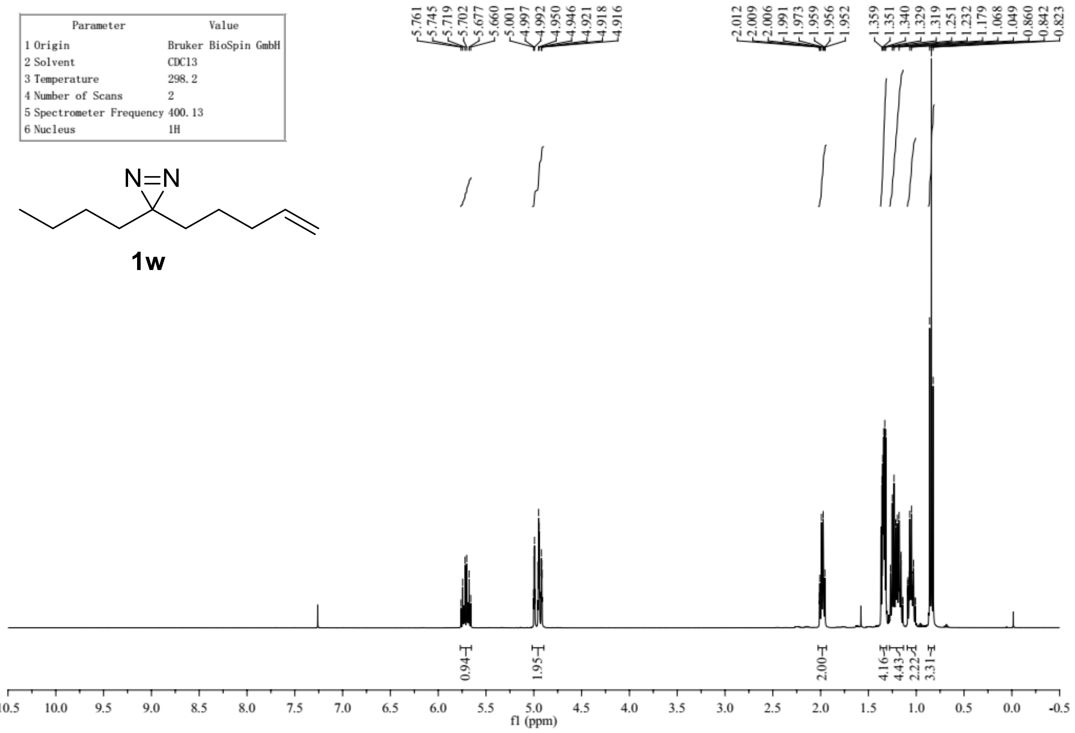
5.761
5.745
5.719
5.702
5.677
5.660
5.001
4.997
4.992
4.950
4.946
4.921
4.918
4.916
2.012
2.009
2.006
1.991
1.973
1.959
1.956
1.952
1.359
1.351
1.340
1.329
1.319
1.251
1.232
1.179
1.068
1.049
0.860
0.842
0.823
Parameter | Value
1 Origin | Bruker BioSpin GmbH
2 Solvent | CDCl3
3 Temperature | 298.2
4 Number of Scans | 2
5 Spectrometer Frequency | 400.13
6 Nucleus | 1H
N=N
1w
0.94
1.95
2.00
4.16
4.43
2.22
3.31
10.5 10.0 9.5 9.0 8.5 8.0 7.5 7.0 6.5 6.0 5.5 5.0 4.5 4.0 3.5 3.0 2.5 2.0 1.5 1.0 0.5 0.0 -0.5
f1 (ppm)

Parameter	Value
1 Origin	Bruker BioSpin GmbH
2 Solvent	CDCl3
3 Temperature	298.2
4 Number of Scans	10
5 Spectrometer Frequency	100.61
6 Nucleus	13C

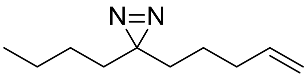

1w

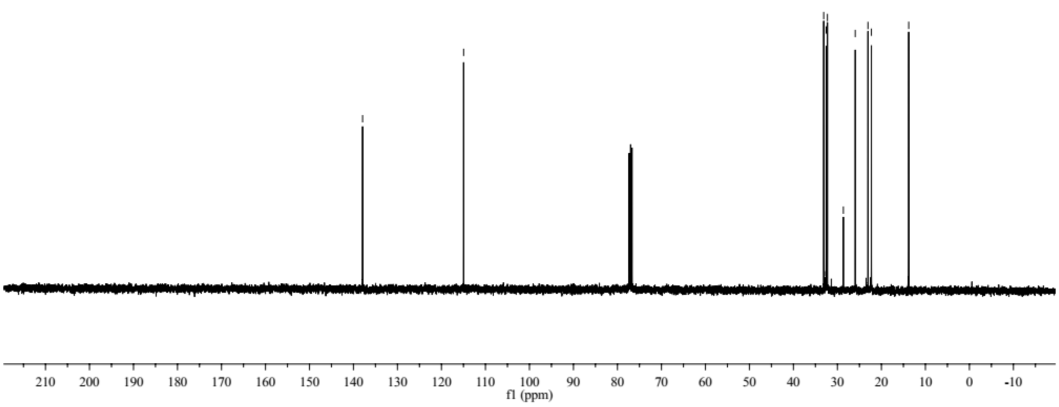

Parameter	Value
1 Origin	Bruker BioSpin GmbH
2 Solvent	CDCl3
3 Temperature	298.1
4 Number of Scans	22
5 Spectrometer Frequency	400.13
6 Nucleus	1H

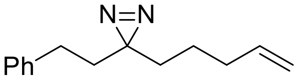

1x

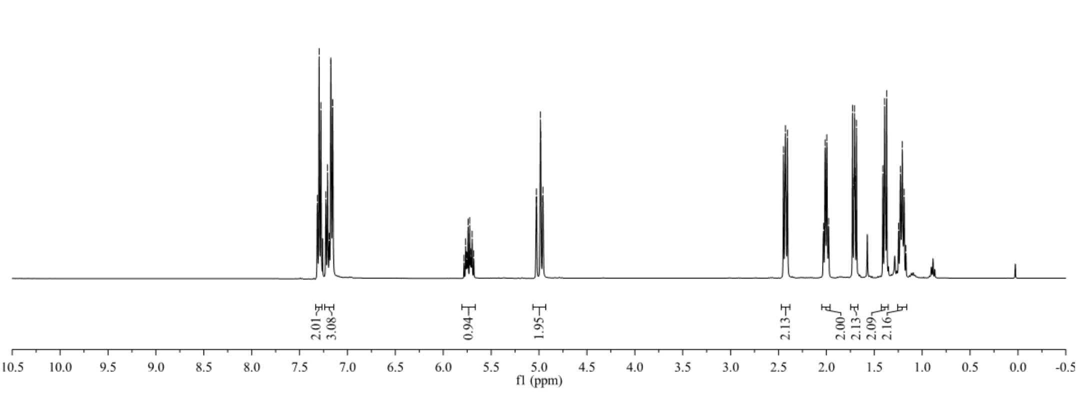

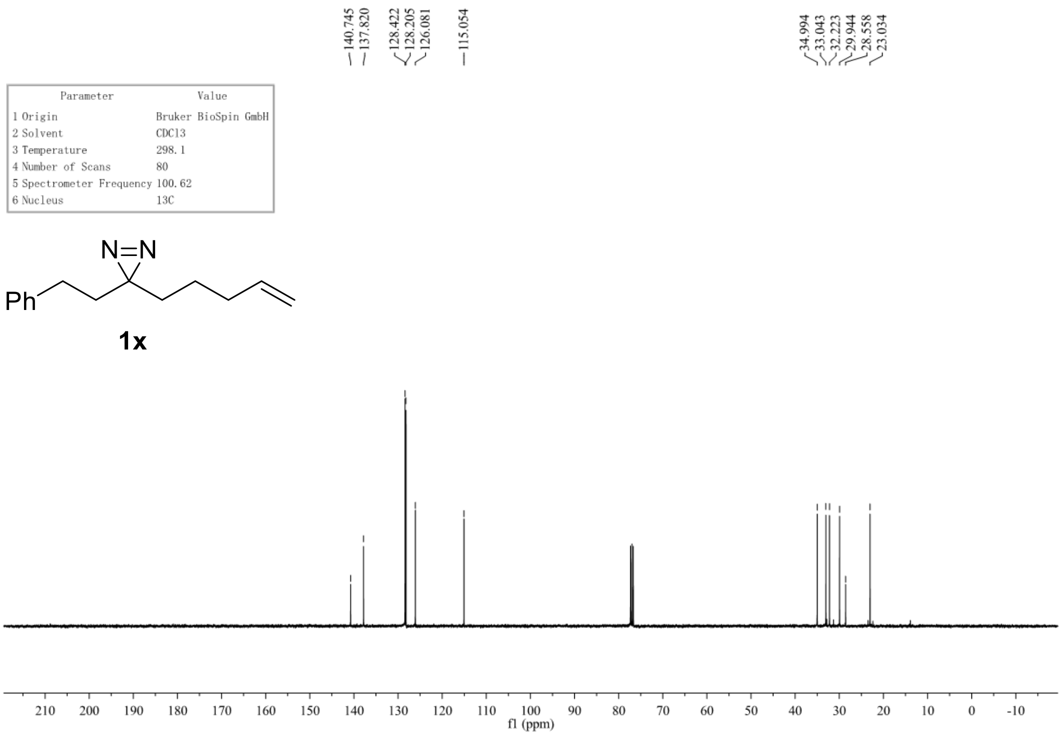
140.745
137.820
128.422
128.205
126.081
115.054
34.994
33.043
32.223
29.944
28.558
23.034
Parameter Value
1 Origin Bruker BioSpin GmbH
2 Solvent CDCl3
3 Temperature 298.1
4 Number of Scans 80
5 Spectrometer Frequency 100.62
6 Nucleus 13C
N=N
Ph
1x
210 200 190 180 170 160 150 140 130 120 110 100 90 80 70 60 50 40 30 20 10 0 -10
f1 (ppm)

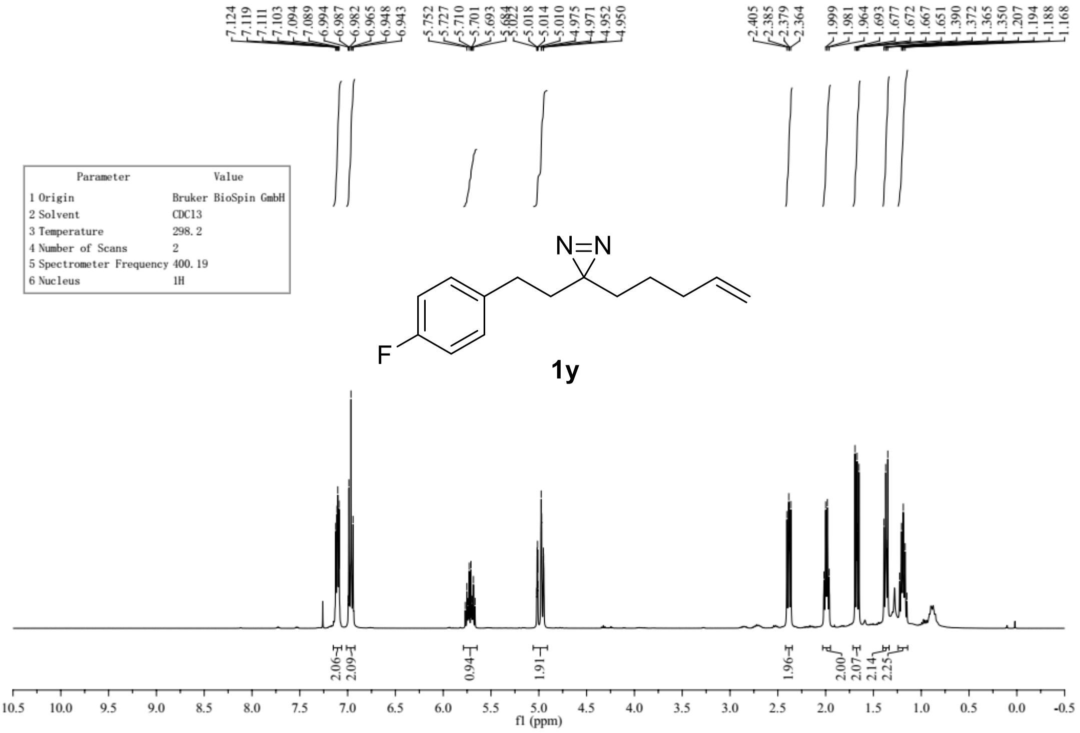
7.124
7.119
7.111
7.103
7.094
7.089
6.994
6.987
6.982
6.965
6.948
6.943
5.752
5.727
5.710
5.701
5.693
5.684
5.022
5.018
5.014
5.010
4.975
4.971
4.952
4.950
2.405
2.385
2.379
2.364
1.999
1.981
1.964
1.693
1.677
1.672
1.667
1.651
1.390
1.372
1.365
1.350
1.207
1.194
1.188
1.168
Parameter Value
1 Origin Bruker BioSpin GmbH
2 Solvent CDCl3
3 Temperature 298.2
4 Number of Scans 2
5 Spectrometer Frequency 400.19
6 Nucleus 1H
N=N
F
1y
2.06
2.09
0.94
1.91
1.96
2.00
2.07
2.14
2.25
10.5 10.0 9.5 9.0 8.5 8.0 7.5 7.0 6.5 6.0 5.5 5.0 4.5 4.0 3.5 3.0 2.5 2.0 1.5 1.0 0.5 0.0 -0.5
f1 (ppm)

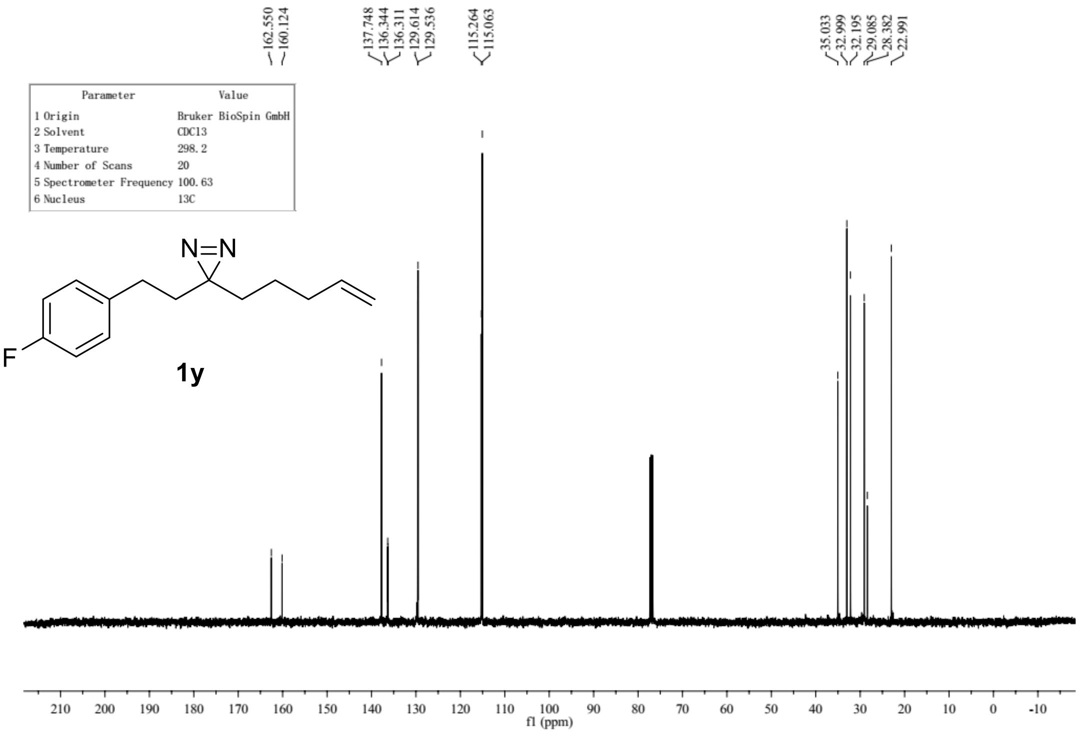
162.550
160.124
137.748
136.344
136.311
129.614
129.536
115.264
115.063
35.033
32.999
32.195
29.085
28.382
22.991
Parameter
Value
1 Origin
Bruker BioSpin GmbH
2 Solvent
CDCl3
3 Temperature
298.2
4 Number of Scans
20
5 Spectrometer Frequency
100.63
6 Nucleus
13C
N=N
F
1y
fl (ppm)

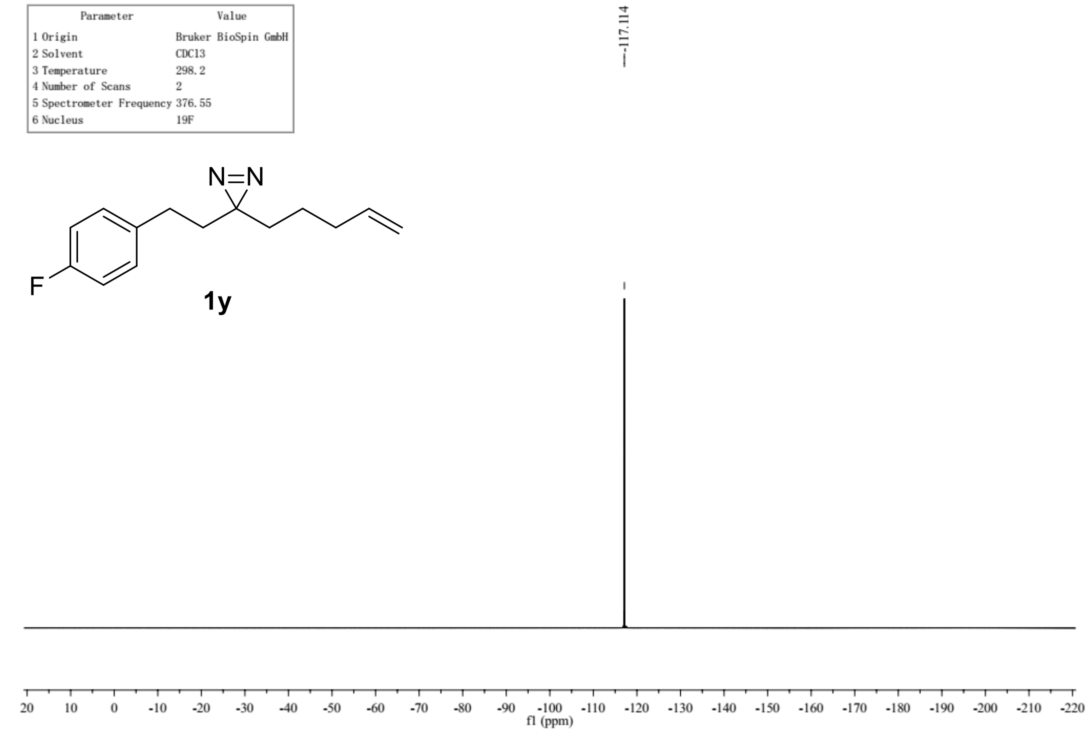
Parameter
Value
1 Origin
Bruker BioSpin GmbH
2 Solvent
CDCl3
3 Temperature
298.2
4 Number of Scans
2
5 Spectrometer Frequency
376.55
6 Nucleus
19F
-117.114
N=N
F
1y
fl (ppm)

Parameter	Value
1 Origin	Bruker BioSpin GmbH
2 Solvent	CDCl3
3 Temperature	298.1
4 Number of Scans	2
5 Spectrometer Frequency	400.13
6 Nucleus	1H

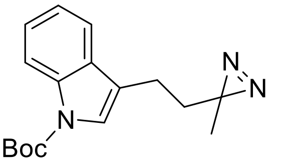

1z

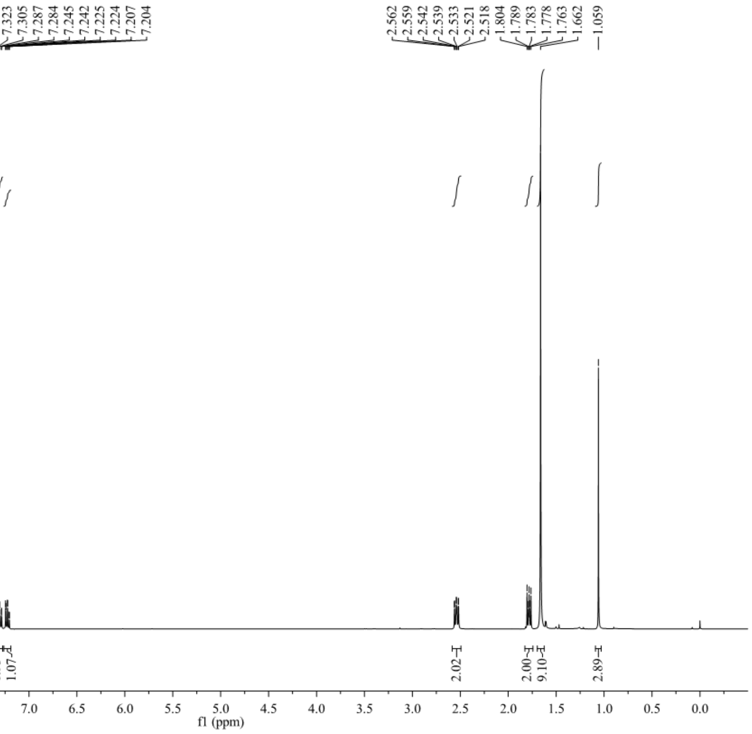

Parameter	Value
1 Origin	Bruker BioSpin GmbH
2 Solvent	CDCl3
3 Temperature	298.2
4 Number of Scans	80
5 Spectrometer Frequency	100.62
6 Nucleus	13C

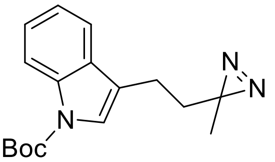

1z

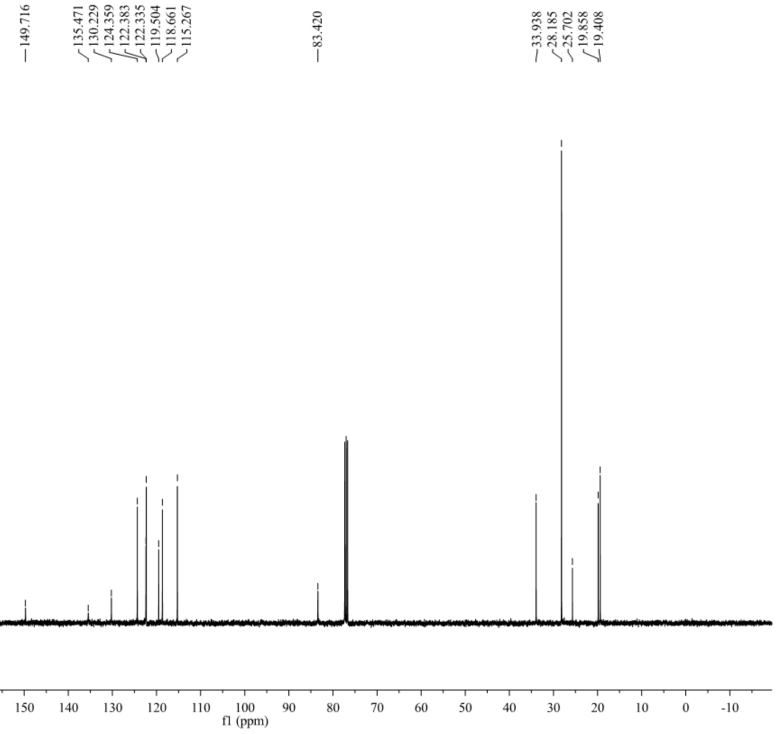

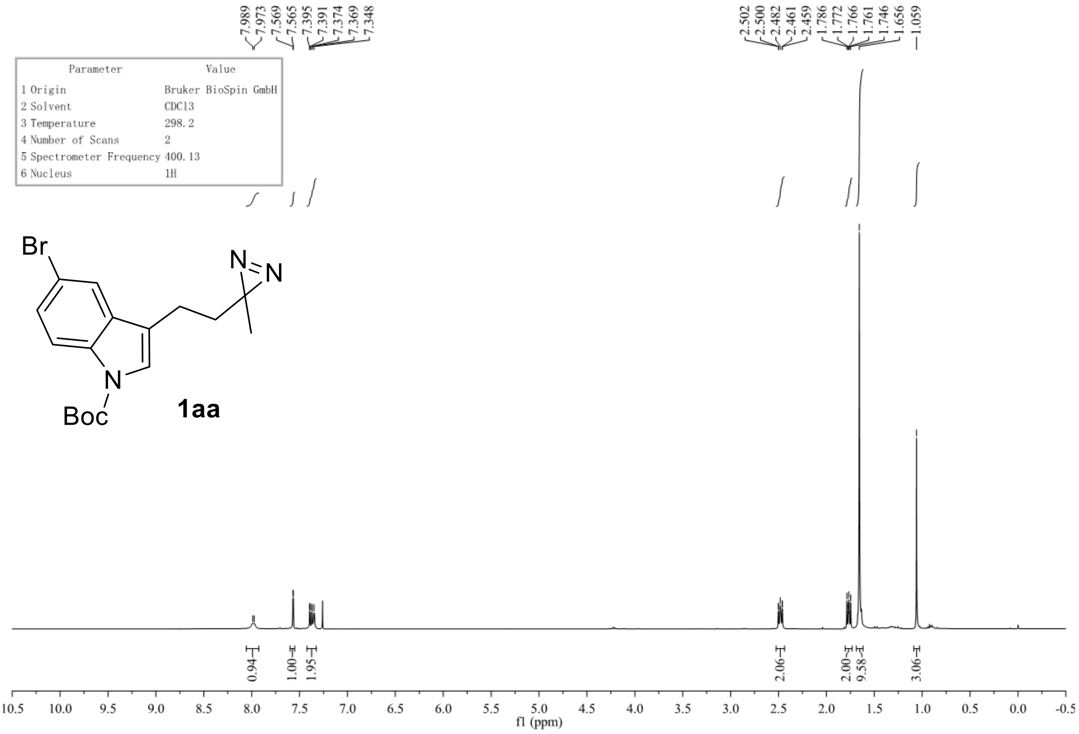

Parameter Value
1 Origin Bruker BioSpin GmbH
2 Solvent CDCl3
3 Temperature 298.2
4 Number of Scans 2
5 Spectrometer Frequency 400.13
6 Nucleus 1H
7.989
7.973
7.569
7.565
7.395
7.391
7.374
7.369
7.348
2.502
2.500
2.482
2.461
2.459
1.786
1.772
1.766
1.761
1.746
1.656
1.059
Br
N
N
N
Boc
1aa
0.94
1.00
1.95
2.06
2.00
9.58
3.06
fl (ppm)

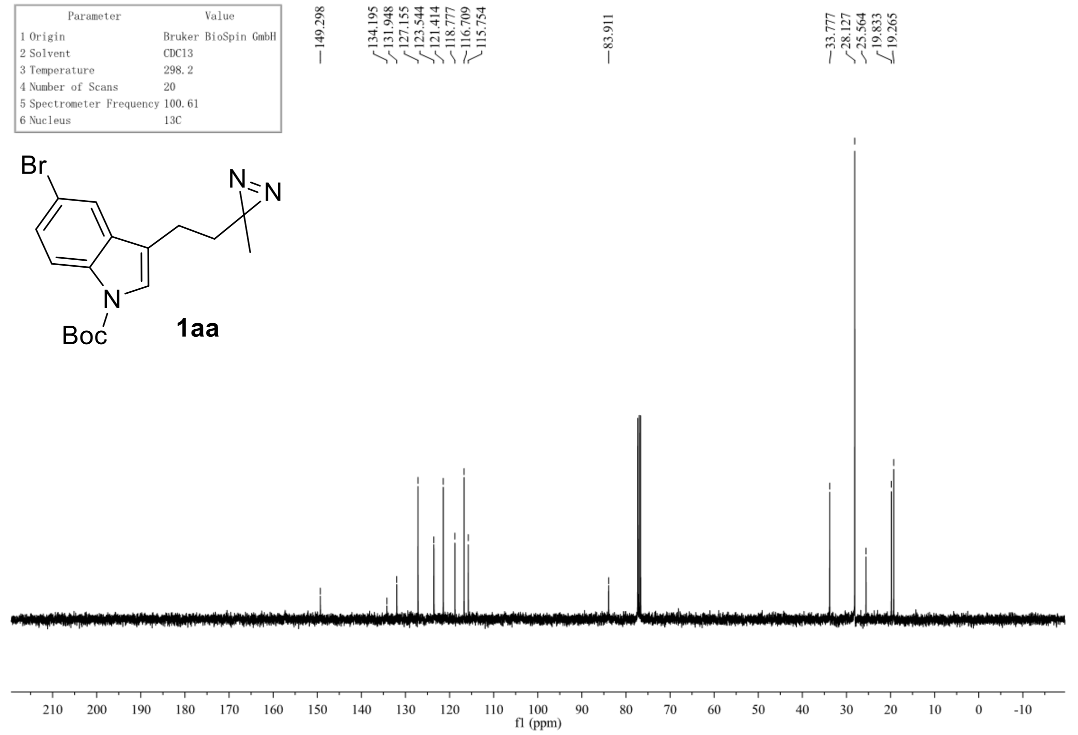

Parameter Value
1 Origin Bruker BioSpin GmbH
2 Solvent CDCl3
3 Temperature 298.2
4 Number of Scans 20
5 Spectrometer Frequency 100.61
6 Nucleus 13C
149.298
134.195
131.948
127.155
123.544
121.414
118.777
116.709
115.754
83.911
33.777
28.127
25.564
19.833
19.265
Br
N
N
N
Boc
1aa
fl (ppm)

Parameter	Value
1 Origin	Bruker BioSpin GmbH
2 Solvent	CDCl3
3 Temperature	298.1
4 Number of Scans	2
5 Spectrometer Frequency	400.13
6 Nucleus	1H

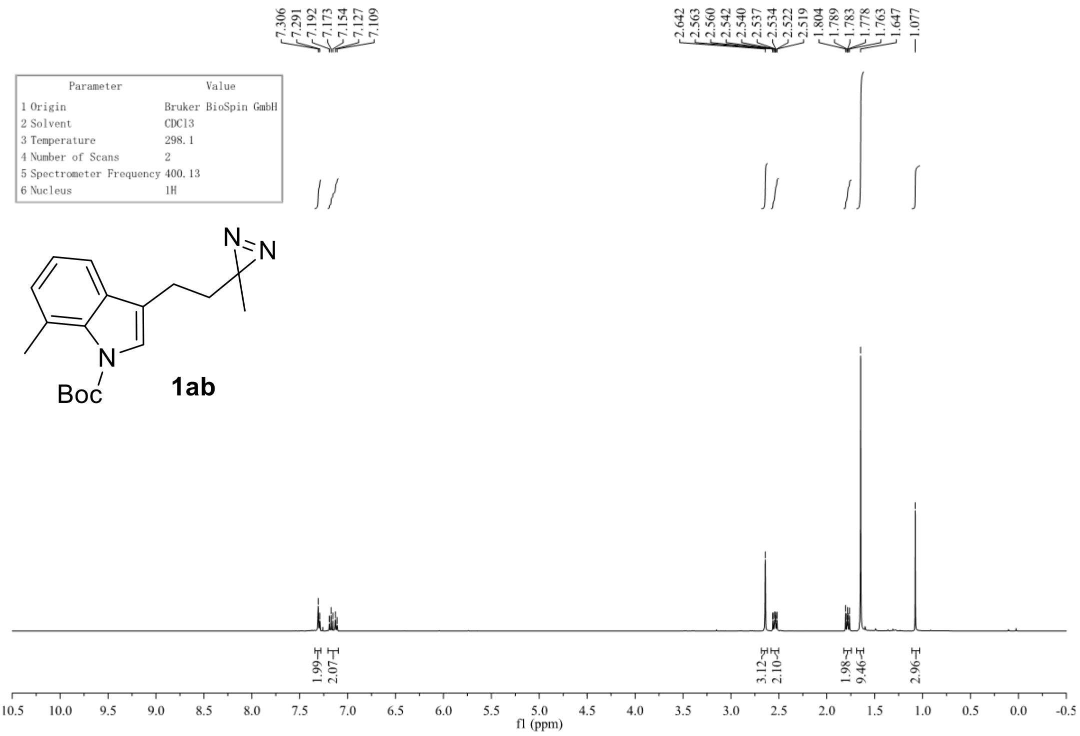

Parameter	Value
1 Origin	Bruker BioSpin GmbH
2 Solvent	CDCl3
3 Temperature	298.1
4 Number of Scans	40
5 Spectrometer Frequency	100.62
6 Nucleus	13C

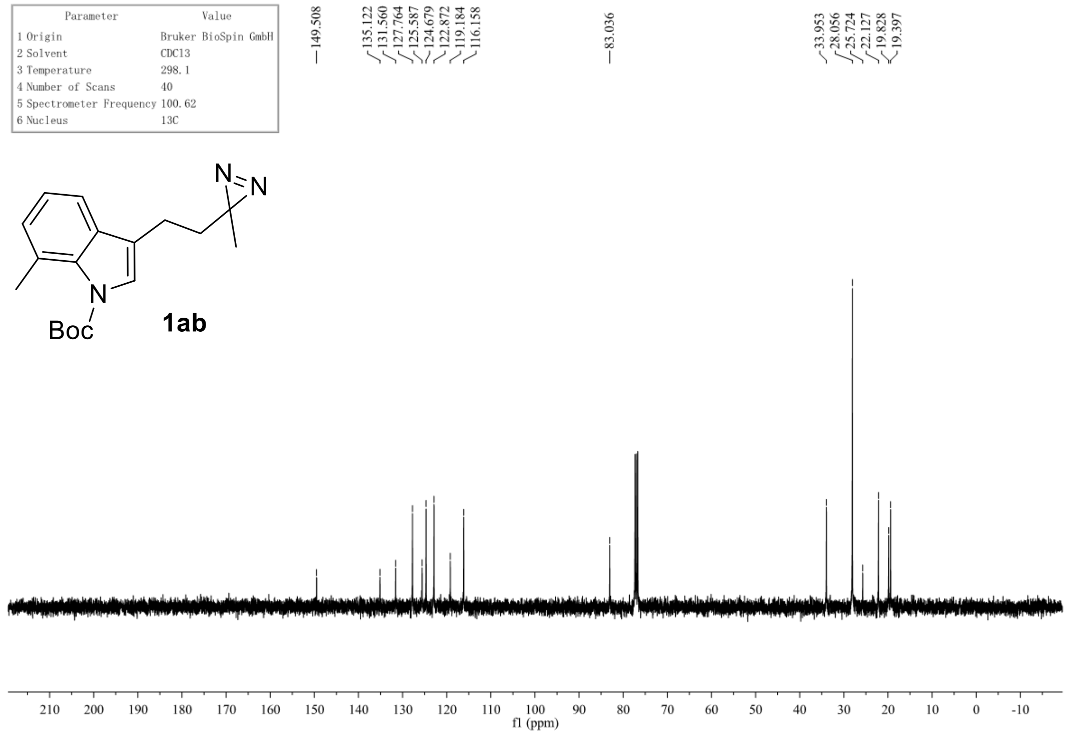

Parameter	Value
1 Origin	Bruker BioSpin GmbH
2 Solvent	CDCl3
3 Temperature	295.2
4 Number of Scans	16
5 Spectrometer Frequency	400.15
6 Nucleus	1H

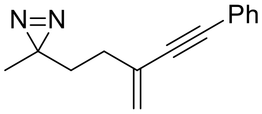

1ac

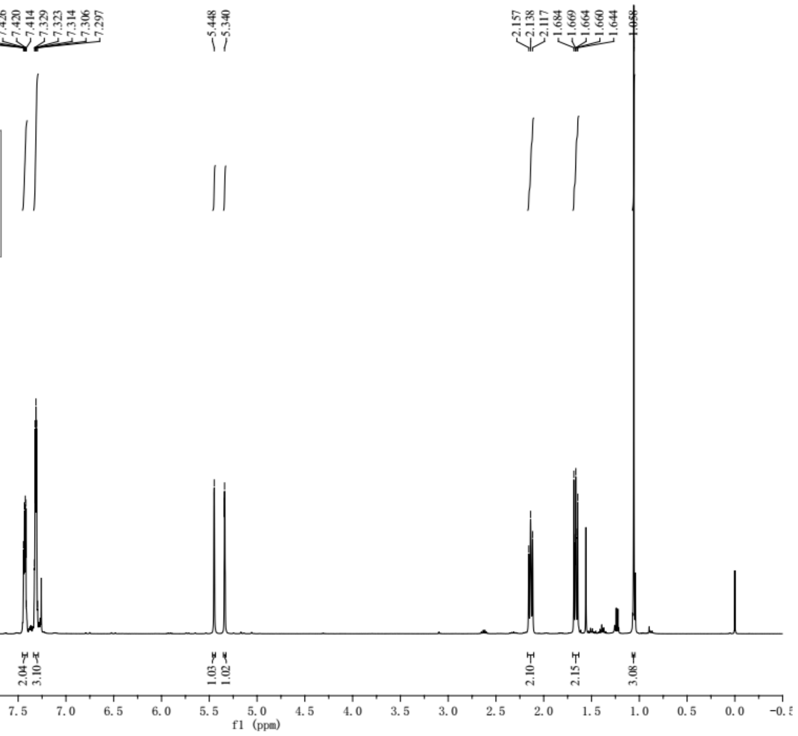

Parameter	Value
1 Origin	Bruker BioSpin GmbH
2 Solvent	CDCl3
3 Temperature	295.5
4 Number of Scans	80
5 Spectrometer Frequency	100.62
6 Nucleus	13C

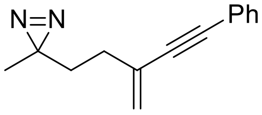

1ac

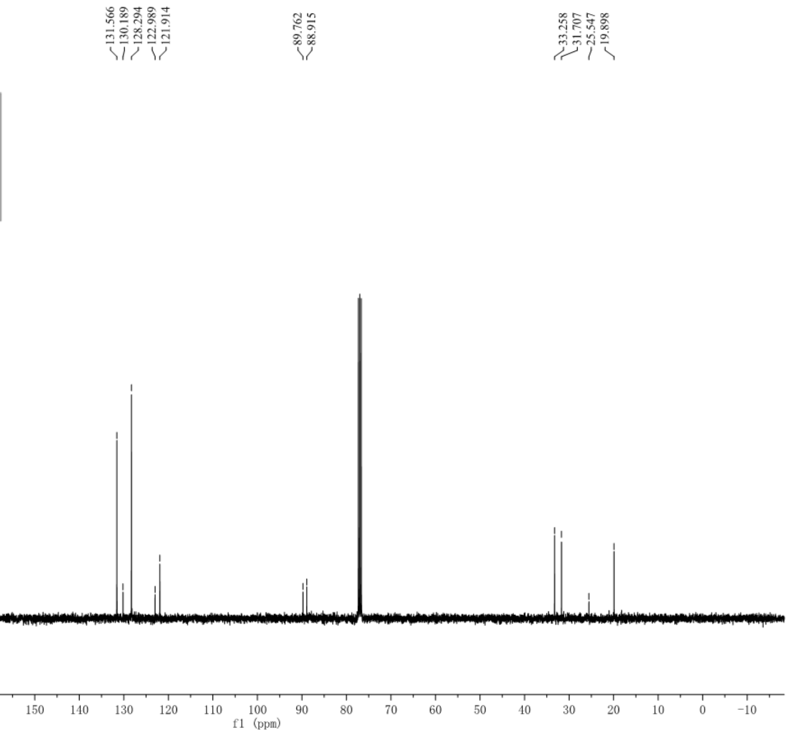

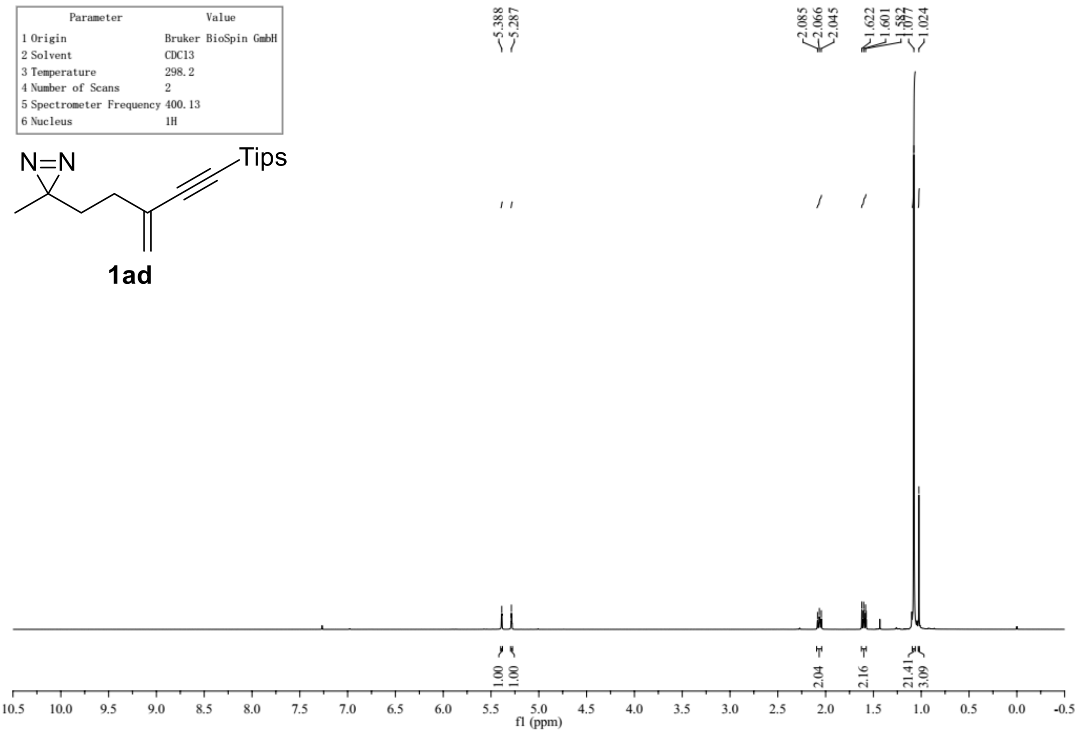
Parameter Value
1 Origin Bruker BioSpin GmbH
2 Solvent CDCl3
3 Temperature 298.2
4 Number of Scans 2
5 Spectrometer Frequency 400.13
6 Nucleus 1H
N=N
Tips
1ad
5.388
5.287
2.085
2.066
2.045
1.622
1.601
1.582
1.077
1.024
1.00
1.00
2.04
2.16
21.41
3.09
f1 (ppm)

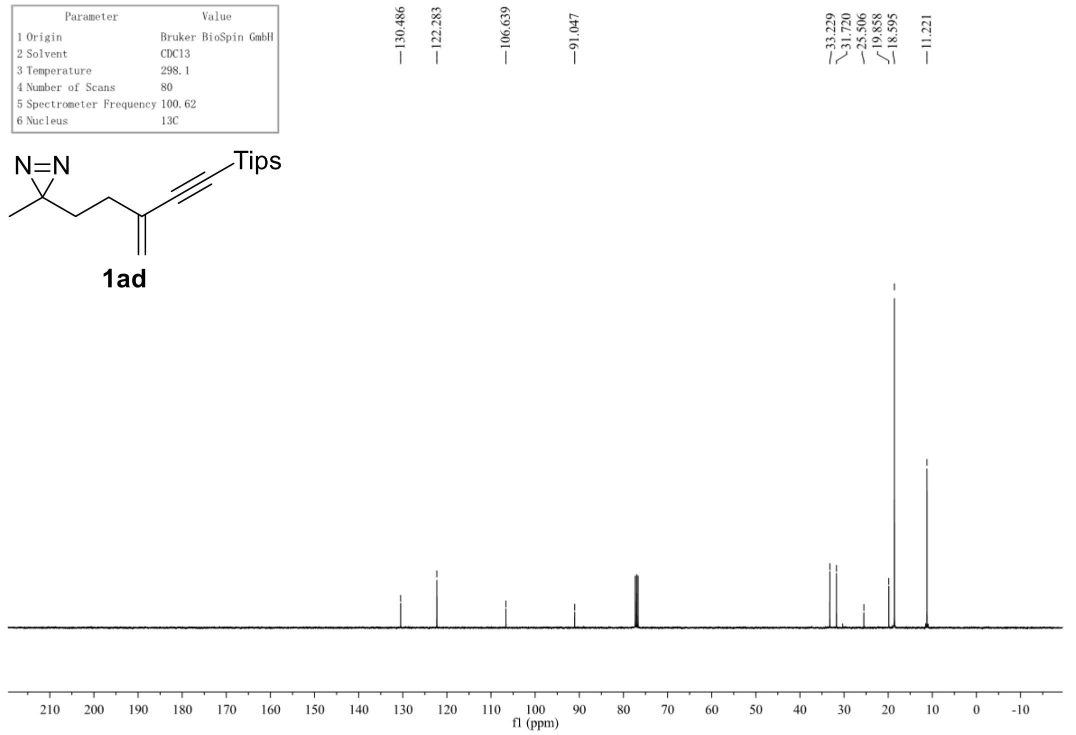
Parameter Value
1 Origin Bruker BioSpin GmbH
2 Solvent CDCl3
3 Temperature 298.1
4 Number of Scans 80
5 Spectrometer Frequency 100.62
6 Nucleus 13C
N=N
Tips
1ad
130.486
122.283
106.639
91.047
33.229
31.720
25.506
19.858
18.595
11.221
f1 (ppm)

Parameter	Value
1 Origin	Bruker BioSpin GmbH
2 Solvent	CDCl3
3 Temperature	295.7
4 Number of Scans	2
5 Spectrometer Frequency	400.15
6 Nucleus	1H

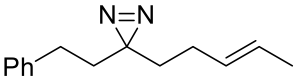

1ae

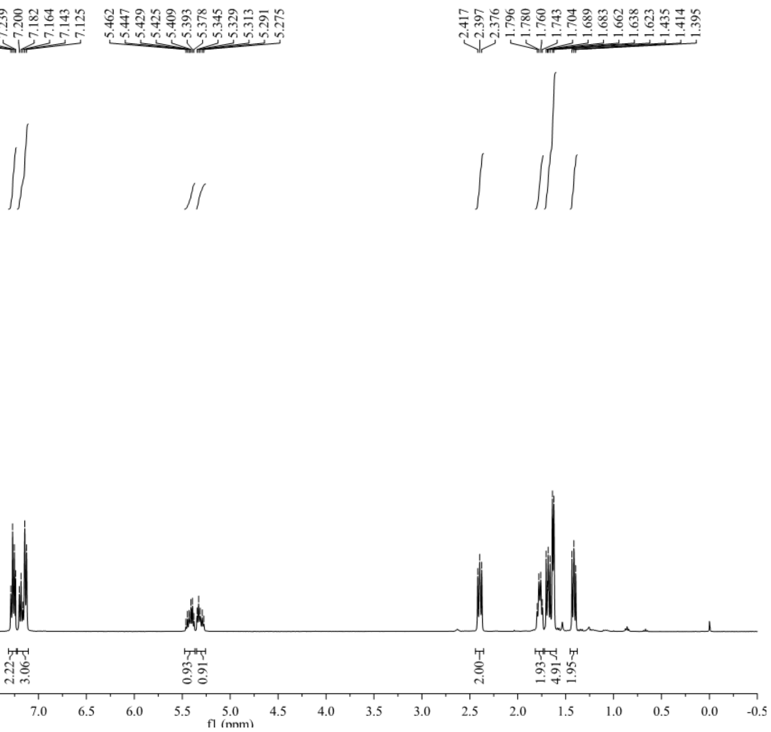

Parameter	Value
1 Origin	Bruker BioSpin GmbH
2 Solvent	CDCl3
3 Temperature	296.1
4 Number of Scans	80
5 Spectrometer Frequency	100.62
6 Nucleus	13C

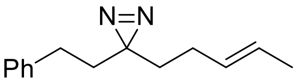

1ae

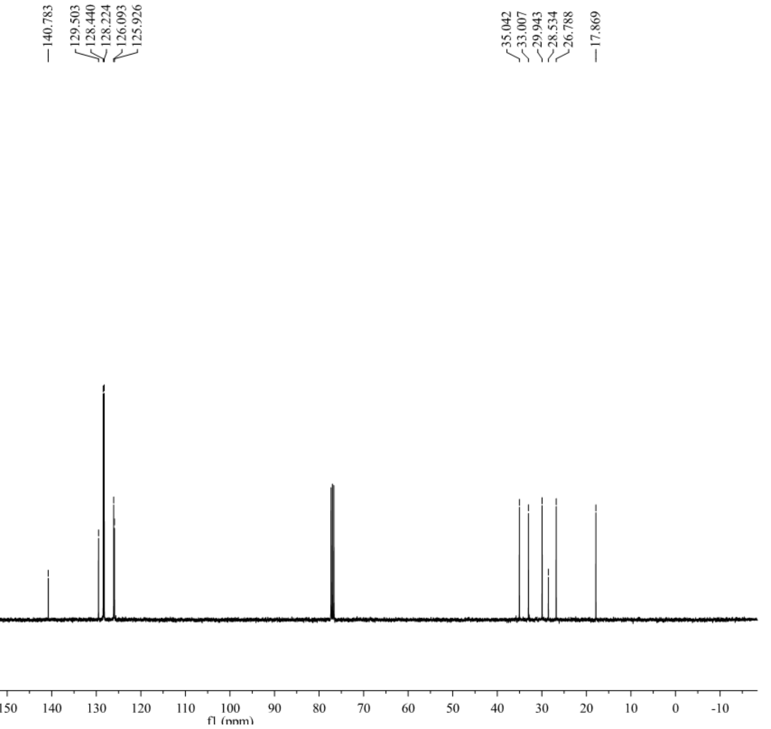

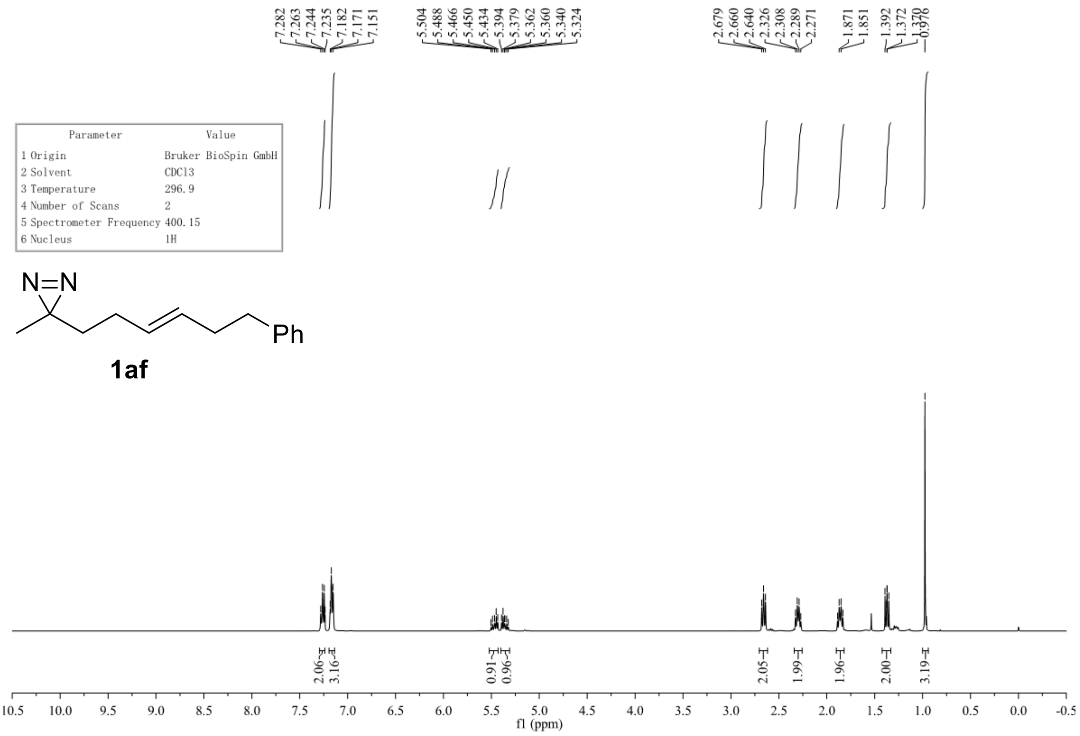
Parameter Value
1 Origin Bruker BioSpin GmbH
2 Solvent CDCl3
3 Temperature 296.9
4 Number of Scans 2
5 Spectrometer Frequency 400.15
6 Nucleus 1H
N=N
Ph
fl (ppm)

1af

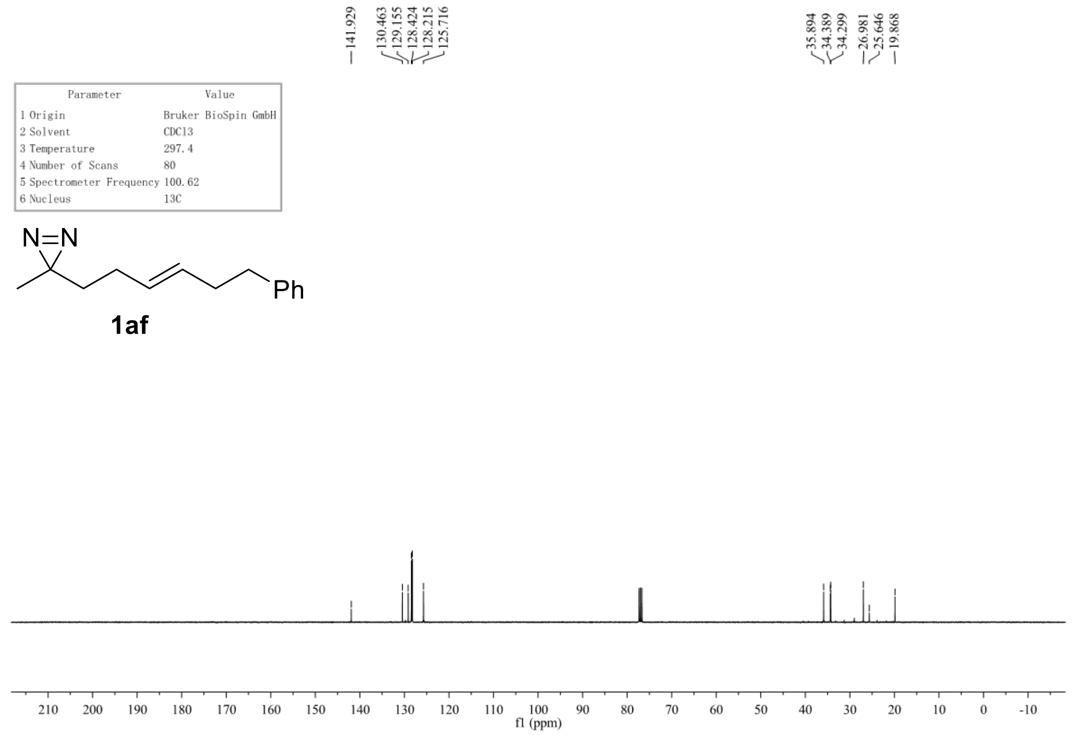
Parameter Value
1 Origin Bruker BioSpin GmbH
2 Solvent CDCl3
3 Temperature 297.4
4 Number of Scans 80
5 Spectrometer Frequency 100.62
6 Nucleus 13C
N=N
Ph
fl (ppm)

1af

Parameter	Value
1 Origin	Bruker BioSpin GmbH
2 Solvent	CDCl3
3 Temperature	298.1
4 Number of Scans	2
5 Spectrometer Frequency	400.13
6 Nucleus	1H

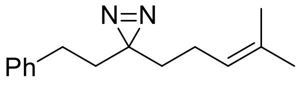

1ag

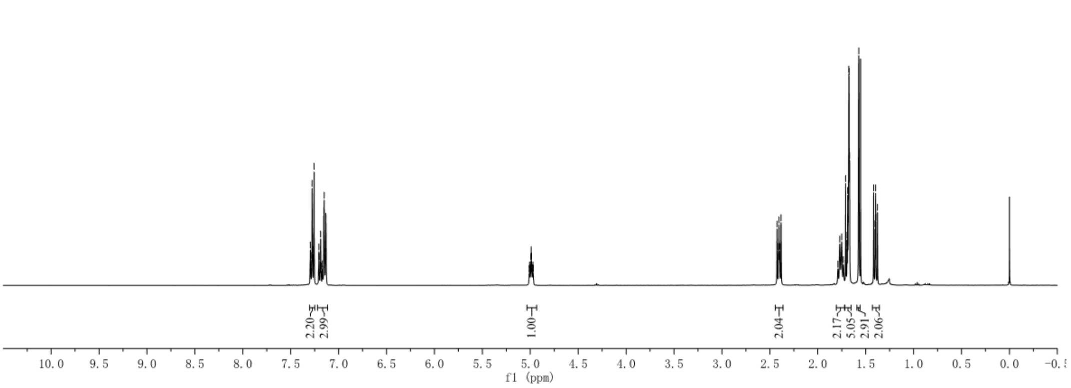

Parameter	Value
1 Origin	Bruker BioSpin GmbH
2 Solvent	CDCl3
3 Temperature	298.2
4 Number of Scans	70
5 Spectrometer Frequency	100.61
6 Nucleus	13C

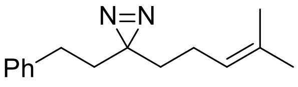

1ag

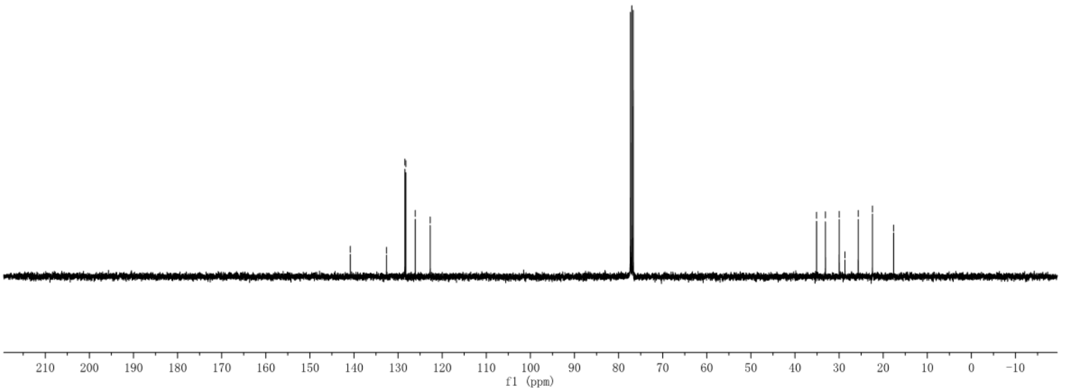

Parameter	Value
1 Origin	Bruker BioSpin GmbH
2 Solvent	CDCl3
3 Temperature	298.1
4 Number of Scans	2
5 Spectrometer Frequency	400.13
6 Nucleus	1H

1ah

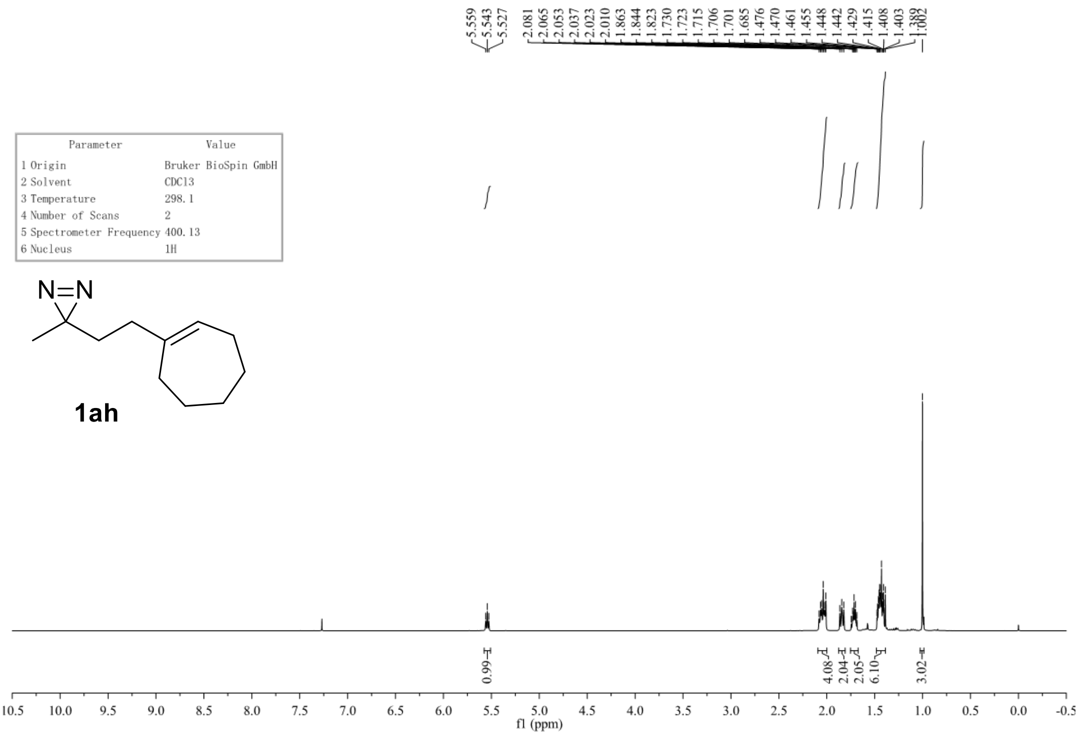

Parameter	Value
1 Origin	Bruker BioSpin GmbH
2 Solvent	CDCl3
3 Temperature	296.0
4 Number of Scans	80
5 Spectrometer Frequency	100.62
6 Nucleus	13C

1ah

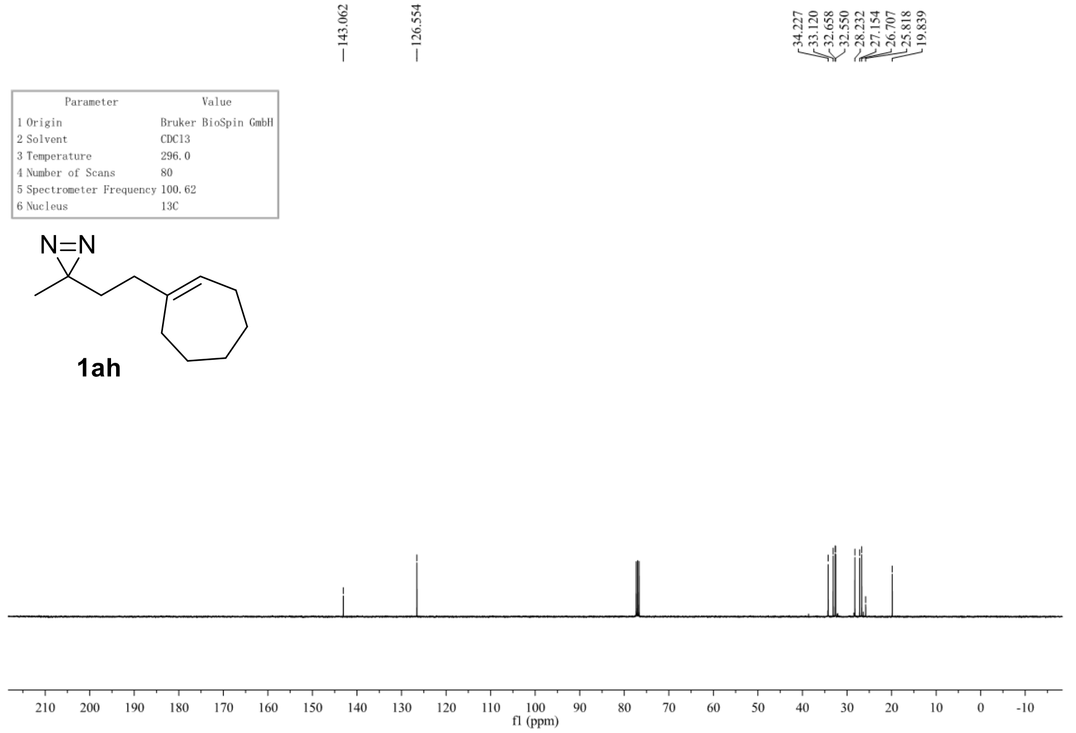

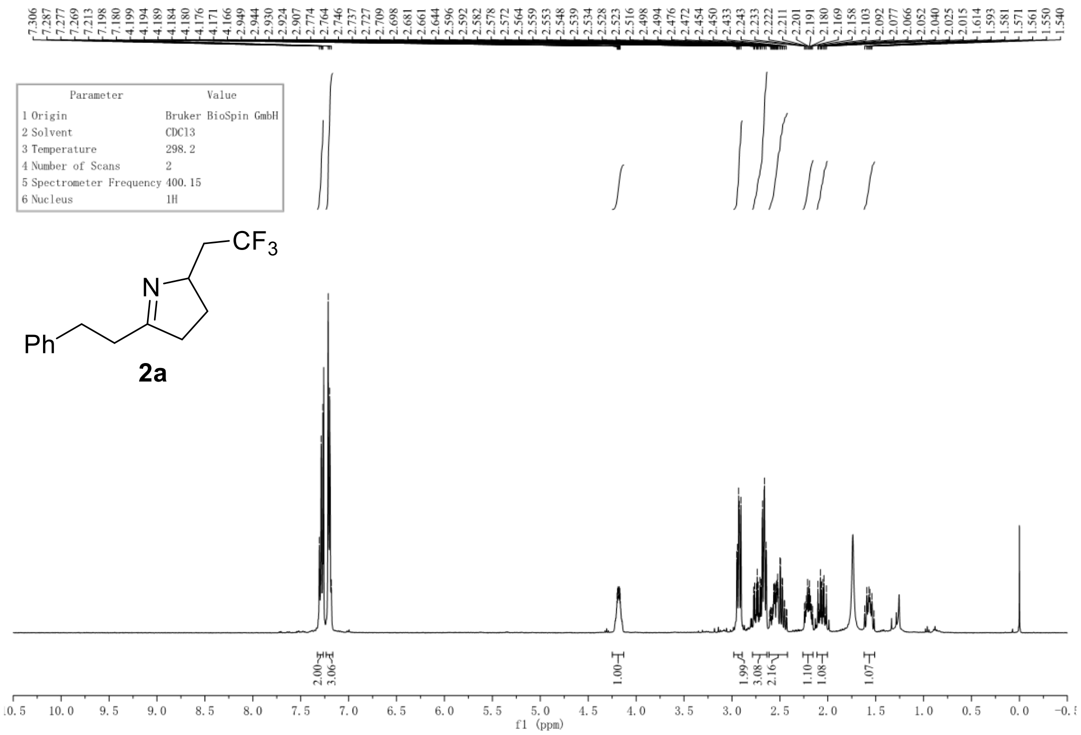

Parameter Value
1 Origin Bruker BioSpin GmbH
2 Solvent CDCl3
3 Temperature 298.2
4 Number of Scans 2
5 Spectrometer Frequency 400.15
6 Nucleus 1H
2a
f1 (ppm)

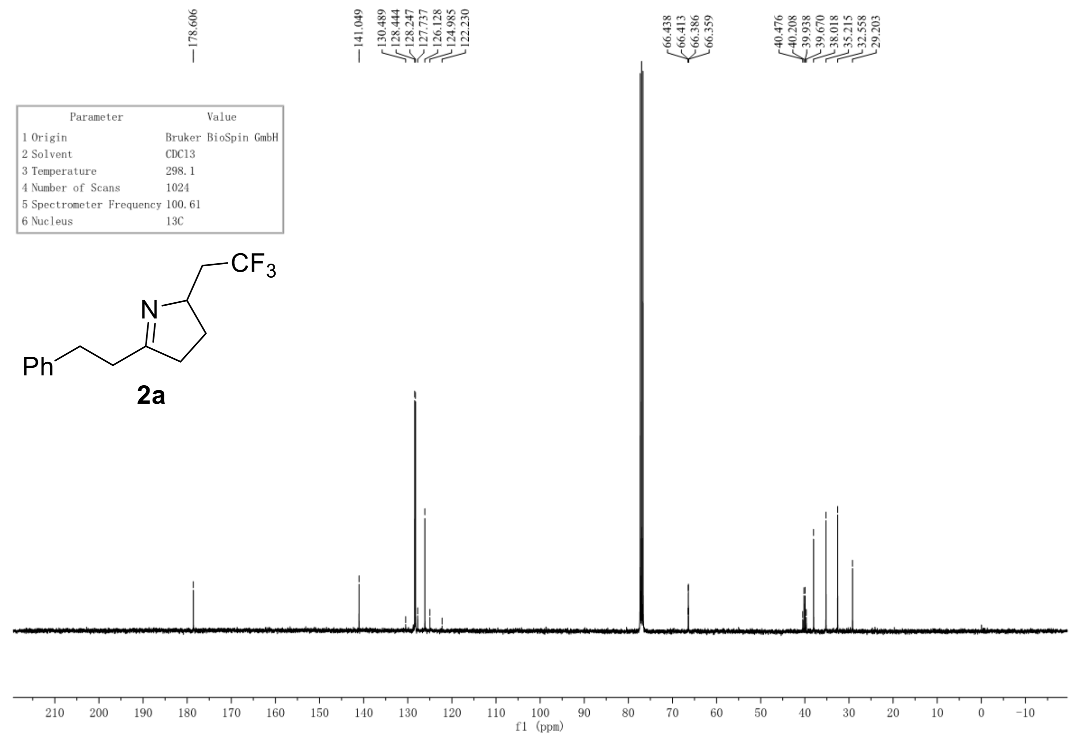

Parameter Value
1 Origin Bruker BioSpin GmbH
2 Solvent CDCl3
3 Temperature 298.1
4 Number of Scans 1024
5 Spectrometer Frequency 100.61
6 Nucleus 13C
2a
f1 (ppm)

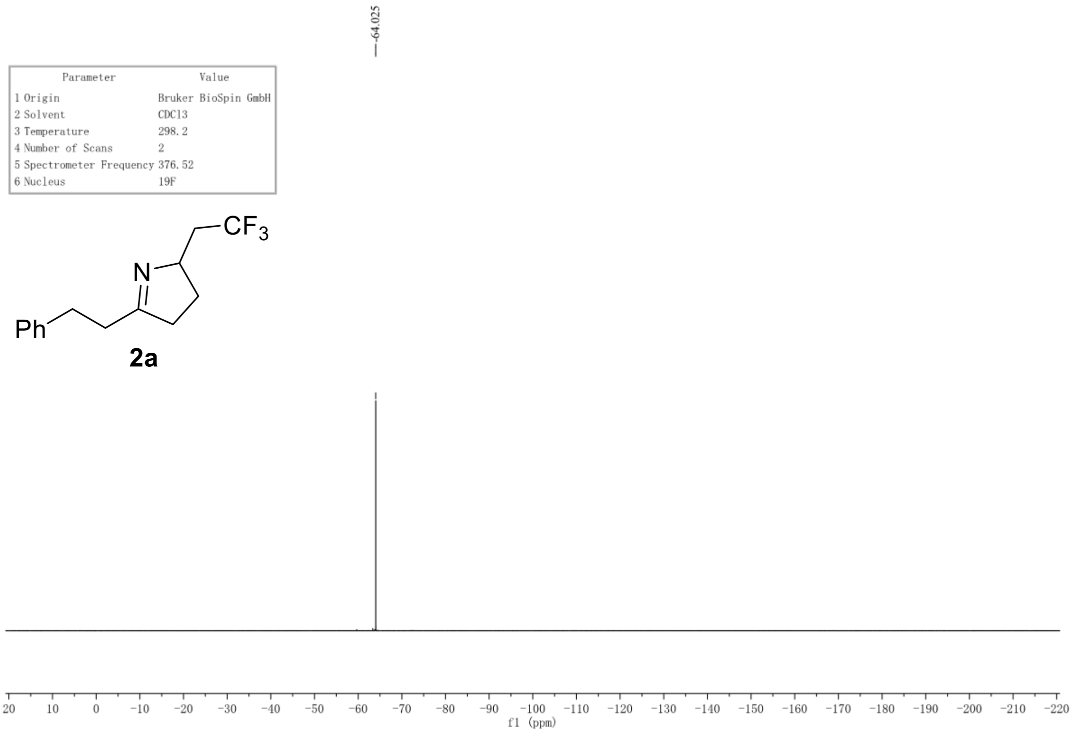
-64.025
Parameter Value
1 Origin Bruker BioSpin GmbH
2 Solvent CDCl3
3 Temperature 298.2
4 Number of Scans 2
5 Spectrometer Frequency 376.52
6 Nucleus 19F
CF3
N
Ph
2a
20 10 0 -10 -20 -30 -40 -50 -60 -70 -80 -90 -100 -110 -120 -130 -140 -150 -160 -170 -180 -190 -200 -210 -220
f1 (ppm)

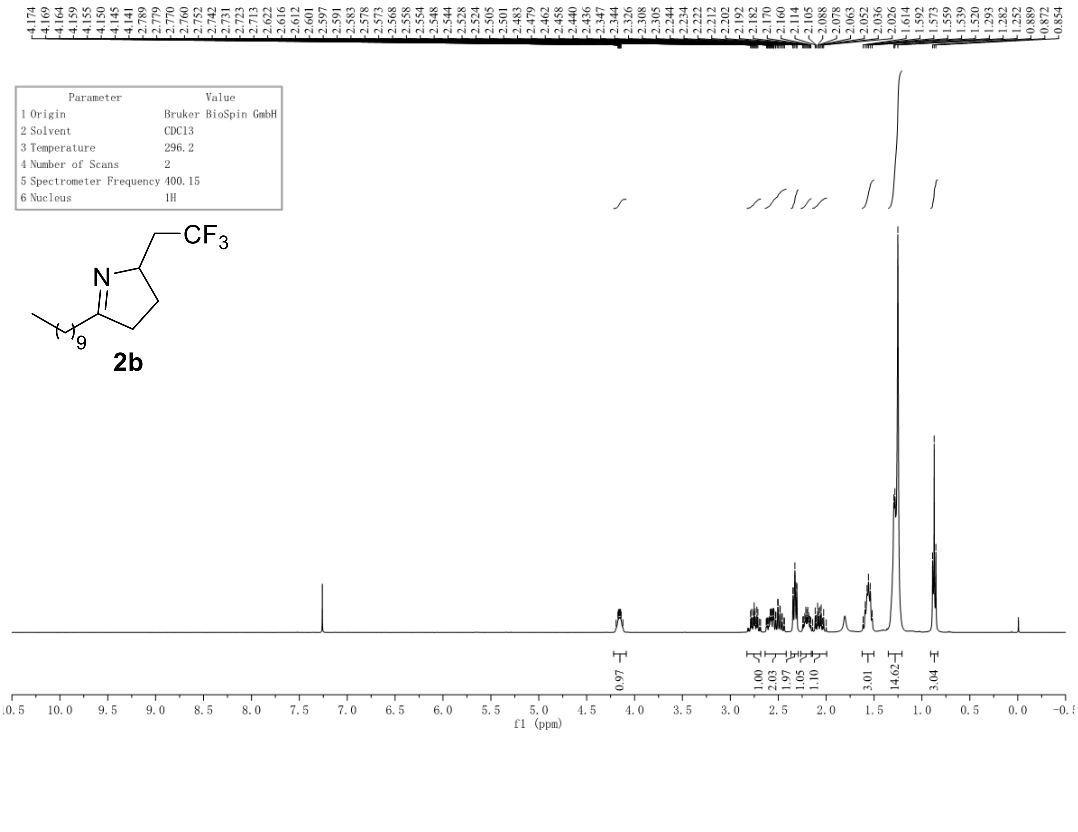
4.174 4.169 4.164 4.159 4.155 4.150 4.145 4.141 2.789 2.779 2.770 2.760 2.752 2.742 2.731 2.723 2.713 2.622 2.616 2.612 2.601 2.597 2.591 2.583 2.578 2.573 2.568 2.558 2.554 2.548 2.544 2.528 2.524 2.505 2.501 2.483 2.479 2.462 2.458 2.440 2.436 2.347 2.344 2.326 2.308 2.305 2.244 2.234 2.222 2.212 2.202 2.192 2.182 2.170 2.160 2.114 2.105 2.088 2.078 2.063 2.052 2.036 2.026 1.614 1.592 1.573 1.559 1.539 1.520 1.293 1.282 1.252 0.889 0.872 0.854
Parameter Value
1 Origin Bruker BioSpin GmbH
2 Solvent CDCl3
3 Temperature 296.2
4 Number of Scans 2
5 Spectrometer Frequency 400.15
6 Nucleus 1H
CF3
N
9
2b
0.97
1.00
2.03
1.97
1.05
1.10
3.01
14.62
3.04
10.5 10.0 9.5 9.0 8.5 8.0 7.5 7.0 6.5 6.0 5.5 5.0 4.5 4.0 3.5 3.0 2.5 2.0 1.5 1.0 0.5 0.0 -0.5
f1 (ppm)

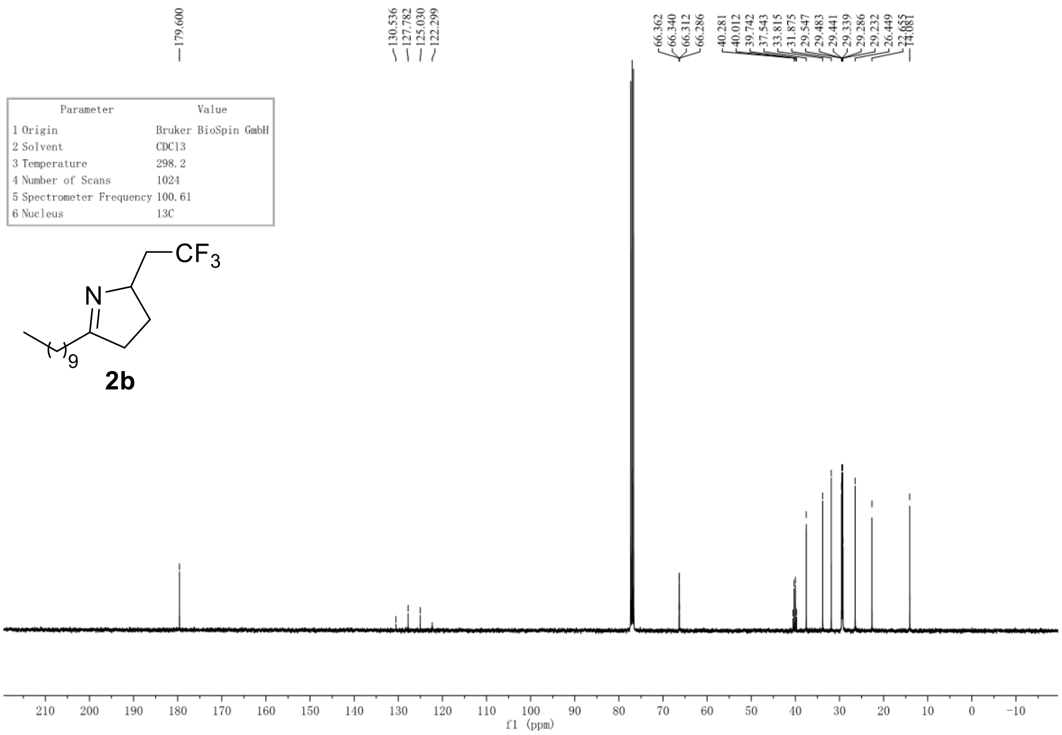
Parameter Value
1 Origin Bruker BioSpin GmbH
2 Solvent CDCl3
3 Temperature 298.2
4 Number of Scans 1024
5 Spectrometer Frequency 100.61
6 Nucleus 13C
CF3
N
9
2b
f1 (ppm)

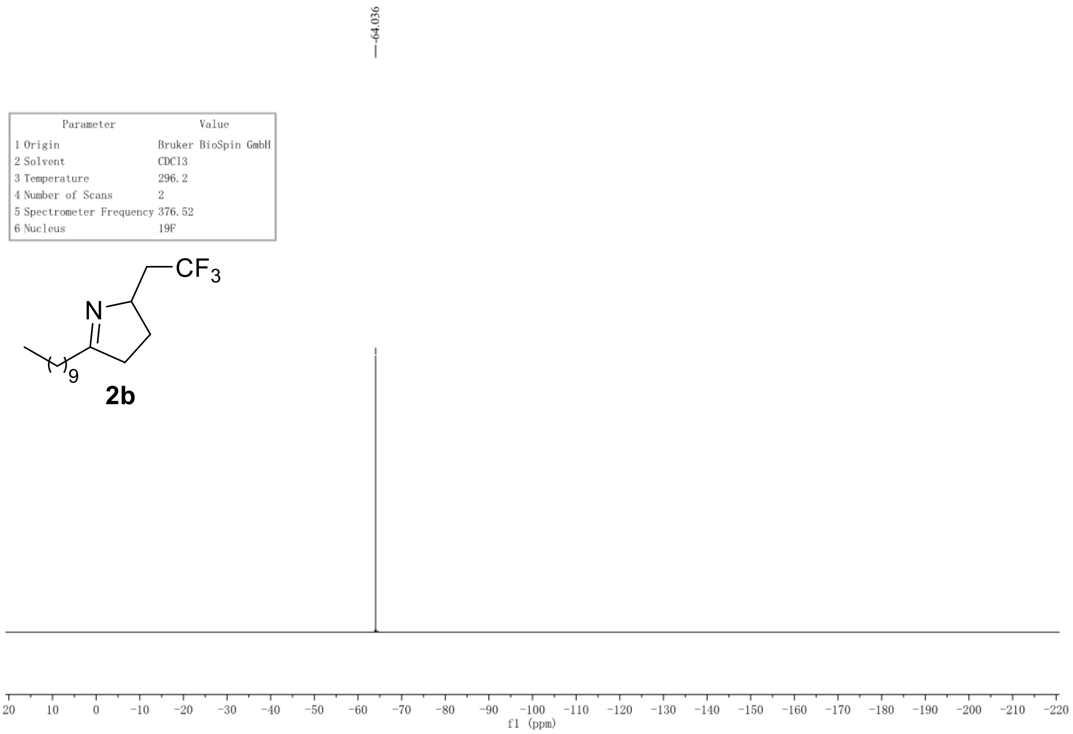
-64.036
Parameter Value
1 Origin Bruker BioSpin GmbH
2 Solvent CDCl3
3 Temperature 296.2
4 Number of Scans 2
5 Spectrometer Frequency 376.52
6 Nucleus 19F
CF3
N
9
2b
f1 (ppm)

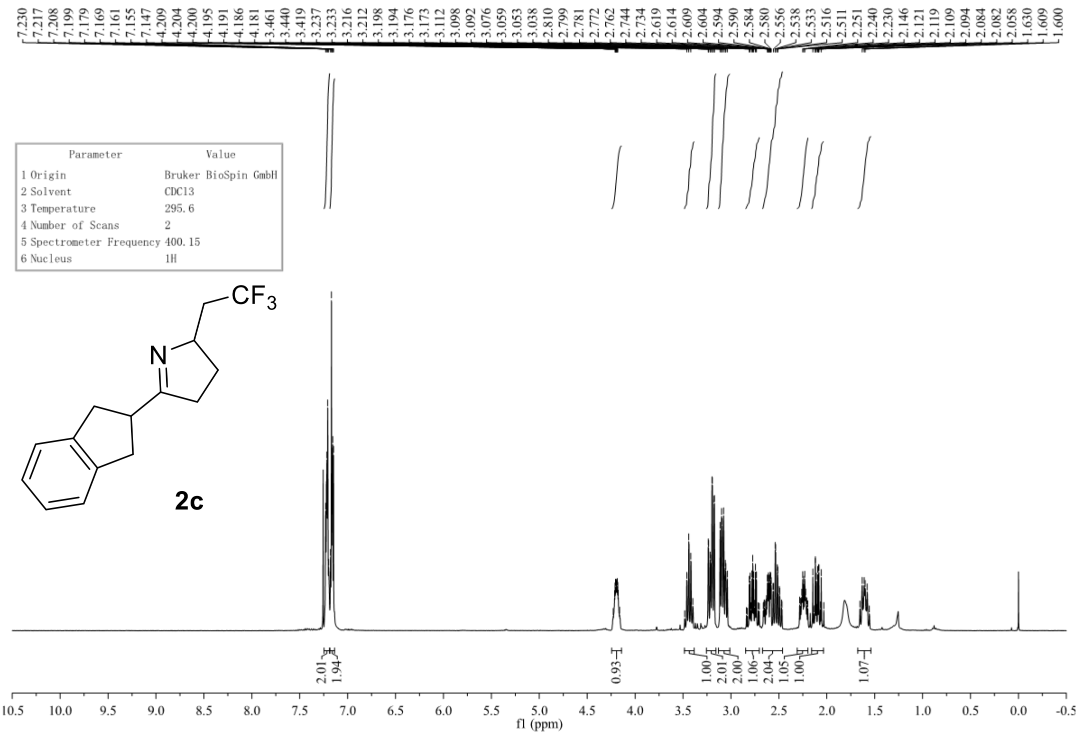
7.230 7.217 7.208 7.199 7.179 7.169 7.161 7.155 7.147 4.209 4.204 4.200 4.195 4.191 4.186 4.181 3.461 3.440 3.419 3.237 3.233 3.216 3.212 3.198 3.194 3.176 3.173 3.112 3.098 3.092 3.076 3.059 3.053 3.038 2.810 2.799 2.781 2.772 2.762 2.744 2.734 2.619 2.614 2.609 2.604 2.594 2.590 2.584 2.580 2.556 2.538 2.533 2.516 2.511 2.251 2.240 2.230 2.146 2.121 2.119 2.109 2.094 2.084 2.082 2.058 1.630 1.609 1.600
Parameter Value
1 Origin Bruker BioSpin GmbH
2 Solvent CDCl3
3 Temperature 295.6
4 Number of Scans 2
5 Spectrometer Frequency 400.15
6 Nucleus 1H
CF3
N
2c
2.01 1.94 0.93 1.00 2.01 2.00 1.06 2.04 1.05 1.00 1.07
f1 (ppm)

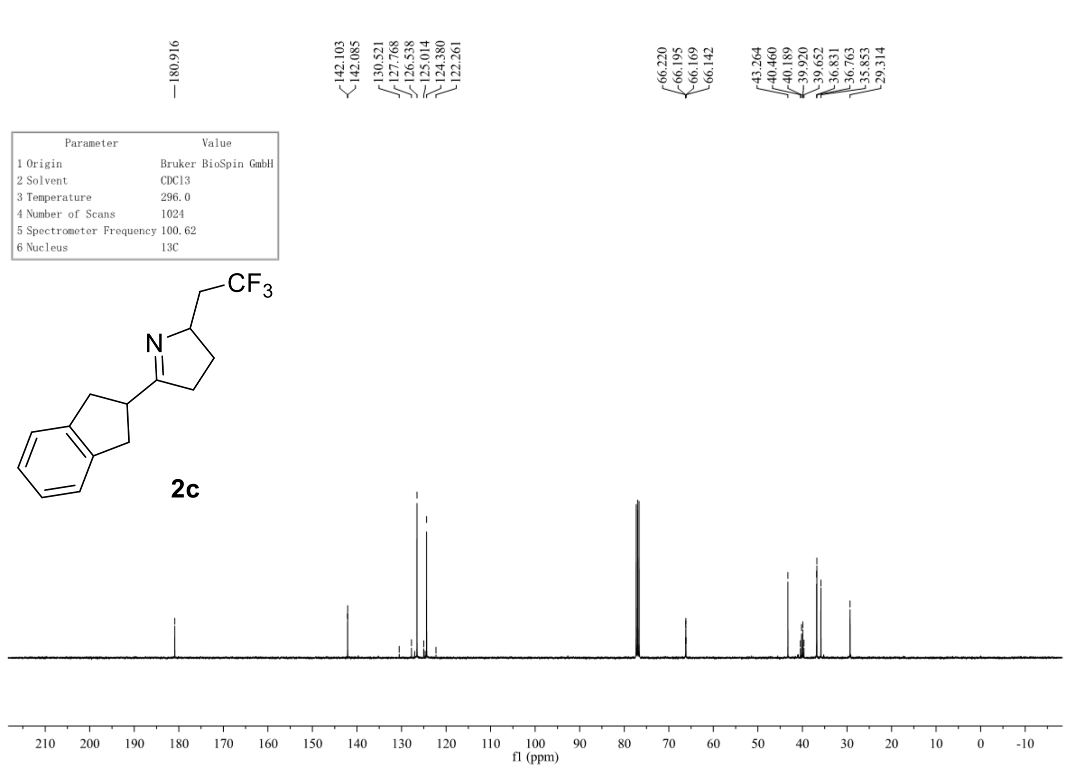
180.916
142.103 142.085 130.521 127.768 126.538 125.014 124.380 122.261
66.220 66.195 66.169 66.142
43.264 40.460 40.189 39.920 39.652 36.831 36.763 35.853 29.314
Parameter Value
1 Origin Bruker BioSpin GmbH
2 Solvent CDCl3
3 Temperature 296.0
4 Number of Scans 1024
5 Spectrometer Frequency 100.62
6 Nucleus 13C
CF3
N
2c
f1 (ppm)

Parameter	Value
1 Origin	Bruker BioSpin GmbH
2 Solvent	CDCl3
3 Temperature	295.7
4 Number of Scans	2
5 Spectrometer Frequency	376.52
6 Nucleus	19F

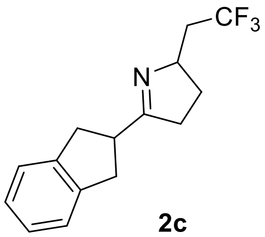

2c

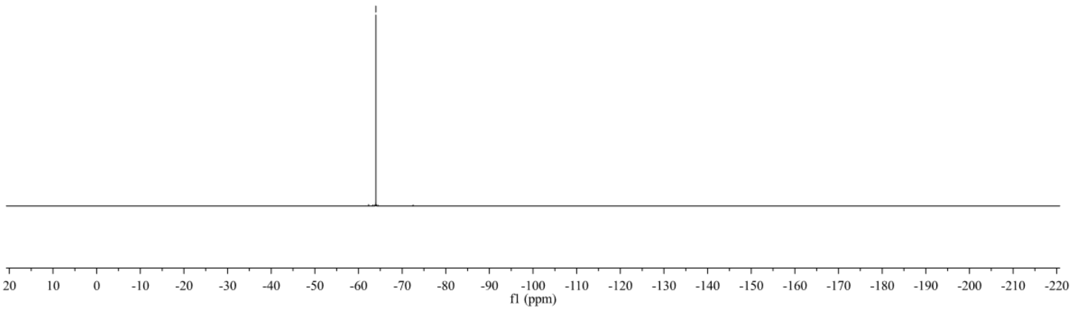

Parameter	Value
1 Origin	Bruker BioSpin GmbH
2 Solvent	CDCl3
3 Temperature	298.2
4 Number of Scans	2
5 Spectrometer Frequency	400.15
6 Nucleus	1H

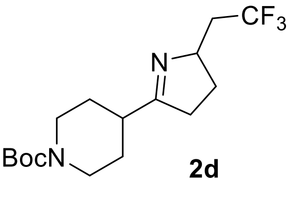

2d

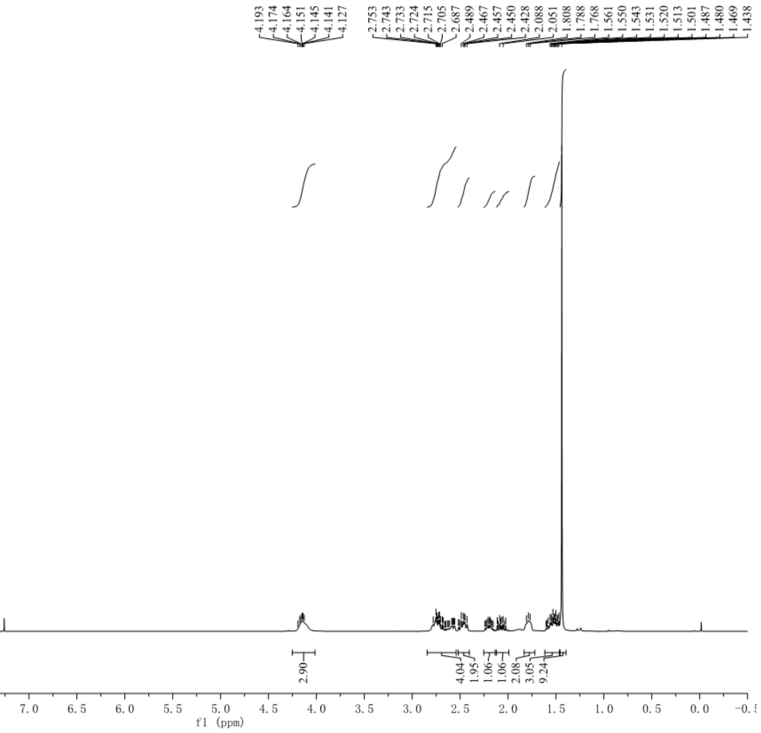

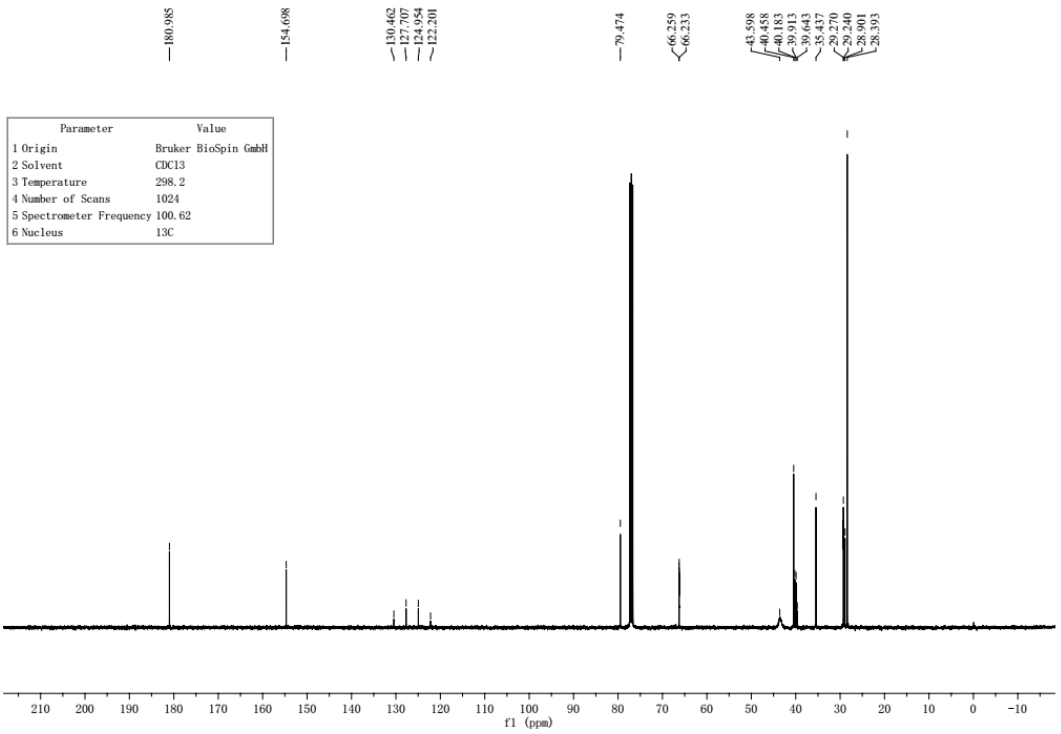
180.985
154.698
130.462
127.707
124.954
122.201
79.474
66.259
66.233
43.598
40.458
40.183
39.913
39.643
35.437
29.270
29.240
28.901
28.393
Parameter Value
1 Origin Bruker BioSpin GmbH
2 Solvent CDCl3
3 Temperature 298.2
4 Number of Scans 1024
5 Spectrometer Frequency 100.62
6 Nucleus 13C
f1 (ppm)

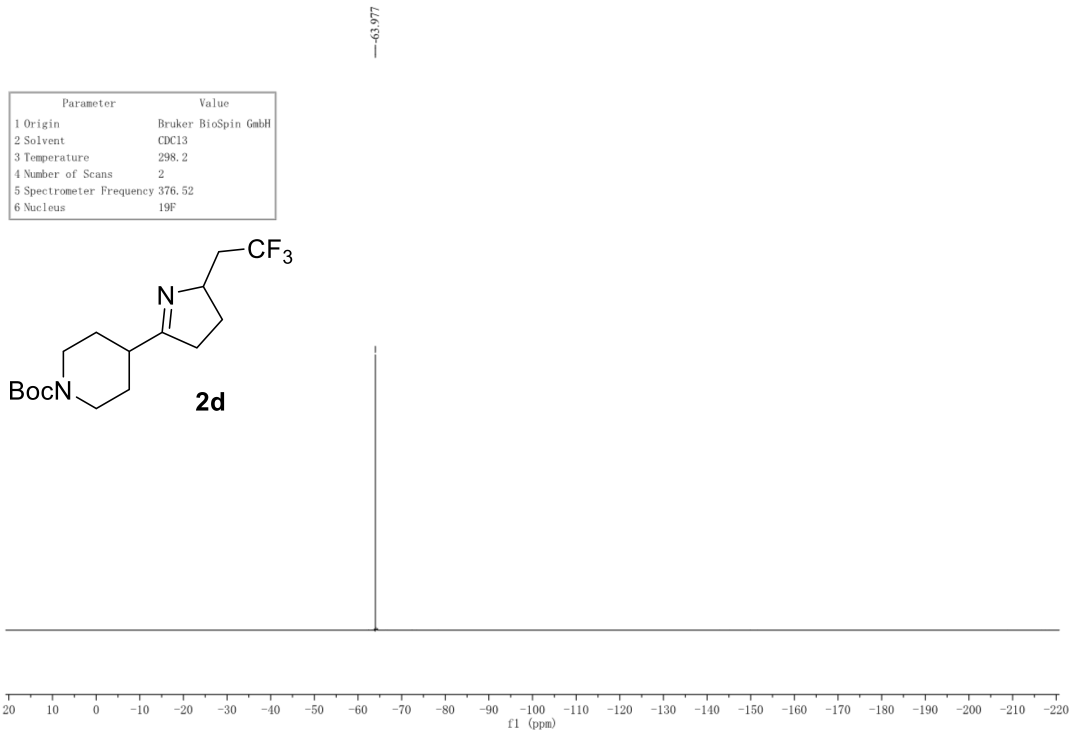
-63.977
Parameter Value
1 Origin Bruker BioSpin GmbH
2 Solvent CDCl3
3 Temperature 298.2
4 Number of Scans 2
5 Spectrometer Frequency 376.52
6 Nucleus 19F
CF3
N
BocN
f1 (ppm)

2d

Parameter	Value
1 Origin	Bruker BioSpin GmbH
2 Solvent	CDCl3
3 Temperature	297.5
4 Number of Scans	2
5 Spectrometer Frequency	400.15
6 Nucleus	1H

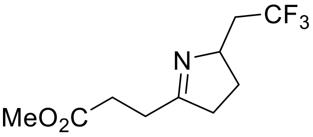

2e

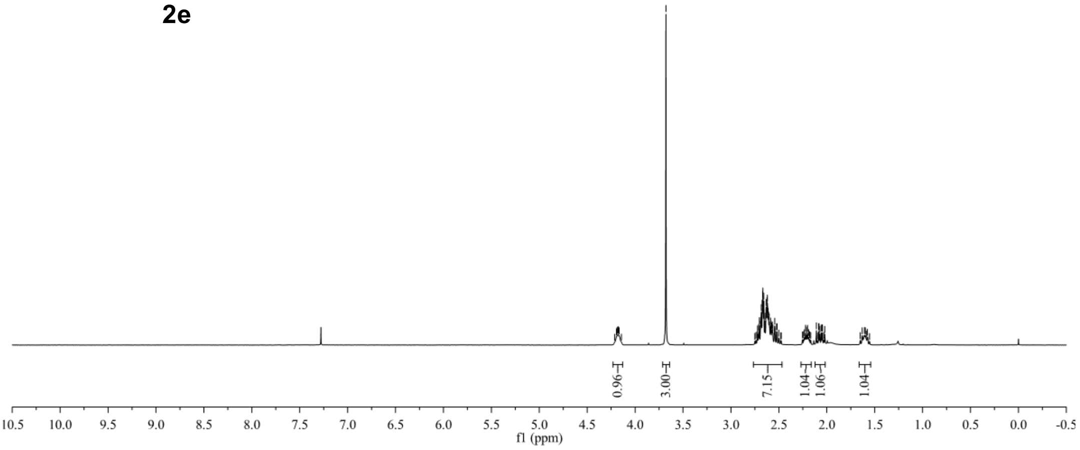

Parameter	Value
1 Origin	Bruker BioSpin GmbH
2 Solvent	CDCl3
3 Temperature	298.0
4 Number of Scans	1024
5 Spectrometer Frequency	100.62
6 Nucleus	13C

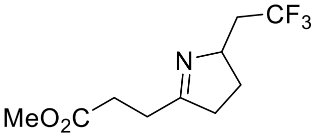

2e

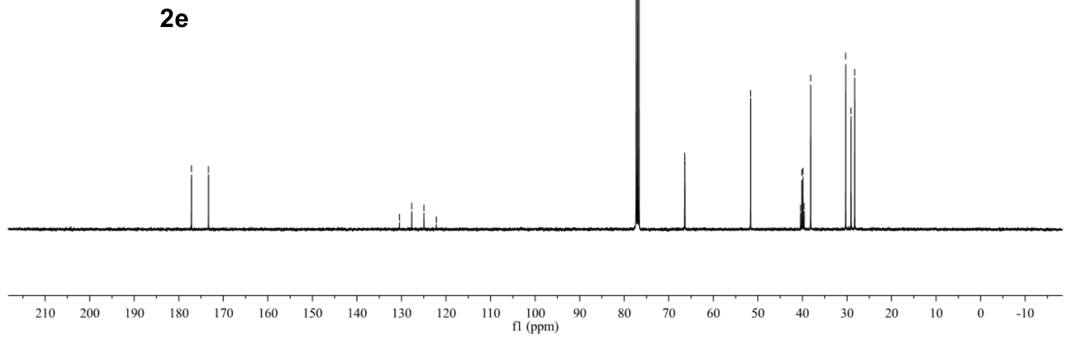

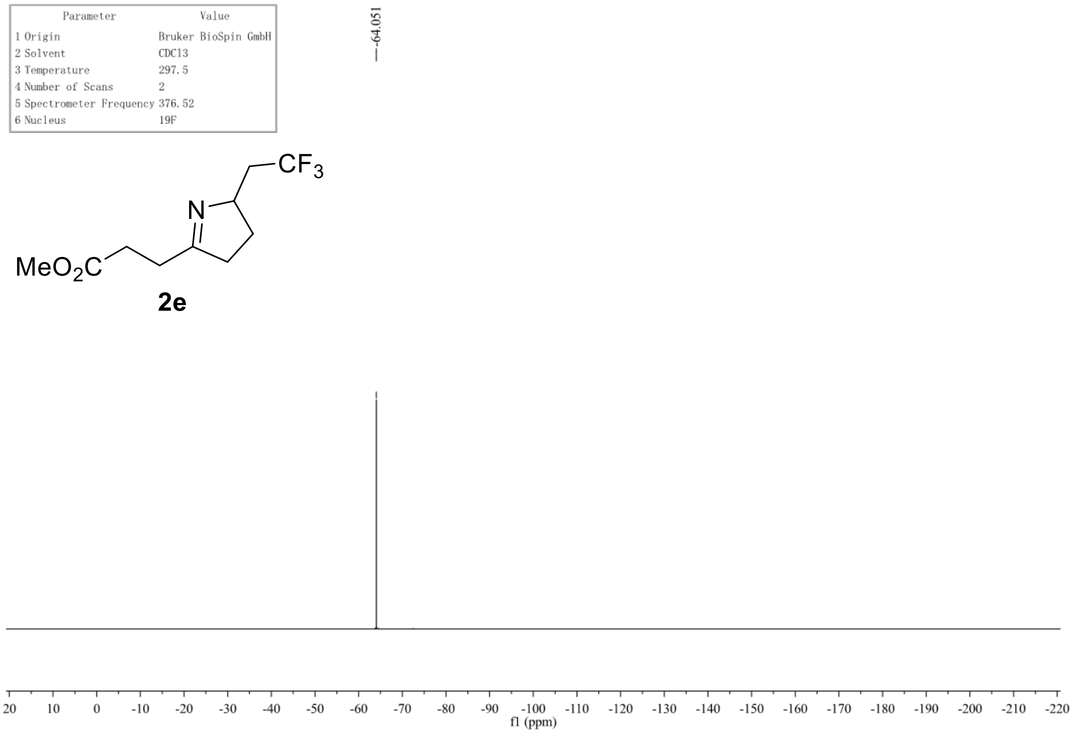

Parameter Value
1 Origin Bruker BioSpin GmbH
2 Solvent CDCl3
3 Temperature 297.5
4 Number of Scans 2
5 Spectrometer Frequency 376.52
6 Nucleus 19F
-64.051
CF3
N
MeO2C
2e
fl (ppm)

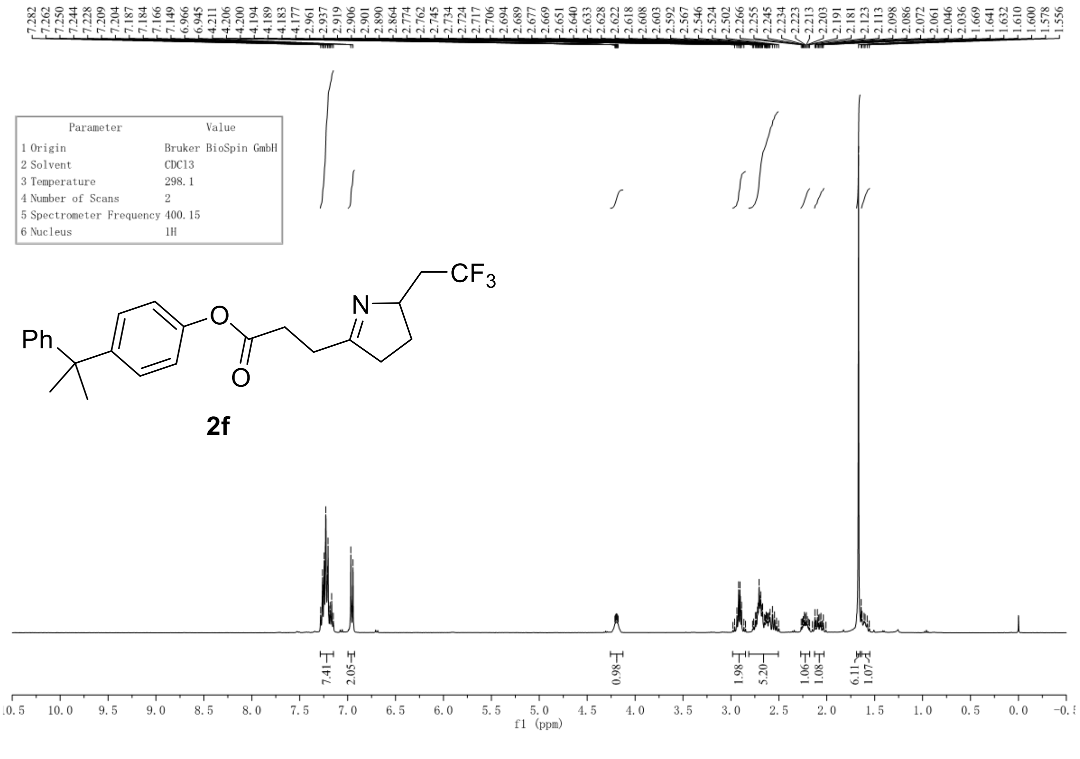

Parameter Value
1 Origin Bruker BioSpin GmbH
2 Solvent CDCl3
3 Temperature 298.1
4 Number of Scans 2
5 Spectrometer Frequency 400.15
6 Nucleus 1H
CF3
N
O
Ph
O
2f
7.41
2.05
0.98
1.98
5.20
1.06
1.08
6.11
1.07
f1 (ppm)

Parameter	Value
1 Origin	Bruker BioSpin GmbH
2 Solvent	CDCl3
3 Temperature	298.2
4 Number of Scans	1024
5 Spectrometer Frequency	100.61
6 Nucleus	13C

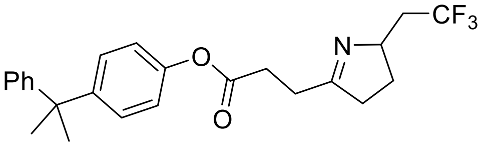

2f

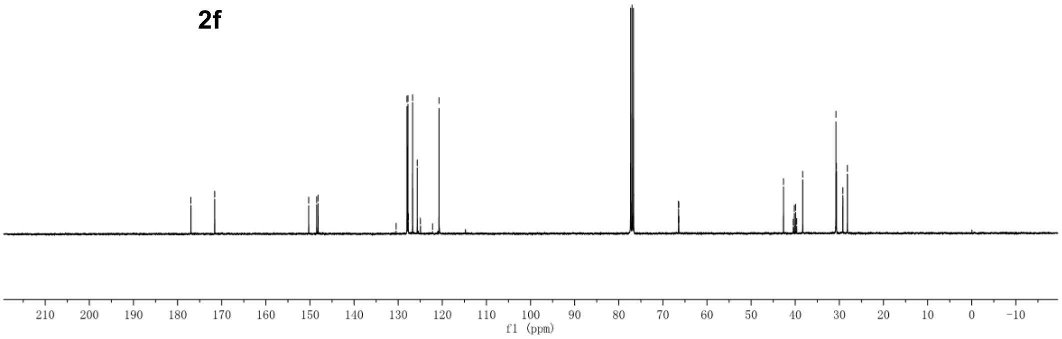

Parameter	Value
1 Origin	Bruker BioSpin GmbH
2 Solvent	CDCl3
3 Temperature	298.2
4 Number of Scans	2
5 Spectrometer Frequency	376.52
6 Nucleus	19F

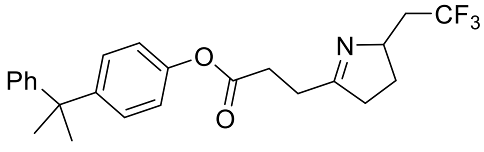

2f

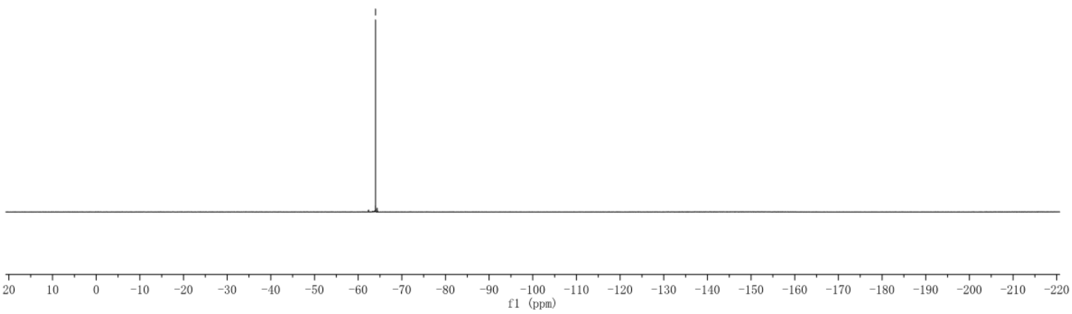

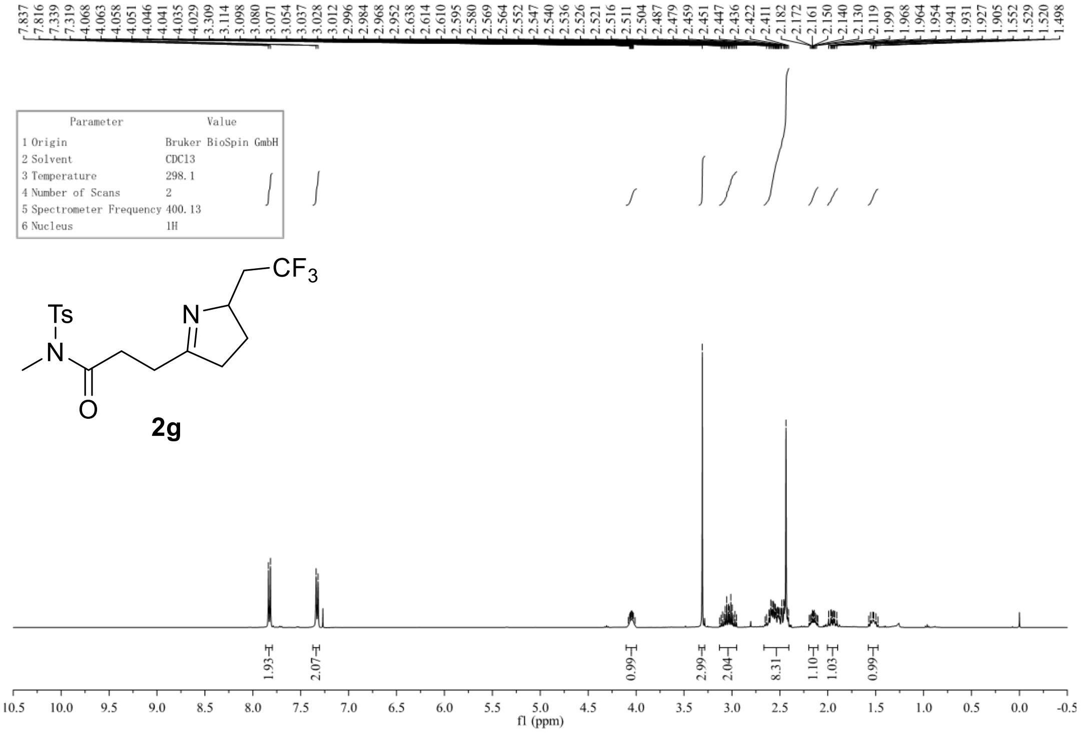
7.837
7.816
7.339
7.319
4.068
4.063
4.058
4.051
4.046
4.041
4.035
4.029
3.309
3.114
3.098
3.080
3.071
3.054
3.037
3.028
3.012
2.996
2.984
2.968
2.952
2.638
2.614
2.610
2.595
2.580
2.569
2.564
2.552
2.547
2.540
2.536
2.526
2.521
2.516
2.511
2.504
2.487
2.479
2.459
2.451
2.447
2.436
2.422
2.411
2.182
2.172
2.161
2.150
2.140
2.130
2.119
1.991
1.968
1.964
1.954
1.941
1.931
1.927
1.905
1.552
1.529
1.520
1.498
Parameter Value
1 Origin Bruker BioSpin GmbH
2 Solvent CDCl3
3 Temperature 298.1
4 Number of Scans 2
5 Spectrometer Frequency 400.13
6 Nucleus 1H
CF3
Ts
N
N
O
2g
1.93
2.07
0.99
2.99
2.04
8.31
1.10
1.03
0.99
10.5 10.0 9.5 9.0 8.5 8.0 7.5 7.0 6.5 6.0 5.5 5.0 4.5 4.0 3.5 3.0 2.5 2.0 1.5 1.0 0.5 0.0 -0.5
f1 (ppm)

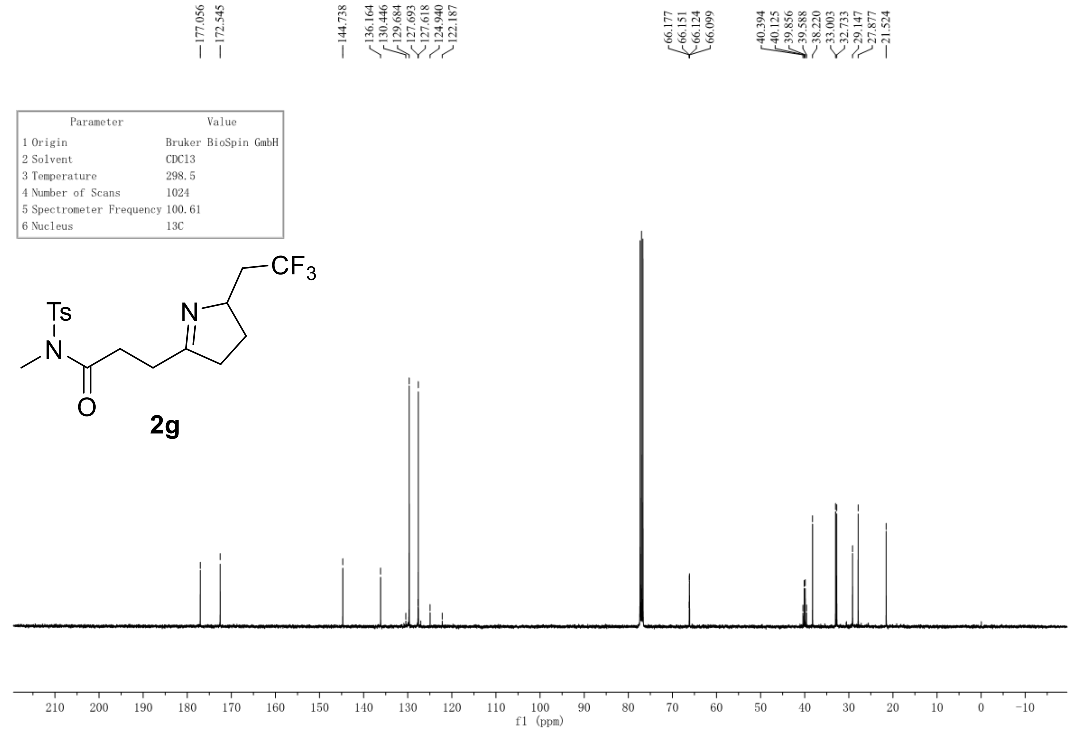
177.056
172.545
144.738
136.164
130.446
129.684
127.693
127.618
124.940
122.187
66.177
66.151
66.124
66.099
40.394
40.125
39.856
39.588
38.220
33.003
32.733
29.147
27.877
21.524
Parameter Value
1 Origin Bruker BioSpin GmbH
2 Solvent CDCl3
3 Temperature 298.5
4 Number of Scans 1024
5 Spectrometer Frequency 100.61
6 Nucleus 13C
CF3
Ts
N
N
O
2g
210 200 190 180 170 160 150 140 130 120 110 100 90 80 70 60 50 40 30 20 10 0 -10
f1 (ppm)

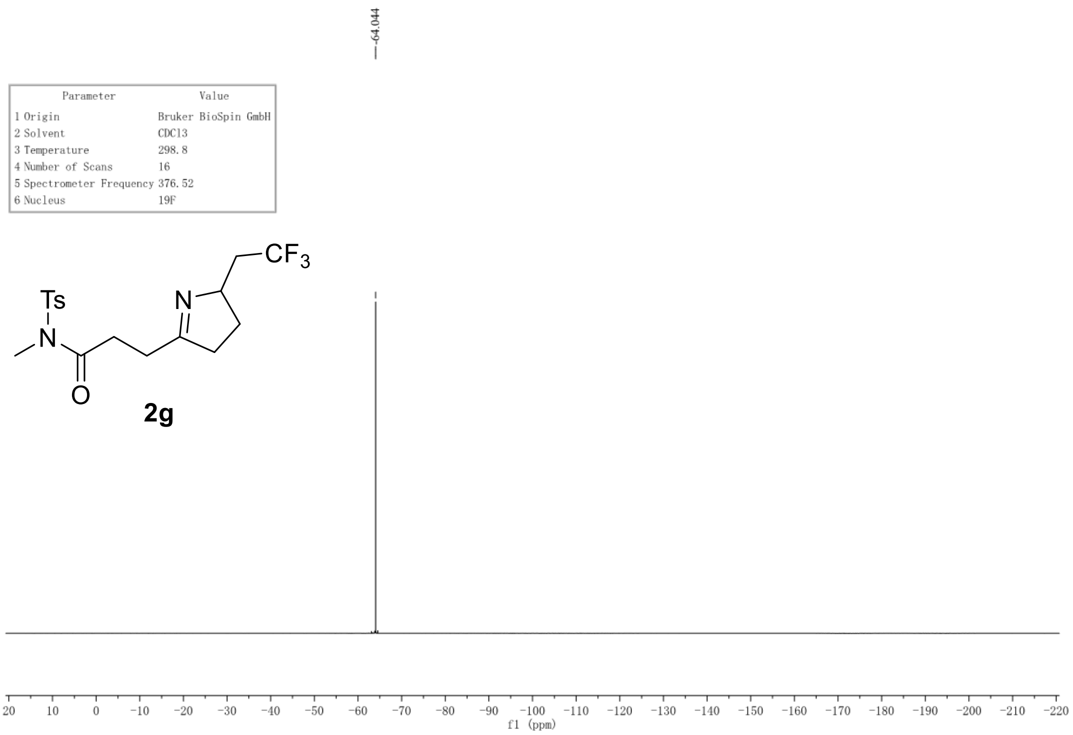

-64.044
Parameter Value
1 Origin Bruker BioSpin GmbH
2 Solvent CDCl3
3 Temperature 298.8
4 Number of Scans 16
5 Spectrometer Frequency 376.52
6 Nucleus 19F
Ts
N
O
N
CF3
2g
20 10 0 -10 -20 -30 -40 -50 -60 -70 -80 -90 -100 -110 -120 -130 -140 -150 -160 -170 -180 -190 -200 -210 -220
f1 (ppm)

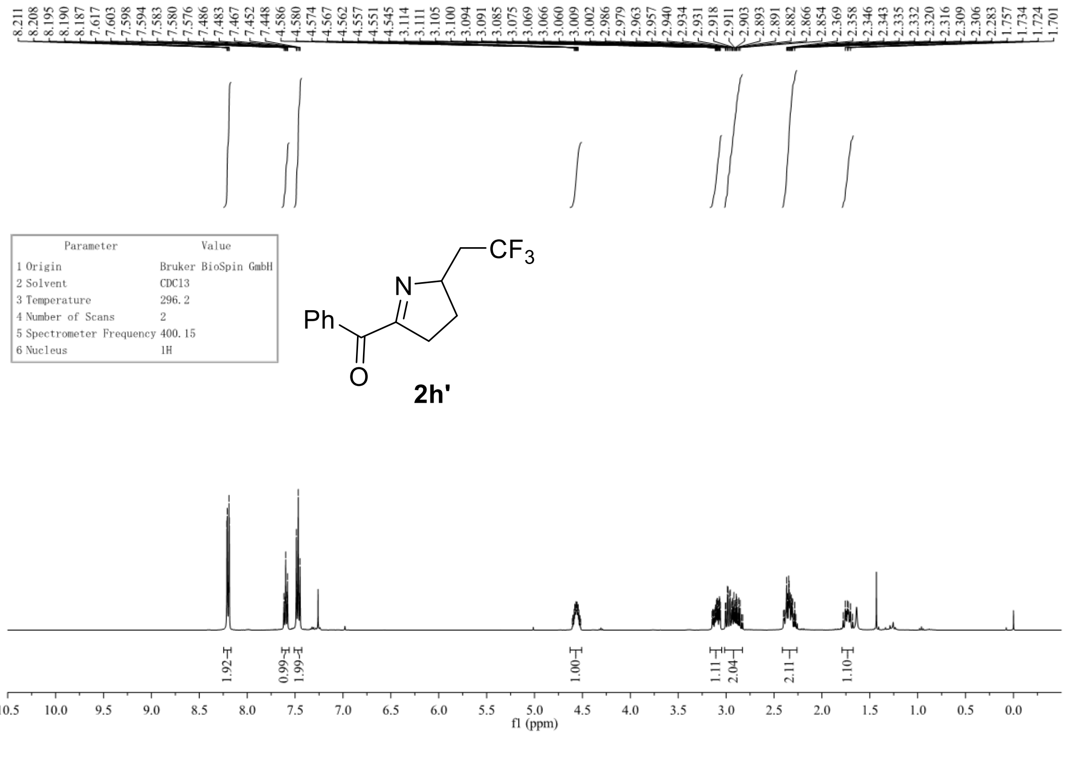

8.211 8.208 8.195 8.190 8.187 7.617 7.603 7.598 7.594 7.583 7.580 7.576 7.486 7.483 7.467 7.452 7.448 4.586 4.580 4.574 4.567 4.562 4.557 4.551 4.545 3.114 3.111 3.105 3.100 3.094 3.091 3.085 3.075 3.069 3.066 3.060 3.009 3.002 2.986 2.979 2.963 2.957 2.940 2.934 2.931 2.918 2.911 2.903 2.893 2.891 2.882 2.866 2.854 2.369 2.358 2.346 2.343 2.335 2.332 2.320 2.316 2.309 2.306 2.283 1.757 1.734 1.724 1.701
Parameter Value
1 Origin Bruker BioSpin GmbH
2 Solvent CDCl3
3 Temperature 296.2
4 Number of Scans 2
5 Spectrometer Frequency 400.15
6 Nucleus 1H
Ph
O
N
CF3
2h'
1.92 0.99 1.99 1.00 1.11 2.04 2.11 1.10
10.5 10.0 9.5 9.0 8.5 8.0 7.5 7.0 6.5 6.0 5.5 5.0 4.5 4.0 3.5 3.0 2.5 2.0 1.5 1.0 0.5 0.0
f1 (ppm)

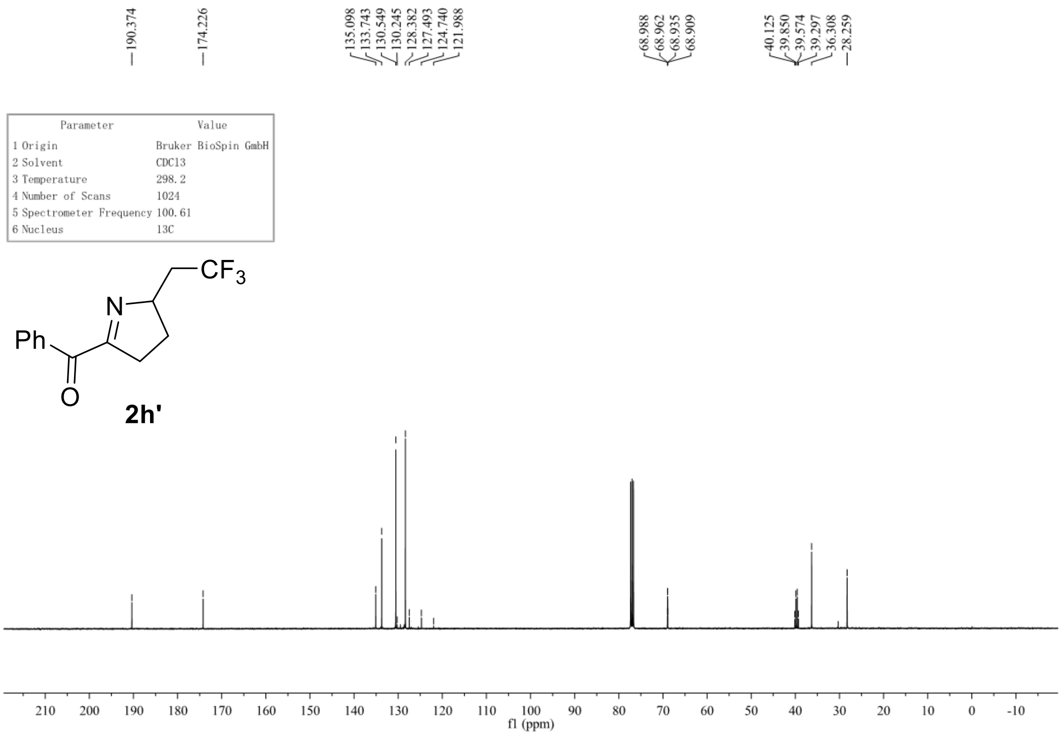
190.374
174.226
135.098
133.743
130.549
130.245
128.382
127.493
124.740
121.988
68.988
68.962
68.935
68.909
40.125
39.850
39.574
39.297
36.308
28.259
Parameter Value
1 Origin Bruker BioSpin GmbH
2 Solvent CDCl3
3 Temperature 298.2
4 Number of Scans 1024
5 Spectrometer Frequency 100.61
6 Nucleus 13C
CF3
N
Ph
O
2h'
fl (ppm)

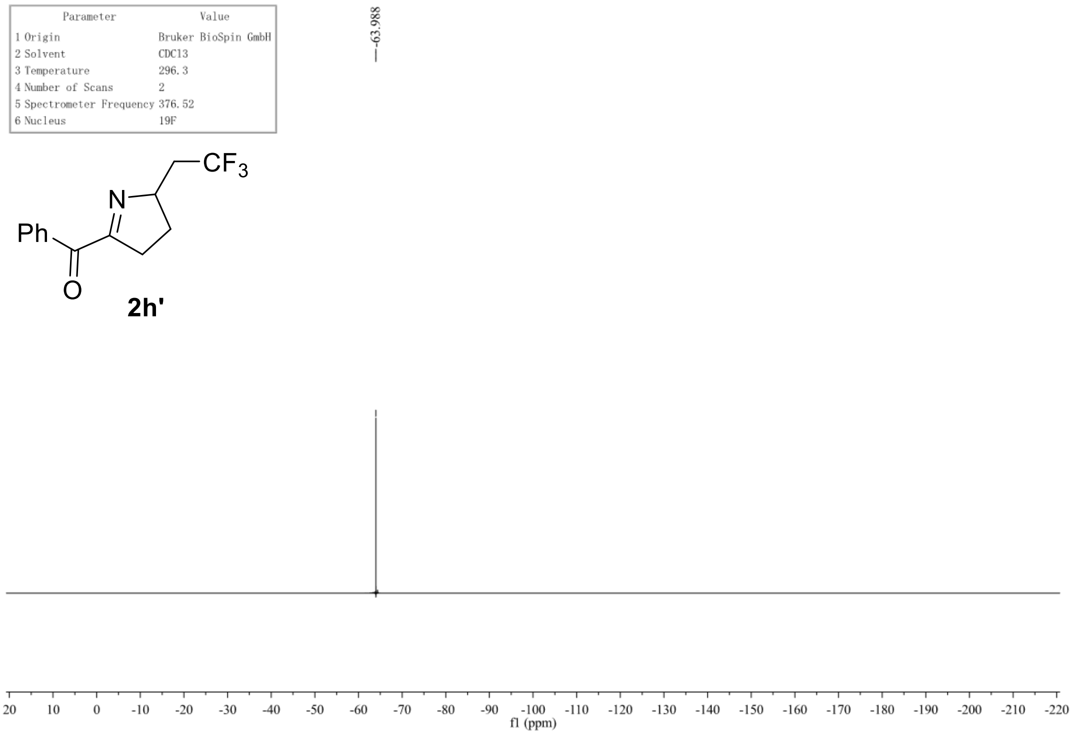
-63.988
Parameter Value
1 Origin Bruker BioSpin GmbH
2 Solvent CDCl3
3 Temperature 296.3
4 Number of Scans 2
5 Spectrometer Frequency 376.52
6 Nucleus 19F
CF3
N
Ph
O
2h'
fl (ppm)

Parameter	Value
1 Origin	Bruker BioSpin GmbH
2 Solvent	CDCl3
3 Temperature	298.1
4 Number of Scans	2
5 Spectrometer Frequency	400.15
6 Nucleus	1H

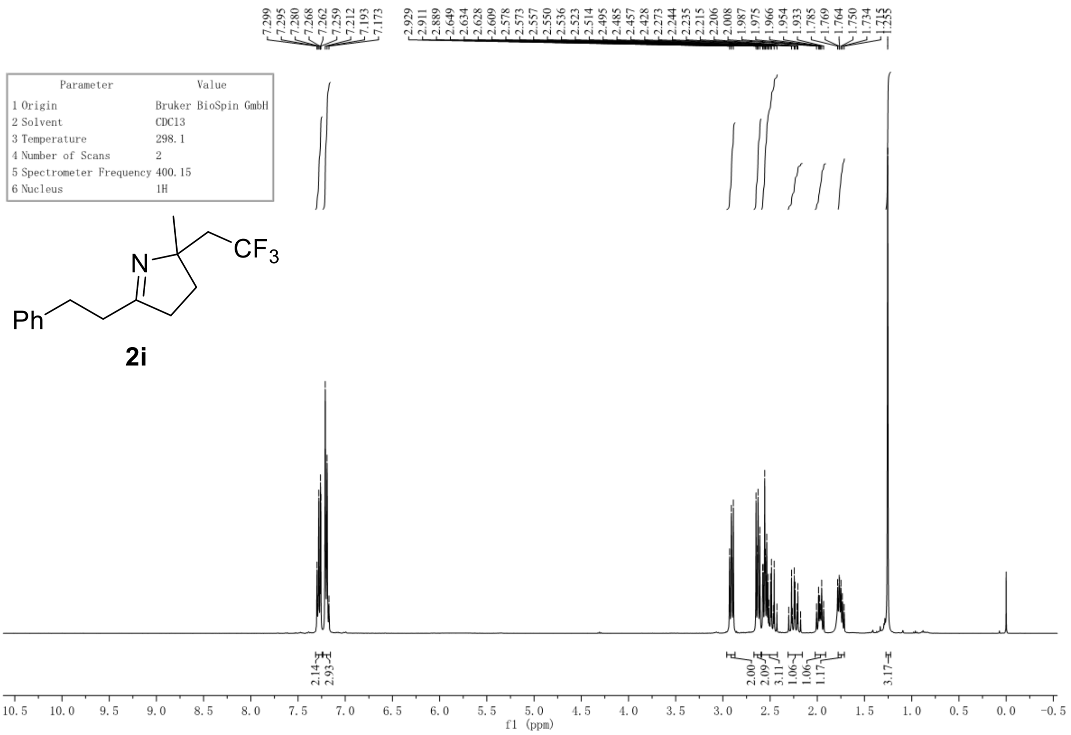

Parameter	Value
1 Origin	Bruker BioSpin GmbH
2 Solvent	CDCl3
3 Temperature	298.1
4 Number of Scans	1024
5 Spectrometer Frequency	100.62
6 Nucleus	13C

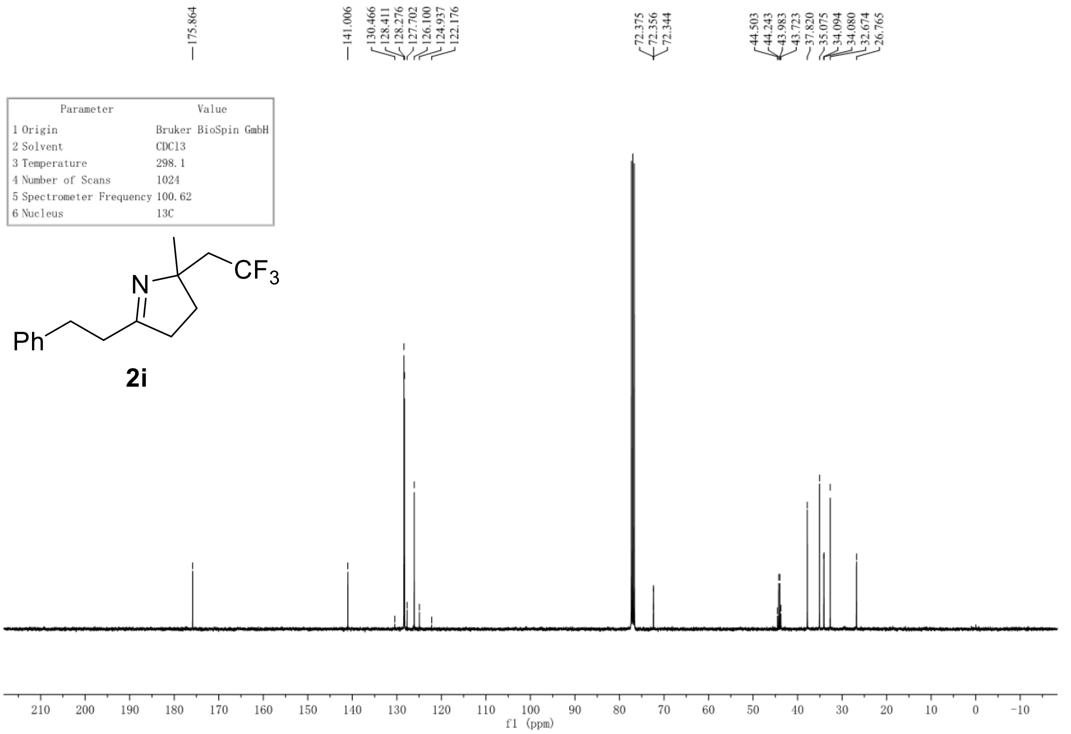

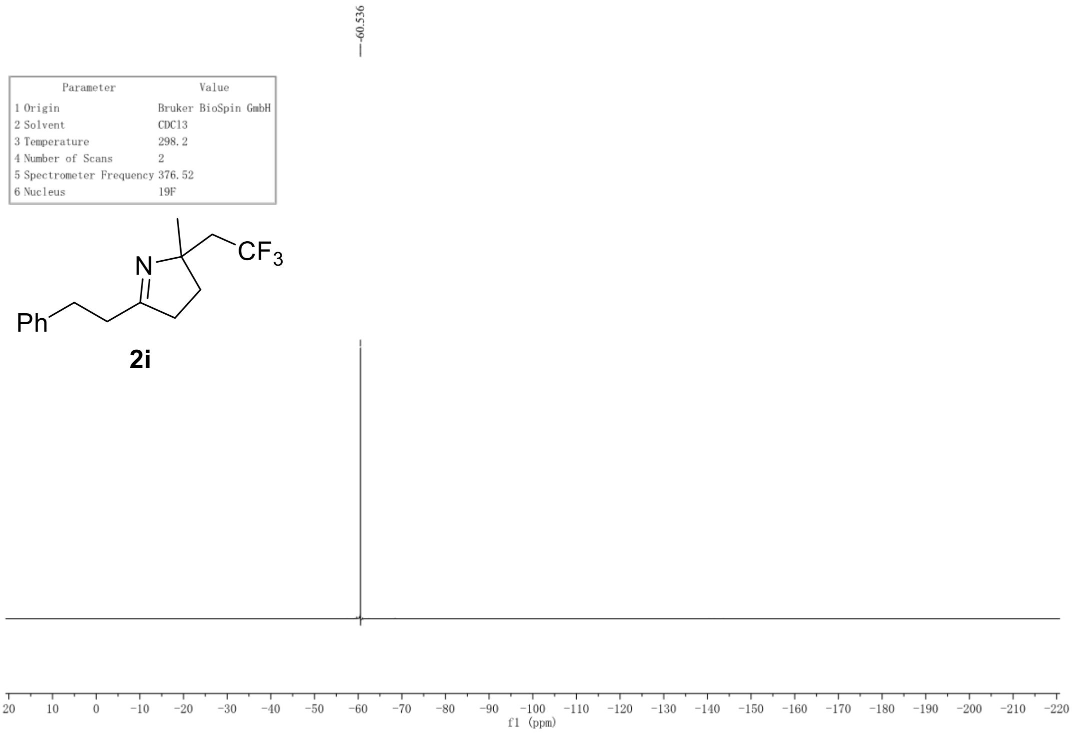

-60.536
Parameter Value
1 Origin Bruker BioSpin GmbH
2 Solvent CDCl3
3 Temperature 298.2
4 Number of Scans 2
5 Spectrometer Frequency 376.52
6 Nucleus 19F
N
CF3
Ph
2i
20 10 0 -10 -20 -30 -40 -50 -60 -70 -80 -90 -100 -110 -120 -130 -140 -150 -160 -170 -180 -190 -200 -210 -220
f1 (ppm)

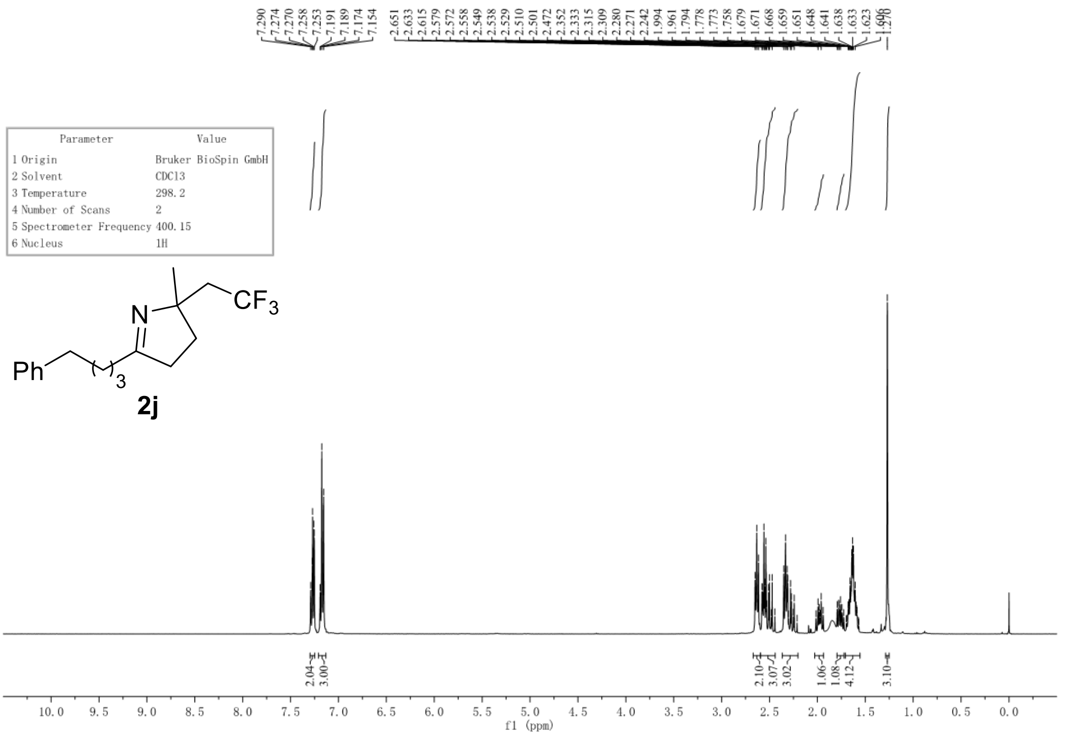

7.290 7.274 7.270 7.258 7.253 7.191 7.189 7.174 7.154
2.651 2.633 2.615 2.579 2.572 2.558 2.549 2.538 2.529 2.510 2.501 2.472 2.352 2.333 2.315 2.309 2.280 2.271 2.242 1.994 1.961 1.794 1.778 1.773 1.758 1.679 1.671 1.668 1.659 1.651 1.648 1.641 1.638 1.633 1.623 1.270
Parameter Value
1 Origin Bruker BioSpin GmbH
2 Solvent CDCl3
3 Temperature 298.2
4 Number of Scans 2
5 Spectrometer Frequency 400.15
6 Nucleus 1H
N
CF3
Ph
3
2j
2.04 3.00 2.10 3.07 3.02 1.06 1.08 4.12 3.10
10.0 9.5 9.0 8.5 8.0 7.5 7.0 6.5 6.0 5.5 5.0 4.5 4.0 3.5 3.0 2.5 2.0 1.5 1.0 0.5 0.0
f1 (ppm)

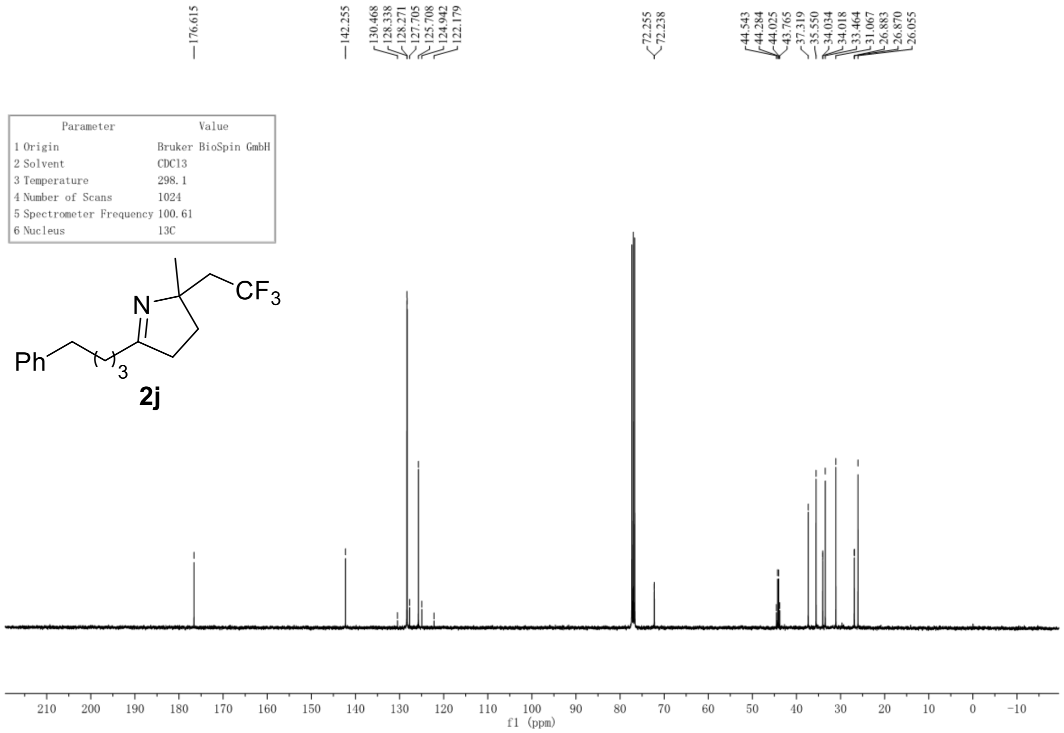
176.615
142.255
130.468
128.338
128.271
127.705
125.708
124.942
122.179
72.255
72.238
44.543
44.284
44.025
43.765
37.319
35.550
34.034
34.018
33.464
31.067
26.883
26.870
26.055
Parameter Value
1 Origin Bruker BioSpin GmbH
2 Solvent CDCl3
3 Temperature 298.1
4 Number of Scans 1024
5 Spectrometer Frequency 100.61
6 Nucleus 13C
N
CF3
Ph
3
2j
f1 (ppm)

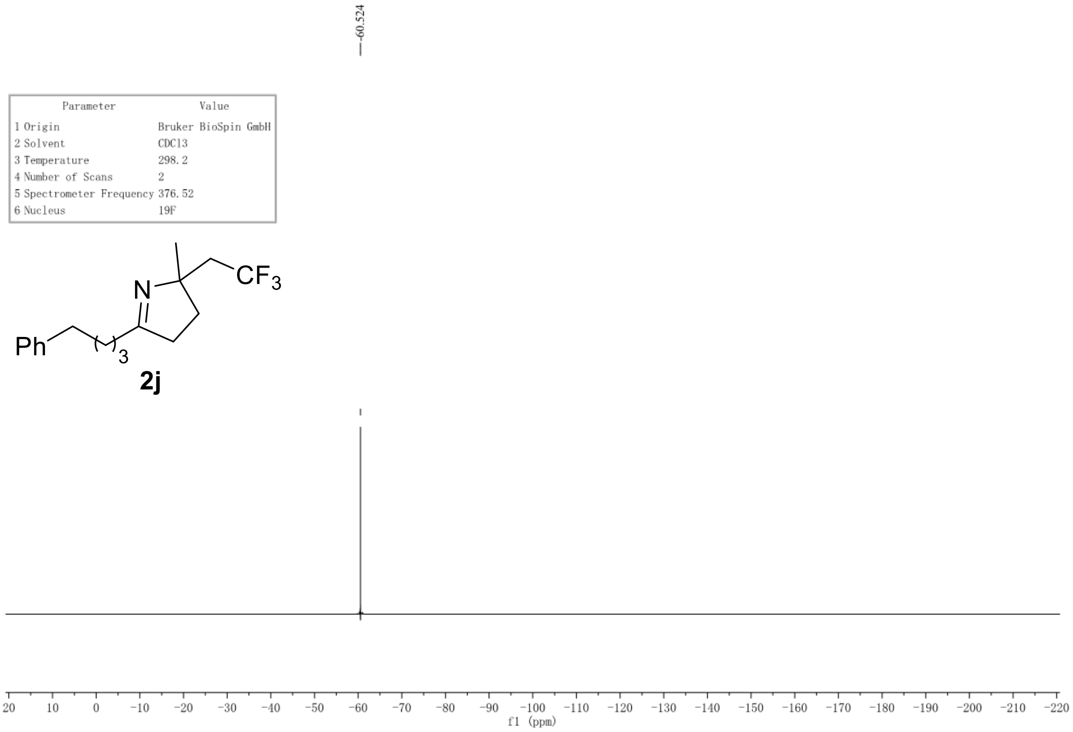
-60.524
Parameter Value
1 Origin Bruker BioSpin GmbH
2 Solvent CDCl3
3 Temperature 298.2
4 Number of Scans 2
5 Spectrometer Frequency 376.52
6 Nucleus 19F
N
CF3
Ph
3
2j
f1 (ppm)

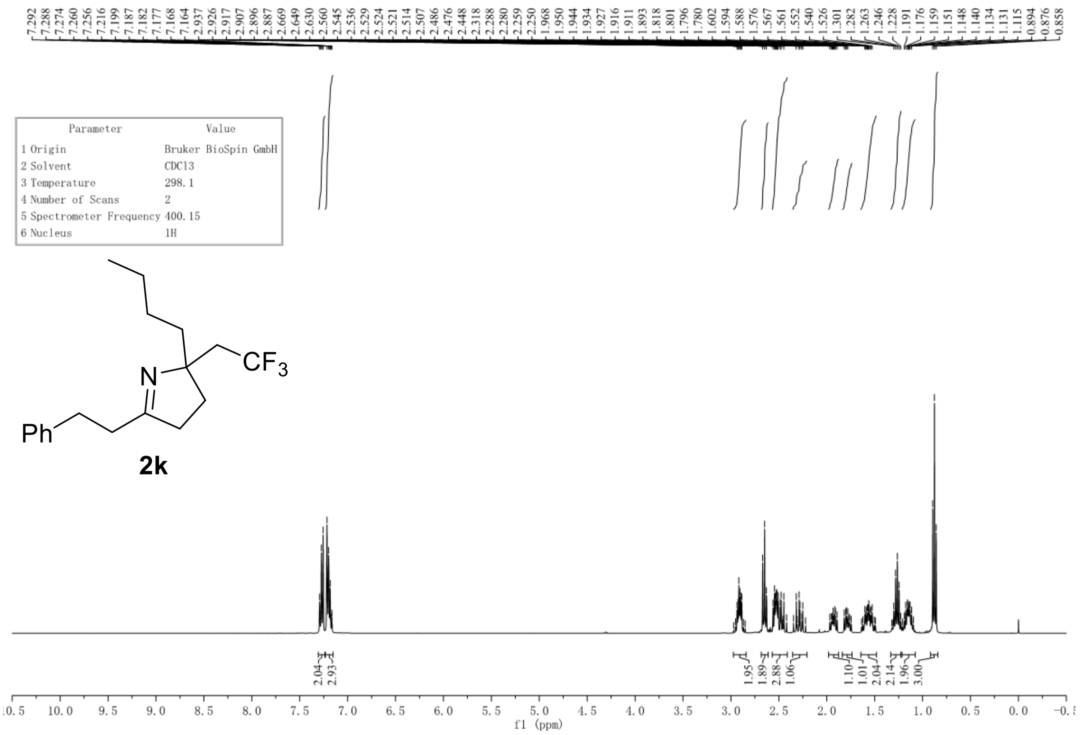

Parameter	Value
1 Origin	Bruker BioSpin GmbH
2 Solvent	CDCl3
3 Temperature	298.1
4 Number of Scans	2
5 Spectrometer Frequency	400.15
6 Nucleus	1H

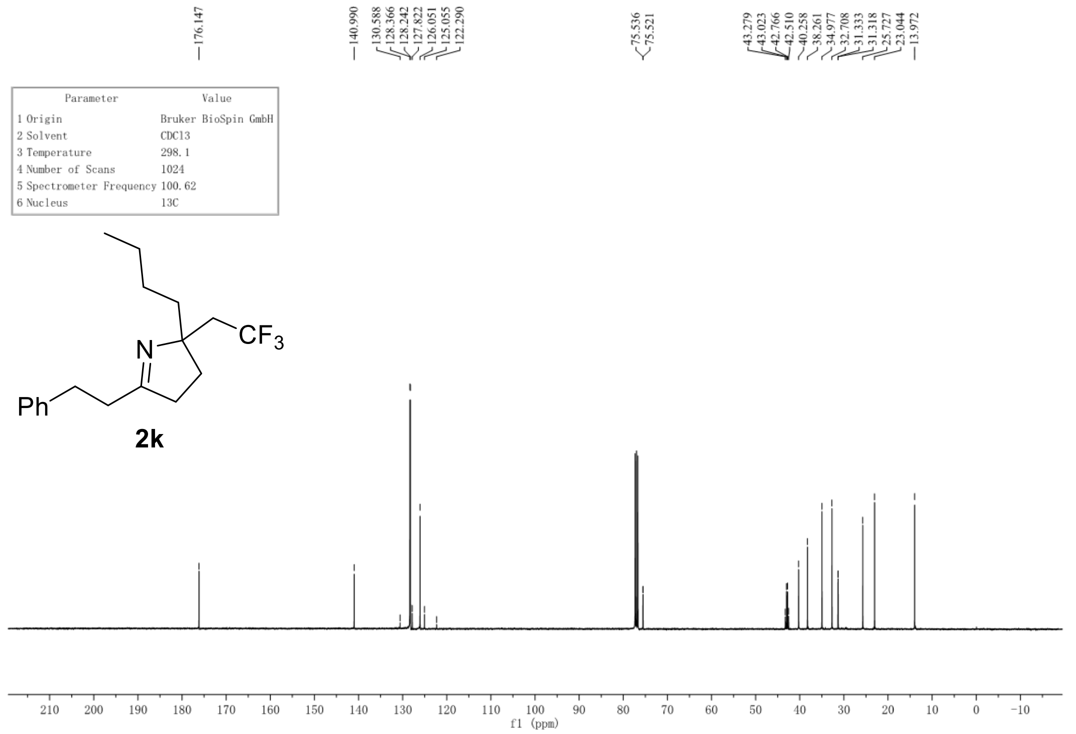

Parameter	Value
1 Origin	Bruker BioSpin GmbH
2 Solvent	CDCl3
3 Temperature	298.1
4 Number of Scans	1024
5 Spectrometer Frequency	100.62
6 Nucleus	13C

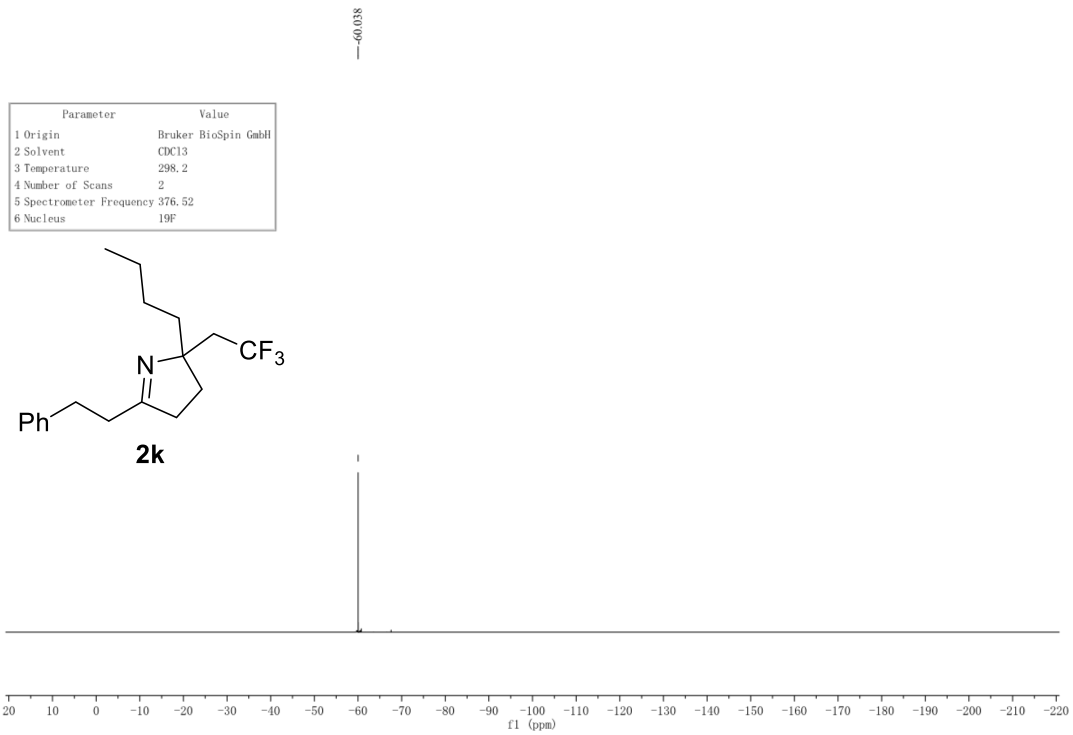
-60.038
Parameter Value
1 Origin Bruker BioSpin GmbH
2 Solvent CDCl3
3 Temperature 298.2
4 Number of Scans 2
5 Spectrometer Frequency 376.52
6 Nucleus 19F
CF3
N
Ph
2k
f1 (ppm)

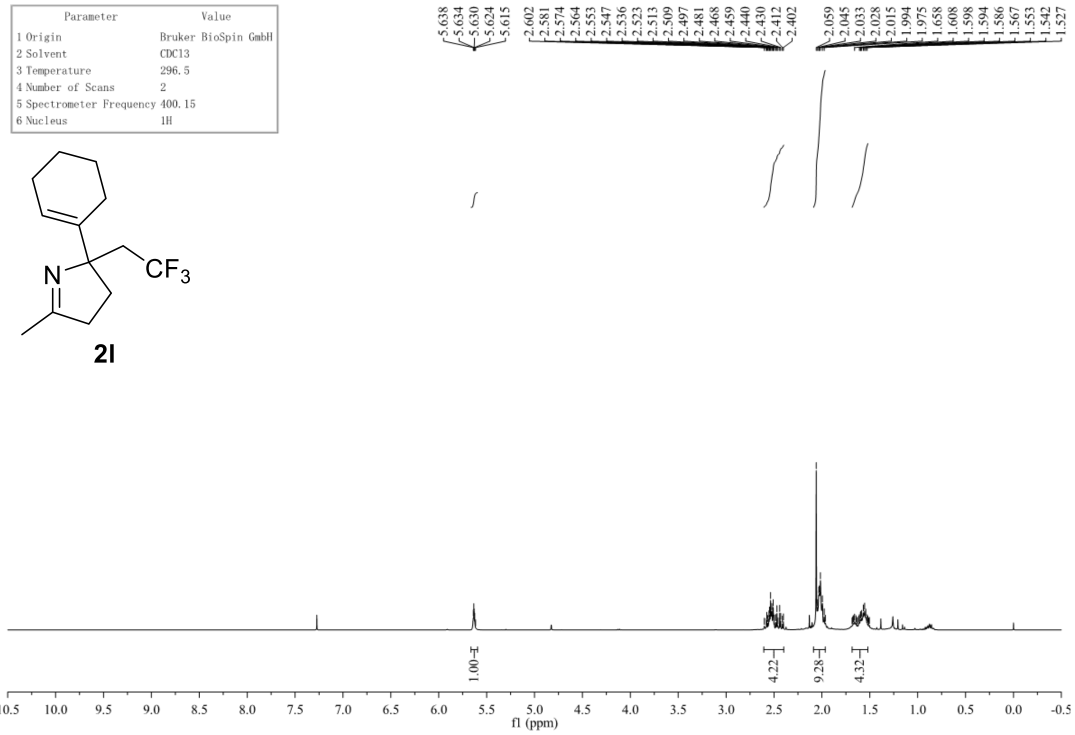
5.638
5.634
5.630
5.624
5.615
2.602
2.581
2.574
2.564
2.553
2.547
2.536
2.523
2.513
2.509
2.497
2.481
2.468
2.459
2.440
2.430
2.412
2.402
2.059
2.045
2.033
2.028
2.015
1.994
1.975
1.658
1.608
1.598
1.594
1.586
1.567
1.553
1.542
1.527
Parameter Value
1 Origin Bruker BioSpin GmbH
2 Solvent CDCl3
3 Temperature 296.5
4 Number of Scans 2
5 Spectrometer Frequency 400.15
6 Nucleus 1H
N
CF3
2l
1.00
4.22
9.28
4.32
f1 (ppm)

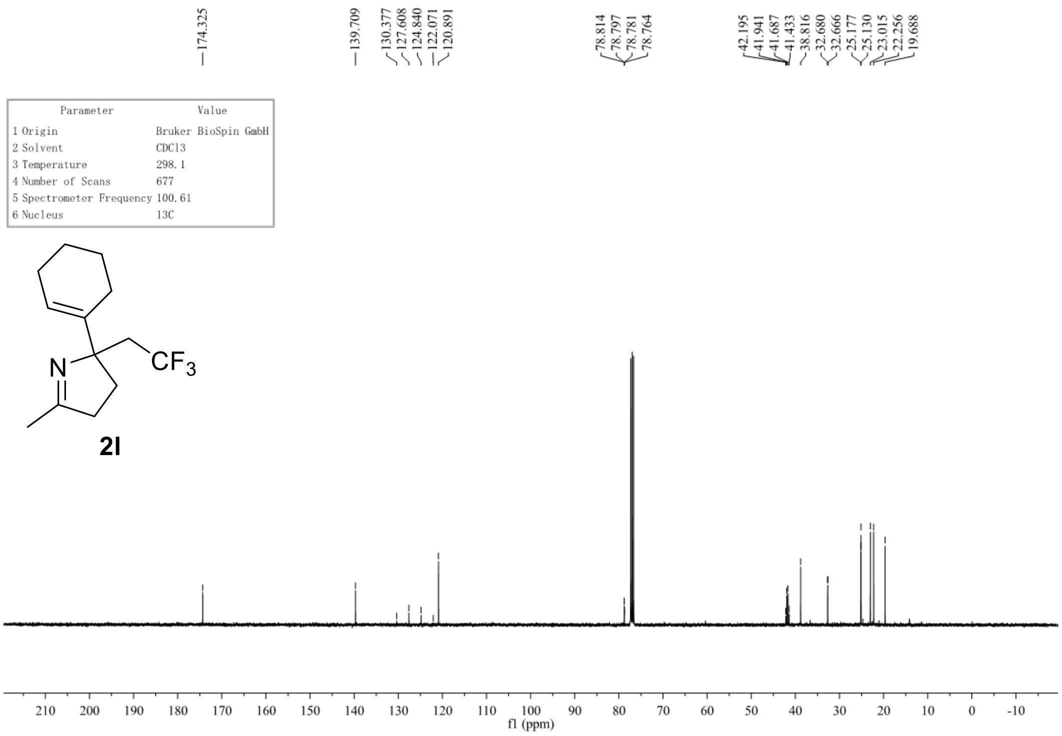

Parameter	Value
1 Origin	Bruker BioSpin GmbH
2 Solvent	CDCl3
3 Temperature	298.1
4 Number of Scans	677
5 Spectrometer Frequency	100.61
6 Nucleus	13C

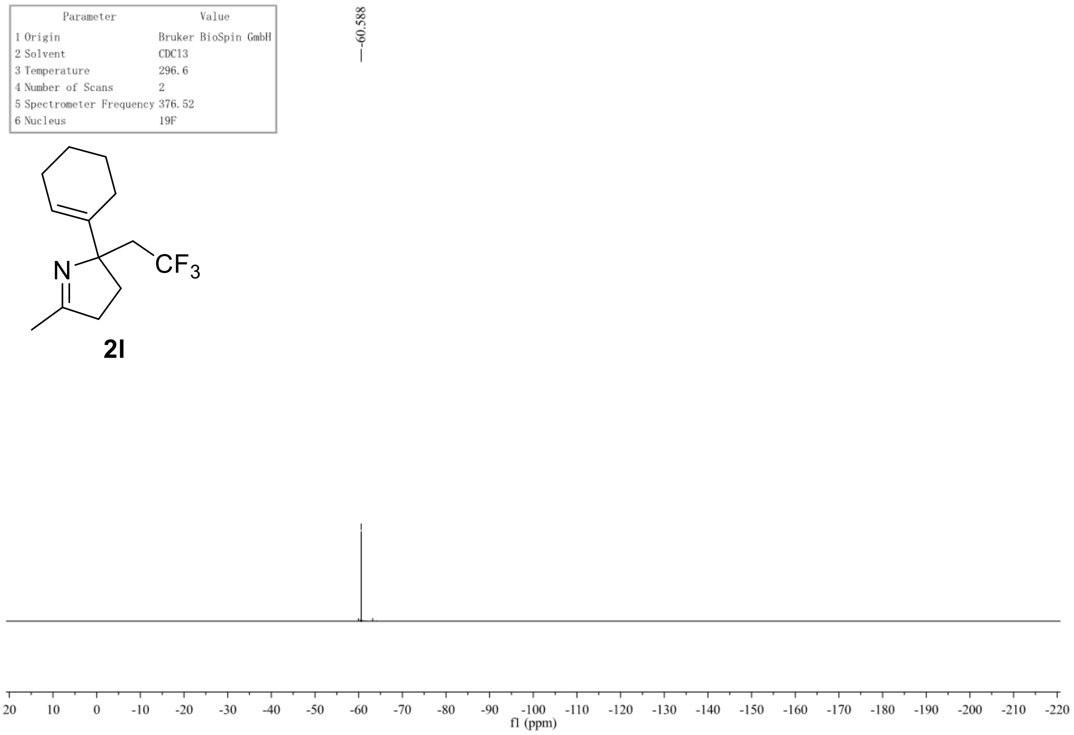

Parameter	Value
1 Origin	Bruker BioSpin GmbH
2 Solvent	CDCl3
3 Temperature	296.6
4 Number of Scans	2
5 Spectrometer Frequency	376.52
6 Nucleus	19F

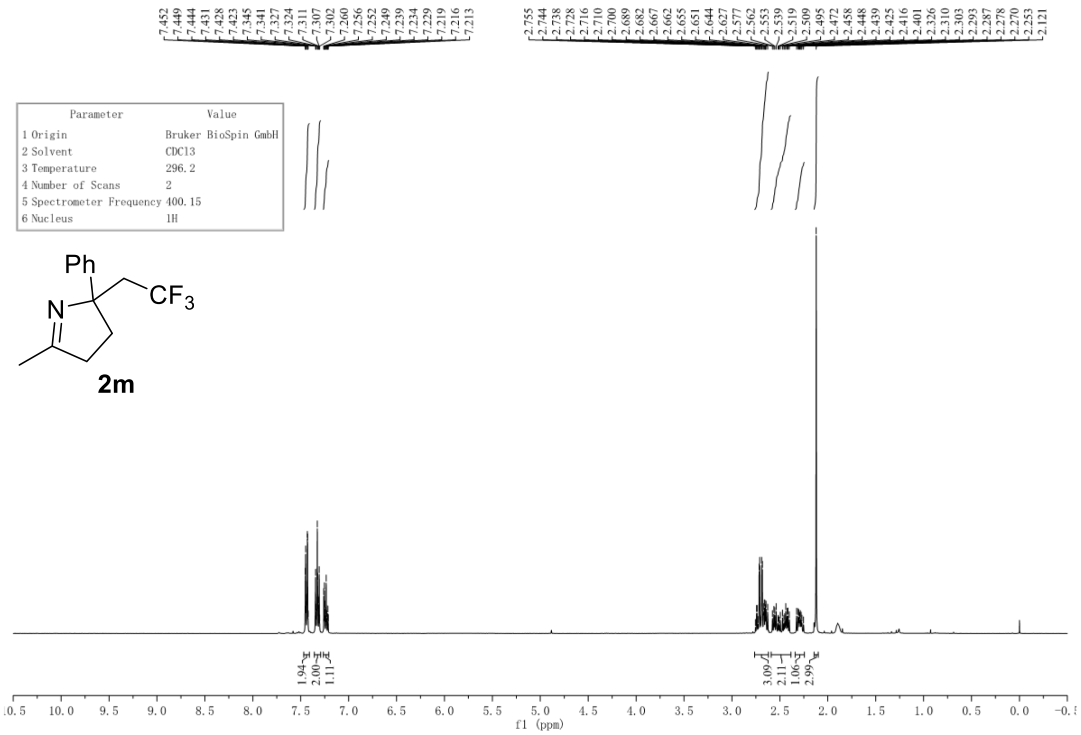
7.452 7.449 7.444 7.431 7.428 7.423 7.345 7.341 7.327 7.324 7.311 7.307 7.302 7.260 7.256 7.252 7.249 7.239 7.234 7.229 7.219 7.216 7.213
2.755 2.744 2.738 2.728 2.716 2.710 2.700 2.689 2.682 2.667 2.662 2.655 2.651 2.644 2.627 2.577 2.562 2.553 2.539 2.519 2.509 2.495 2.472 2.458 2.448 2.439 2.425 2.416 2.401 2.326 2.310 2.303 2.293 2.287 2.278 2.270 2.253 2.121
Parameter Value
1 Origin Bruker BioSpin GmbH
2 Solvent CDCl3
3 Temperature 296.2
4 Number of Scans 2
5 Spectrometer Frequency 400.15
6 Nucleus 1H
Ph
N
CF3
2m
1.94 2.00 1.11
3.09 2.11 1.06 2.99
10.5 10.0 9.5 9.0 8.5 8.0 7.5 7.0 6.5 6.0 5.5 5.0 4.5 4.0 3.5 3.0 2.5 2.0 1.5 1.0 0.5 0.0 -0.5
f1 (ppm)

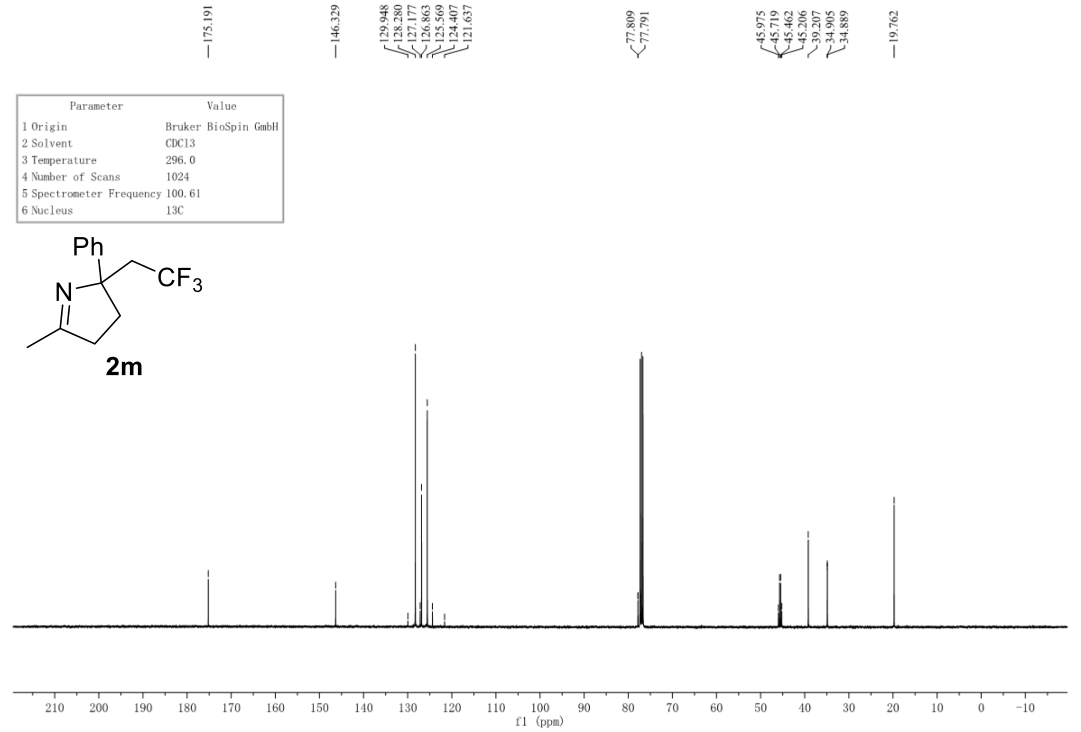
175.191
146.329
129.948 128.280 127.177 126.863 125.569 124.407 121.637
77.809 77.791
45.975 45.719 45.462 45.206 39.207 34.905 34.889
19.762
Parameter Value
1 Origin Bruker BioSpin GmbH
2 Solvent CDCl3
3 Temperature 296.0
4 Number of Scans 1024
5 Spectrometer Frequency 100.61
6 Nucleus 13C
Ph
N
CF3
2m
210 200 190 180 170 160 150 140 130 120 110 100 90 80 70 60 50 40 30 20 10 0 -10
f1 (ppm)

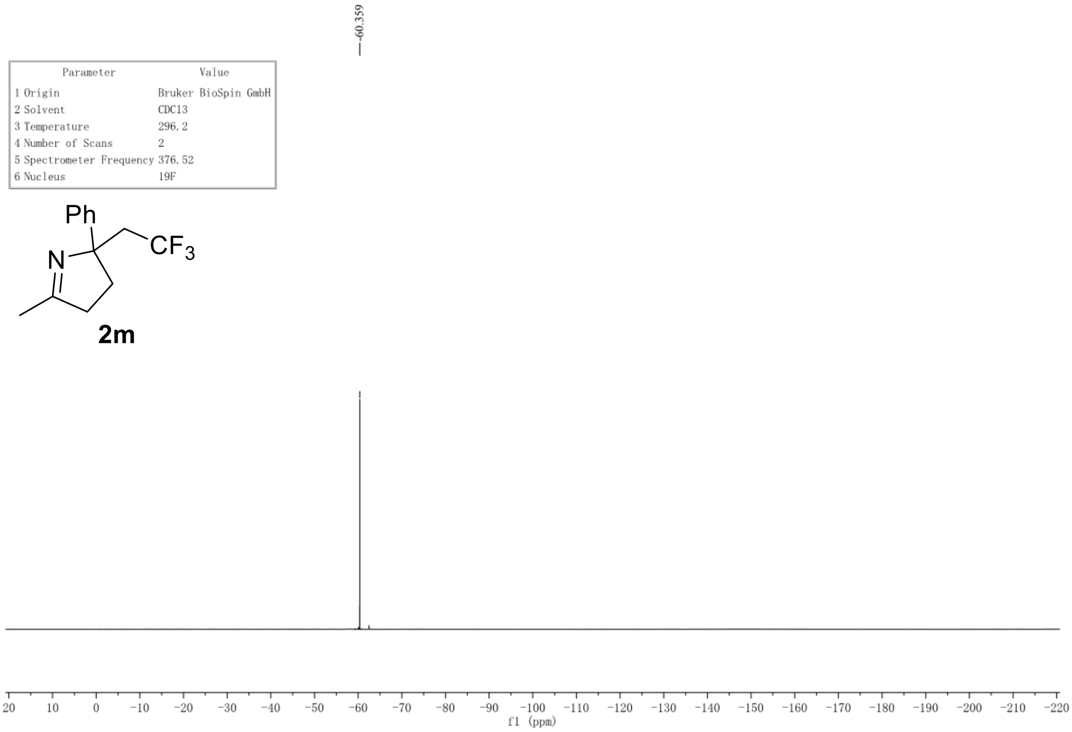

—60.359
Parameter Value
1 Origin Bruker BioSpin GmbH
2 Solvent CDCl3
3 Temperature 296.2
4 Number of Scans 2
5 Spectrometer Frequency 376.52
6 Nucleus 19F
Ph
N
CF3
2m
20 10 0 −10 −20 −30 −40 −50 −60 −70 −80 −90 −100 −110 −120 −130 −140 −150 −160 −170 −180 −190 −200 −210 −220
f1 (ppm)

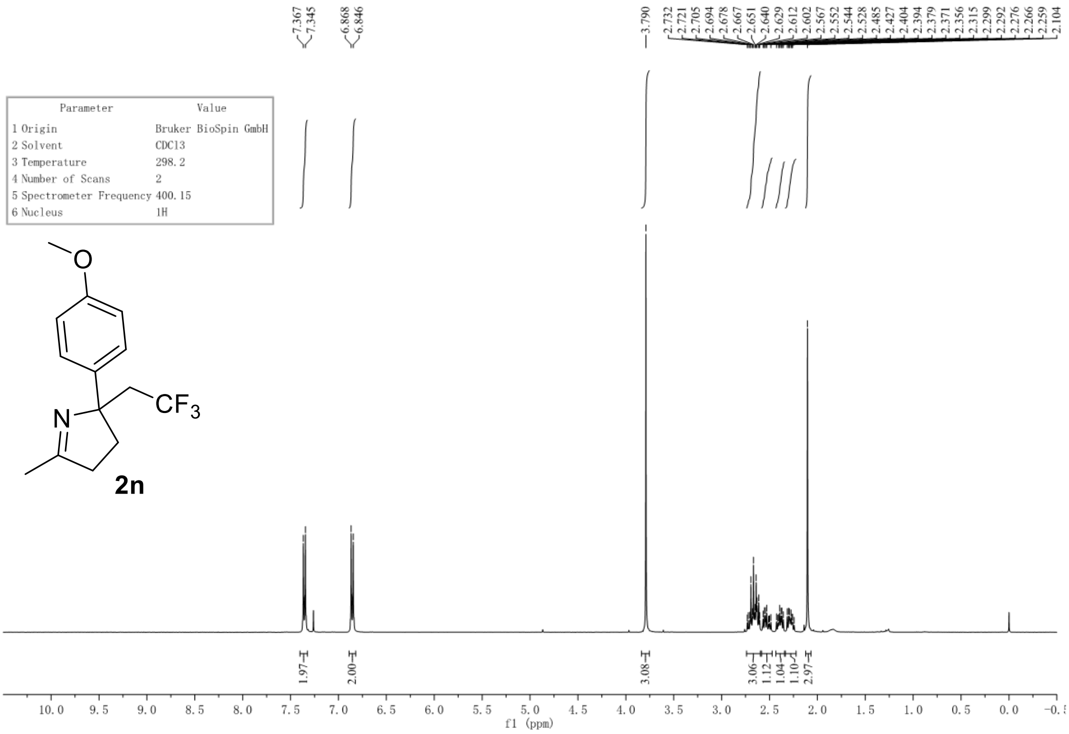

7.367
7.345
6.868
6.846
3.790
2.732 2.721 2.705 2.694 2.678 2.667 2.651 2.640 2.629 2.612 2.602 2.567 2.552 2.544 2.528 2.485 2.427 2.404 2.394 2.379 2.371 2.356 2.315 2.299 2.292 2.276 2.266 2.259 2.104
Parameter Value
1 Origin Bruker BioSpin GmbH
2 Solvent CDCl3
3 Temperature 298.2
4 Number of Scans 2
5 Spectrometer Frequency 400.15
6 Nucleus 1H
O
N
CF3
2n
1.97
2.00
3.08
3.06 1.12 1.04 1.10 2.97
10.0 9.5 9.0 8.5 8.0 7.5 7.0 6.5 6.0 5.5 5.0 4.5 4.0 3.5 3.0 2.5 2.0 1.5 1.0 0.5 0.0
f1 (ppm)

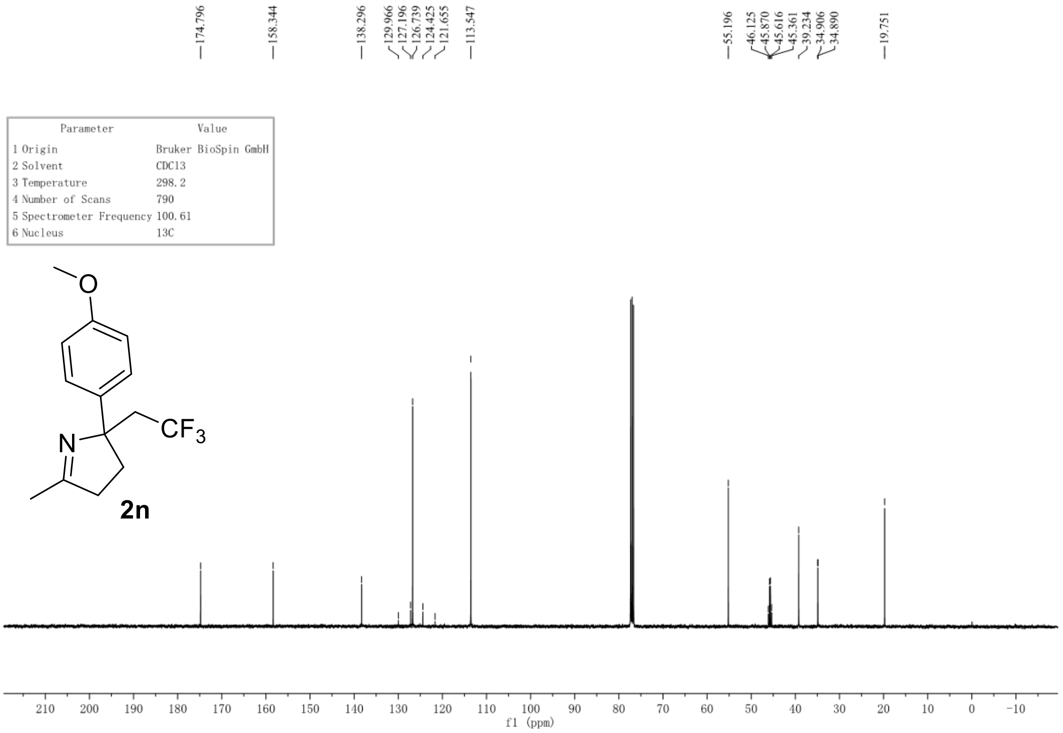
174.796
158.344
138.296
129.966
127.196
126.739
124.425
121.655
113.547
55.196
46.125
45.870
45.616
45.361
39.234
34.906
34.890
19.751
Parameter Value
1 Origin Bruker BioSpin GmbH
2 Solvent CDCl3
3 Temperature 298.2
4 Number of Scans 790
5 Spectrometer Frequency 100.61
6 Nucleus 13C
O
N
CF3
2n
210 200 190 180 170 160 150 140 130 120 110 100 90 80 70 60 50 40 30 20 10 0 -10
f1 (ppm)

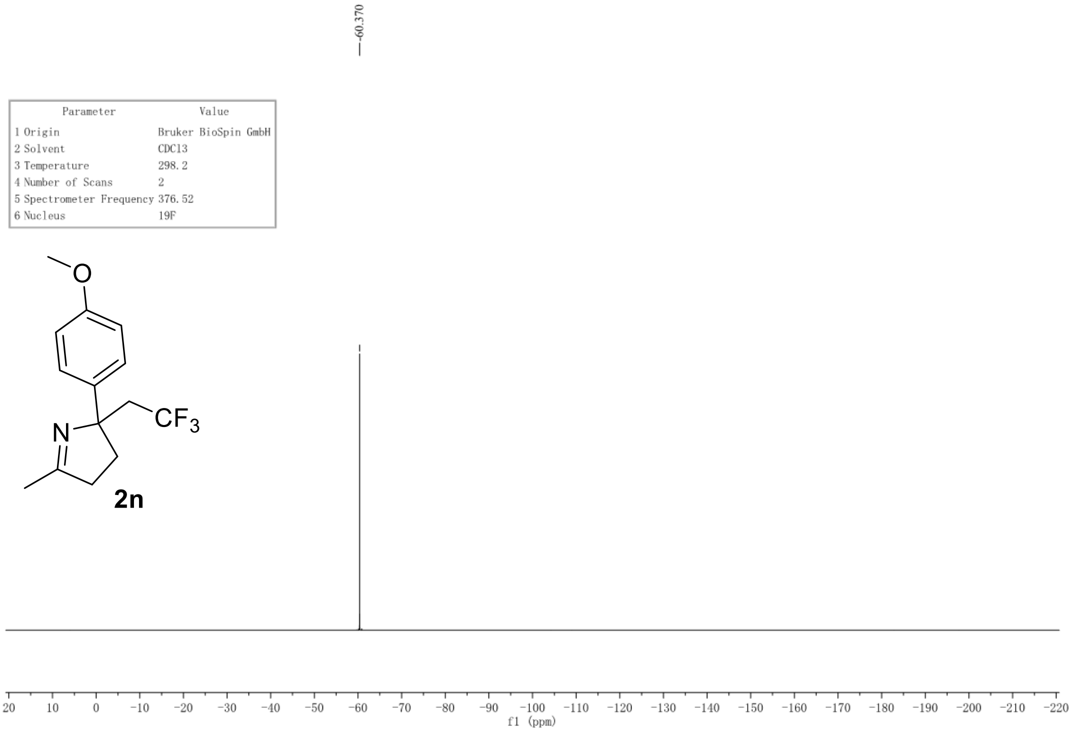
-60.370
Parameter Value
1 Origin Bruker BioSpin GmbH
2 Solvent CDCl3
3 Temperature 298.2
4 Number of Scans 2
5 Spectrometer Frequency 376.52
6 Nucleus 19F
O
N
CF3
2n
20 10 0 -10 -20 -30 -40 -50 -60 -70 -80 -90 -100 -110 -120 -130 -140 -150 -160 -170 -180 -190 -200 -210 -220
f1 (ppm)

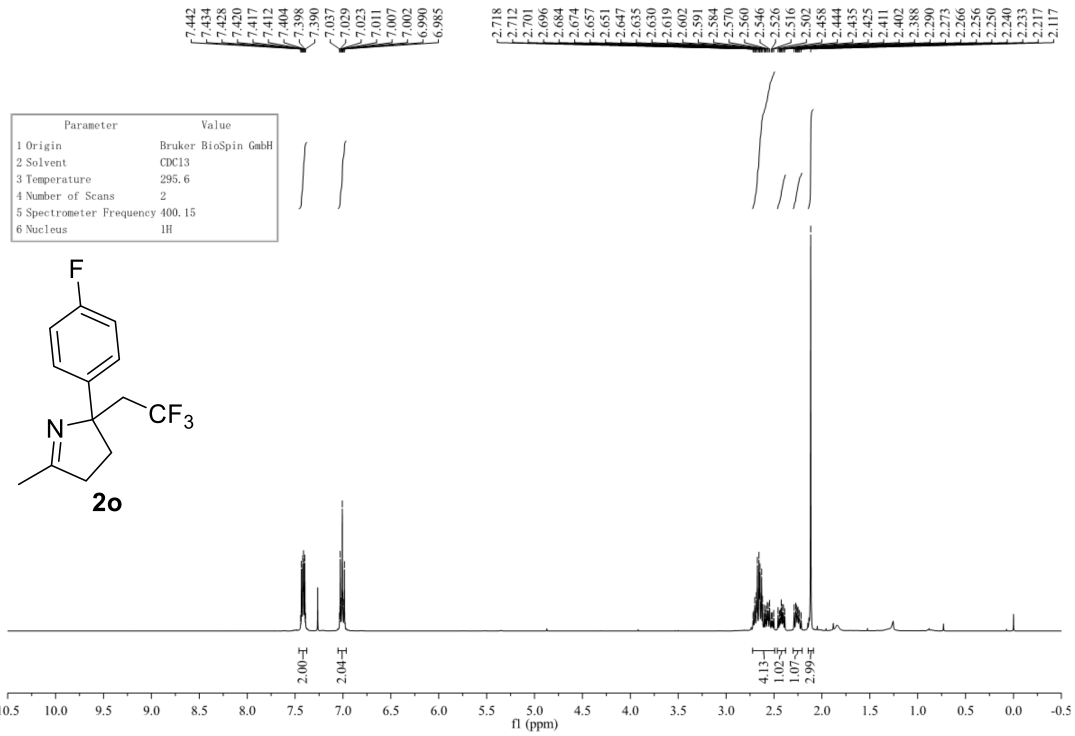

Parameter	Value
1 Origin	Bruker BioSpin GmbH
2 Solvent	CDCl3
3 Temperature	295.6
4 Number of Scans	2
5 Spectrometer Frequency	400.15
6 Nucleus	1H

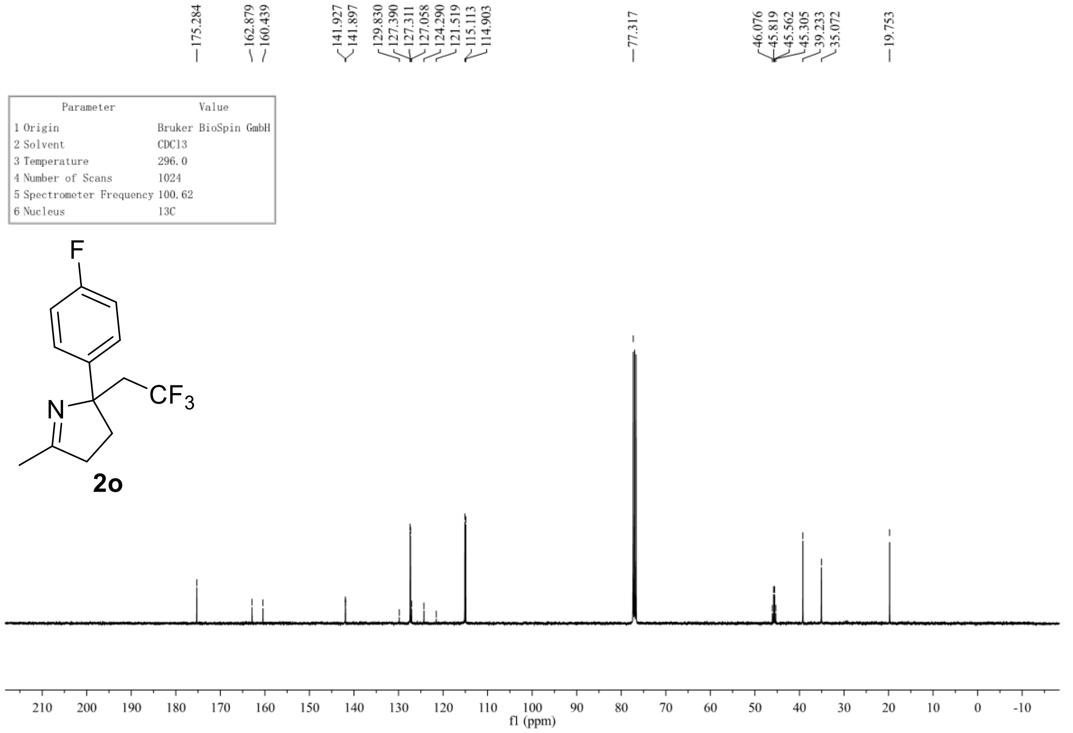

Parameter	Value
1 Origin	Bruker BioSpin GmbH
2 Solvent	CDCl3
3 Temperature	296.0
4 Number of Scans	1024
5 Spectrometer Frequency	100.62
6 Nucleus	13C

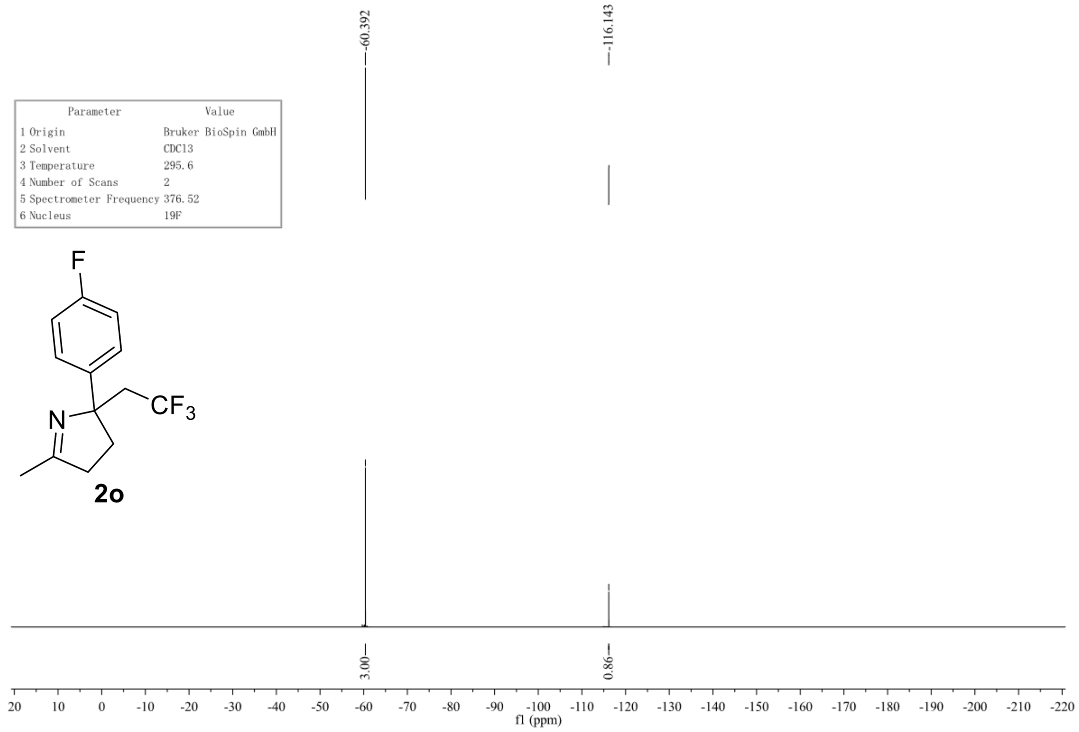
-60.392
-116.143
Parameter Value
1 Origin Bruker BioSpin GmbH
2 Solvent CDCl3
3 Temperature 295.6
4 Number of Scans 2
5 Spectrometer Frequency 376.52
6 Nucleus 19F
F
N
CF3
2o
3.00
0.86
f1 (ppm)

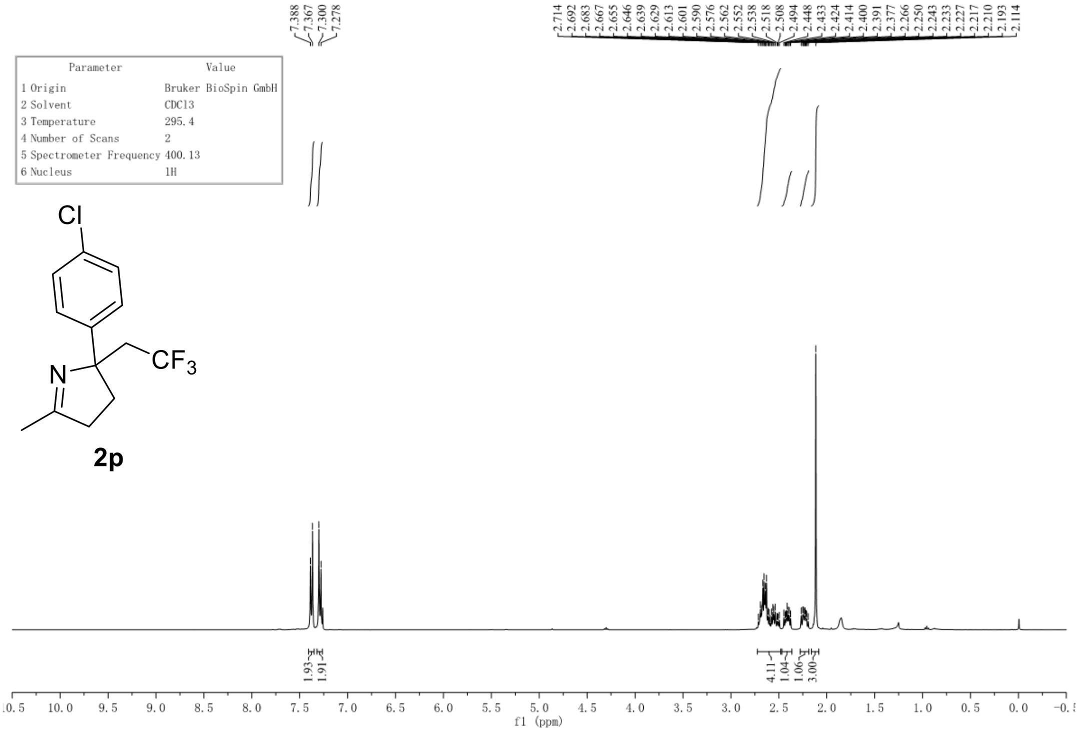
7.388
7.367
7.300
7.278
2.714
2.692
2.683
2.667
2.655
2.646
2.639
2.629
2.613
2.601
2.590
2.576
2.562
2.552
2.538
2.518
2.508
2.494
2.448
2.433
2.424
2.414
2.400
2.391
2.377
2.266
2.250
2.243
2.233
2.227
2.217
2.210
2.193
2.114
Parameter Value
1 Origin Bruker BioSpin GmbH
2 Solvent CDCl3
3 Temperature 295.4
4 Number of Scans 2
5 Spectrometer Frequency 400.13
6 Nucleus 1H
Cl
N
CF3
2p
1.93
1.91
4.11
1.04
1.06
3.00
f1 (ppm)

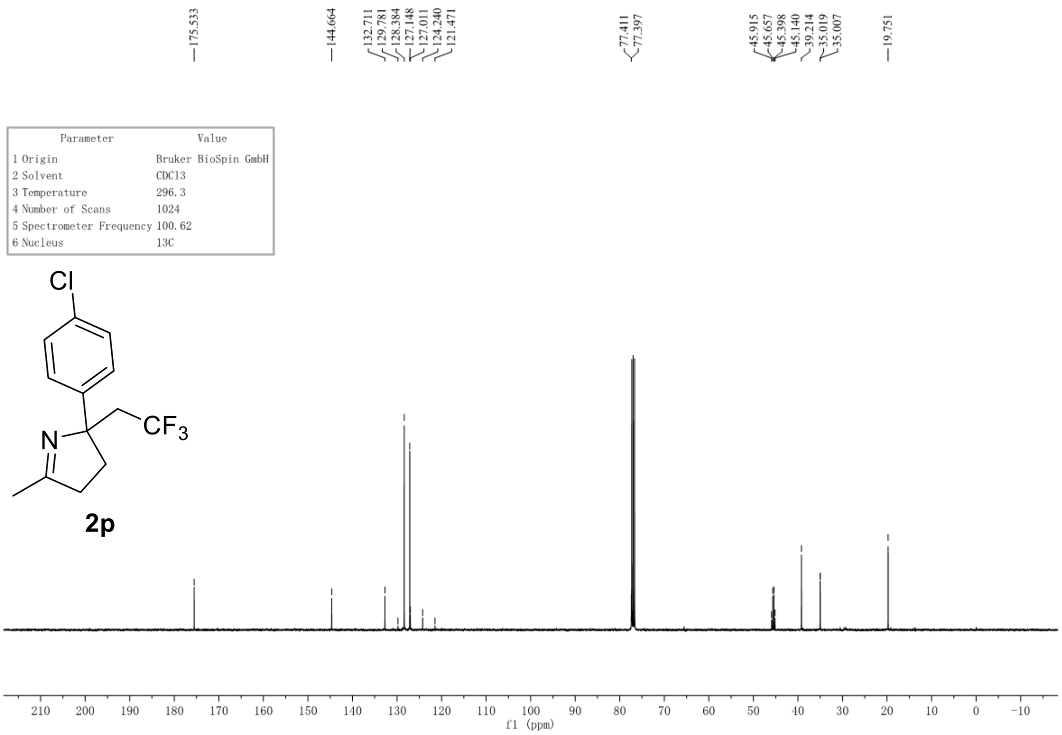
175.533
144.664
132.711
129.781
128.384
127.148
127.011
124.240
121.471
77.411
77.397
45.915
45.657
45.398
45.140
39.214
35.019
35.007
19.751
Parameter Value
1 Origin Bruker BioSpin GmbH
2 Solvent CDCl3
3 Temperature 296.3
4 Number of Scans 1024
5 Spectrometer Frequency 100.62
6 Nucleus 13C
Cl
N
CF3
2p
210 200 190 180 170 160 150 140 130 120 110 100 90 80 70 60 50 40 30 20 10 0 -10
f1 (ppm)

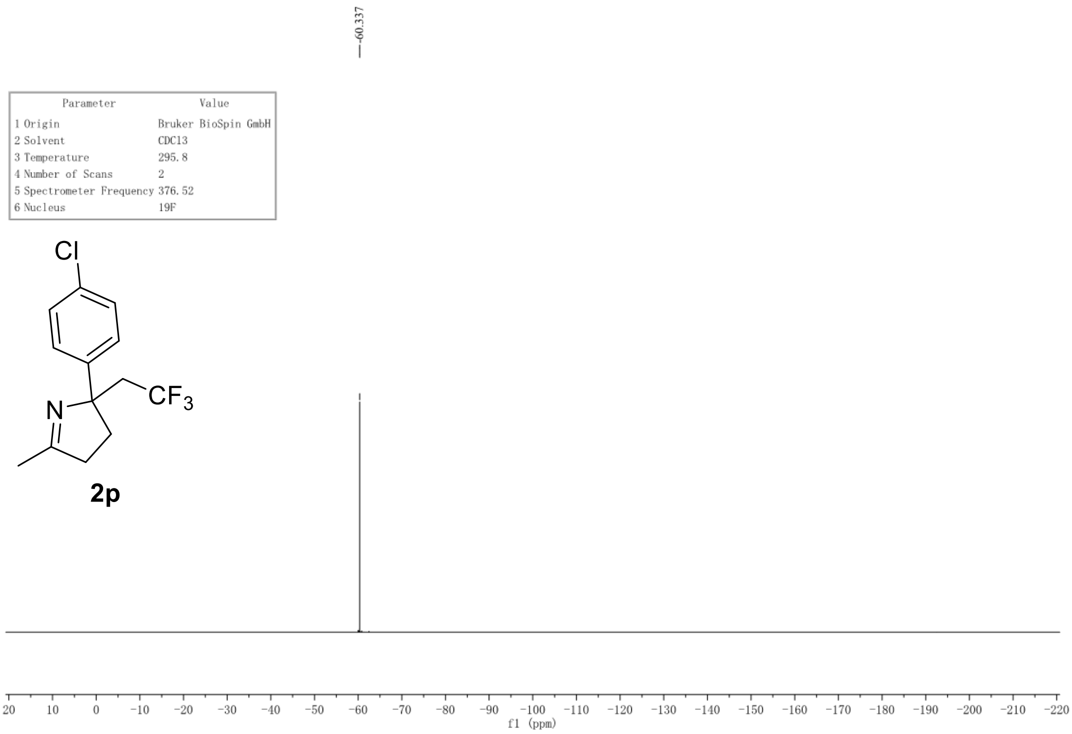
-60.337
Parameter Value
1 Origin Bruker BioSpin GmbH
2 Solvent CDCl3
3 Temperature 295.8
4 Number of Scans 2
5 Spectrometer Frequency 376.52
6 Nucleus 19F
Cl
N
CF3
2p
20 10 0 -10 -20 -30 -40 -50 -60 -70 -80 -90 -100 -110 -120 -130 -140 -150 -160 -170 -180 -190 -200 -210 -220
f1 (ppm)

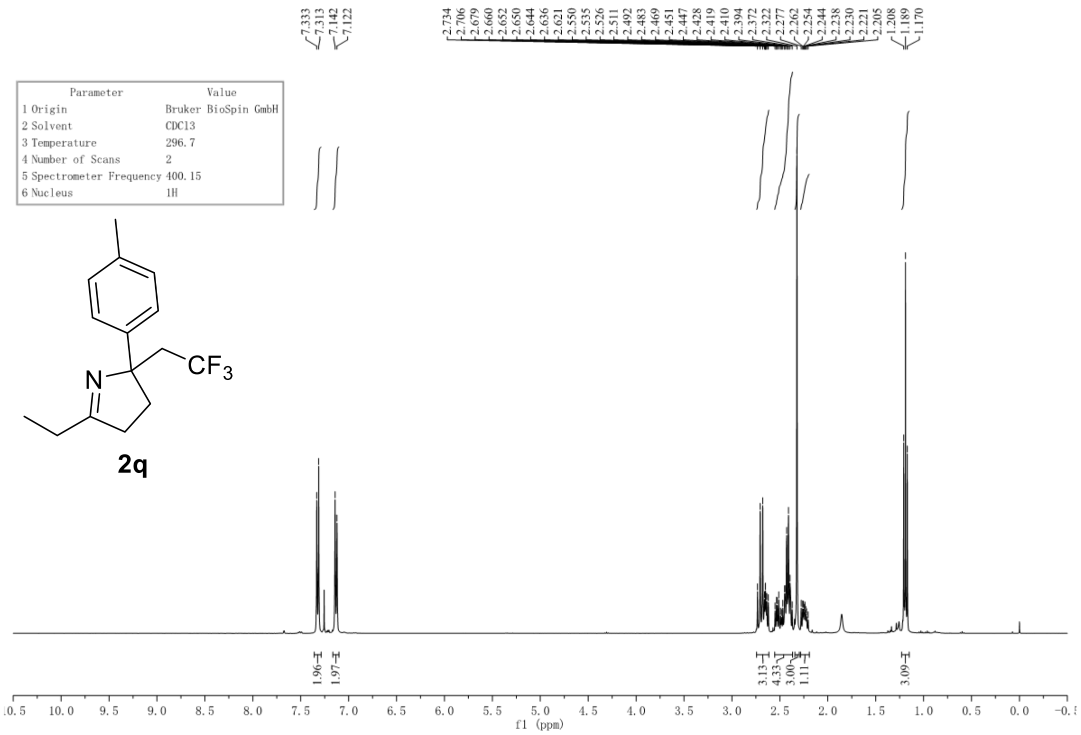
7.333
7.313
7.142
7.122
2.734
2.706
2.679
2.660
2.652
2.650
2.644
2.636
2.621
2.550
2.535
2.526
2.511
2.492
2.483
2.469
2.451
2.447
2.428
2.419
2.410
2.394
2.372
2.322
2.277
2.262
2.254
2.244
2.238
2.230
2.221
2.205
1.208
1.189
1.170
Parameter Value
1 Origin Bruker BioSpin GmbH
2 Solvent CDCl3
3 Temperature 296.7
4 Number of Scans 2
5 Spectrometer Frequency 400.15
6 Nucleus 1H
N
CF3
1.96
1.97
3.13
4.33
3.00
1.11
3.09
f1 (ppm)

2q

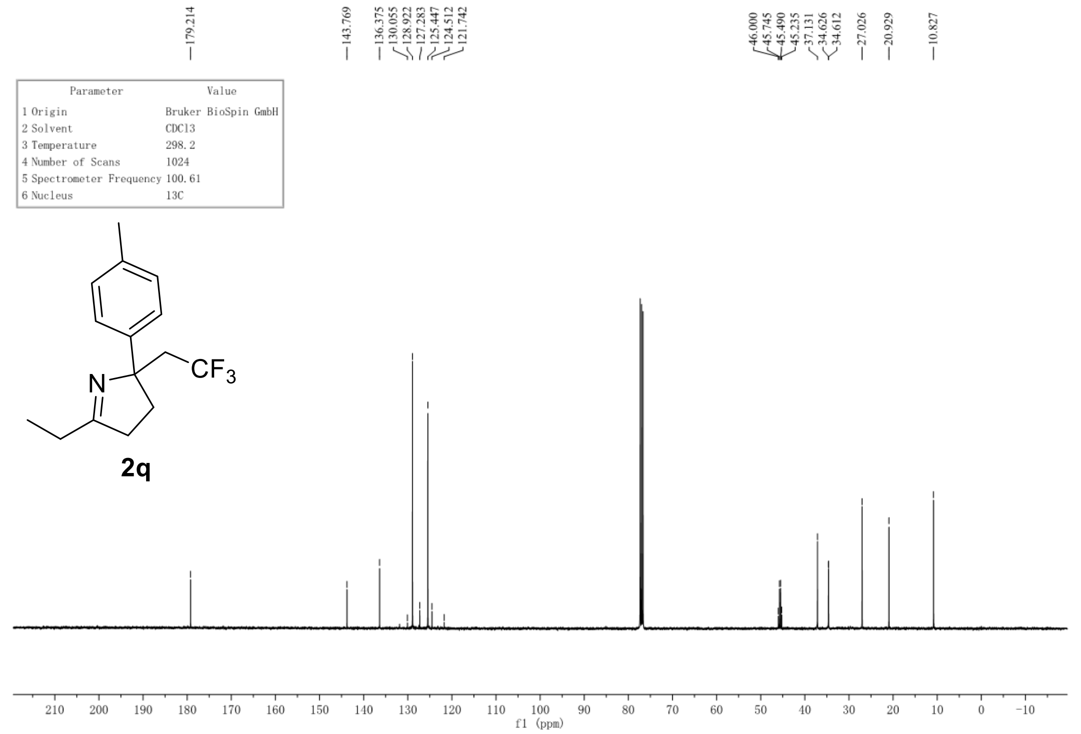
179.214
143.769
136.375
130.055
128.922
127.283
125.447
124.512
121.742
46.000
45.745
45.490
45.235
37.131
34.626
34.612
27.026
20.929
10.827
Parameter Value
1 Origin Bruker BioSpin GmbH
2 Solvent CDCl3
3 Temperature 298.2
4 Number of Scans 1024
5 Spectrometer Frequency 100.61
6 Nucleus 13C
N
CF3
f1 (ppm)

2q

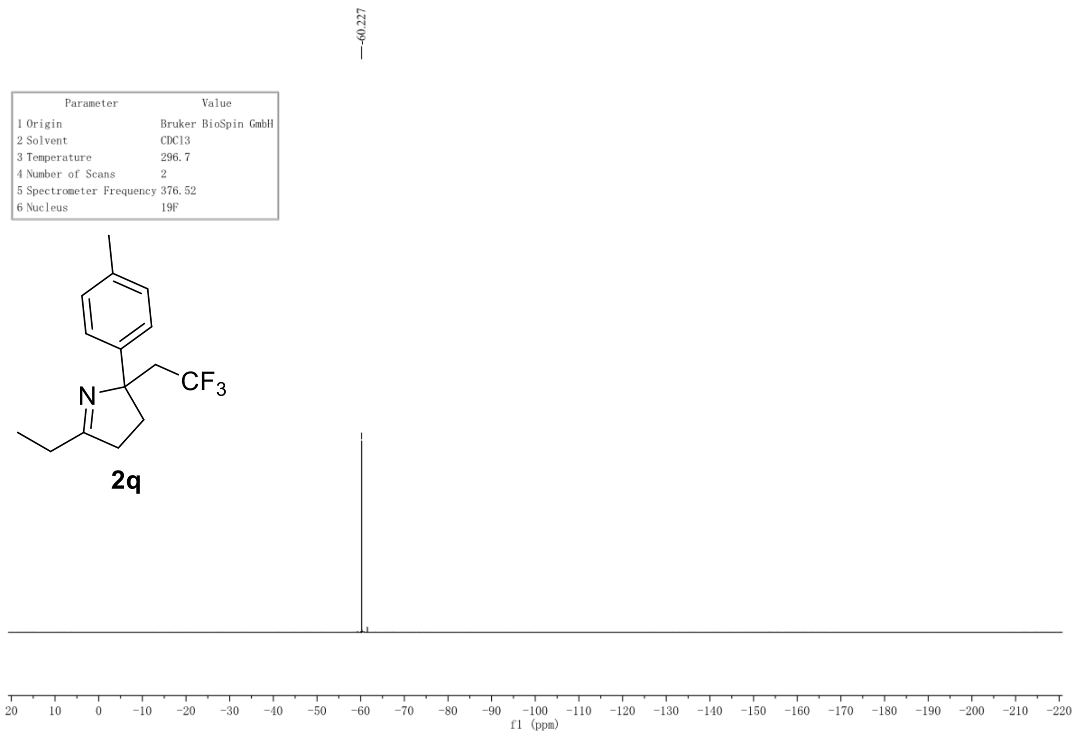
-60.227
Parameter Value
1 Origin Bruker BioSpin GmbH
2 Solvent CDCl3
3 Temperature 296.7
4 Number of Scans 2
5 Spectrometer Frequency 376.52
6 Nucleus 19F
CF3
N
2q
f1 (ppm)

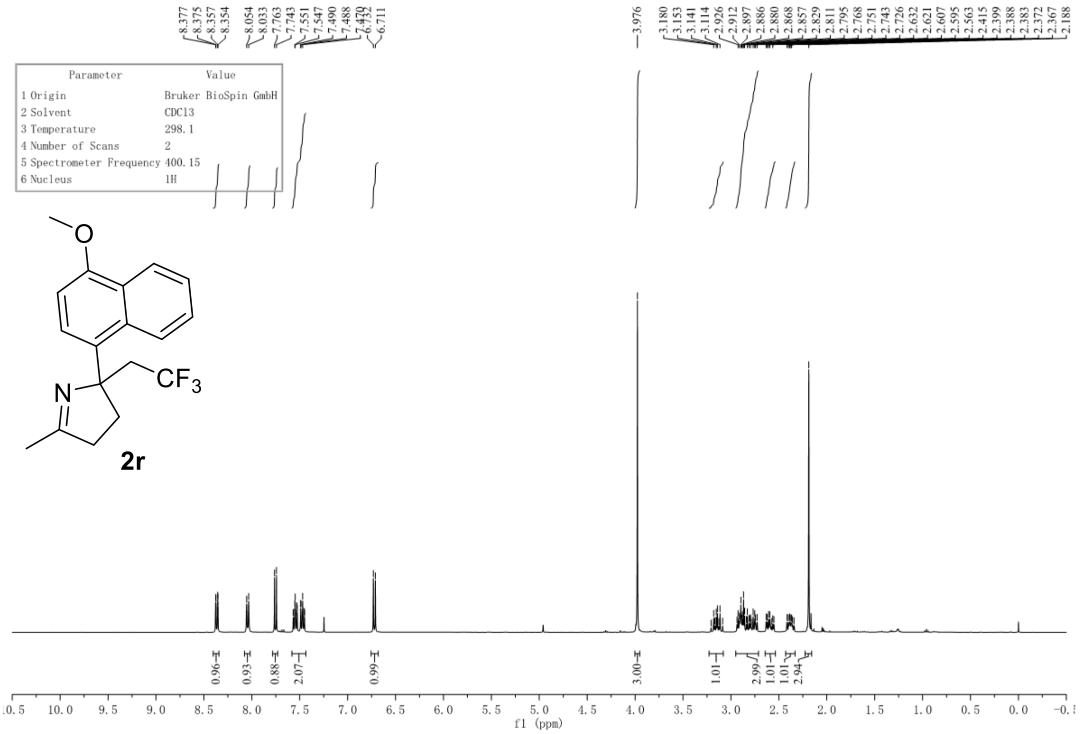
Parameter Value
1 Origin Bruker BioSpin GmbH
2 Solvent CDCl3
3 Temperature 298.1
4 Number of Scans 2
5 Spectrometer Frequency 400.15
6 Nucleus 1H
O
N
CF3
2r
f1 (ppm)

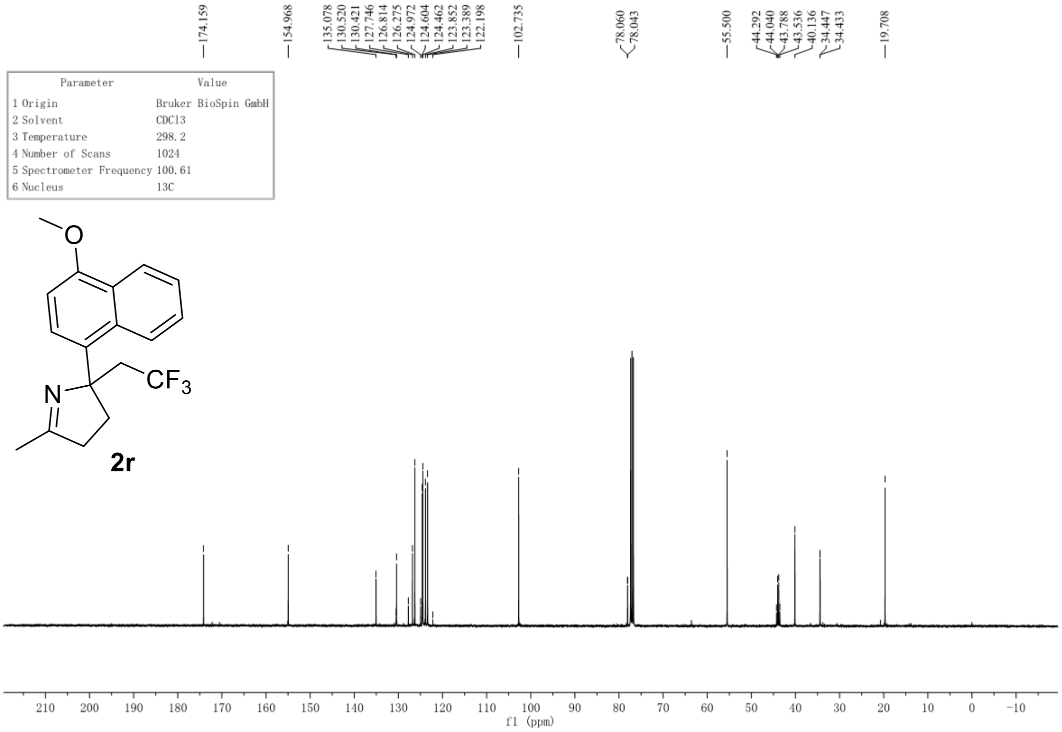
174.159
154.968
135.078
130.520
130.421
127.746
126.814
126.275
124.972
124.604
124.462
123.852
123.389
122.198
102.735
78.060
78.043
55.500
44.292
44.040
43.788
43.536
40.136
34.447
34.433
19.708
Parameter Value
1 Origin Bruker BioSpin GmbH
2 Solvent CDCl3
3 Temperature 298.2
4 Number of Scans 1024
5 Spectrometer Frequency 100.61
6 Nucleus 13C
O
N
CF3
2r
f1 (ppm)

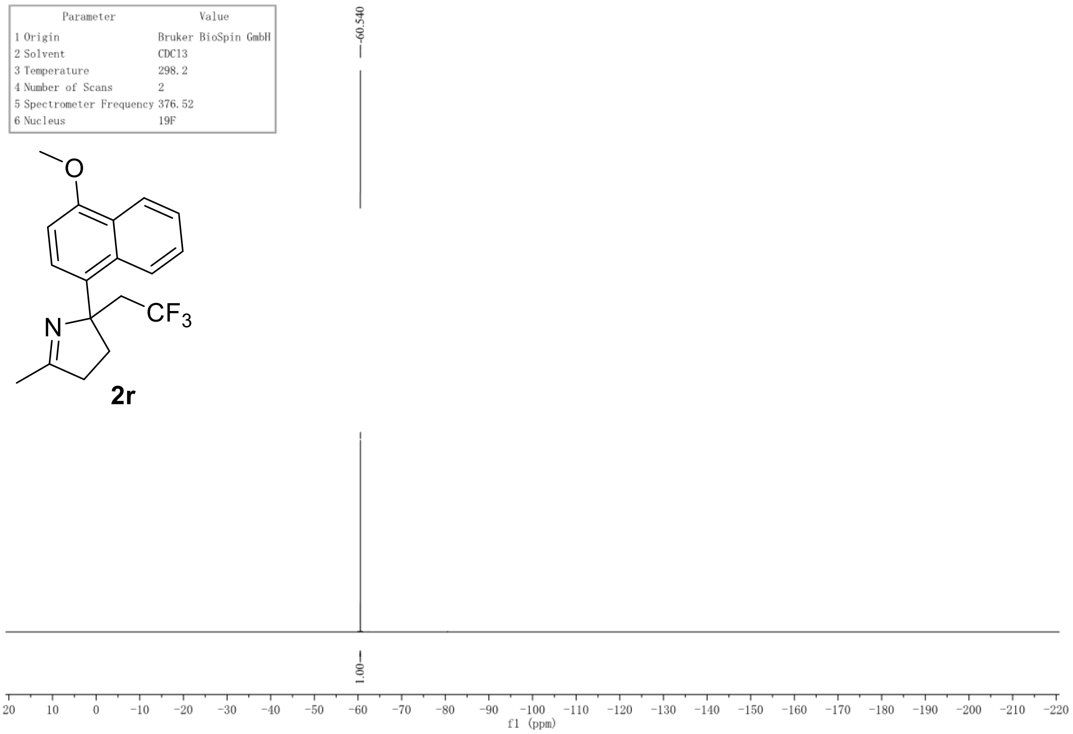
Parameter Value
1 Origin Bruker BioSpin GmbH
2 Solvent CDCl3
3 Temperature 298.2
4 Number of Scans 2
5 Spectrometer Frequency 376.52
6 Nucleus 19F
-60.540
O
N
CF3
2r
1.00
f1 (ppm)

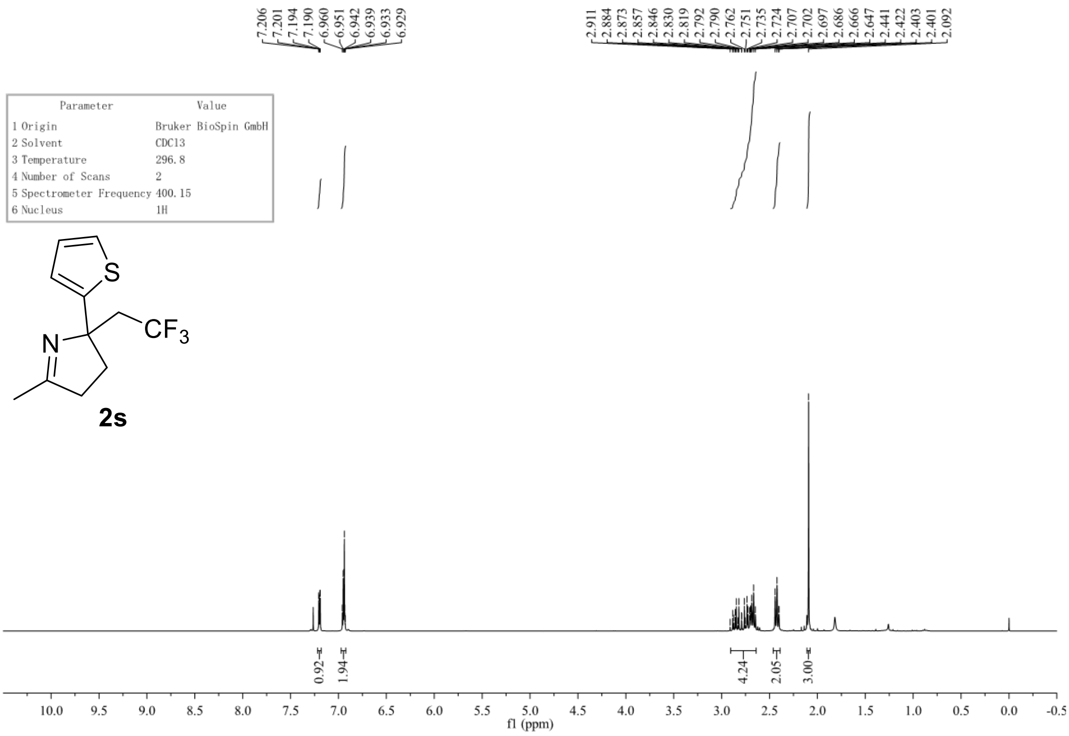
7.206 7.201 7.194 7.190 6.960 6.951 6.942 6.939 6.933 6.929
2.911 2.884 2.873 2.857 2.846 2.830 2.819 2.792 2.790 2.762 2.751 2.735 2.724 2.707 2.702 2.697 2.686 2.666 2.647 2.441 2.422 2.403 2.401 2.092
Parameter Value
1 Origin Bruker BioSpin GmbH
2 Solvent CDCl3
3 Temperature 296.8
4 Number of Scans 2
5 Spectrometer Frequency 400.15
6 Nucleus 1H
S
N
CF3
2s
0.92
1.94
4.24
2.05
3.00
10.0 9.5 9.0 8.5 8.0 7.5 7.0 6.5 6.0 5.5 5.0 4.5 4.0 3.5 3.0 2.5 2.0 1.5 1.0 0.5 0.0 -0.5
f1 (ppm)

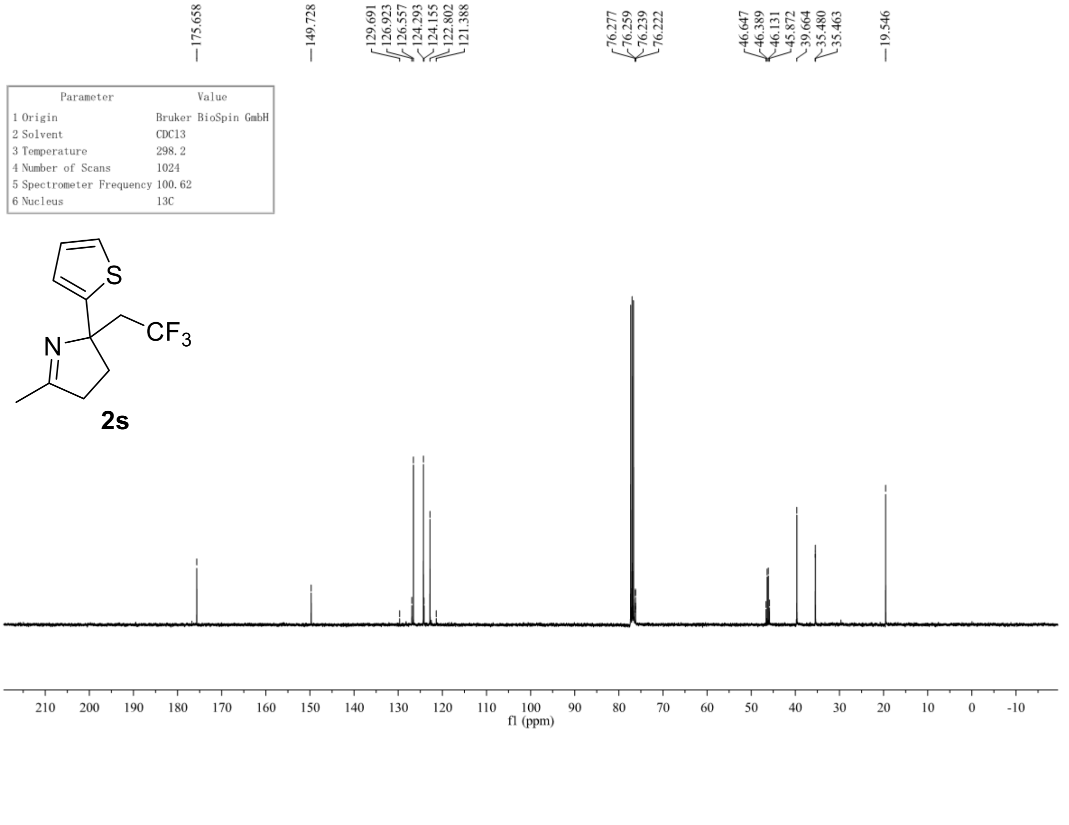
175.658
149.728
129.691 126.923 126.557 124.293 124.155 122.802 121.388
76.277 76.259 76.239 76.222
46.647 46.389 46.131 45.872 39.664 35.480 35.463
19.546
Parameter Value
1 Origin Bruker BioSpin GmbH
2 Solvent CDCl3
3 Temperature 298.2
4 Number of Scans 1024
5 Spectrometer Frequency 100.62
6 Nucleus 13C
S
N
CF3
2s
210 200 190 180 170 160 150 140 130 120 110 100 90 80 70 60 50 40 30 20 10 0 -10
f1 (ppm)

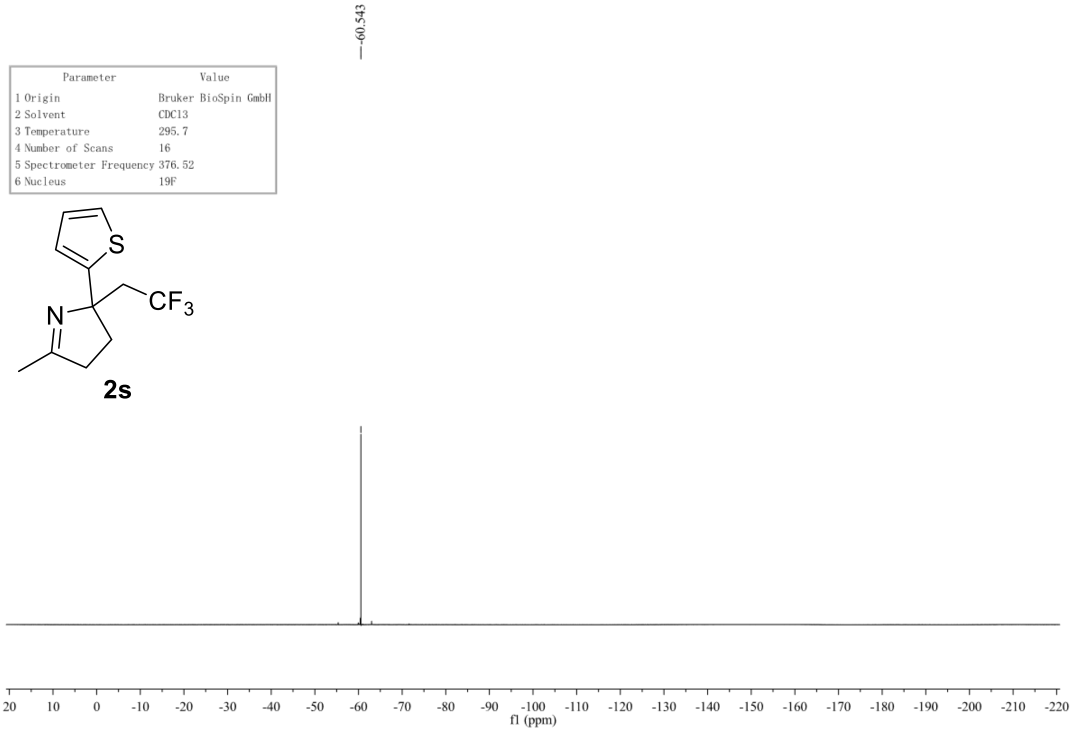

—-60.543
Parameter Value
1 Origin Bruker BioSpin GmbH
2 Solvent CDCl3
3 Temperature 295.7
4 Number of Scans 16
5 Spectrometer Frequency 376.52
6 Nucleus 19F
S
N
CF3
2s
20 10 0 -10 -20 -30 -40 -50 -60 -70 -80 -90 -100 -110 -120 -130 -140 -150 -160 -170 -180 -190 -200 -210 -220
f1 (ppm)

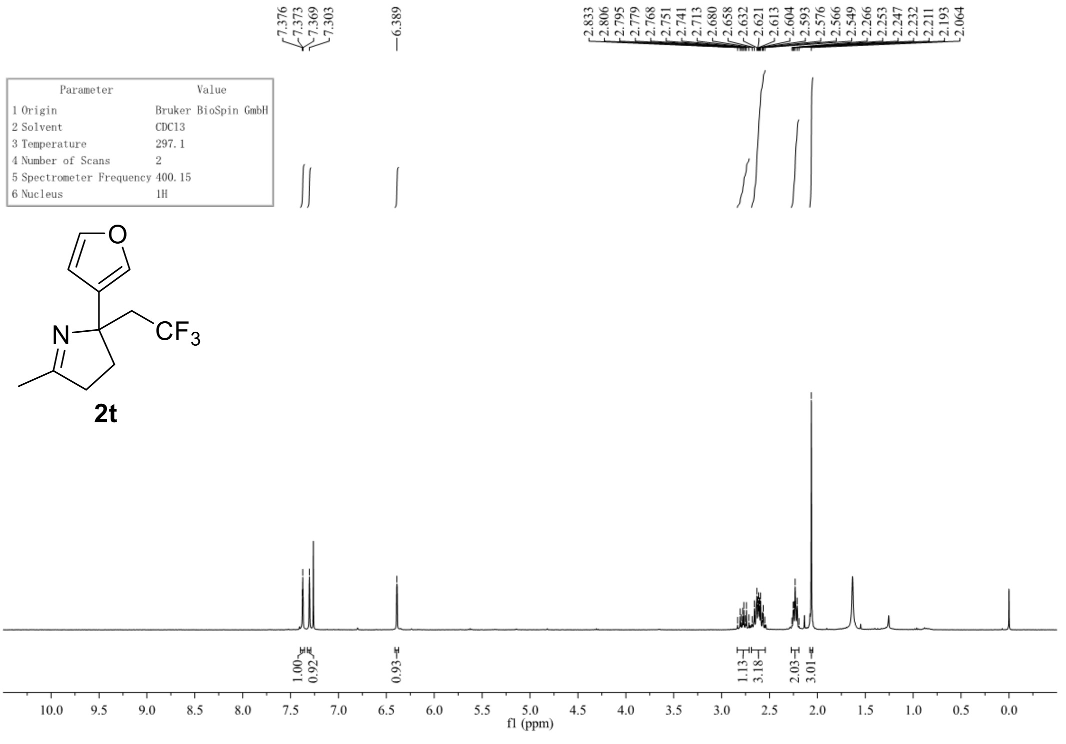

7.376 7.373 7.369 7.303
6.389
2.833 2.806 2.795 2.779 2.768 2.751 2.741 2.713 2.680 2.658 2.632 2.621 2.613 2.604 2.593 2.576 2.566 2.549 2.266 2.253 2.247 2.232 2.211 2.193 2.064
Parameter Value
1 Origin Bruker BioSpin GmbH
2 Solvent CDCl3
3 Temperature 297.1
4 Number of Scans 2
5 Spectrometer Frequency 400.15
6 Nucleus 1H
O
N
CF3
2t
1.00 0.92
0.93
1.13 3.18
2.03 3.01
10.0 9.5 9.0 8.5 8.0 7.5 7.0 6.5 6.0 5.5 5.0 4.5 4.0 3.5 3.0 2.5 2.0 1.5 1.0 0.5 0.0
f1 (ppm)

Parameter	Value
1 Origin	Bruker BioSpin GmbH
2 Solvent	CDCl3
3 Temperature	297.8
4 Number of Scans	1024
5 Spectrometer Frequency	100.62
6 Nucleus	13C

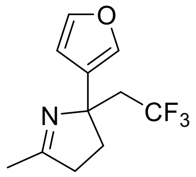

2t

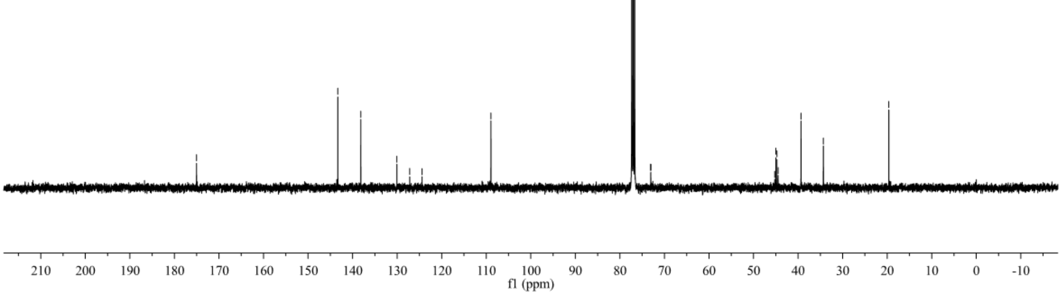

Parameter	Value
1 Origin	Bruker BioSpin GmbH
2 Solvent	CDCl3
3 Temperature	297.1
4 Number of Scans	2
5 Spectrometer Frequency	376.52
6 Nucleus	19F

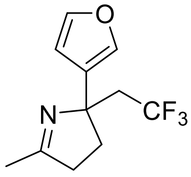

2t

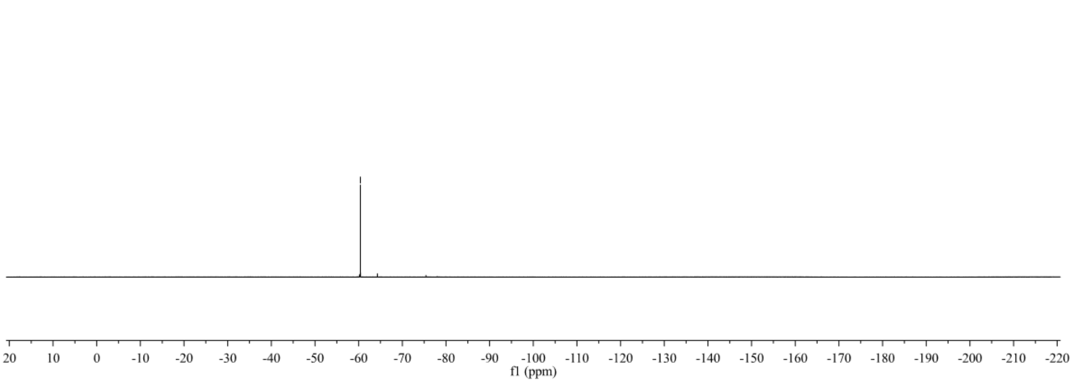

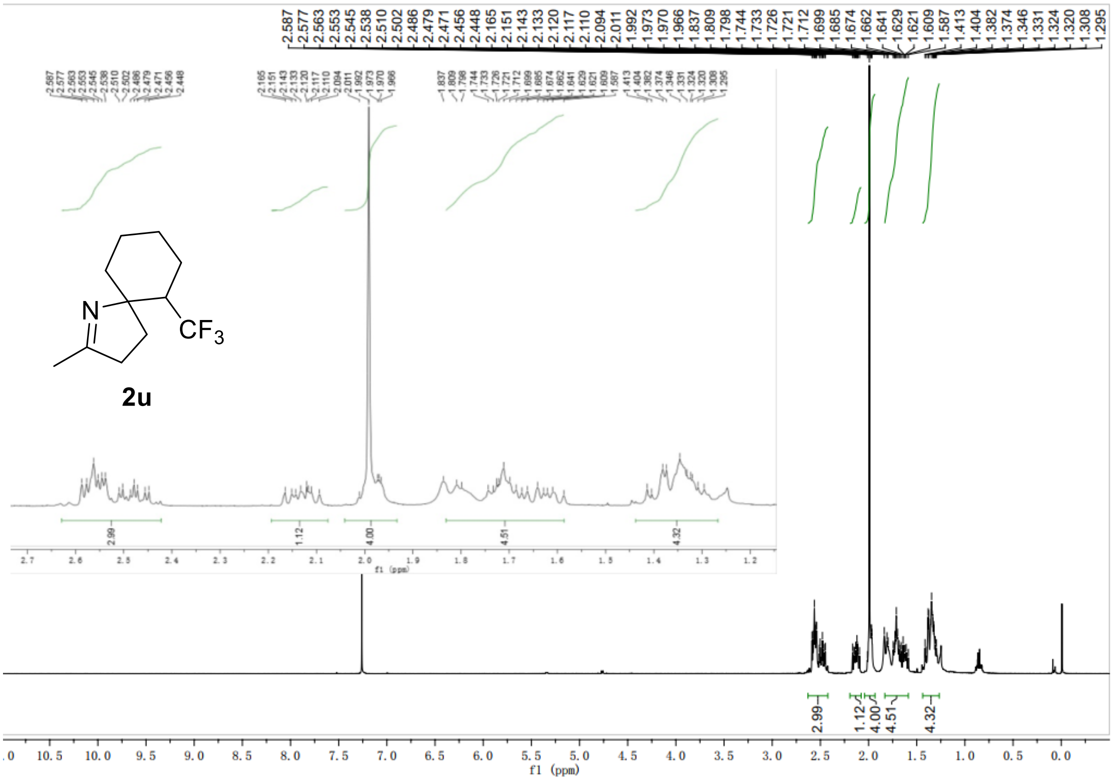
2.587
2.577
2.563
2.553
2.545
2.538
2.510
2.502
2.486
2.479
2.471
2.456
2.448
2.165
2.151
2.143
2.133
2.120
2.117
2.110
2.094
2.011
1.992
1.973
1.970
1.966
1.837
1.809
1.798
1.744
1.733
1.726
1.721
1.712
1.699
1.685
1.674
1.662
1.641
1.629
1.621
1.609
1.587
1.413
1.404
1.382
1.374
1.346
1.331
1.324
1.320
1.308
1.295
2u
2.99
1.12
4.00
4.51
4.32
f1 (ppm)

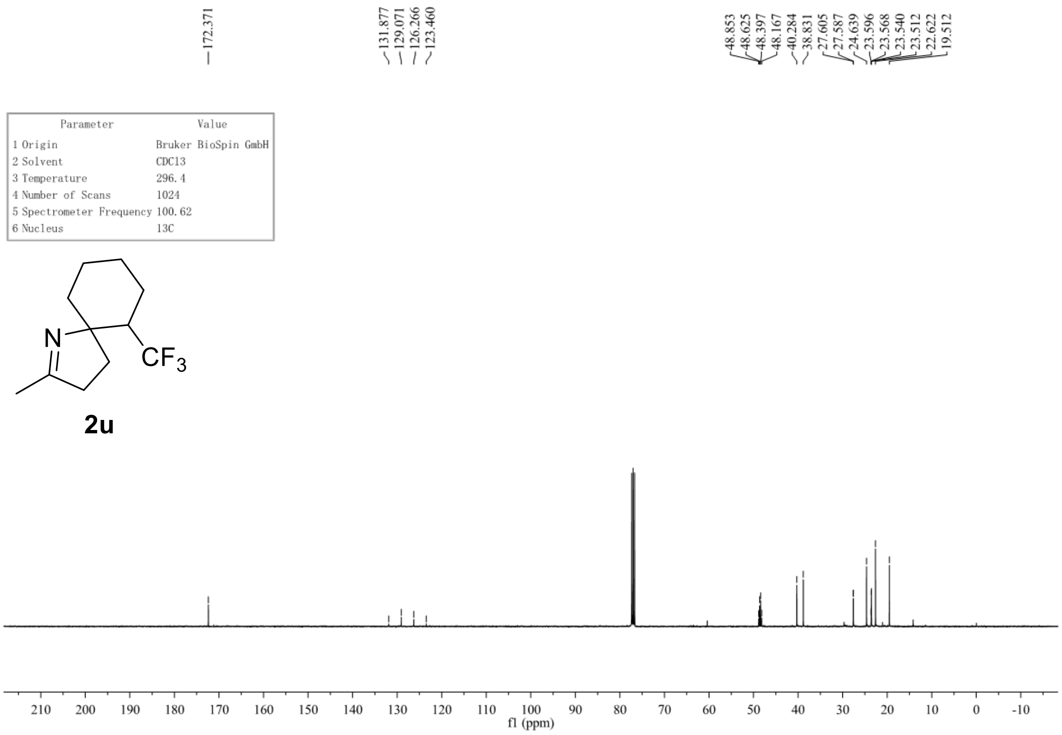
172.371
131.877
129.071
126.266
123.460
48.853
48.625
48.397
48.167
40.284
38.831
27.605
27.587
24.639
23.596
23.568
23.540
23.512
22.622
19.512
Parameter Value
1 Origin Bruker BioSpin GmbH
2 Solvent CDCl3
3 Temperature 296.4
4 Number of Scans 1024
5 Spectrometer Frequency 100.62
6 Nucleus 13C
2u
f1 (ppm)

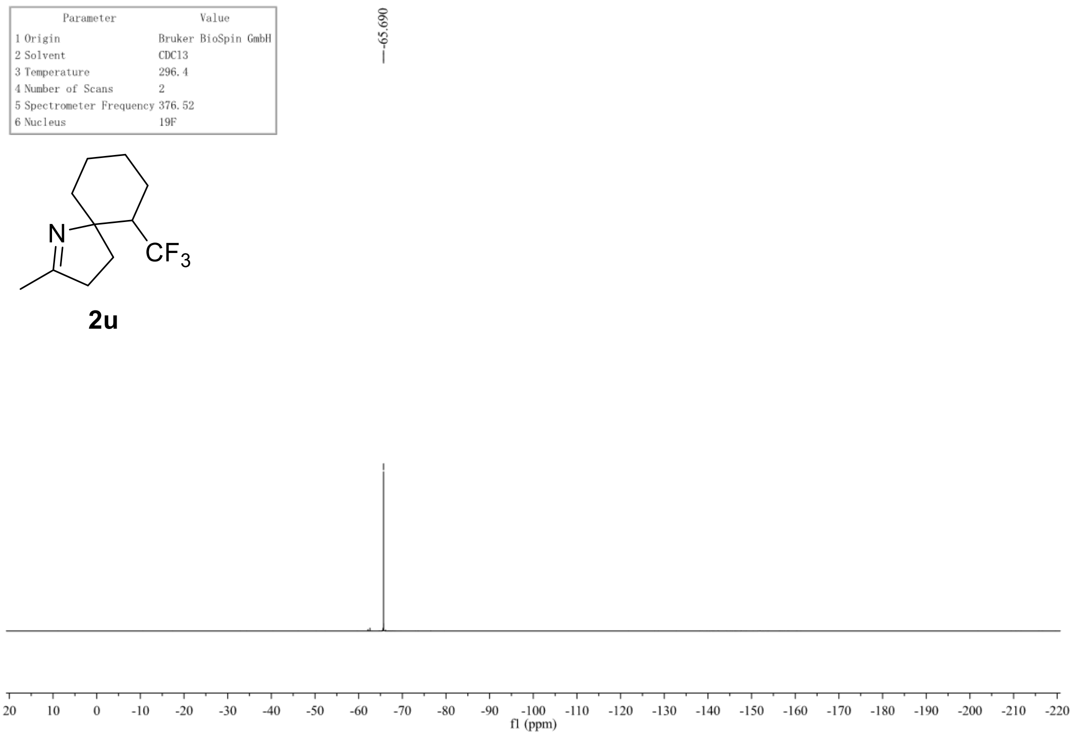

Parameter Value
1 Origin Bruker BioSpin GmbH
2 Solvent CDCl3
3 Temperature 296.4
4 Number of Scans 2
5 Spectrometer Frequency 376.52
6 Nucleus 19F
—-65.690
N
CF3
2u
20 10 0 -10 -20 -30 -40 -50 -60 -70 -80 -90 -100 -110 -120 -130 -140 -150 -160 -170 -180 -190 -200 -210 -220
f1 (ppm)

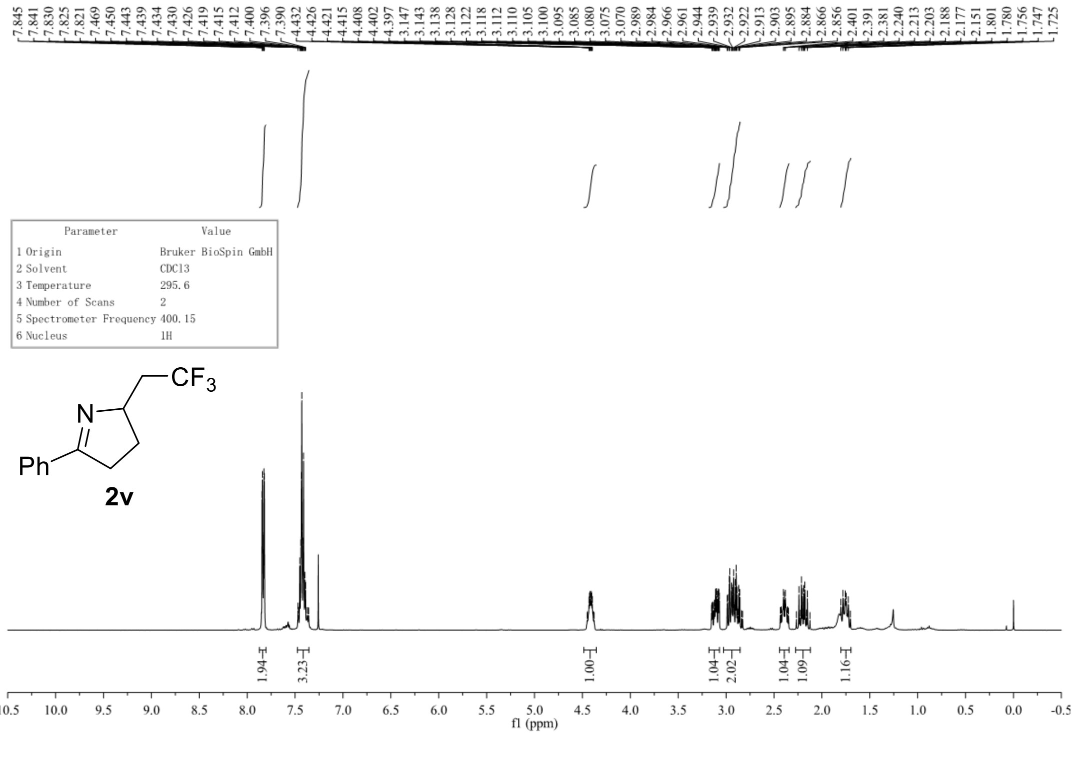

7.845 7.841 7.830 7.825 7.821 7.469 7.450 7.443 7.439 7.434 7.430 7.426 7.419 7.415 7.412 7.400 7.396 7.390 4.432 4.426 4.421 4.415 4.408 4.402 4.397 3.147 3.143 3.138 3.128 3.122 3.118 3.112 3.110 3.105 3.100 3.095 3.085 3.080 3.075 3.070 2.989 2.984 2.966 2.961 2.944 2.939 2.932 2.922 2.913 2.903 2.895 2.884 2.866 2.856 2.401 2.391 2.381 2.240 2.213 2.203 2.188 2.177 2.151 1.801 1.780 1.756 1.747 1.725
Parameter Value
1 Origin Bruker BioSpin GmbH
2 Solvent CDCl3
3 Temperature 295.6
4 Number of Scans 2
5 Spectrometer Frequency 400.15
6 Nucleus 1H
CF3
N
Ph
2v
1.94 3.23 1.00 1.04 2.02 1.04 1.09 1.16
10.5 10.0 9.5 9.0 8.5 8.0 7.5 7.0 6.5 6.0 5.5 5.0 4.5 4.0 3.5 3.0 2.5 2.0 1.5 1.0 0.5 0.0 -0.5
f1 (ppm)

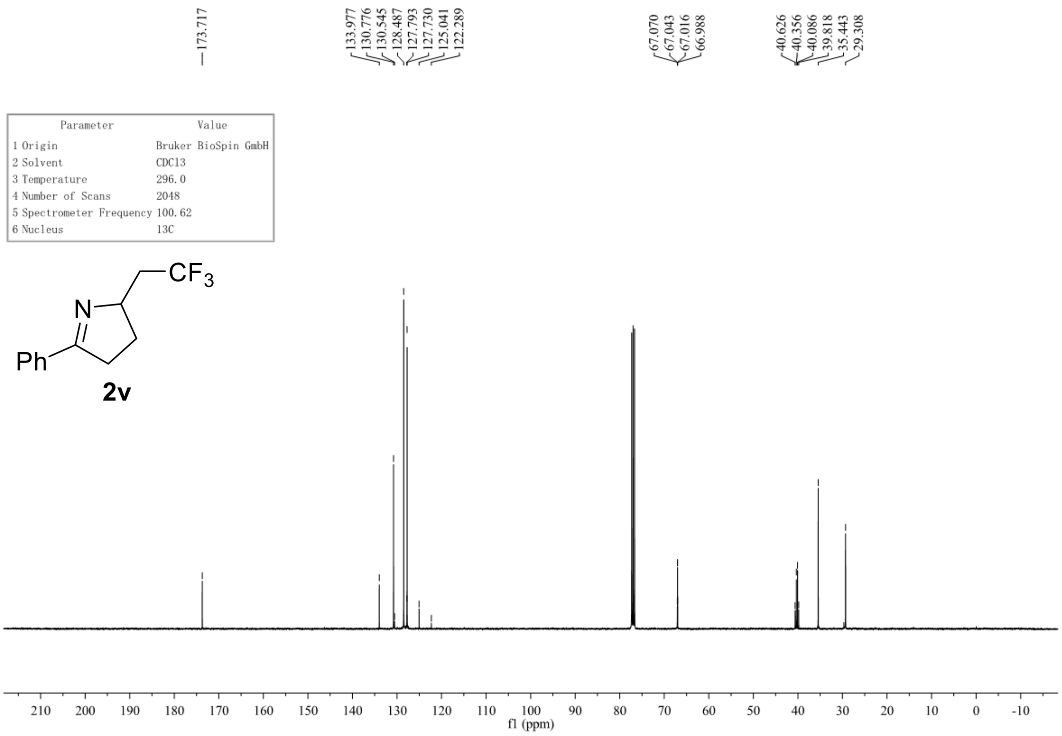

Parameter	Value
1 Origin	Bruker BioSpin GmbH
2 Solvent	CDCl3
3 Temperature	296.0
4 Number of Scans	2048
5 Spectrometer Frequency	100.62
6 Nucleus	13C

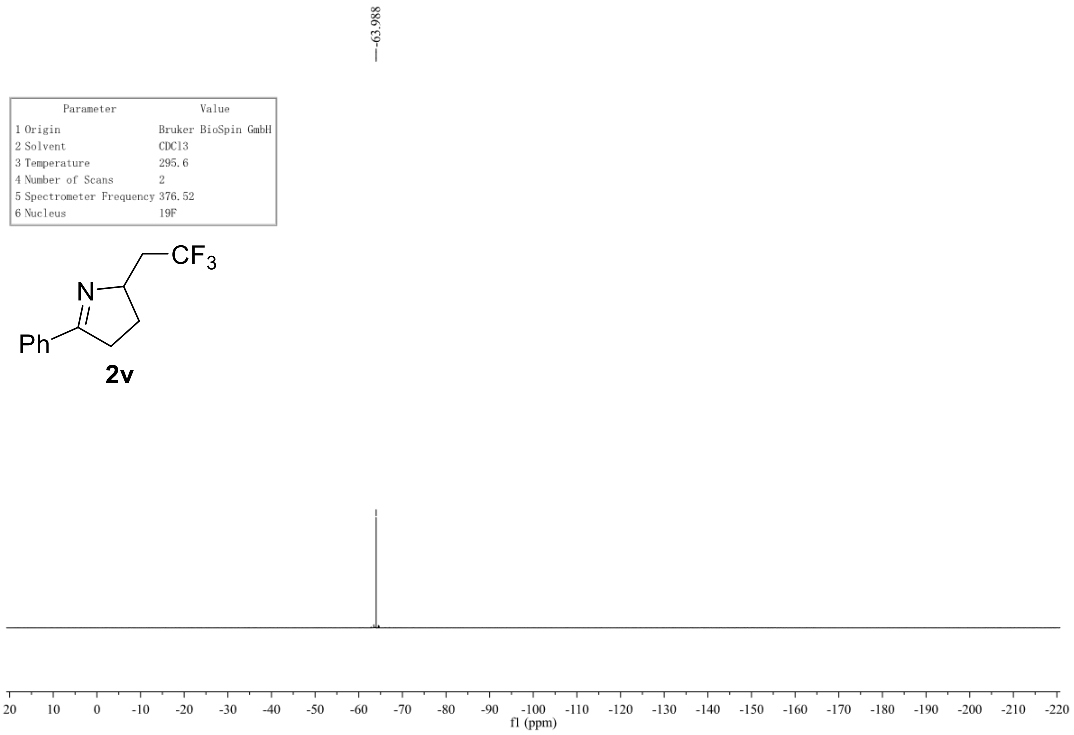

Parameter	Value
1 Origin	Bruker BioSpin GmbH
2 Solvent	CDCl3
3 Temperature	295.6
4 Number of Scans	2
5 Spectrometer Frequency	376.52
6 Nucleus	19F

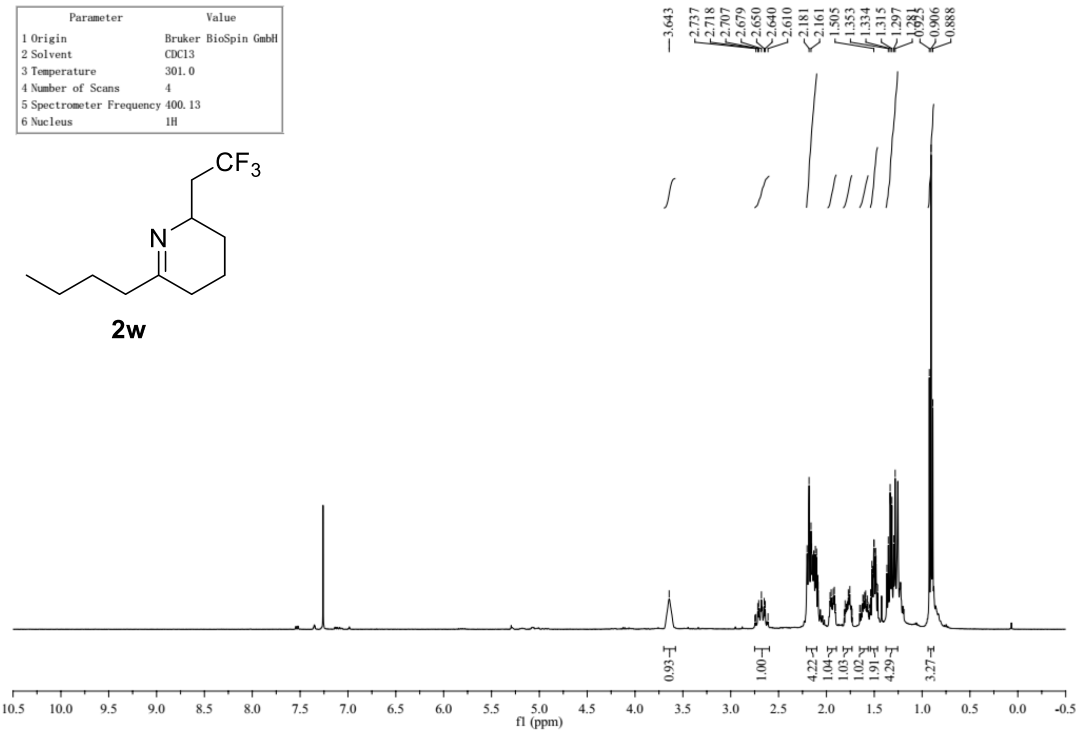
Parameter Value
1 Origin Bruker BioSpin GmbH
2 Solvent CDCl3
3 Temperature 301.0
4 Number of Scans 4
5 Spectrometer Frequency 400.13
6 Nucleus 1H
CF3
N
2w
3.643
2.737 2.718 2.707 2.679 2.650 2.640 2.610 2.181 2.161 1.505 1.353 1.334 1.315 1.297 0.906 0.888
0.93 1.00 4.22 1.04 1.03 1.02 1.91 4.29 3.27
f1 (ppm)

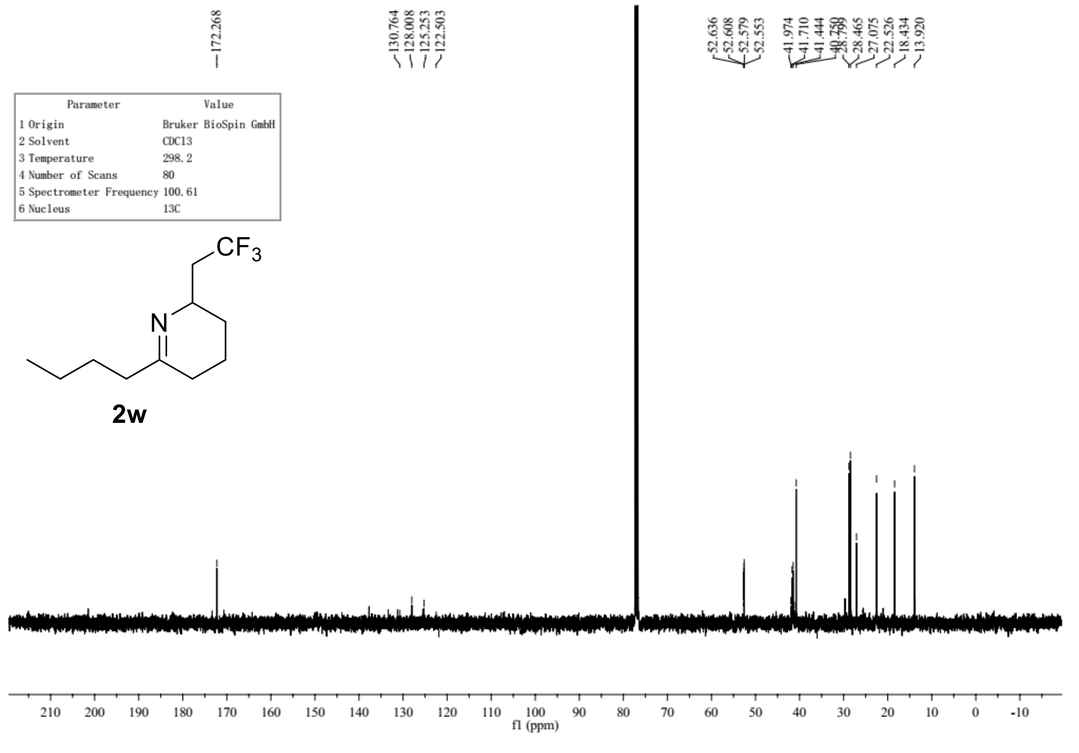
172.268
130.764 128.008 125.253 122.503
52.636 52.608 52.579 52.553 41.974 41.710 41.444 28.465 27.075 22.526 18.434 13.920
Parameter Value
1 Origin Bruker BioSpin GmbH
2 Solvent CDCl3
3 Temperature 298.2
4 Number of Scans 80
5 Spectrometer Frequency 100.61
6 Nucleus 13C
CF3
N
2w
f1 (ppm)

Parameter	Value
1 Origin	Bruker BioSpin GmbH
2 Solvent	CDCl3
3 Temperature	301.1
4 Number of Scans	16
5 Spectrometer Frequency	376.50
6 Nucleus	19F

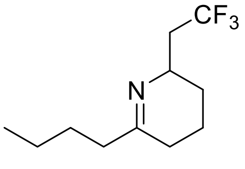

2w

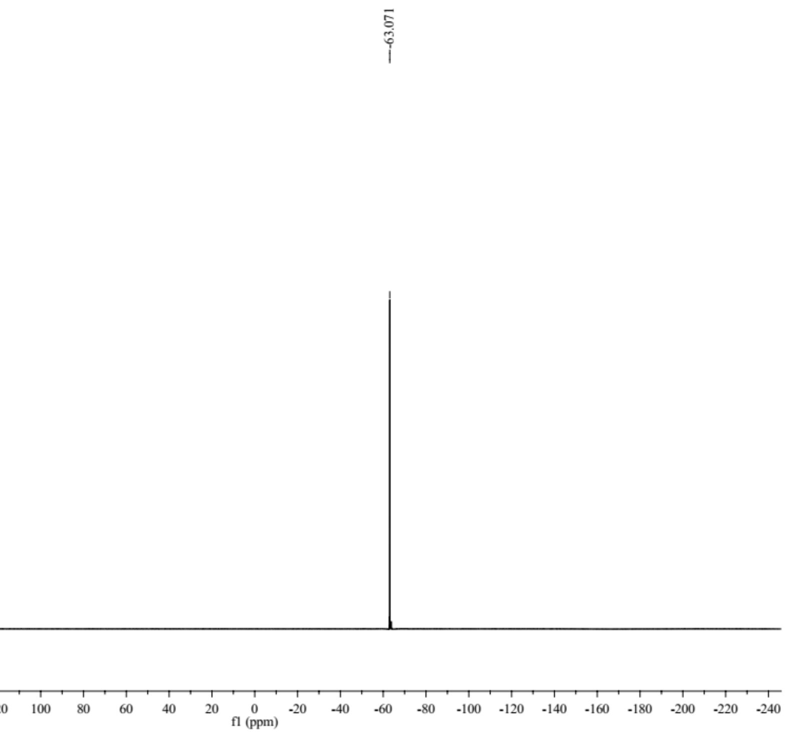

Parameter	Value
1 Origin	Bruker BioSpin GmbH
2 Solvent	CDCl3
3 Temperature	297.0
4 Number of Scans	2
5 Spectrometer Frequency	400.15
6 Nucleus	1H

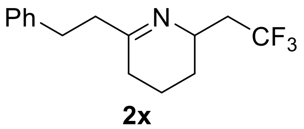

2x

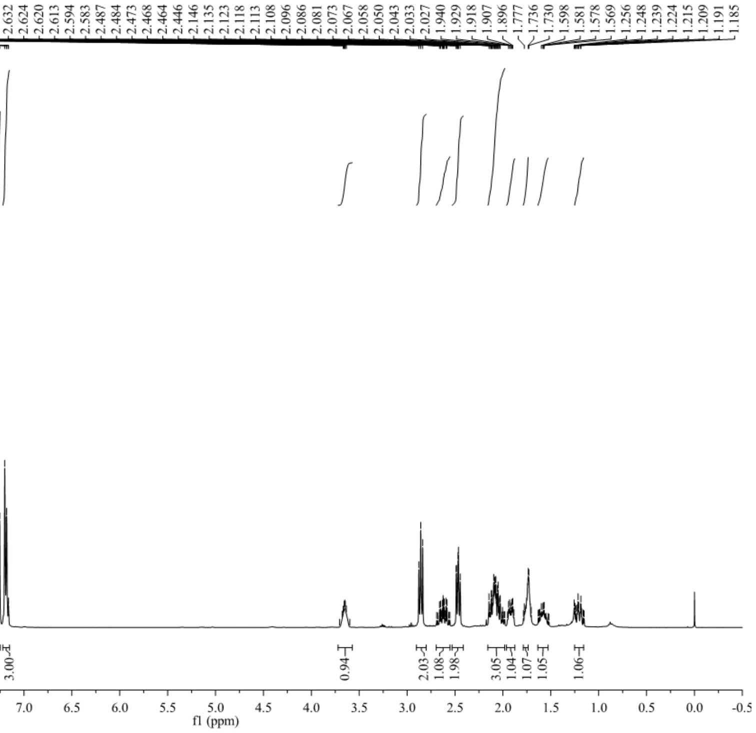

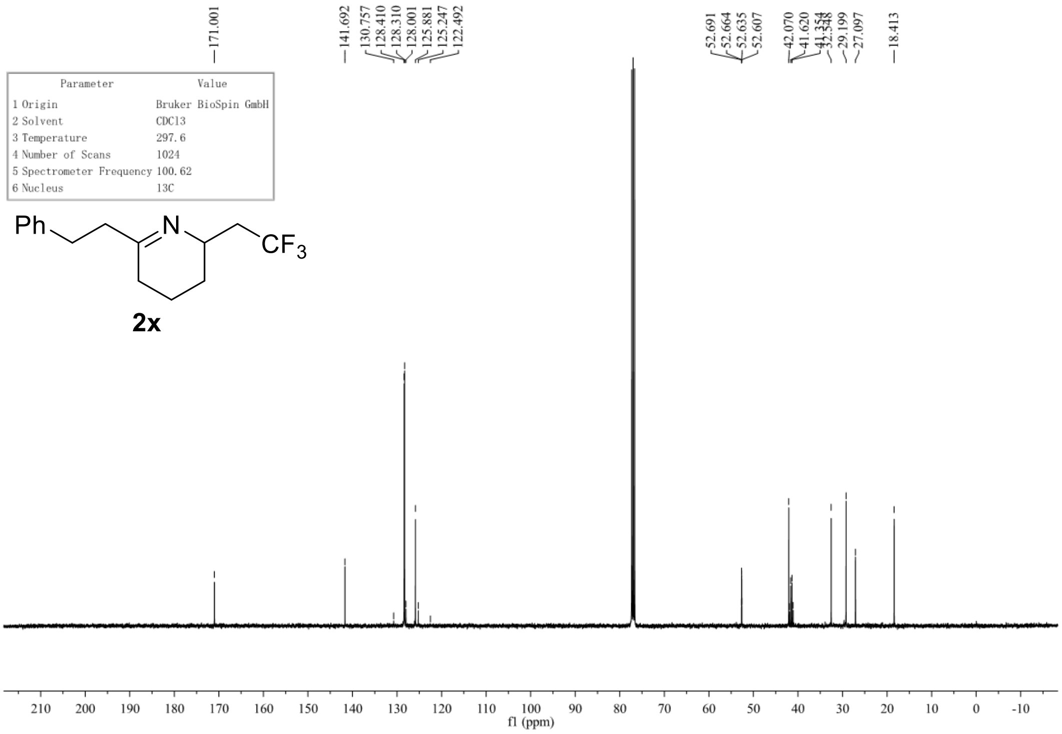

Parameter	Value
1 Origin	Bruker BioSpin GmbH
2 Solvent	CDCl3
3 Temperature	297.6
4 Number of Scans	1024
5 Spectrometer Frequency	100.62
6 Nucleus	13C

2x

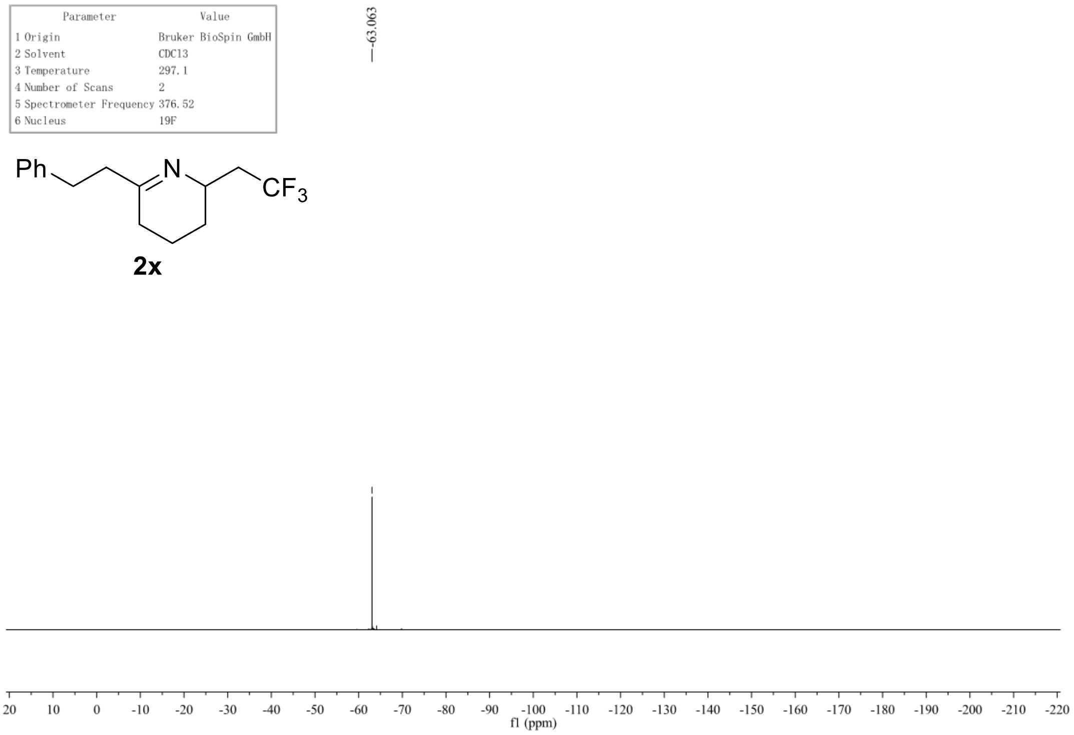

Parameter	Value
1 Origin	Bruker BioSpin GmbH
2 Solvent	CDCl3
3 Temperature	297.1
4 Number of Scans	2
5 Spectrometer Frequency	376.52
6 Nucleus	19F

2x

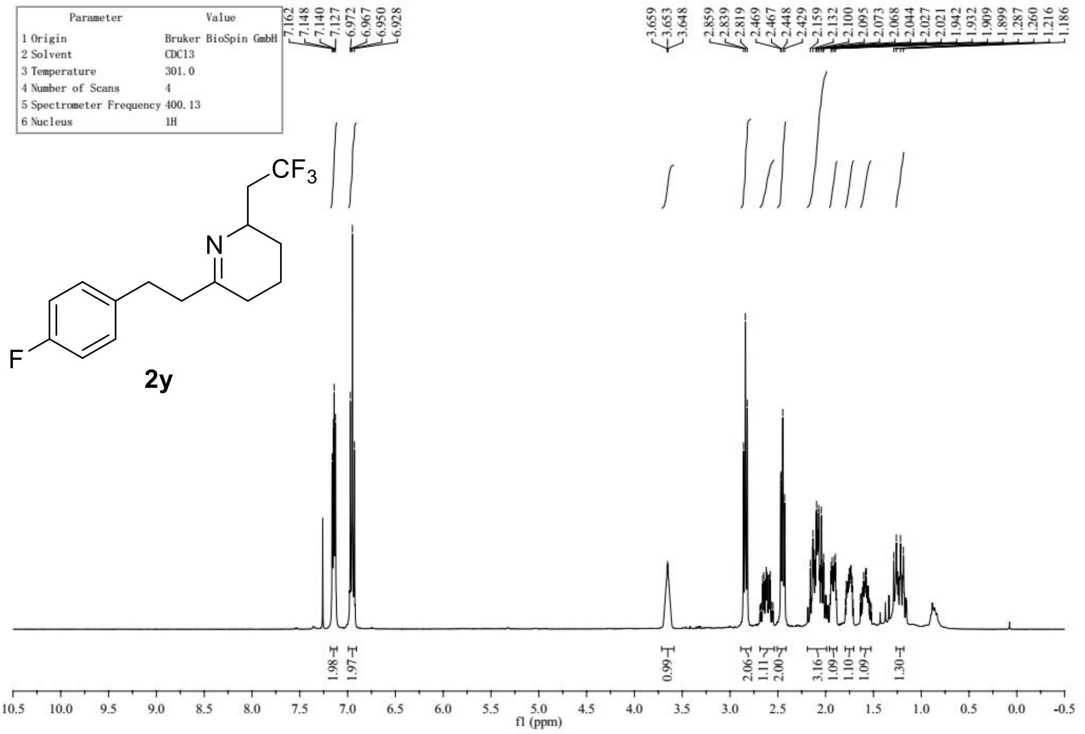
Parameter Value
1 Origin Bruker BioSpin GmbH
2 Solvent CDCl3
3 Temperature 301.0
4 Number of Scans 4
5 Spectrometer Frequency 400.13
6 Nucleus 1H
CF3
N
F
2y
f1 (ppm)

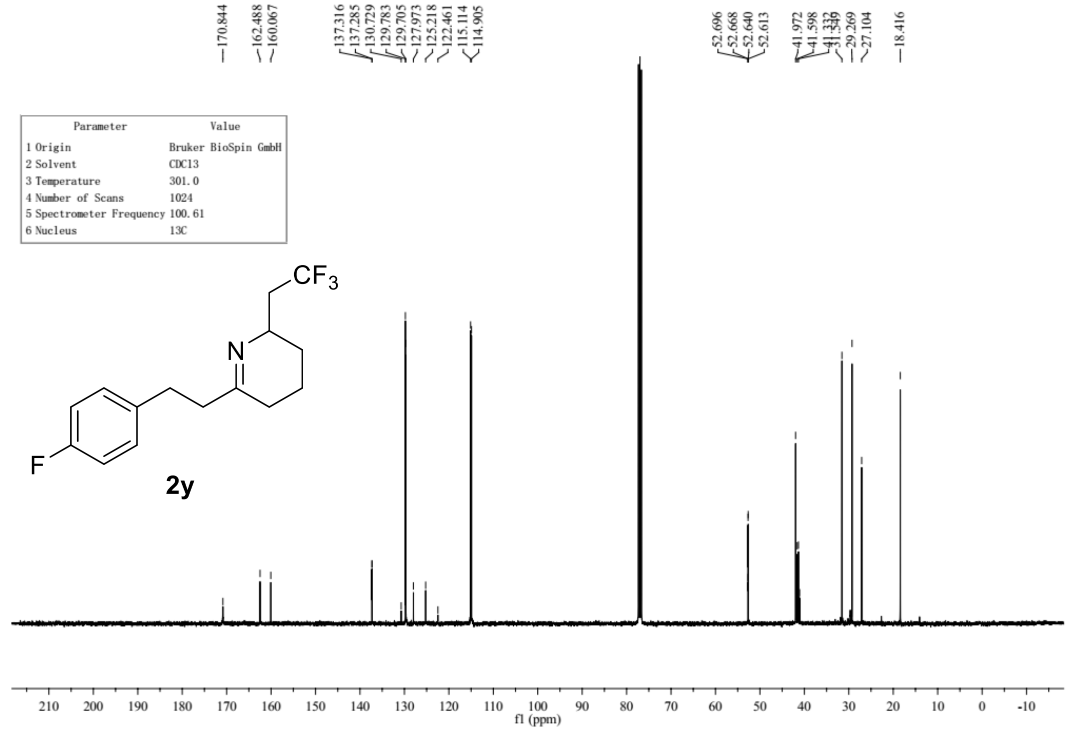
Parameter Value
1 Origin Bruker BioSpin GmbH
2 Solvent CDC13
3 Temperature 301.0
4 Number of Scans 1024
5 Spectrometer Frequency 100.61
6 Nucleus 13C
CF3
N
F
2y
f1 (ppm)

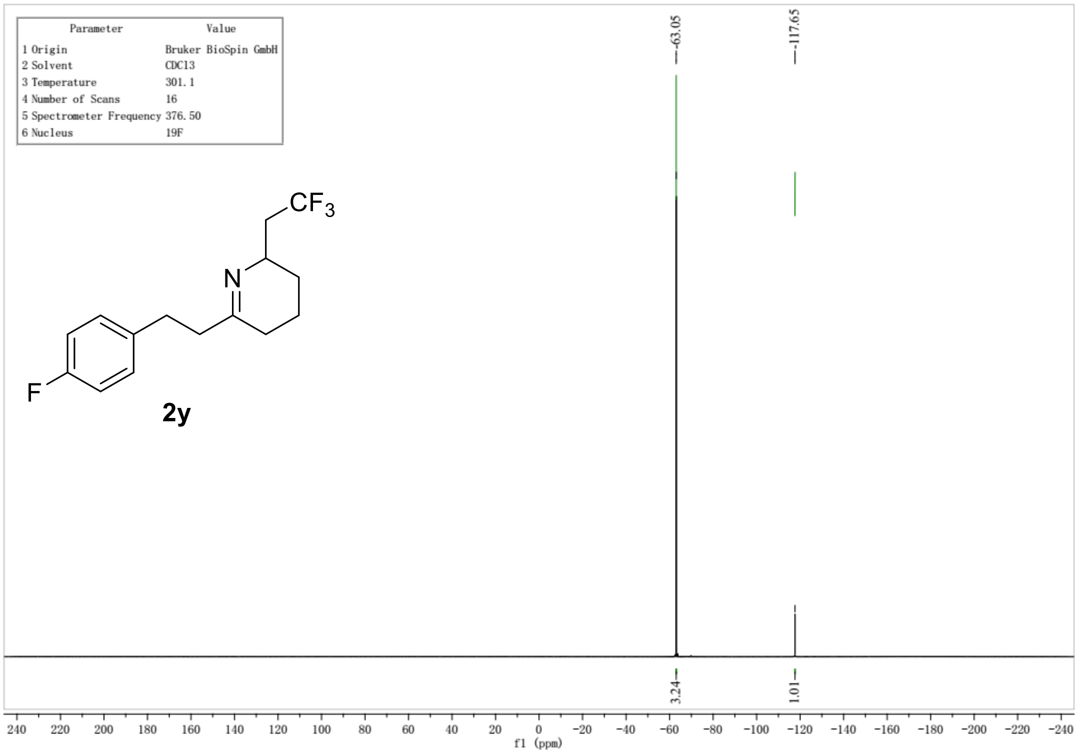
Parameter Value
1 Origin Bruker BioSpin GmbH
2 Solvent CDCl3
3 Temperature 301.1
4 Number of Scans 16
5 Spectrometer Frequency 376.50
6 Nucleus 19F
-63.05
-117.65
CF3
N
F
2y
3.24
1.01
f1 (ppm)

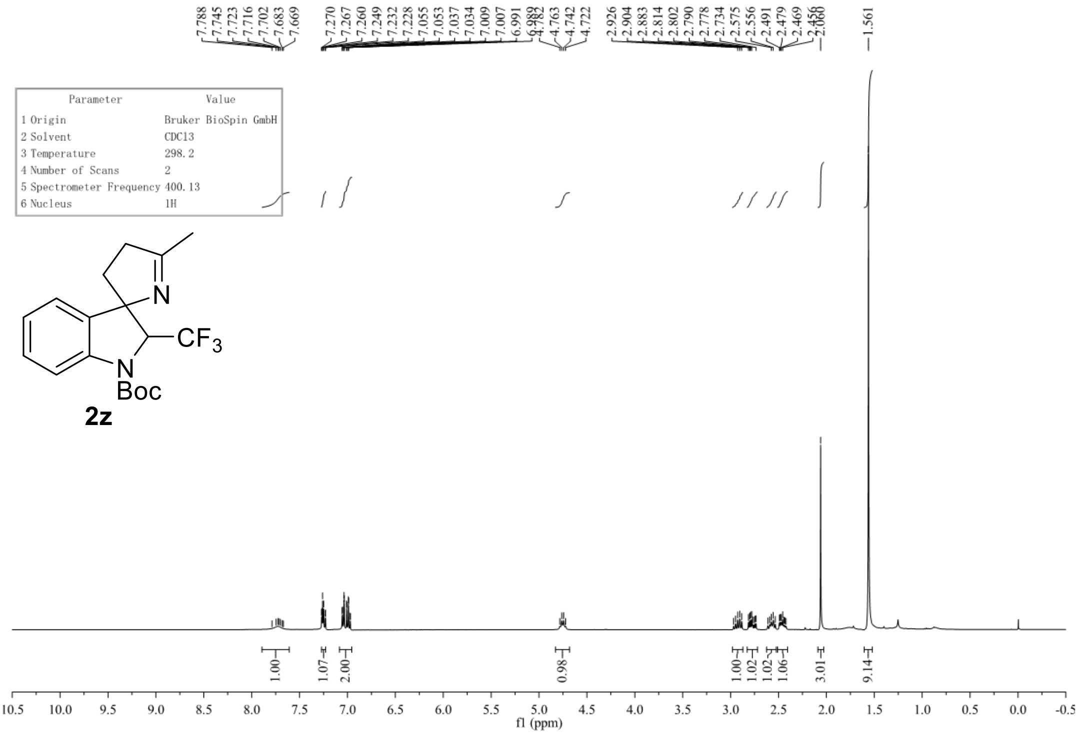
Parameter Value
1 Origin Bruker BioSpin GmbH
2 Solvent CDCl3
3 Temperature 298.2
4 Number of Scans 2
5 Spectrometer Frequency 400.13
6 Nucleus 1H
-1.561
N
CF3
N
Boc
2z
1.00
1.07
2.00
0.98
1.00
1.02
1.02
1.06
3.01
9.14
f1 (ppm)

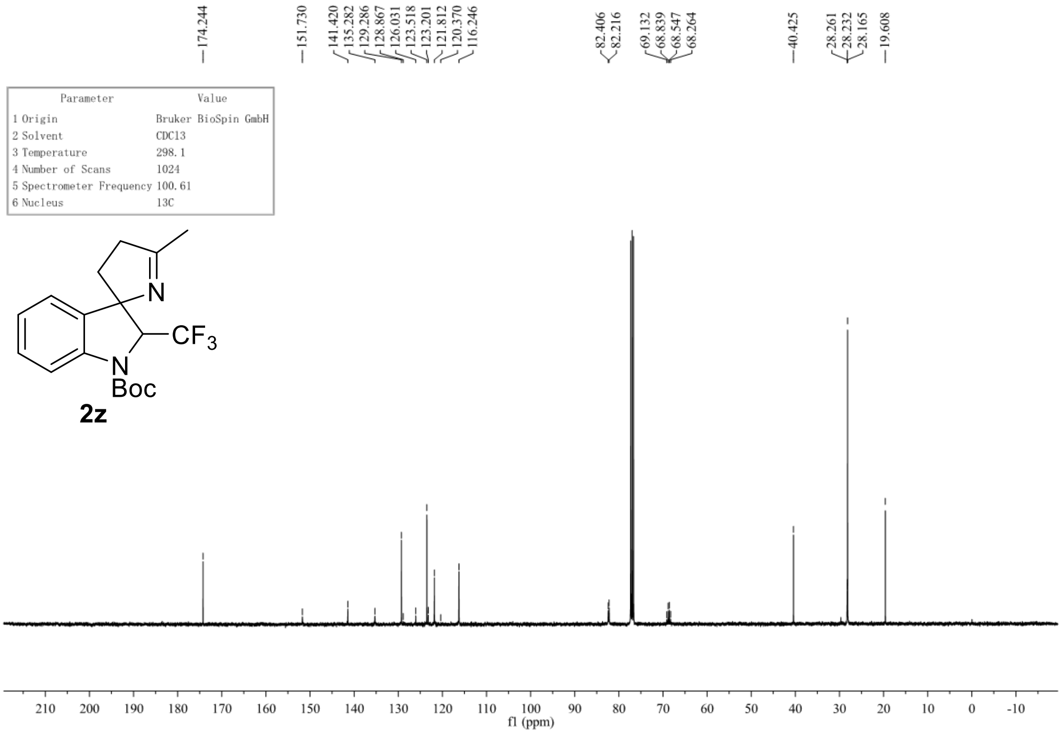

Parameter	Value
1 Origin	Bruker BioSpin GmbH
2 Solvent	CDCl3
3 Temperature	298.1
4 Number of Scans	1024
5 Spectrometer Frequency	100.61
6 Nucleus	13C

2z

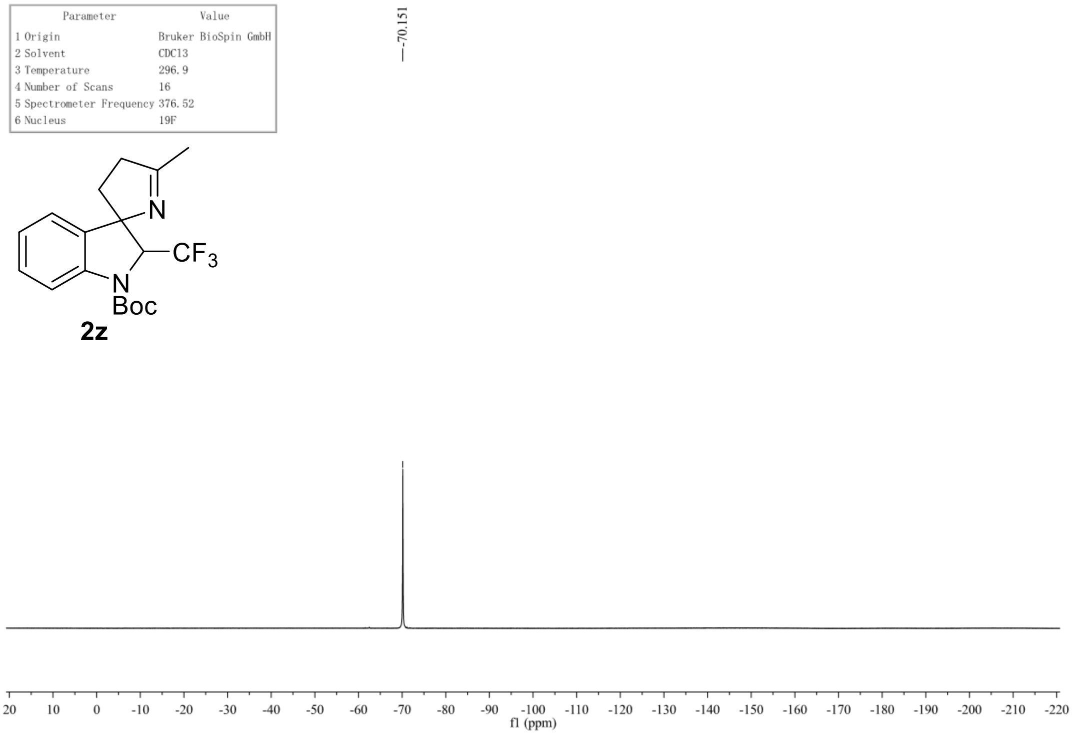

Parameter	Value
1 Origin	Bruker BioSpin GmbH
2 Solvent	CDCl3
3 Temperature	296.9
4 Number of Scans	16
5 Spectrometer Frequency	376.52
6 Nucleus	19F

2z

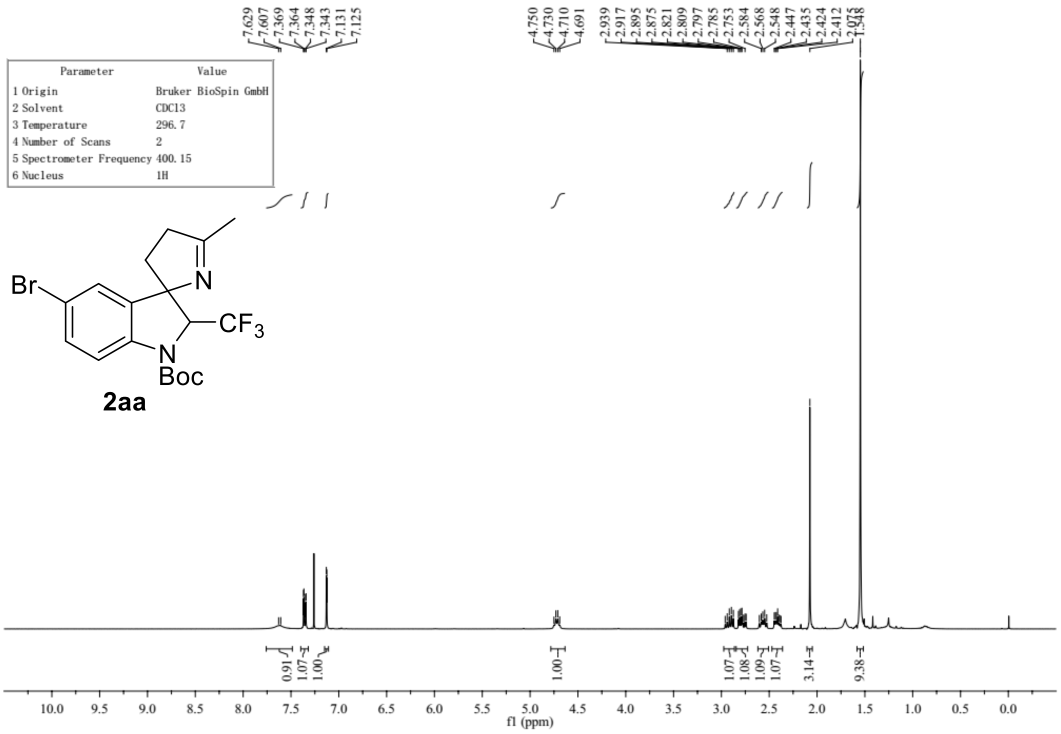
7.629
7.607
7.369
7.364
7.348
7.343
7.131
7.125
4.750
4.730
4.710
4.691
2.939
2.917
2.895
2.875
2.821
2.809
2.797
2.785
2.753
2.584
2.568
2.548
2.447
2.435
2.424
2.412
2.075
1.548
Parameter Value
1 Origin Bruker BioSpin GmbH
2 Solvent CDCl3
3 Temperature 296.7
4 Number of Scans 2
5 Spectrometer Frequency 400.15
6 Nucleus 1H
Br
N
CF3
N
Boc
2aa
0.91
1.07
1.00
1.00
1.07
1.08
1.09
1.07
3.14
9.38
10.0 9.5 9.0 8.5 8.0 7.5 7.0 6.5 6.0 5.5 5.0 4.5 4.0 3.5 3.0 2.5 2.0 1.5 1.0 0.5 0.0
f1 (ppm)

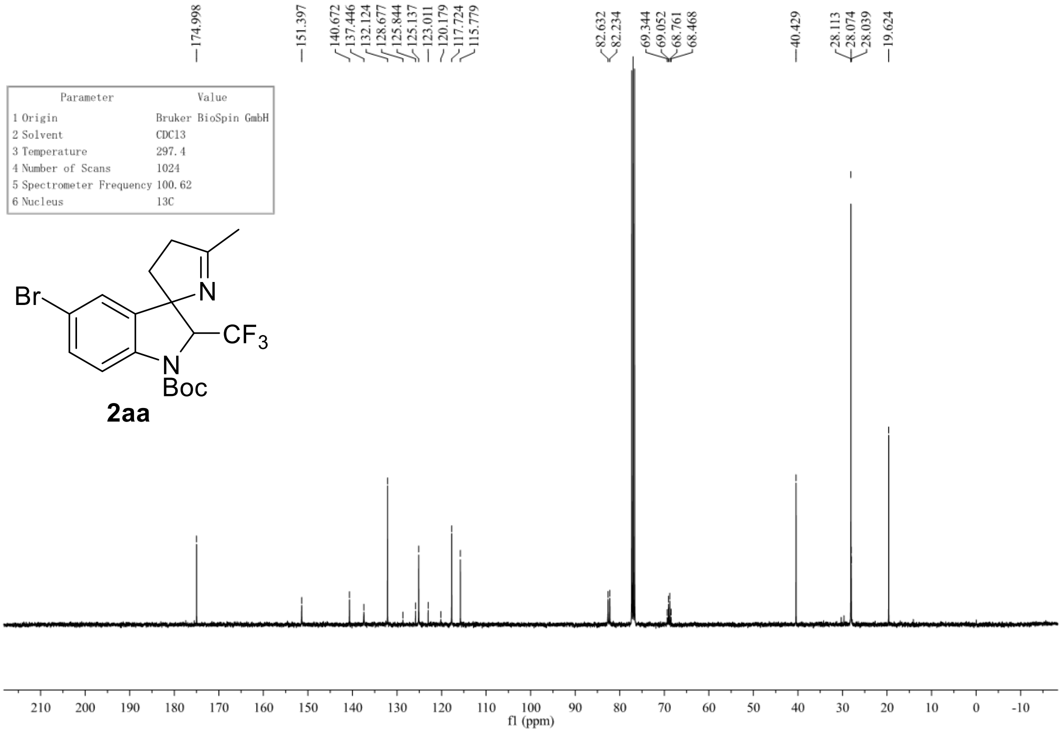
174.998
151.397
140.672
137.446
132.124
128.677
125.844
125.137
123.011
120.179
117.724
115.779
82.632
82.234
69.344
69.052
68.761
68.468
40.429
28.113
28.074
28.039
19.624
Parameter Value
1 Origin Bruker BioSpin GmbH
2 Solvent CDCl3
3 Temperature 297.4
4 Number of Scans 1024
5 Spectrometer Frequency 100.62
6 Nucleus 13C
Br
N
CF3
N
Boc
2aa
210 200 190 180 170 160 150 140 130 120 110 100 90 80 70 60 50 40 30 20 10 0 -10
f1 (ppm)

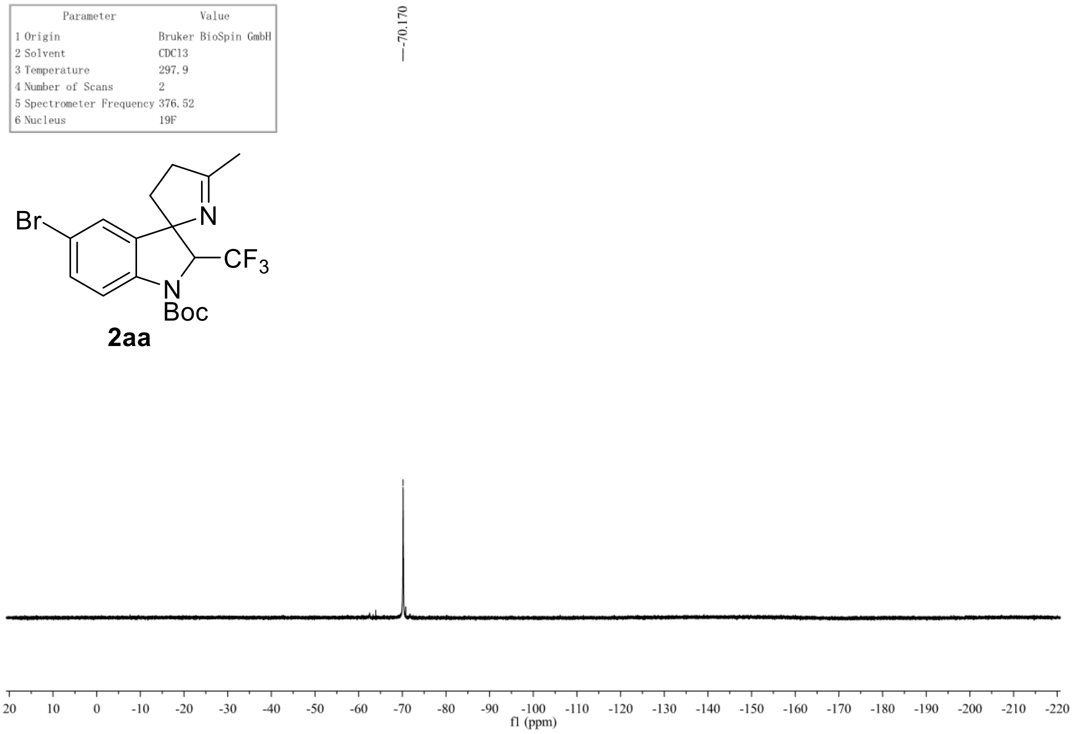
Parameter Value
1 Origin Bruker BioSpin GmbH
2 Solvent CDCl3
3 Temperature 297.9
4 Number of Scans 2
5 Spectrometer Frequency 376.52
6 Nucleus 19F
-70.170
Br
N
CF3
N
Boc
2aa
fl (ppm)

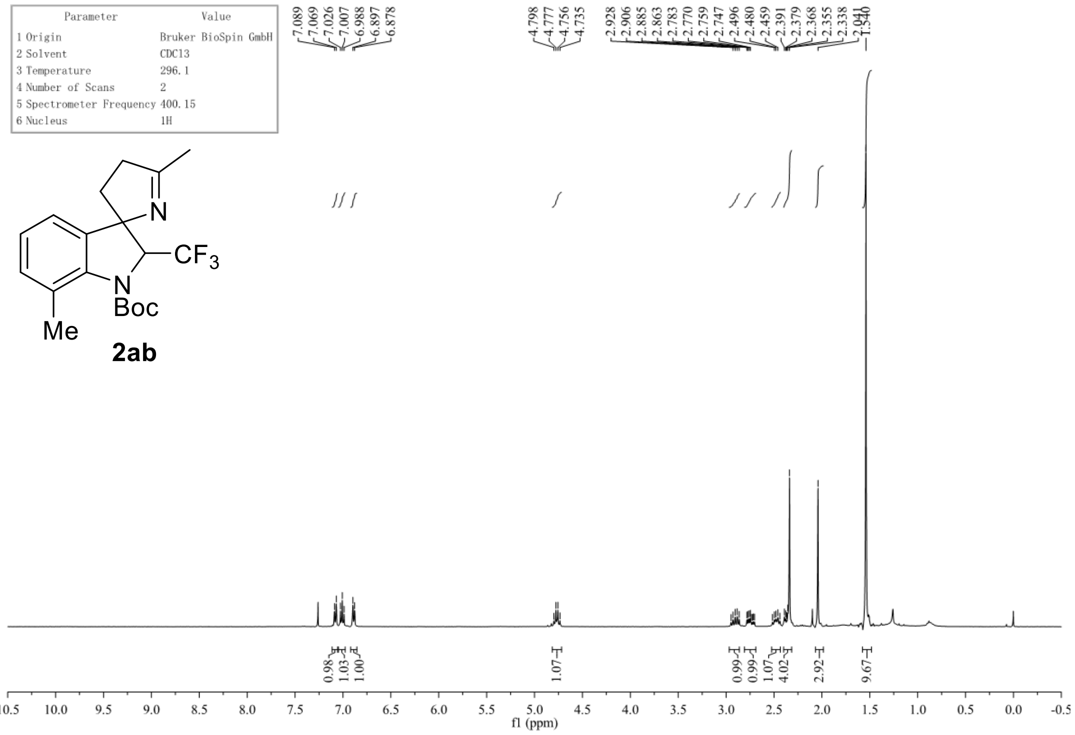
Parameter Value
1 Origin Bruker BioSpin GmbH
2 Solvent CDCl3
3 Temperature 296.1
4 Number of Scans 2
5 Spectrometer Frequency 400.15
6 Nucleus 1H
7.089
7.069
7.026
7.007
6.988
6.897
6.878
4.798
4.777
4.756
4.735
2.928
2.906
2.885
2.863
2.783
2.770
2.759
2.747
2.496
2.480
2.459
2.391
2.379
2.368
2.355
2.338
2.041
1.540
N
CF3
N
Boc
Me
2ab
0.98
1.03
1.00
1.07
0.99
0.99
1.07
4.02
2.92
9.67
fl (ppm)

Parameter	Value
1 Origin	Bruker BioSpin GmbH
2 Solvent	CDCl3
3 Temperature	298.1
4 Number of Scans	1024
5 Spectrometer Frequency	100.62
6 Nucleus	13C

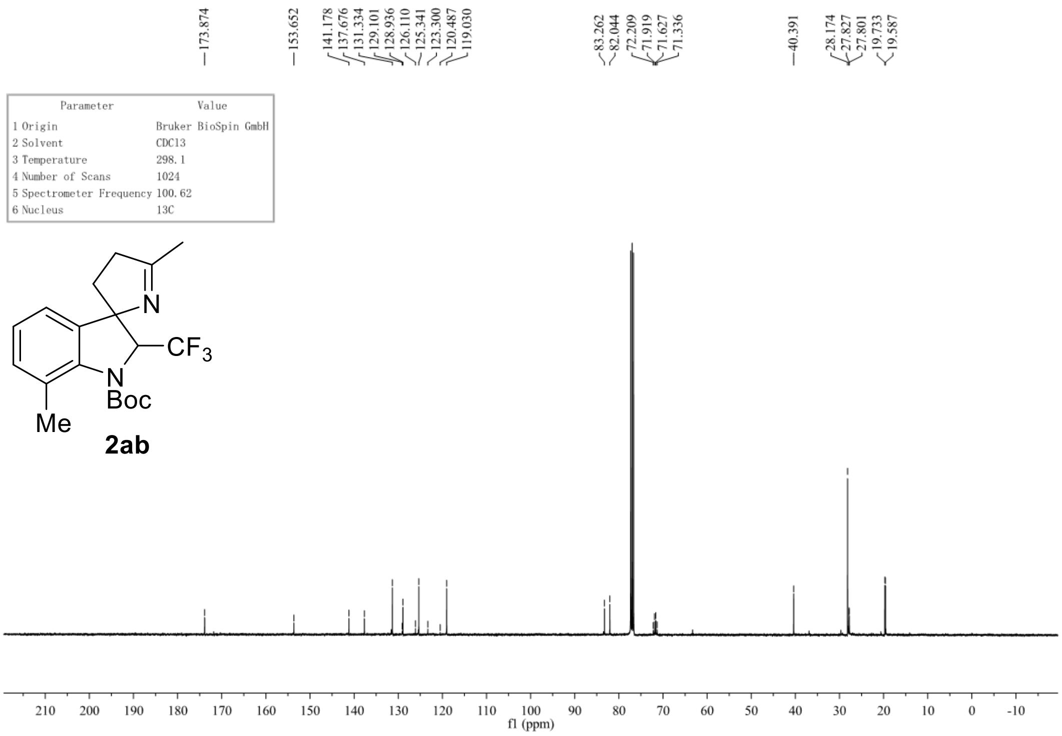

2ab

Parameter	Value
1 Origin	Bruker BioSpin GmbH
2 Solvent	CDCl3
3 Temperature	296.1
4 Number of Scans	2
5 Spectrometer Frequency	376.52
6 Nucleus	19F

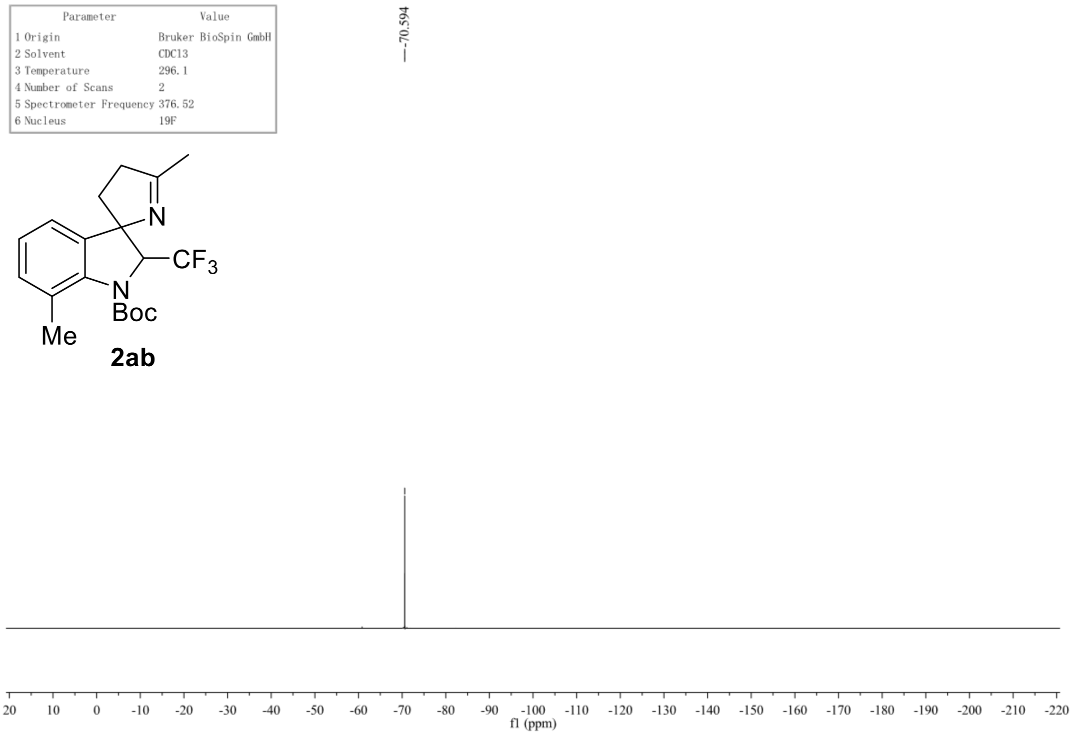

2ab

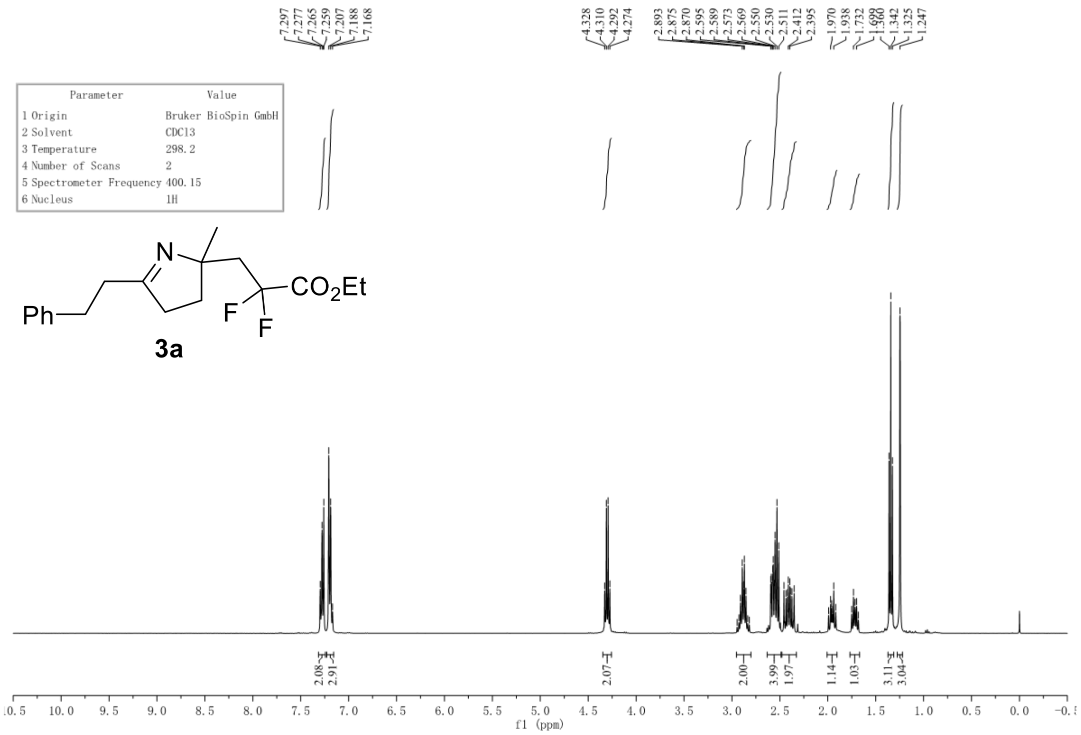

Parameter Value
1 Origin Bruker BioSpin GmbH
2 Solvent CDCl3
3 Temperature 298.2
4 Number of Scans 2
5 Spectrometer Frequency 400.15
6 Nucleus 1H
N
CO2Et
Ph
F
F
3a
f1 (ppm)

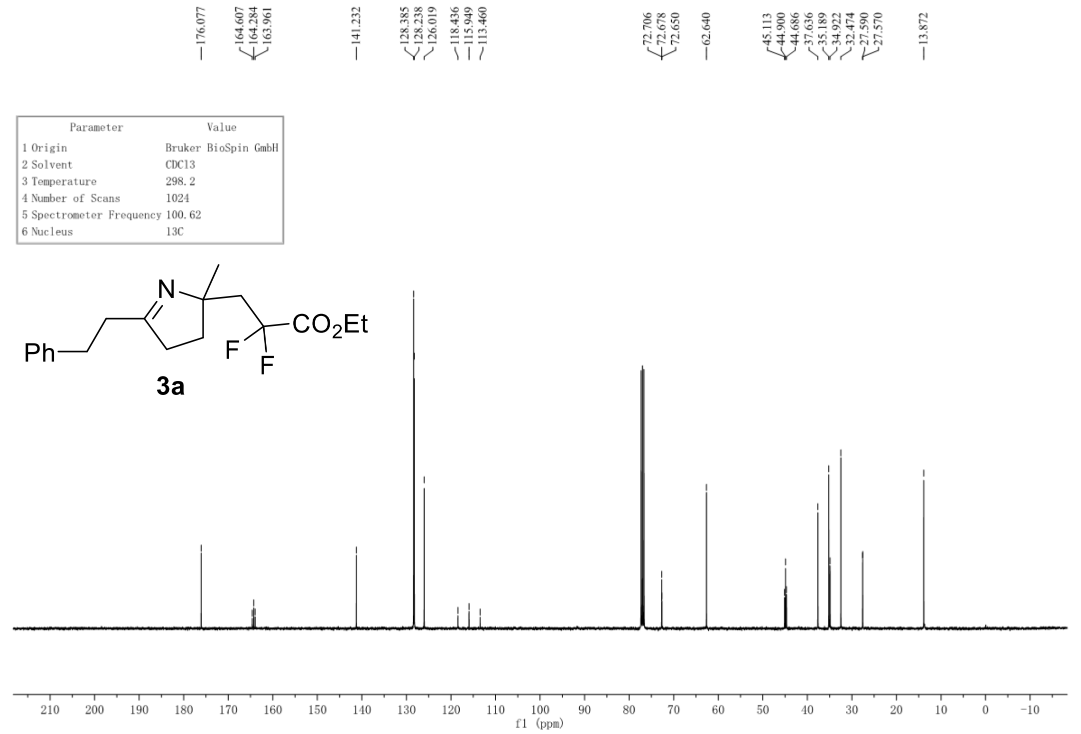

Parameter Value
1 Origin Bruker BioSpin GmbH
2 Solvent CDCl3
3 Temperature 298.2
4 Number of Scans 1024
5 Spectrometer Frequency 100.62
6 Nucleus 13C
N
CO2Et
Ph
F
F
3a
f1 (ppm)

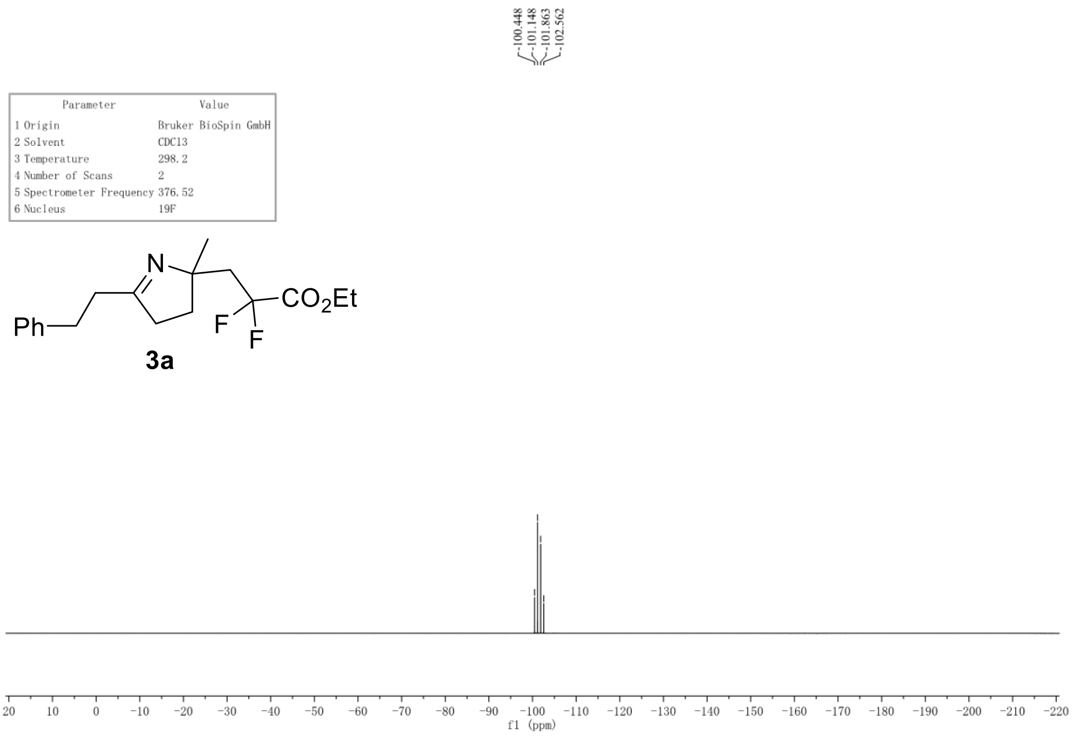
-100.448
-101.148
-101.863
-102.562
Parameter Value
1 Origin Bruker BioSpin GmbH
2 Solvent CDCl3
3 Temperature 298.2
4 Number of Scans 2
5 Spectrometer Frequency 376.52
6 Nucleus 19F
N
CO2Et
Ph
F
F
3a
20 10 0 −10 −20 −30 −40 −50 −60 −70 −80 −90 −100 −110 −120 −130 −140 −150 −160 −170 −180 −190 −200 −210 −220
f1 (ppm)

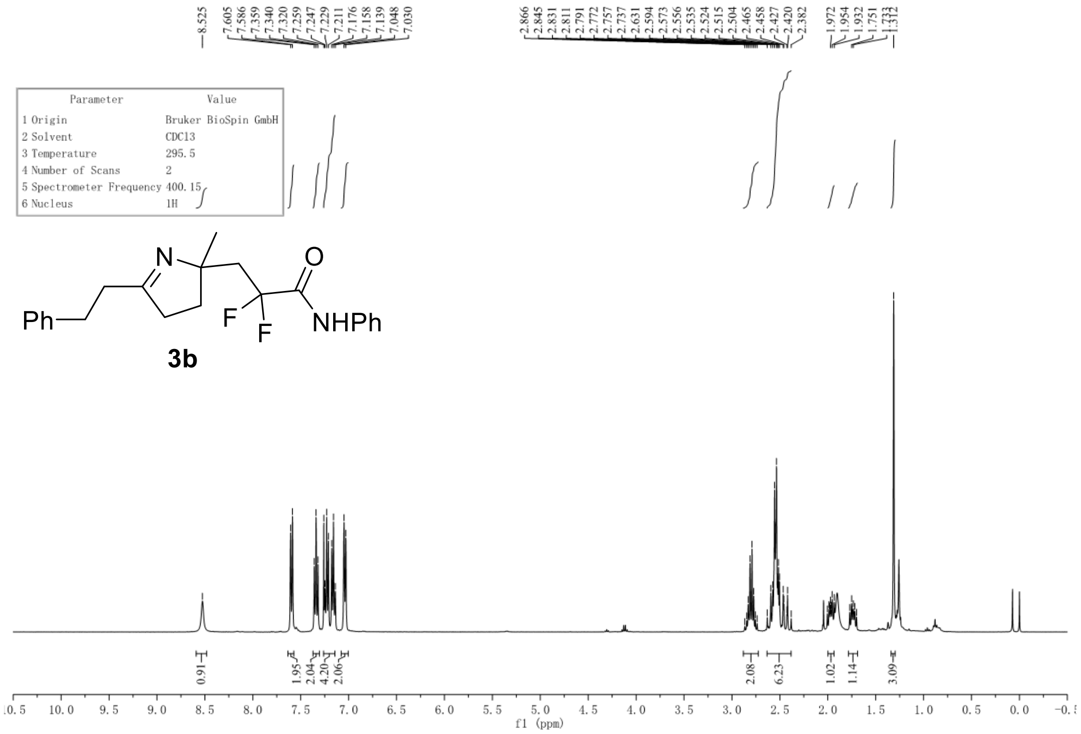
8.525
7.605 7.586 7.359 7.340 7.320 7.259 7.247 7.229 7.211 7.176 7.158 7.139 7.048 7.030
2.866 2.845 2.831 2.811 2.791 2.772 2.757 2.737 2.631 2.594 2.573 2.556 2.535 2.524 2.515 2.504 2.465 2.458 2.427 2.420 2.382
1.972 1.954 1.932 1.751 1.733 1.312
Parameter Value
1 Origin Bruker BioSpin GmbH
2 Solvent CDCl3
3 Temperature 295.5
4 Number of Scans 2
5 Spectrometer Frequency 400.15
6 Nucleus 1H
N
O
Ph
F
F
NHPh
3b
0.91 1.95 2.04 4.20 2.06 2.08 6.23 1.02 1.14 3.09
10.5 10.0 9.5 9.0 8.5 8.0 7.5 7.0 6.5 6.0 5.5 5.0 4.5 4.0 3.5 3.0 2.5 2.0 1.5 1.0 0.5 0.0 −0.5
f1 (ppm)

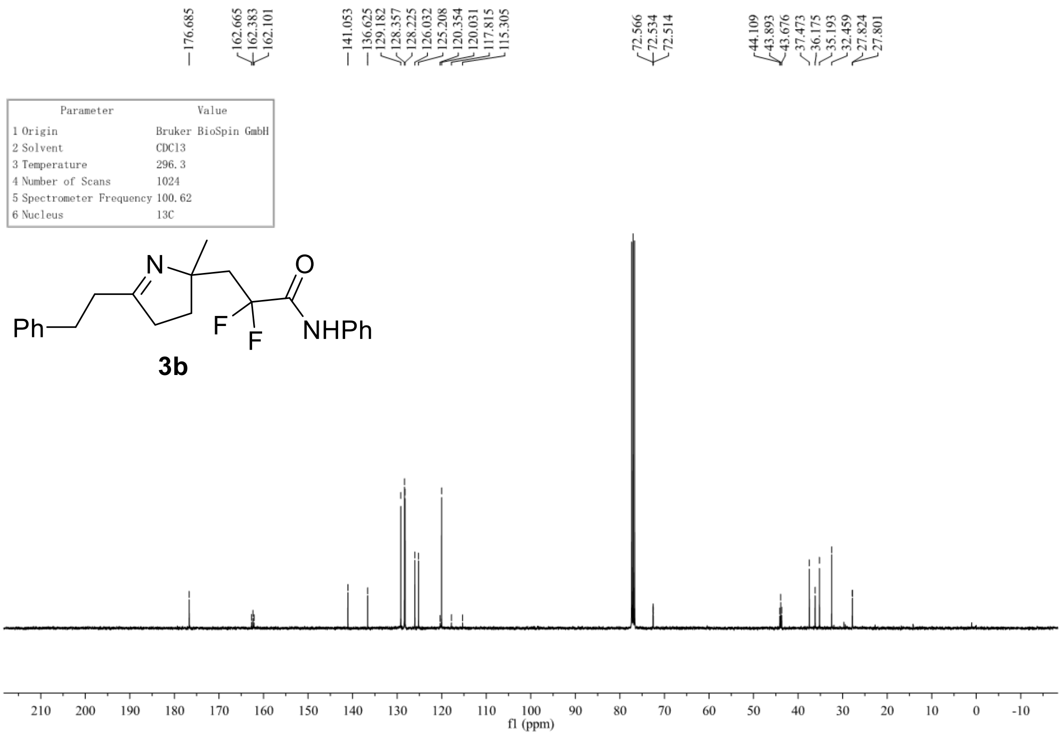
176.685
162.665
162.383
162.101
141.053
136.625
129.182
128.357
128.225
126.032
125.208
120.354
120.031
117.815
115.305
72.566
72.534
72.514
44.109
43.893
43.676
37.473
36.175
35.193
32.459
27.824
27.801
Parameter Value
1 Origin Bruker BioSpin GmbH
2 Solvent CDCl3
3 Temperature 296.3
4 Number of Scans 1024
5 Spectrometer Frequency 100.62
6 Nucleus 13C
N
O
Ph
F
F
NHPh
3b
f1 (ppm)

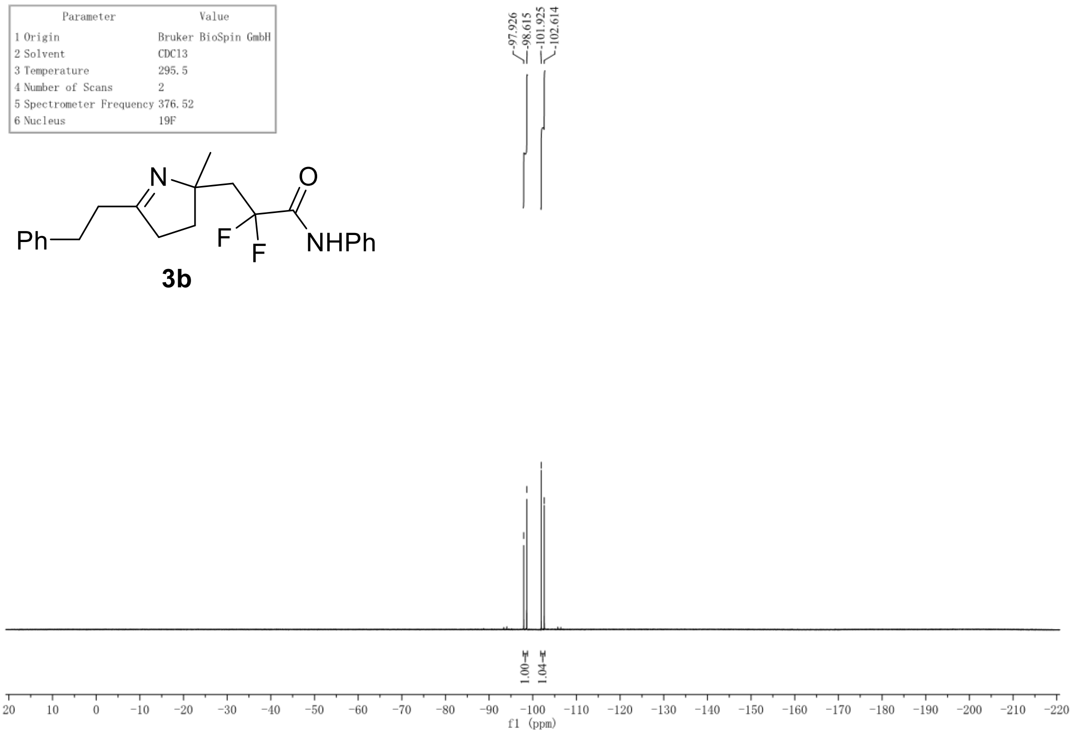
Parameter Value
1 Origin Bruker BioSpin GmbH
2 Solvent CDCl3
3 Temperature 295.5
4 Number of Scans 2
5 Spectrometer Frequency 376.52
6 Nucleus 19F
-97.926
-98.615
-101.925
-102.614
N
O
Ph
F
F
NHPh
3b
1.00
1.04
f1 (ppm)

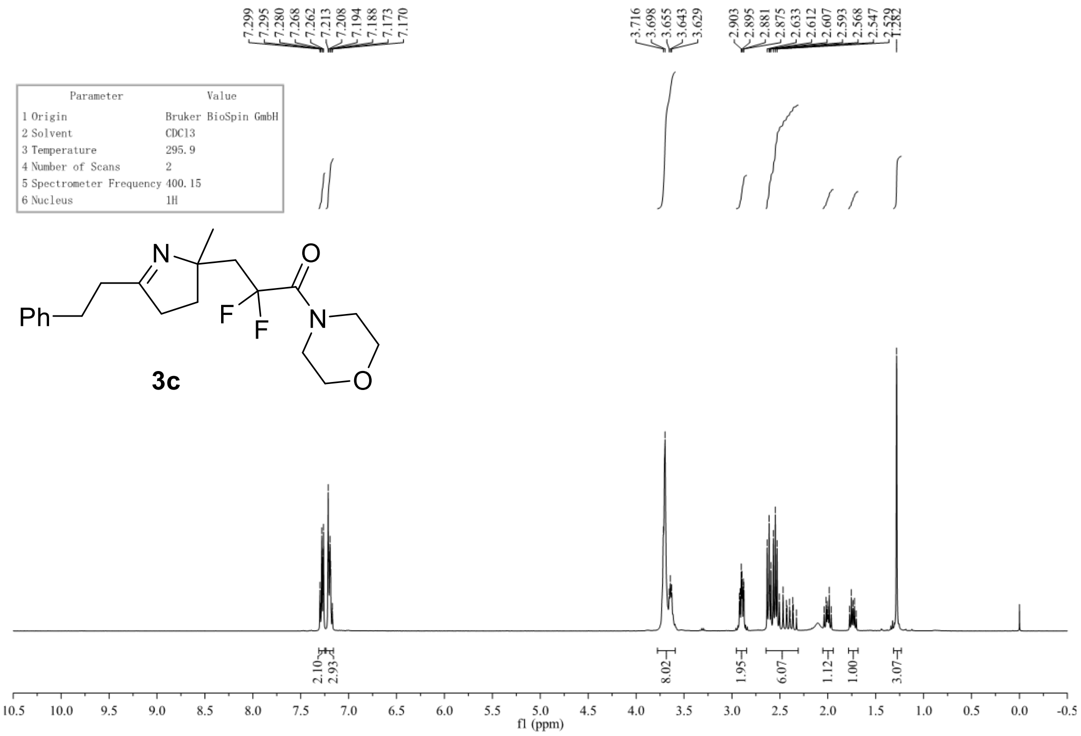
7.299
7.295
7.280
7.268
7.262
7.213
7.208
7.194
7.188
7.173
7.170
3.716
3.698
3.655
3.643
3.629
2.903
2.895
2.881
2.875
2.633
2.612
2.607
2.593
2.568
2.547
2.529
1.282
Parameter Value
1 Origin Bruker BioSpin GmbH
2 Solvent CDCl3
3 Temperature 295.9
4 Number of Scans 2
5 Spectrometer Frequency 400.15
6 Nucleus 1H
Ph
N
O
F
F
N
O
3c
2.10
2.93
8.02
1.95
6.07
1.12
1.00
3.07
fl (ppm)

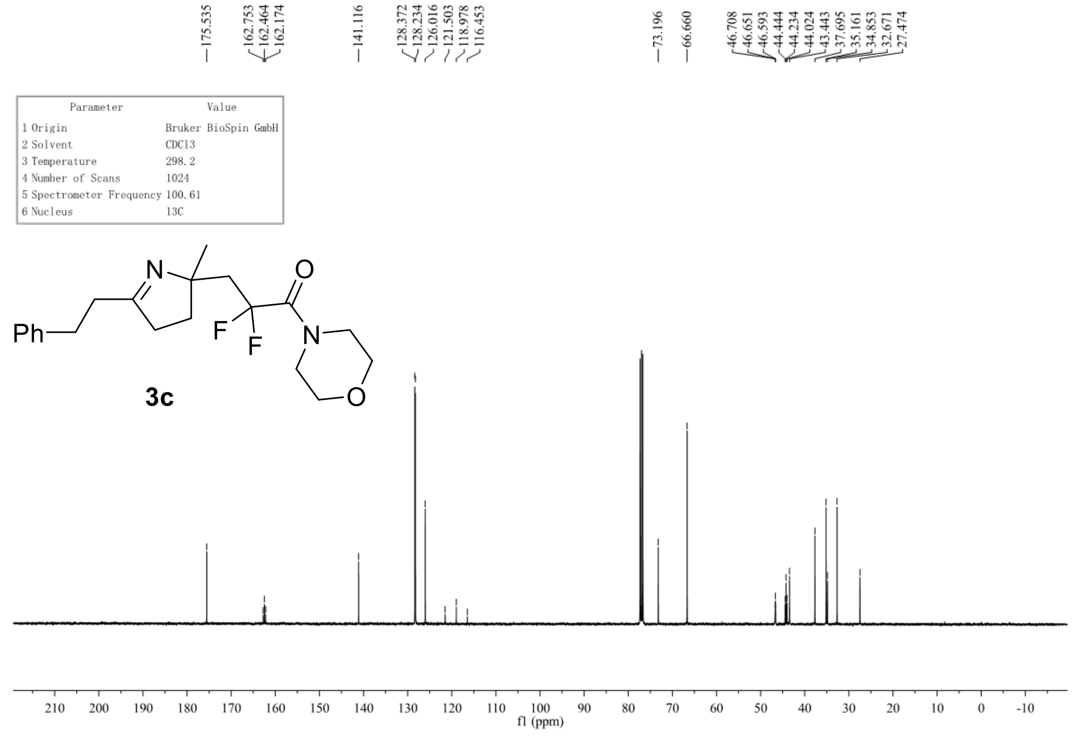
175.535
162.753
162.464
162.174
141.116
128.372
128.234
126.016
121.503
118.978
116.453
73.196
66.660
46.708
46.651
46.593
44.444
44.234
44.024
43.443
37.695
35.161
34.853
32.671
27.474
Parameter Value
1 Origin Bruker BioSpin GmbH
2 Solvent CDCl3
3 Temperature 298.2
4 Number of Scans 1024
5 Spectrometer Frequency 100.61
6 Nucleus 13C
Ph
N
O
F
F
N
O
3c
fl (ppm)

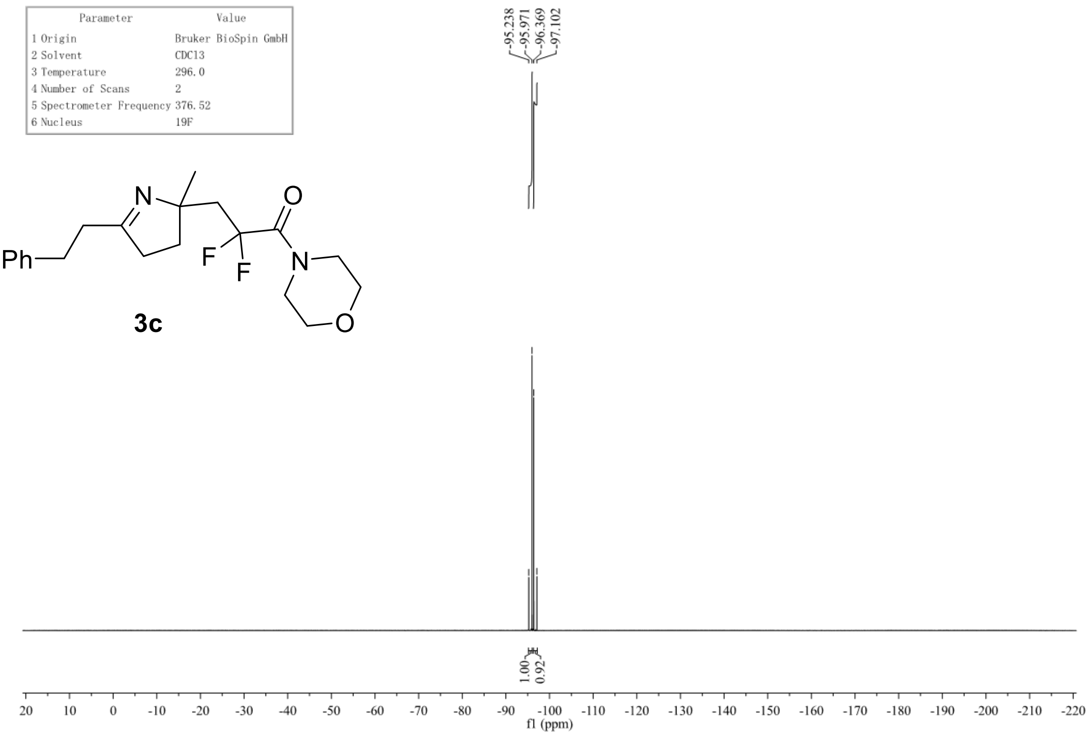

Parameter Value
1 Origin Bruker BioSpin GmbH
2 Solvent CDCl3
3 Temperature 296.0
4 Number of Scans 2
5 Spectrometer Frequency 376.52
6 Nucleus 19F
-95.238
-95.971
-96.369
-97.102
Ph
N
O
F
F
N
O
3c
1.00
0.92
f1 (ppm)

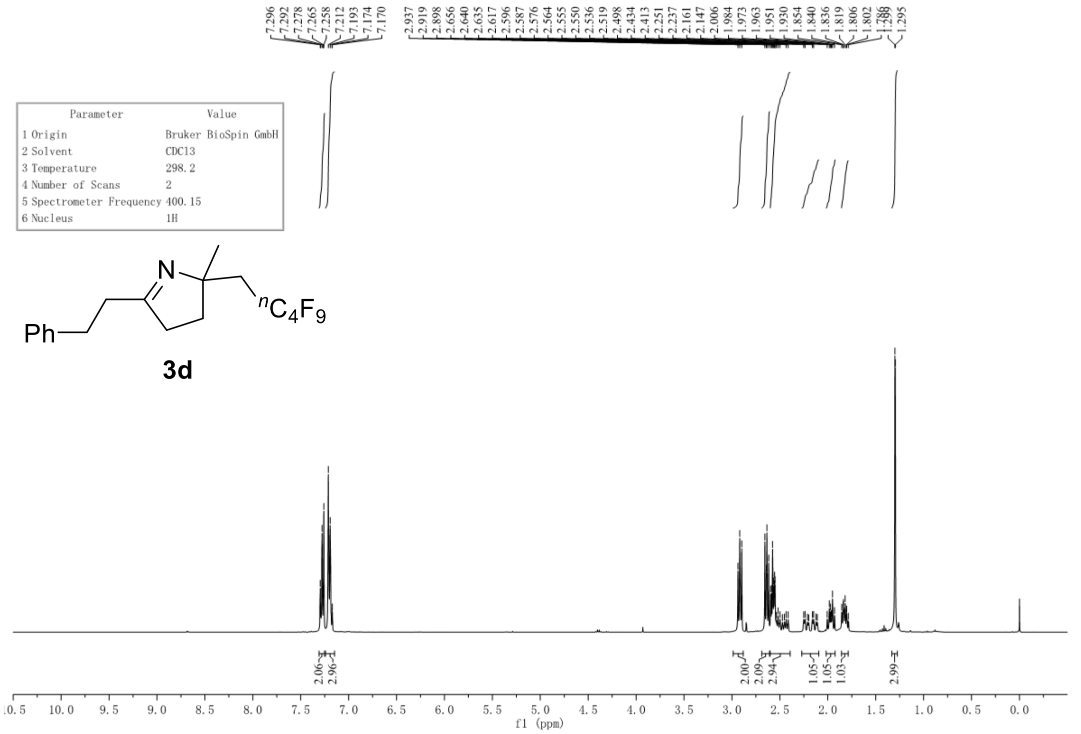

7.296
7.292
7.278
7.265
7.258
7.212
7.193
7.174
7.170
2.937
2.919
2.898
2.656
2.640
2.635
2.617
2.596
2.587
2.576
2.564
2.555
2.550
2.536
2.519
2.498
2.434
2.413
2.251
2.237
2.161
2.147
2.006
1.984
1.973
1.963
1.951
1.930
1.854
1.840
1.836
1.819
1.806
1.802
1.295
Parameter Value
1 Origin Bruker BioSpin GmbH
2 Solvent CDCl3
3 Temperature 298.2
4 Number of Scans 2
5 Spectrometer Frequency 400.15
6 Nucleus 1H
Ph
N
$^{n}C_4F_9$
3d
2.06
2.96
2.00
2.09
2.94
1.05
1.05
1.03
2.99
f1 (ppm)

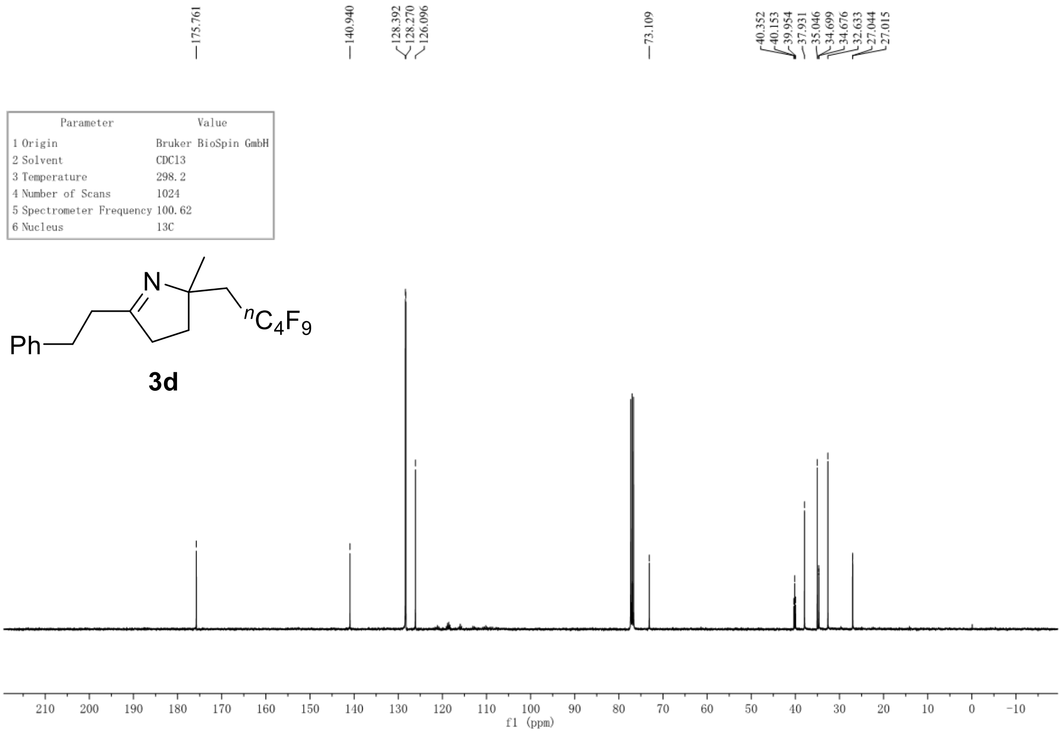

Parameter	Value
1 Origin	Bruker BioSpin GmbH
2 Solvent	CDCl3
3 Temperature	298.2
4 Number of Scans	1024
5 Spectrometer Frequency	100.62
6 Nucleus	13C

3d

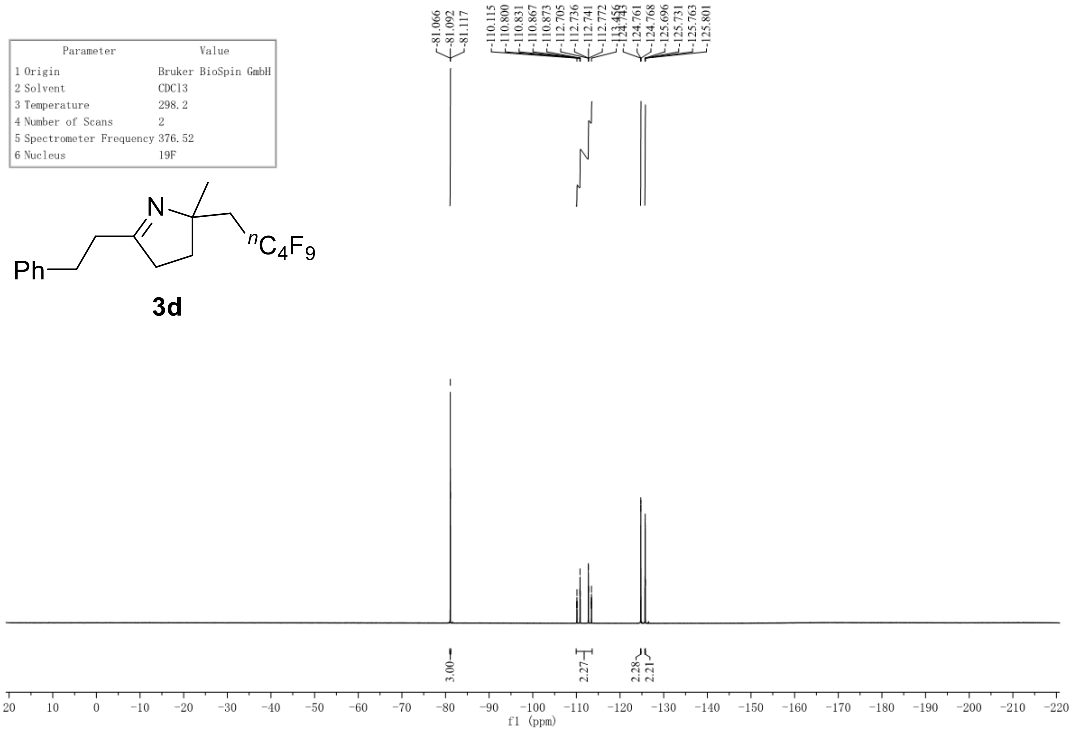

Parameter	Value
1 Origin	Bruker BioSpin GmbH
2 Solvent	CDCl3
3 Temperature	298.2
4 Number of Scans	2
5 Spectrometer Frequency	376.52
6 Nucleus	19F

3d

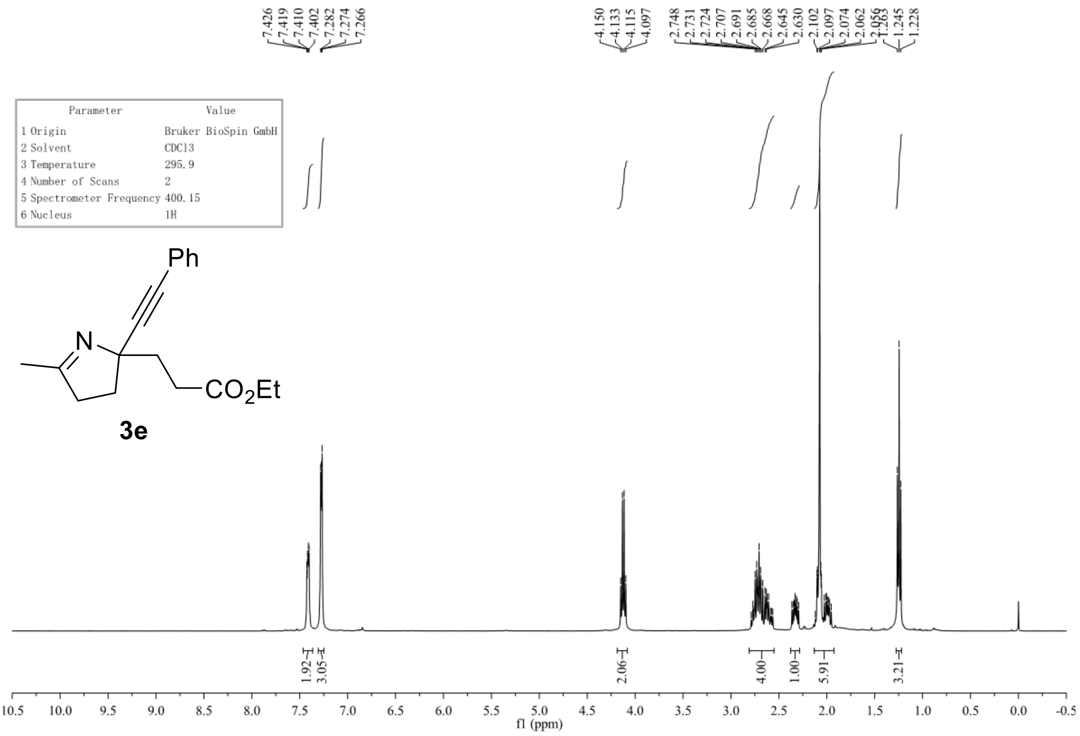

7.426 7.419 7.410 7.402 7.282 7.274 7.266
4.150 4.133 4.115 4.097
2.748 2.731 2.724 2.707 2.691 2.685 2.668 2.645 2.630 2.102 2.097 2.074 2.062 2.056 1.263 1.245 1.228
Parameter Value
1 Origin Bruker BioSpin GmbH
2 Solvent CDCl3
3 Temperature 295.9
4 Number of Scans 2
5 Spectrometer Frequency 400.15
6 Nucleus 1H
Ph
N
CO2Et
3e
1.92 3.05 2.06 4.00 1.00 5.91 3.21
10.5 10.0 9.5 9.0 8.5 8.0 7.5 7.0 6.5 6.0 5.5 5.0 4.5 4.0 3.5 3.0 2.5 2.0 1.5 1.0 0.5 0.0 -0.5
f1 (ppm)

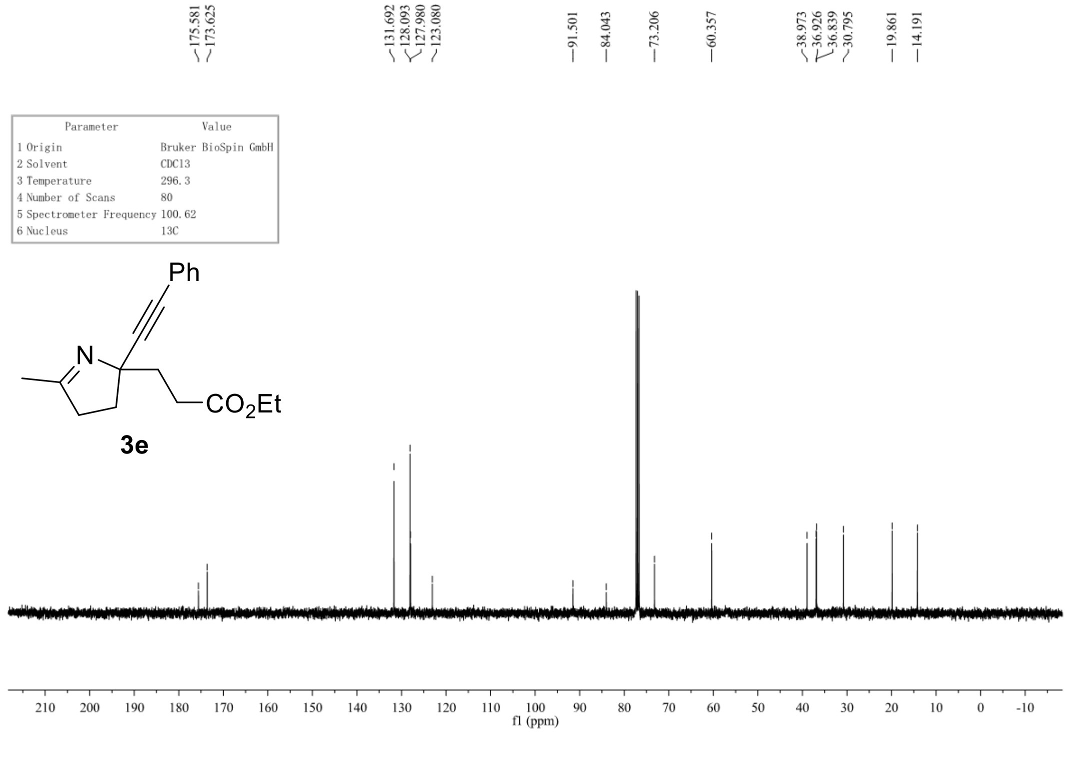

175.581 173.625
131.692 128.093 127.980 123.080
91.501 84.043 73.206 60.357
38.973 36.926 36.839 30.795 19.861 14.191
Parameter Value
1 Origin Bruker BioSpin GmbH
2 Solvent CDCl3
3 Temperature 296.3
4 Number of Scans 80
5 Spectrometer Frequency 100.62
6 Nucleus 13C
Ph
N
CO2Et
3e
210 200 190 180 170 160 150 140 130 120 110 100 90 80 70 60 50 40 30 20 10 0 -10
f1 (ppm)

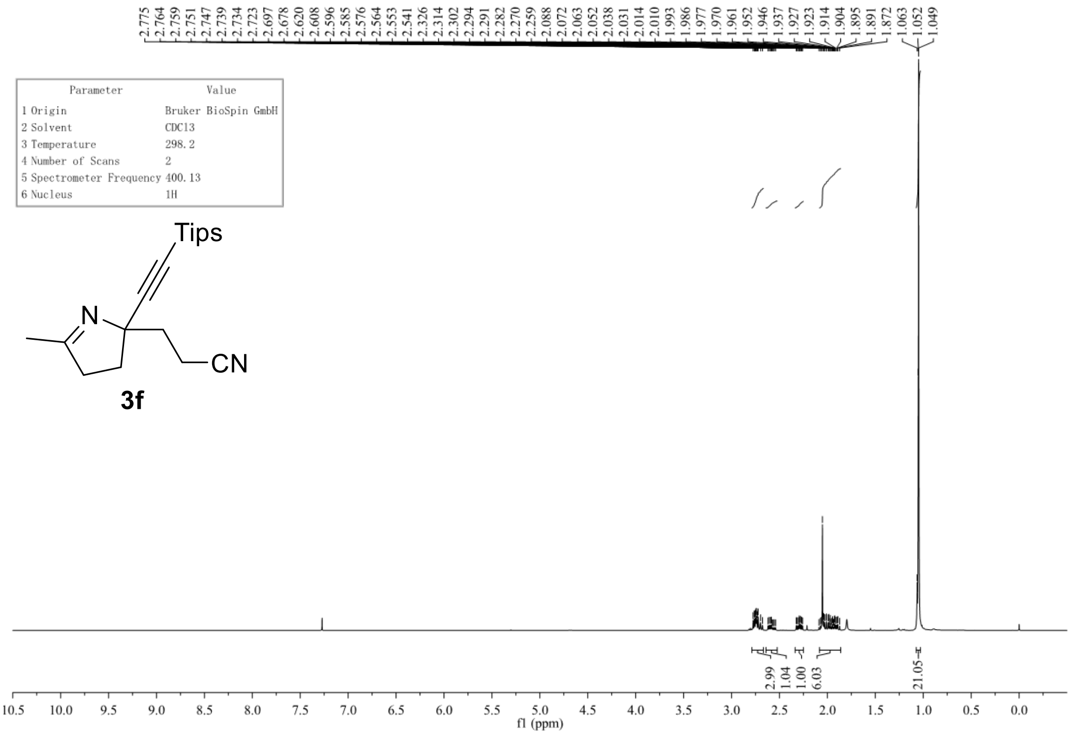

Parameter	Value
1 Origin	Bruker BioSpin GmbH
2 Solvent	CDCl3
3 Temperature	298.2
4 Number of Scans	2
5 Spectrometer Frequency	400.13
6 Nucleus	1H

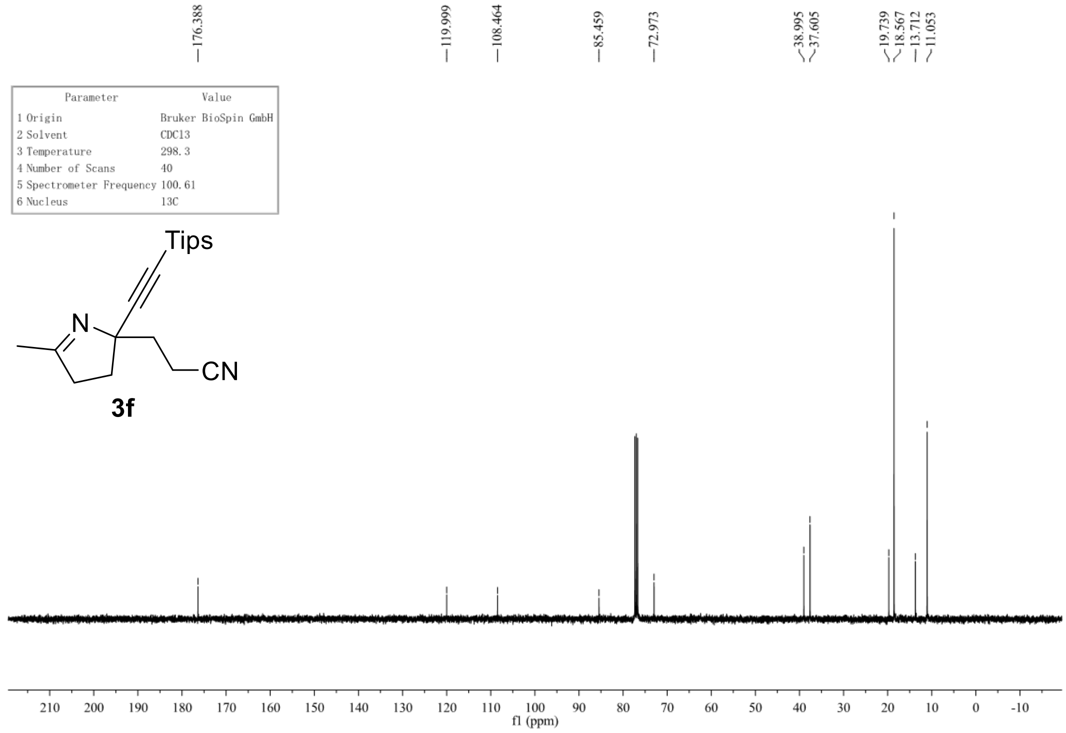

Parameter	Value
1 Origin	Bruker BioSpin GmbH
2 Solvent	CDCl3
3 Temperature	298.3
4 Number of Scans	40
5 Spectrometer Frequency	100.61
6 Nucleus	13C

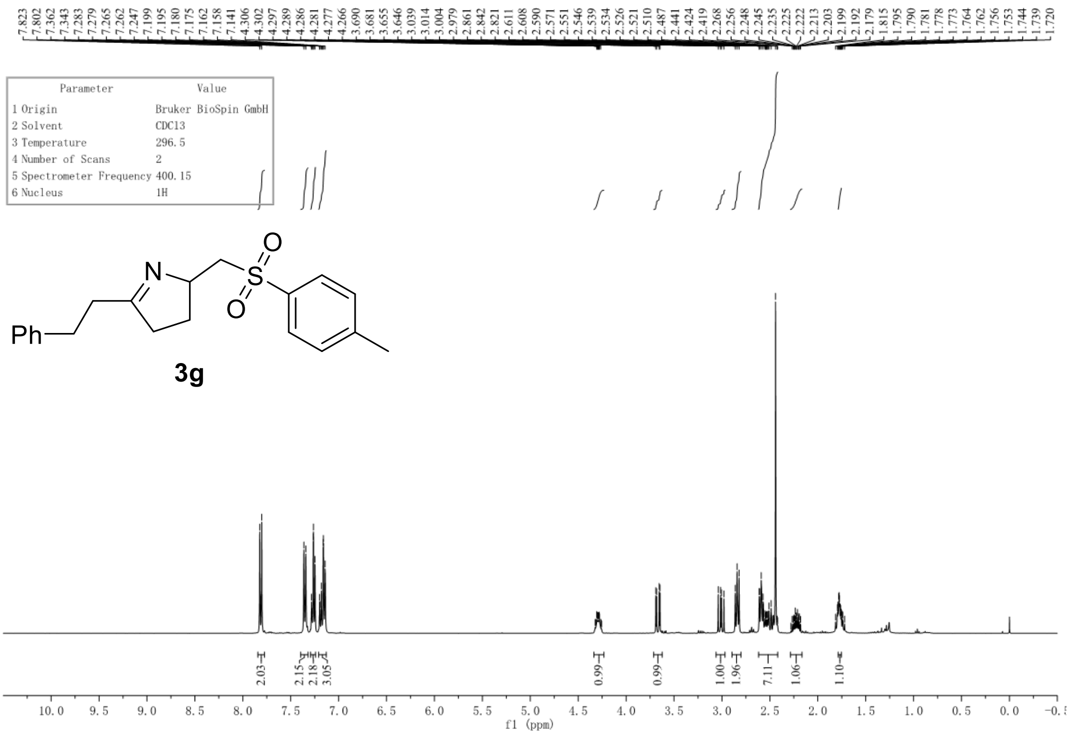
7.823 7.802 7.362 7.343 7.283 7.279 7.265 7.262 7.247 7.199 7.195 7.180 7.175 7.162 7.158 7.141 4.306 4.302 4.297 4.289 4.286 4.281 4.277 4.266 3.690 3.681 3.655 3.646 3.039 3.014 3.004 2.979 2.861 2.842 2.821 2.611 2.608 2.590 2.571 2.551 2.546 2.539 2.534 2.526 2.521 2.510 2.487 2.441 2.424 2.419 2.268 2.256 2.248 2.245 2.235 2.225 2.222 2.213 2.203 2.199 2.192 2.179 1.815 1.795 1.790 1.781 1.778 1.773 1.764 1.762 1.756 1.753 1.744 1.739 1.720
Parameter Value
1 Origin Bruker BioSpin GmbH
2 Solvent CDCl3
3 Temperature 296.5
4 Number of Scans 2
5 Spectrometer Frequency 400.15
6 Nucleus 1H
N
S
O
O
Ph
3g
2.03 2.15 2.18 3.05 0.99 0.99 1.00 1.96 7.11 1.06 1.10
f1 (ppm)

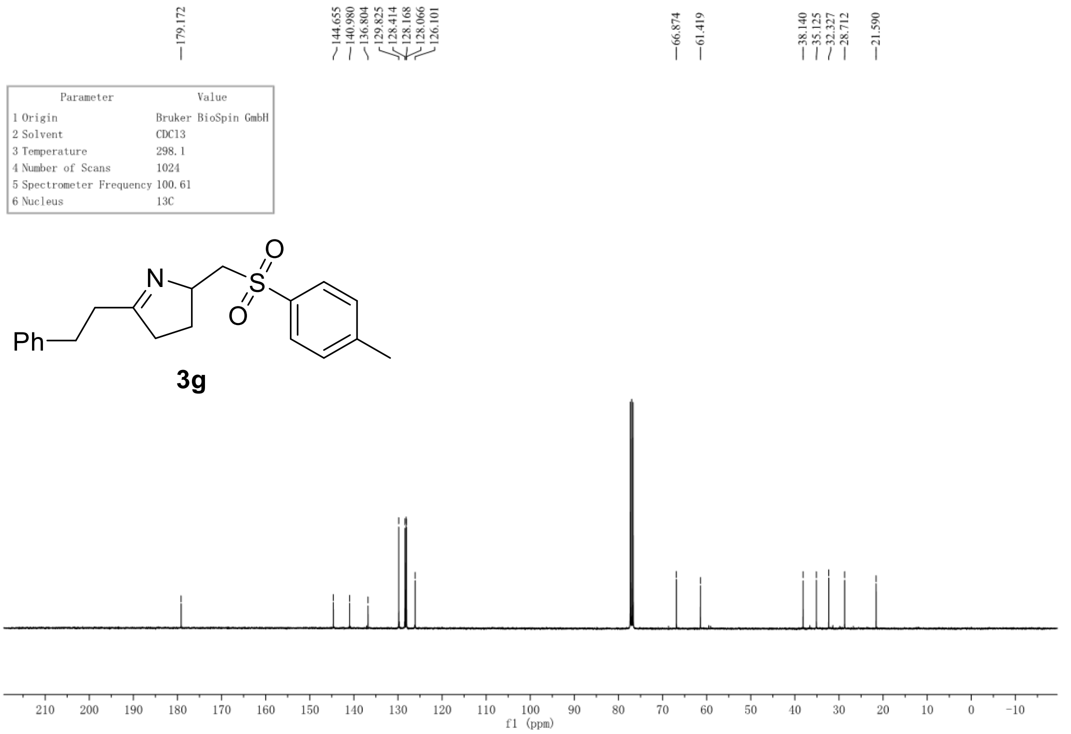
179.172 144.655 140.980 136.804 129.825 128.414 128.168 128.066 126.101 66.874 61.419 38.140 35.125 32.327 28.712 21.590
Parameter Value
1 Origin Bruker BioSpin GmbH
2 Solvent CDCl3
3 Temperature 298.1
4 Number of Scans 1024
5 Spectrometer Frequency 100.61
6 Nucleus 13C
N
S
O
O
Ph
3g
f1 (ppm)

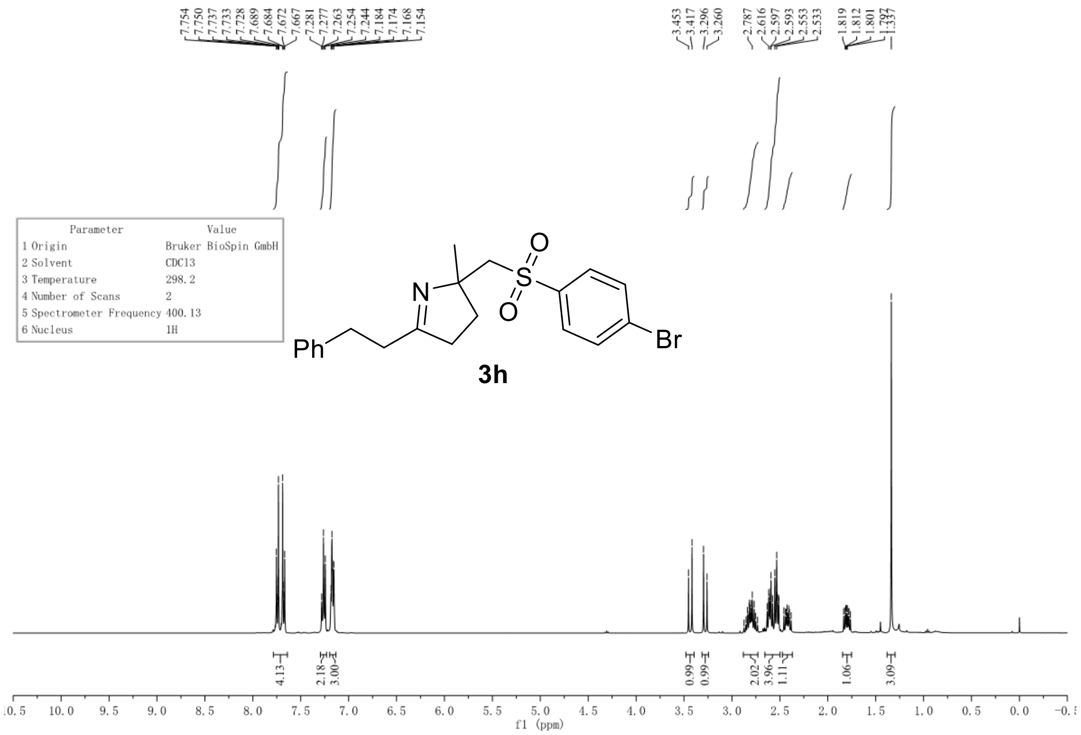
7.754 7.750 7.737 7.733 7.728 7.689 7.684 7.672 7.667 7.281 7.277 7.263 7.254 7.244 7.184 7.174 7.168 7.154
3.453 3.417 3.296 3.260
2.787 2.616 2.597 2.593 2.553 2.533
1.819 1.812 1.801 1.797 1.337
Parameter | Value
1 Origin | Bruker BioSpin GmbH
2 Solvent | CDCl3
3 Temperature | 298.2
4 Number of Scans | 2
5 Spectrometer Frequency | 400.13
6 Nucleus | 1H
N
O
S
O
Ph
Br
3h
4.13
2.18
3.00
0.99
0.99
2.02
3.96
1.11
1.06
3.09
f1 (ppm)

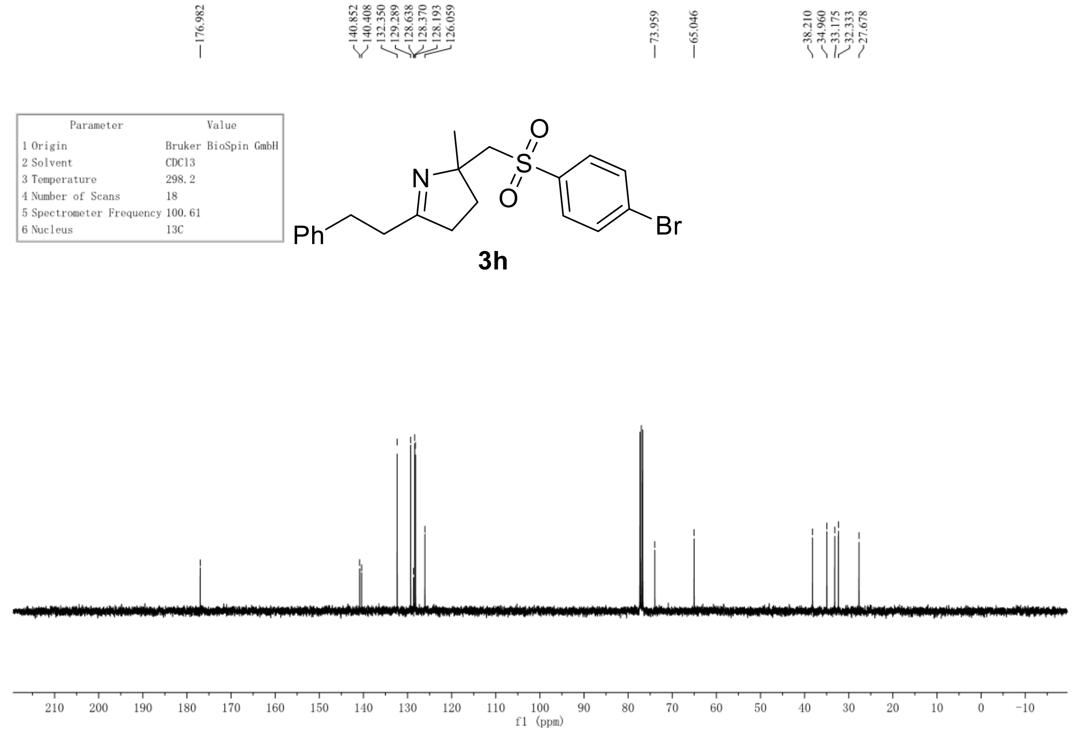
176.982
140.852 140.408 132.350 129.289 128.638 128.370 128.193 126.059
73.959
65.046
38.210 34.960 33.175 32.333 27.678
Parameter | Value
1 Origin | Bruker BioSpin GmbH
2 Solvent | CDCl3
3 Temperature | 298.2
4 Number of Scans | 18
5 Spectrometer Frequency | 100.61
6 Nucleus | 13C
N
O
S
O
Ph
Br
3h
f1 (ppm)

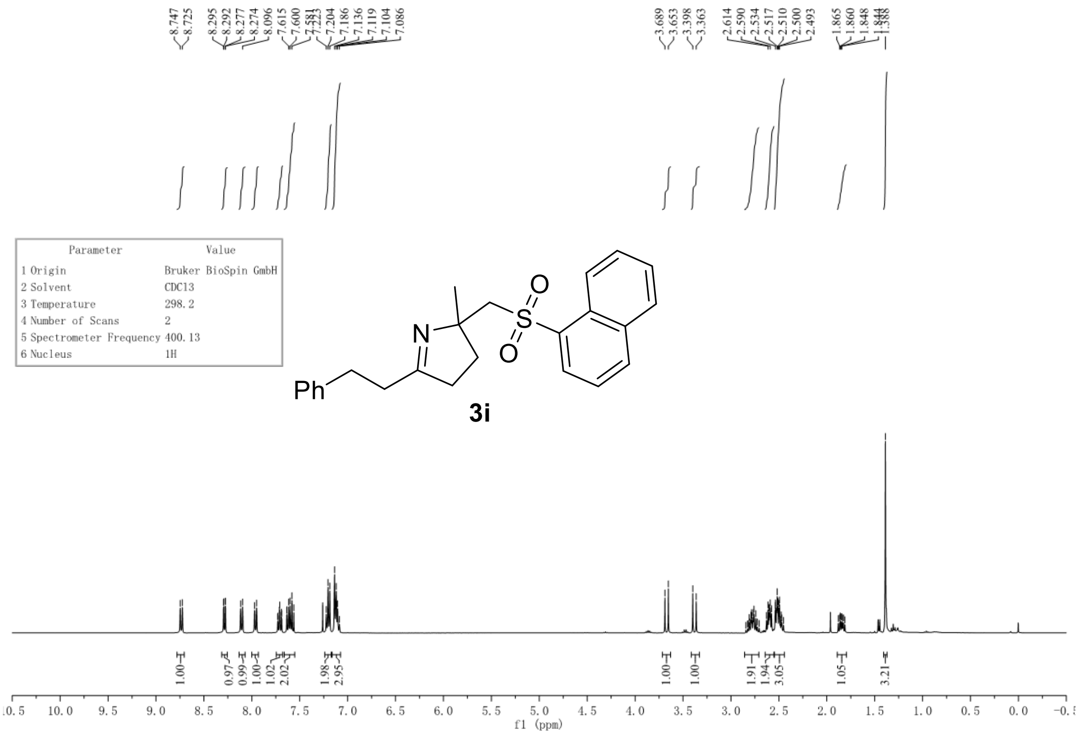
8.747
8.725
8.295
8.292
8.277
8.274
8.096
7.615
7.600
7.204
7.186
7.136
7.119
7.104
7.086
3.689
3.653
3.398
3.363
2.614
2.590
2.534
2.517
2.510
2.500
2.493
1.865
1.860
1.848
1.388
Parameter Value
1 Origin Bruker BioSpin GmbH
2 Solvent CDCl3
3 Temperature 298.2
4 Number of Scans 2
5 Spectrometer Frequency 400.13
6 Nucleus 1H
N
O
S
O
Ph
3i
1.00
0.97
0.99
1.00
1.02
2.02
1.98
2.95
1.00
1.00
1.91
1.94
3.05
1.05
3.21
f1 (ppm)

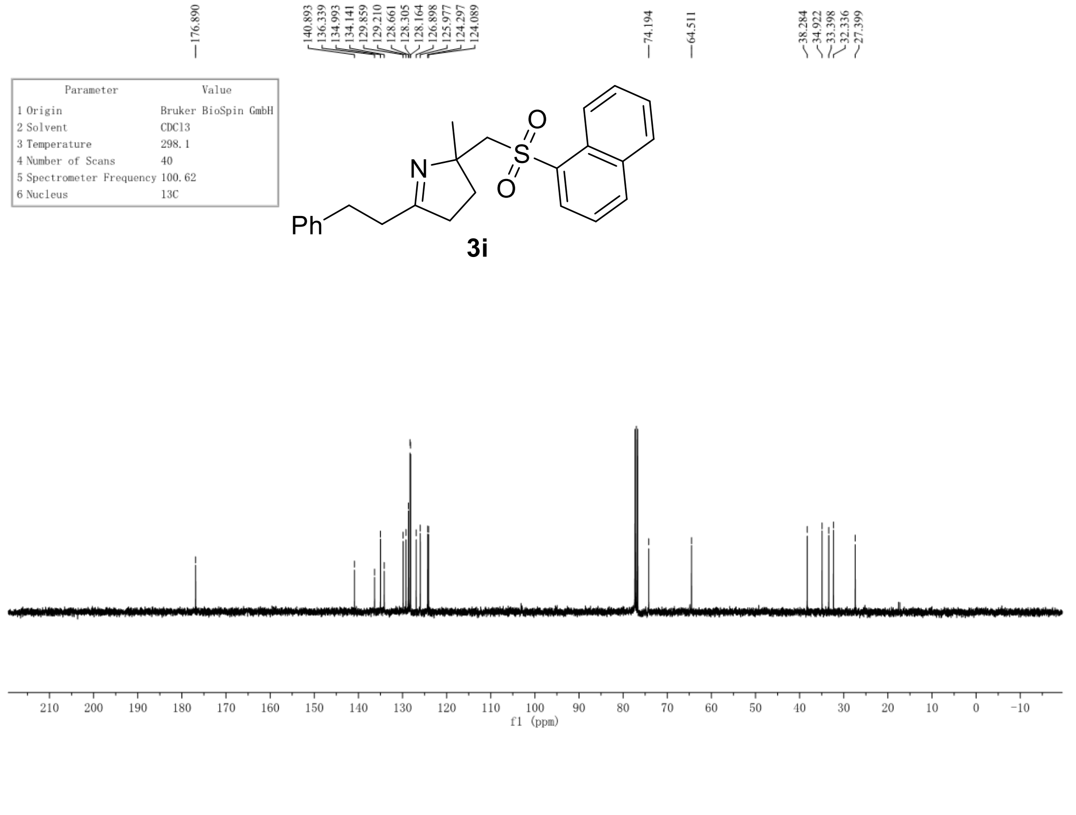
176.890
140.893
136.339
134.993
134.141
129.859
129.210
128.661
128.305
128.164
126.898
125.977
124.297
124.089
74.194
64.511
38.284
34.922
33.398
32.336
27.399
Parameter Value
1 Origin Bruker BioSpin GmbH
2 Solvent CDCl3
3 Temperature 298.1
4 Number of Scans 40
5 Spectrometer Frequency 100.62
6 Nucleus 13C
N
O
S
O
Ph
3i
f1 (ppm)

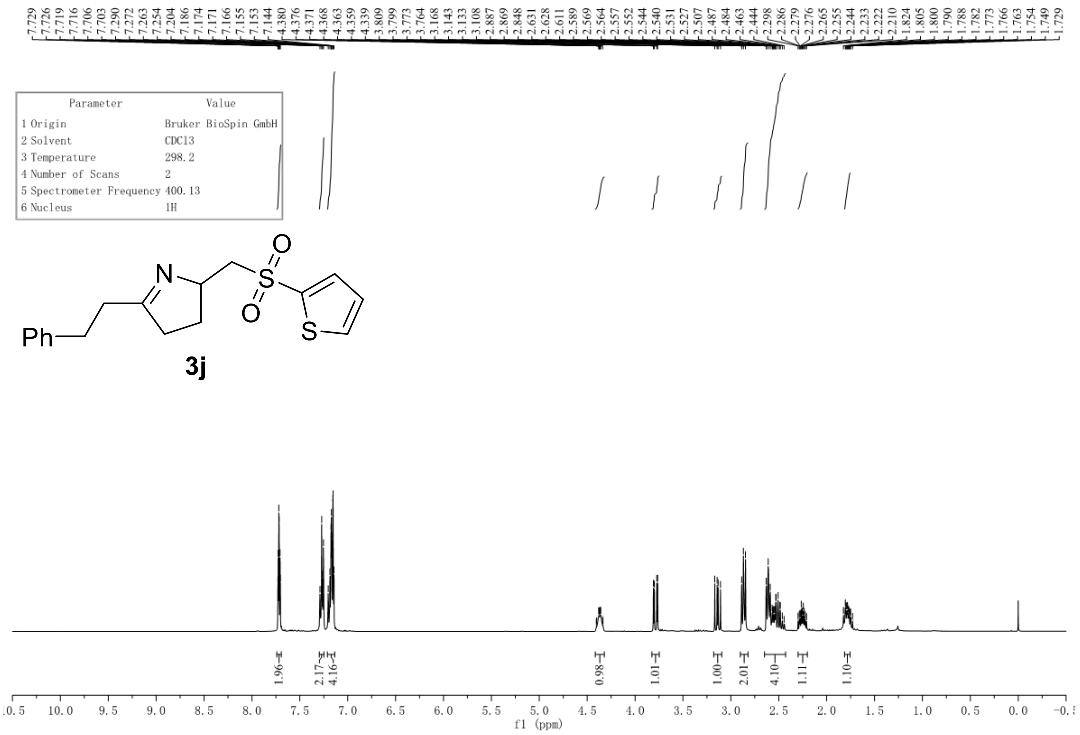
7.729 7.726 7.719 7.716 7.706 7.703 7.290 7.272 7.263 7.254 7.204 7.186 7.174 7.171 7.166 7.155 7.153 7.144 4.380 4.376 4.371 4.368 4.363 4.359 4.339 3.809 3.799 3.773 3.764 3.168 3.143 3.133 3.108 2.887 2.869 2.848 2.631 2.628 2.611 2.589 2.569 2.564 2.557 2.552 2.544 2.540 2.531 2.527 2.507 2.487 2.484 2.463 2.444 2.298 2.286 2.279 2.276 2.265 2.255 2.244 2.233 2.222 2.210 1.824 1.805 1.800 1.790 1.788 1.782 1.773 1.766 1.763 1.754 1.749 1.729
Parameter Value
1 Origin Bruker BioSpin GmbH
2 Solvent CDCl3
3 Temperature 298.2
4 Number of Scans 2
5 Spectrometer Frequency 400.13
6 Nucleus 1H
Ph
N
S
O
O
S
3j
1.96 2.17 4.16 0.98 1.01 1.00 2.01 4.10 1.11 1.10
10.5 10.0 9.5 9.0 8.5 8.0 7.5 7.0 6.5 6.0 5.5 5.0 4.5 4.0 3.5 3.0 2.5 2.0 1.5 1.0 0.5 0.0 -0.5
f1 (ppm)

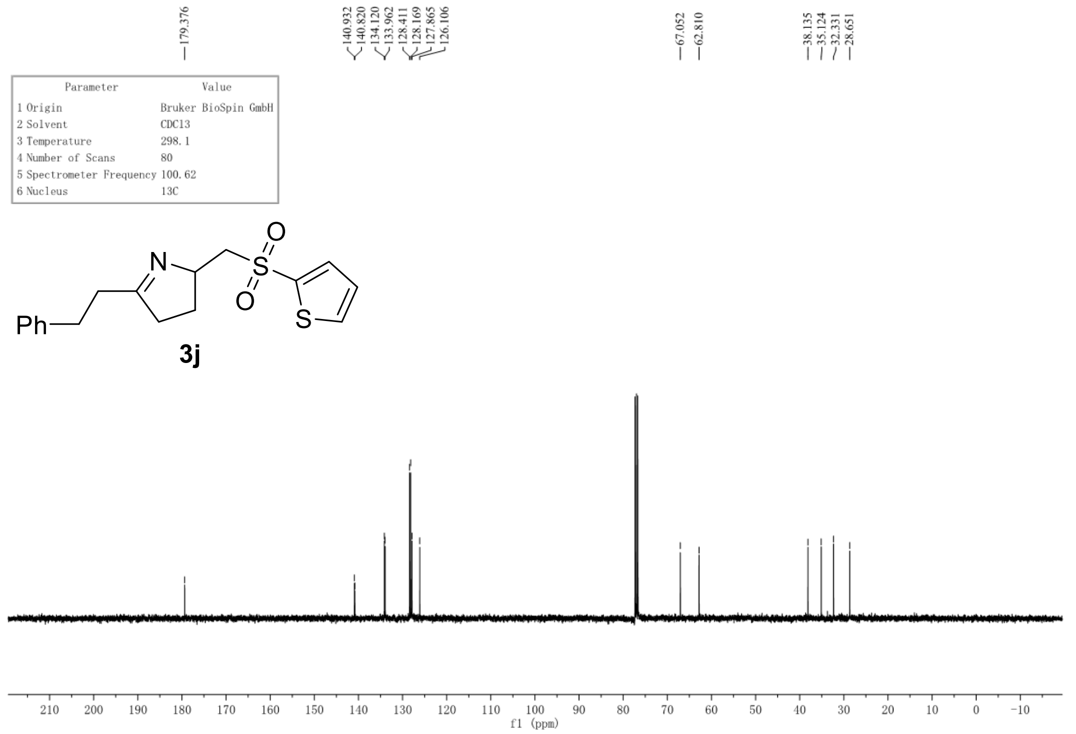
179.376
140.932 140.820 134.120 133.962 128.411 128.169 127.865 126.106
67.052 62.810
38.135 35.124 32.331 28.651
Parameter Value
1 Origin Bruker BioSpin GmbH
2 Solvent CDCl3
3 Temperature 298.1
4 Number of Scans 80
5 Spectrometer Frequency 100.62
6 Nucleus 13C
Ph
N
S
O
O
S
3j
210 200 190 180 170 160 150 140 130 120 110 100 90 80 70 60 50 40 30 20 10 0 -10
f1 (ppm)

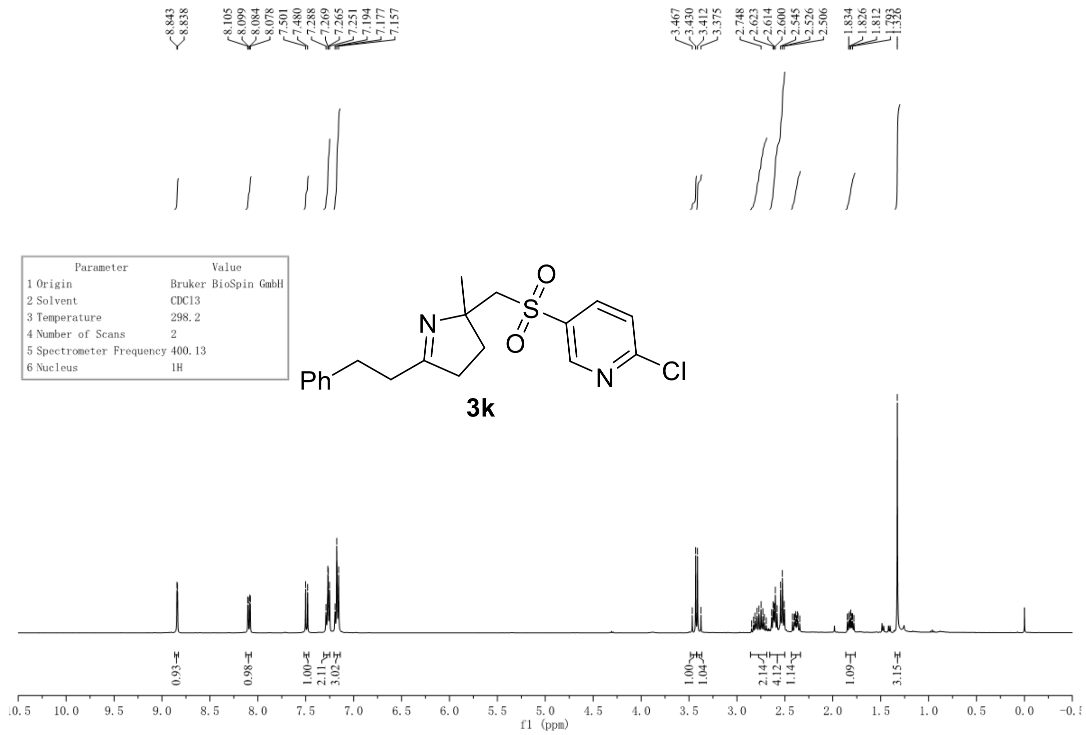
Parameter Value
1 Origin Bruker BioSpin GmbH
2 Solvent CDCl3
3 Temperature 298.2
4 Number of Scans 2
5 Spectrometer Frequency 400.13
6 Nucleus 1H
Ph
N
S
O
O
N
Cl
3k
f1 (ppm)

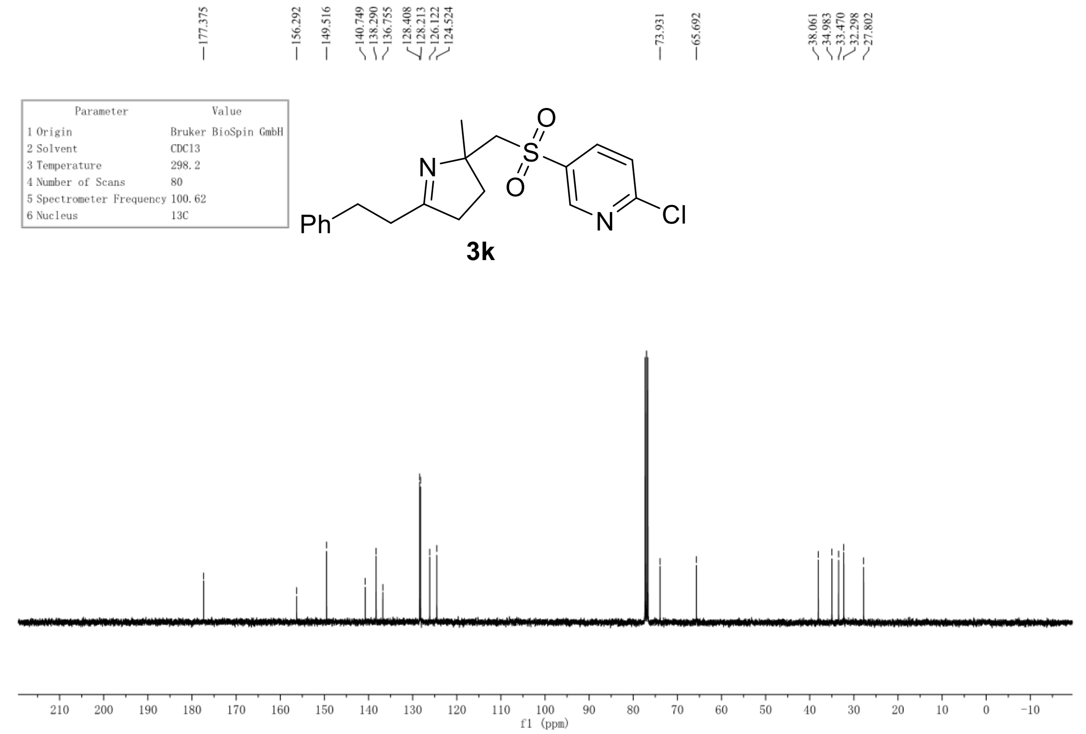
Parameter Value
1 Origin Bruker BioSpin GmbH
2 Solvent CDCl3
3 Temperature 298.2
4 Number of Scans 80
5 Spectrometer Frequency 100.62
6 Nucleus 13C
177.375
156.292
149.516
140.749
138.290
136.755
128.408
128.213
126.122
124.524
73.931
65.692
38.061
34.983
33.470
32.298
27.802
Ph
N
S
O
O
N
Cl
3k
f1 (ppm)

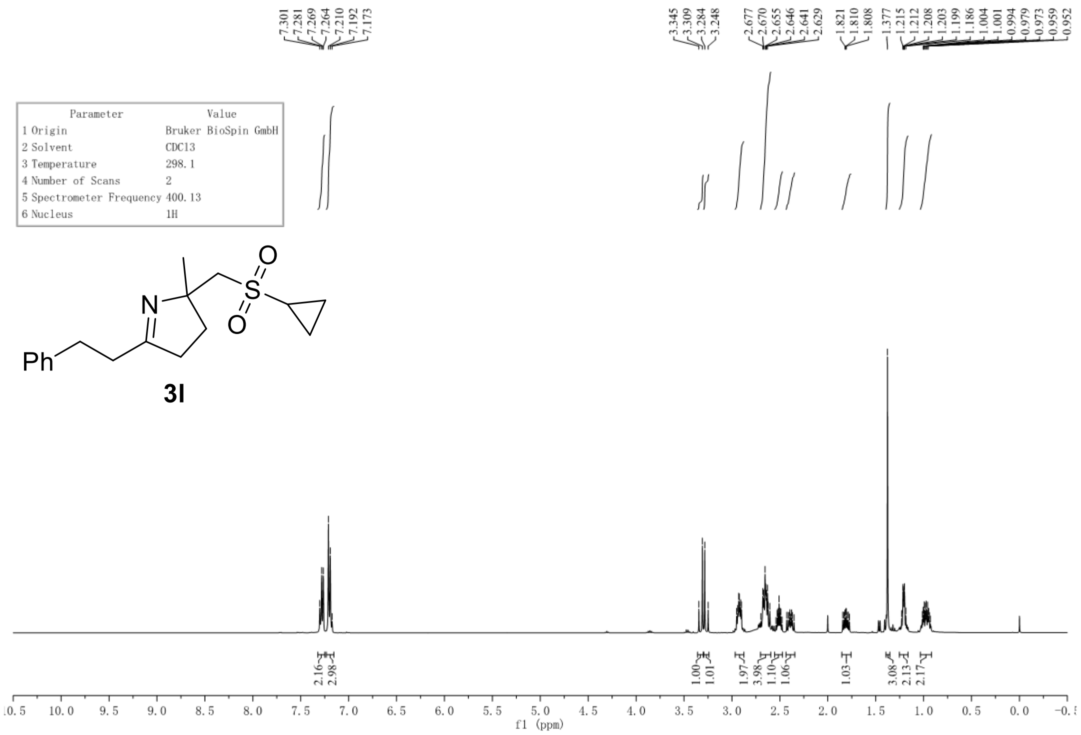

Parameter	Value
1 Origin	Bruker BioSpin GmbH
2 Solvent	CDCl3
3 Temperature	298.1
4 Number of Scans	2
5 Spectrometer Frequency	400.13
6 Nucleus	1H

3l

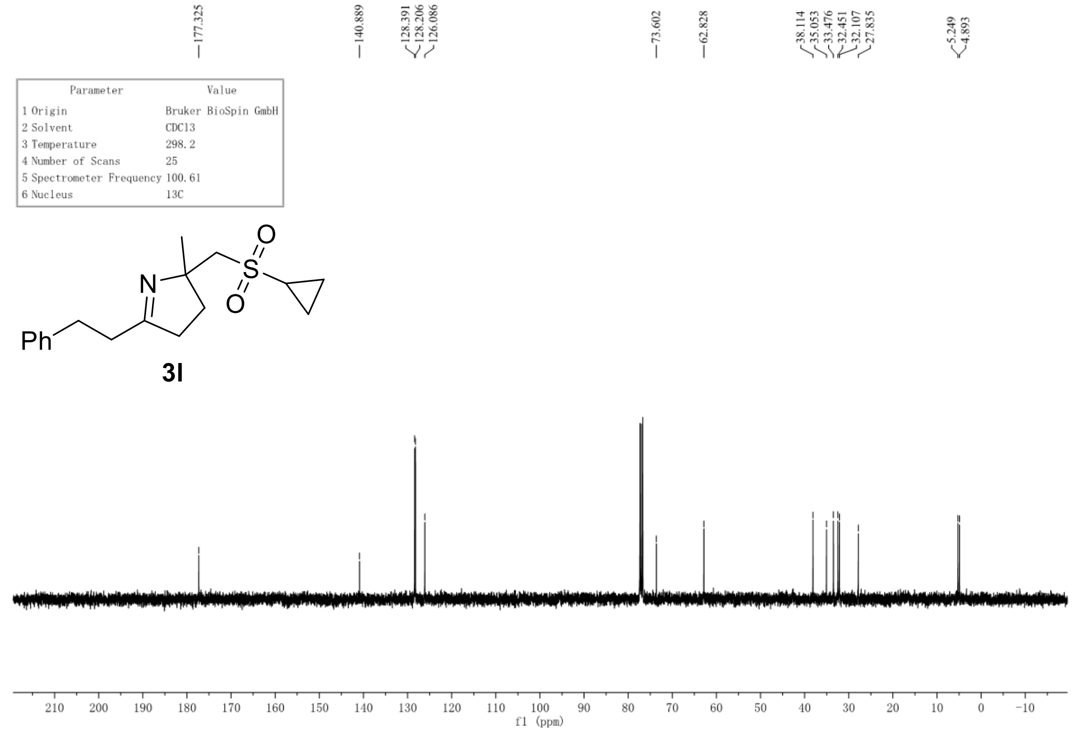

Parameter	Value
1 Origin	Bruker BioSpin GmbH
2 Solvent	CDCl3
3 Temperature	298.2
4 Number of Scans	25
5 Spectrometer Frequency	100.61
6 Nucleus	13C

3l

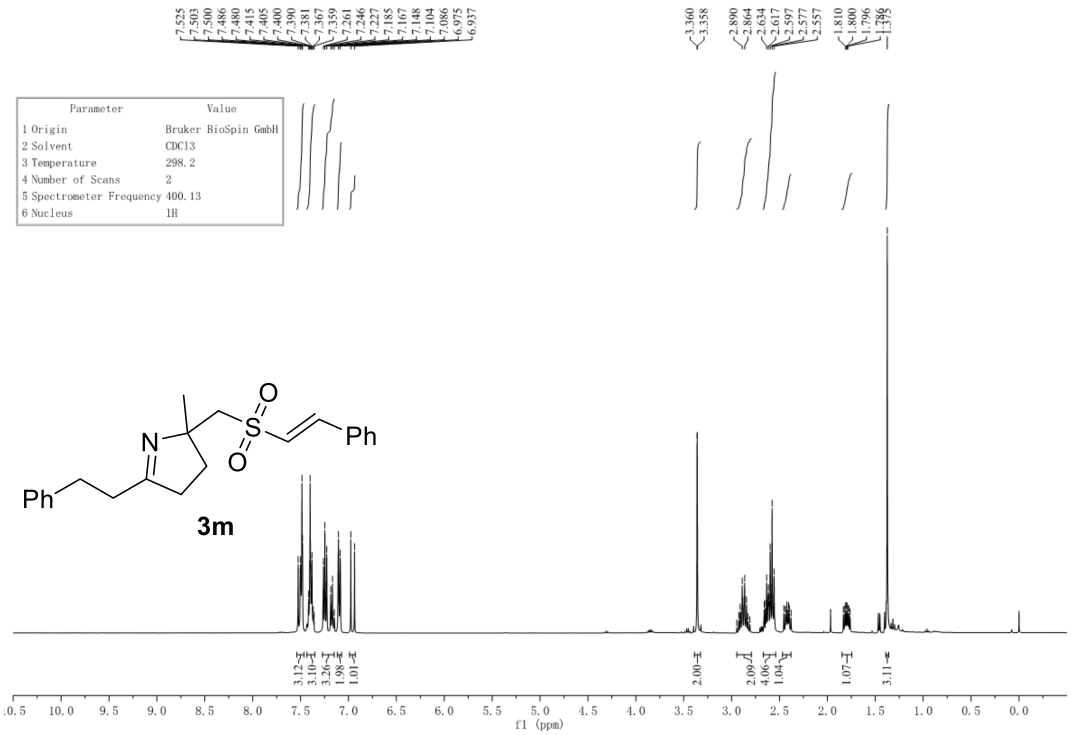
7.525 7.503 7.500 7.486 7.480 7.415 7.405 7.400 7.390 7.381 7.367 7.359 7.261 7.246 7.227 7.185 7.167 7.148 7.104 7.086 6.975 6.937
3.360 3.358 2.890 2.864 2.634 2.617 2.597 2.577 2.557 1.810 1.800 1.796 1.786 1.375
Parameter Value
1 Origin Bruker BioSpin GmbH
2 Solvent CDCl3
3 Temperature 298.2
4 Number of Scans 2
5 Spectrometer Frequency 400.13
6 Nucleus 1H
N
S
O
O
Ph
Ph
3m
3.12 3.10 3.26 1.98 1.01 2.00 2.09 4.06 1.04 1.07 3.11
f1 (ppm)

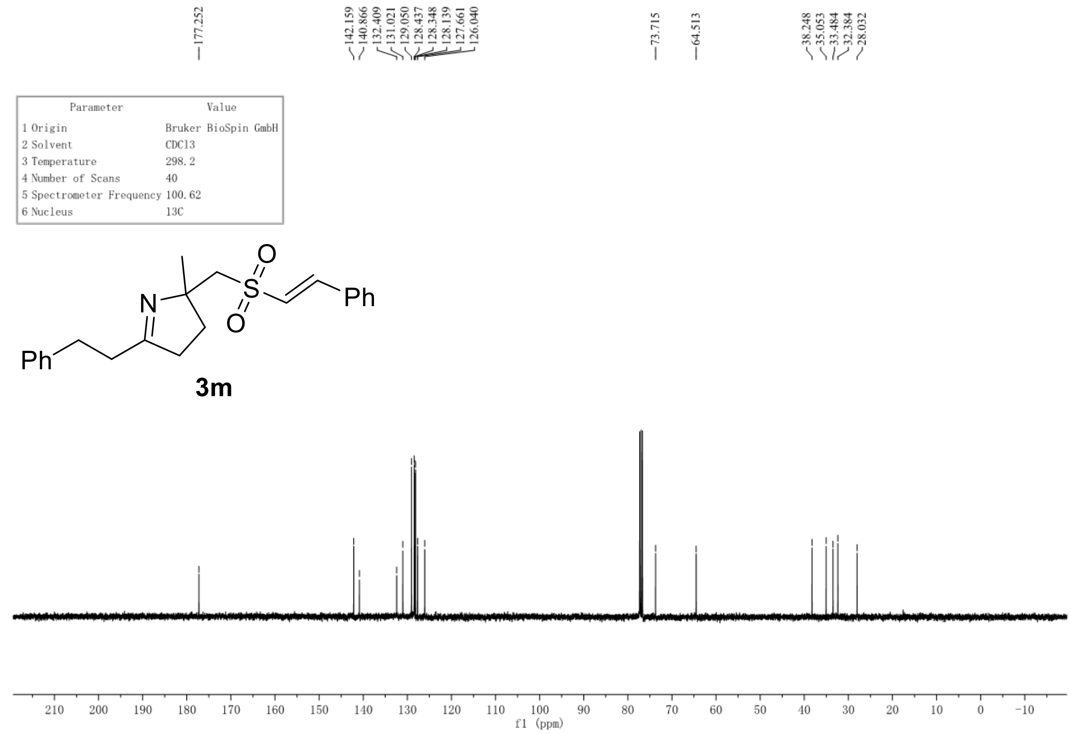
177.252
142.159 140.866 132.409 131.021 129.050 128.437 128.348 128.139 127.661 126.040
73.715
64.513
38.248 35.053 33.484 32.384 28.032
Parameter Value
1 Origin Bruker BioSpin GmbH
2 Solvent CDCl3
3 Temperature 298.2
4 Number of Scans 40
5 Spectrometer Frequency 100.62
6 Nucleus 13C
N
S
O
O
Ph
Ph
3m
f1 (ppm)

Parameter	Value
1 Origin	Bruker BioSpin GmbH
2 Solvent	CDCl3
3 Temperature	298.2
4 Number of Scans	2
5 Spectrometer Frequency	400.13
6 Nucleus	1H

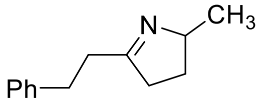

3n

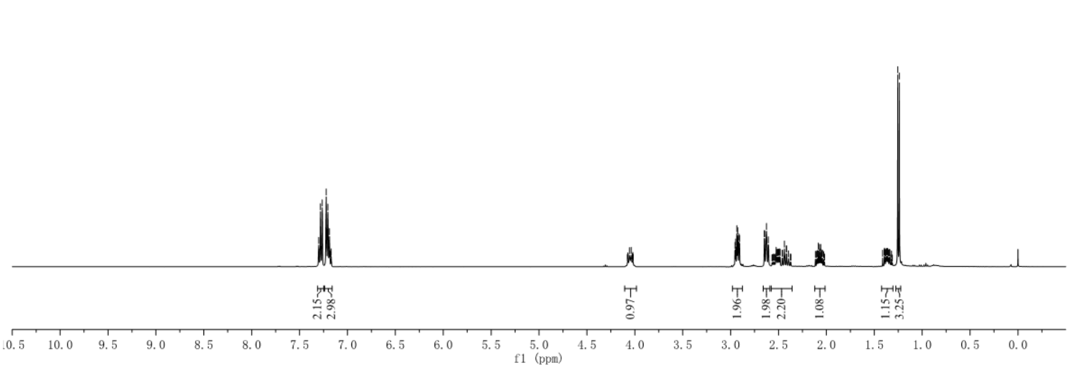

Parameter	Value
1 Origin	Bruker BioSpin GmbH
2 Solvent	CDCl3
3 Temperature	298.1
4 Number of Scans	80
5 Spectrometer Frequency	100.62
6 Nucleus	13C

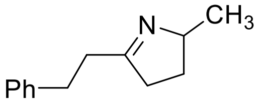

3n

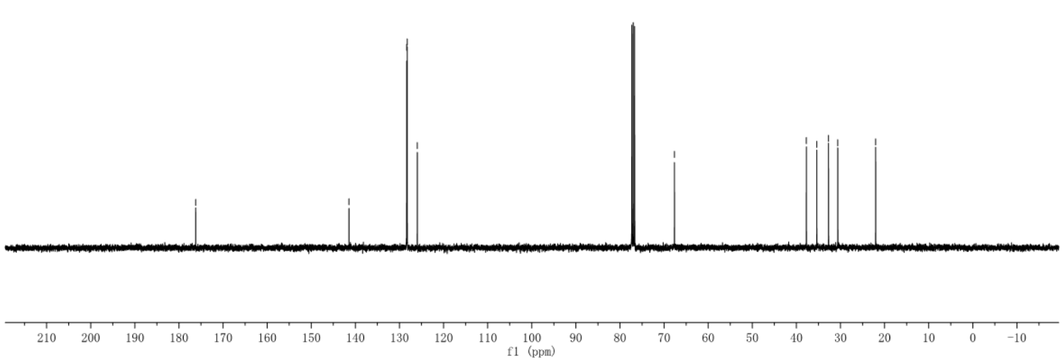

Parameter	Value
1 Origin	Bruker BioSpin GmbH
2 Solvent	CDCl3
3 Temperature	298.2
4 Number of Scans	2
5 Spectrometer Frequency	400.13
6 Nucleus	1H

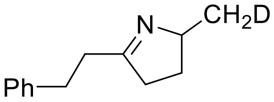

3o

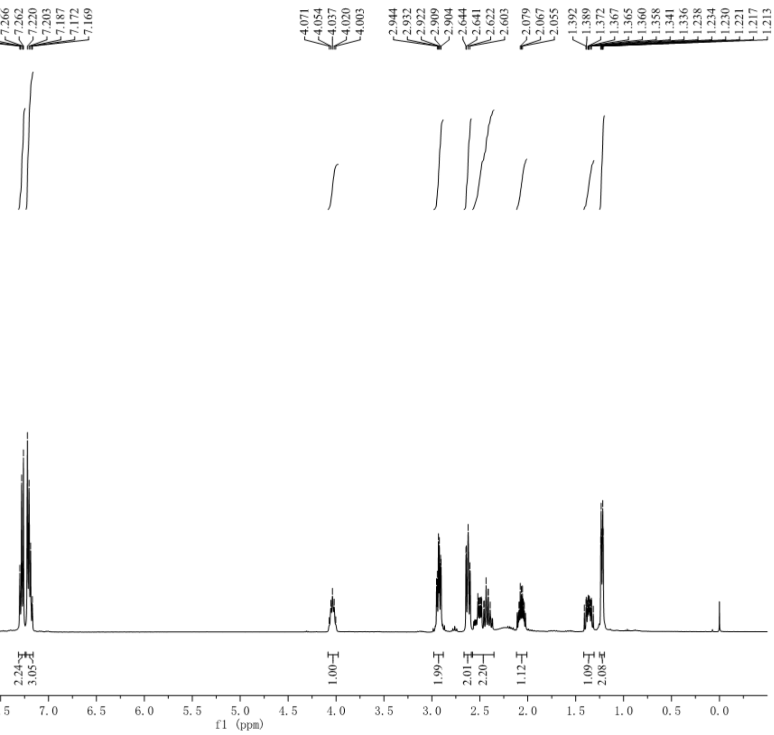

Parameter	Value
1 Origin	Bruker BioSpin GmbH
2 Solvent	CDCl3
3 Temperature	298.1
4 Number of Scans	80
5 Spectrometer Frequency	100.62
6 Nucleus	13C

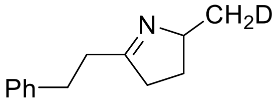

3o

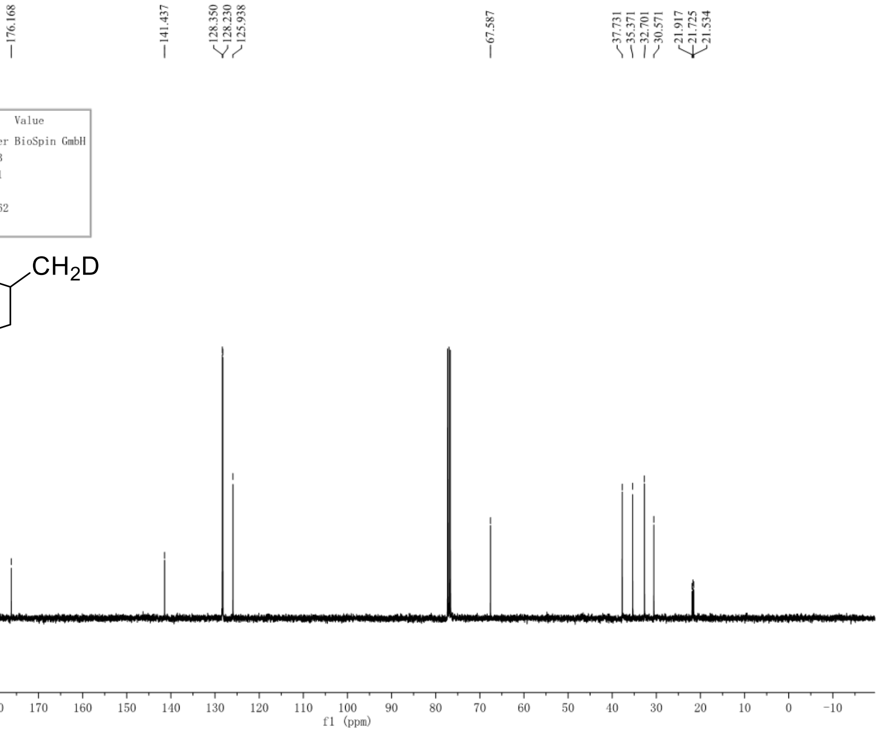

Parameter	Value
1 Origin	Bruker BioSpin GmbH
2 Solvent	CDCl3
3 Temperature	298.2
4 Number of Scans	2
5 Spectrometer Frequency	400.13
6 Nucleus	1H

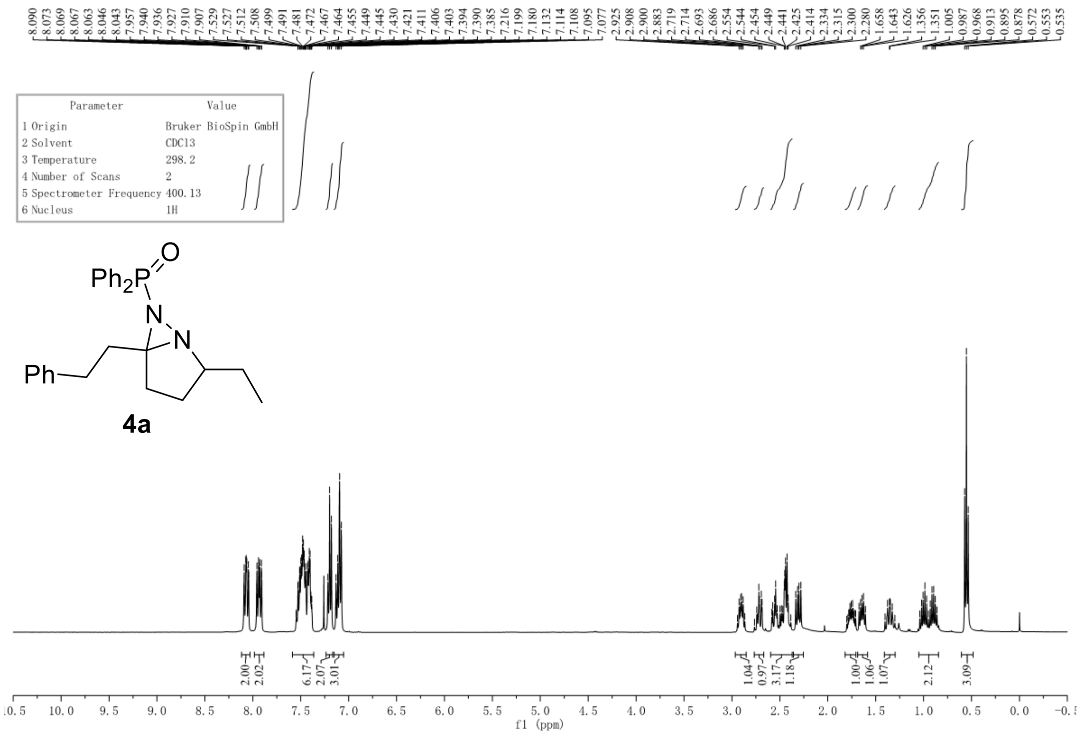

Parameter	Value
1 Origin	Bruker BioSpin GmbH
2 Solvent	CDCl3
3 Temperature	298.1
4 Number of Scans	1024
5 Spectrometer Frequency	100.62
6 Nucleus	13C

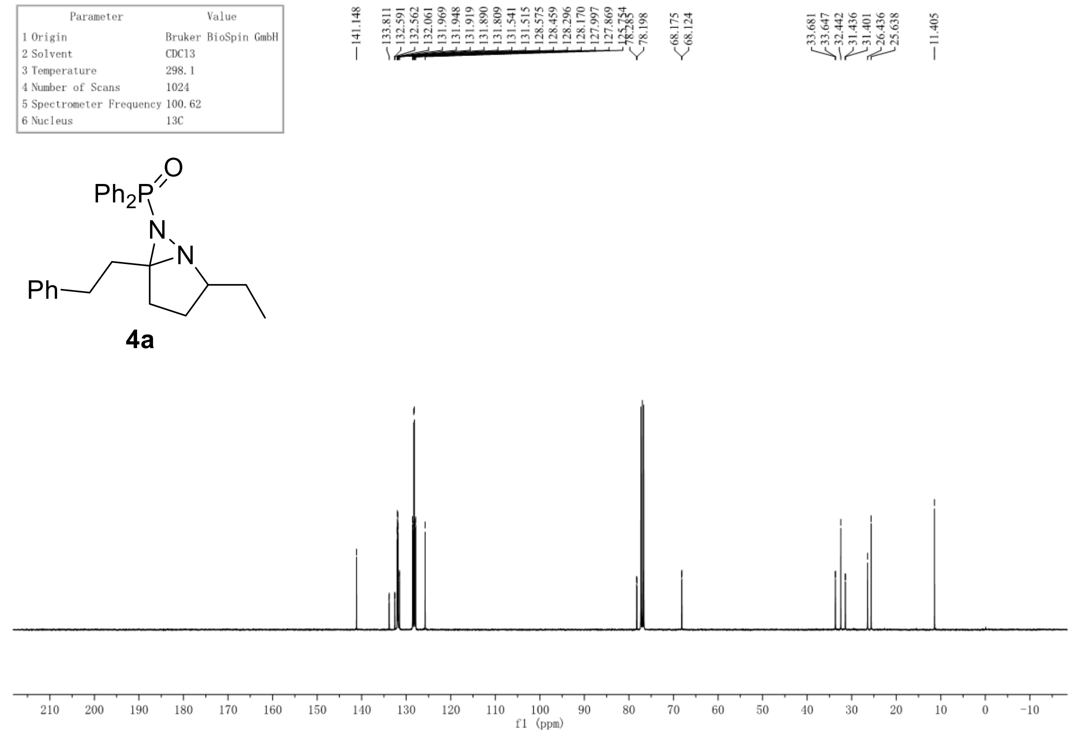

Parameter	Value
1 Origin	Bruker BioSpin GmbH
2 Solvent	CDCl3
3 Temperature	298.3
4 Number of Scans	2
5 Spectrometer Frequency	161.97
6 Nucleus	31P

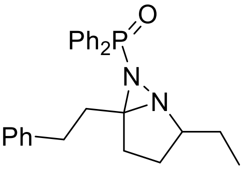

4a

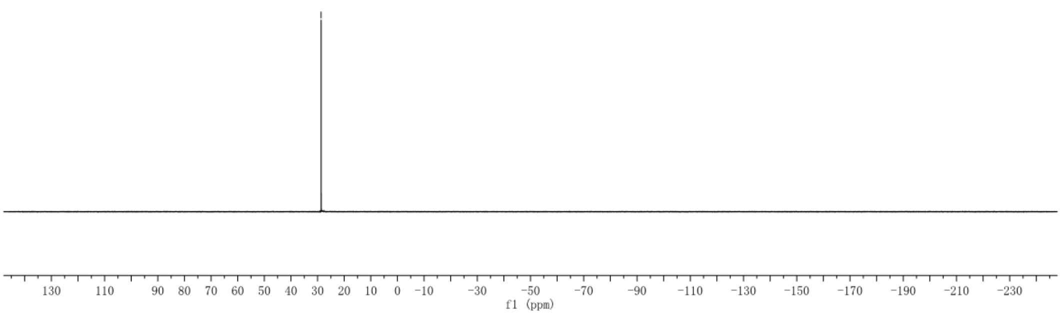

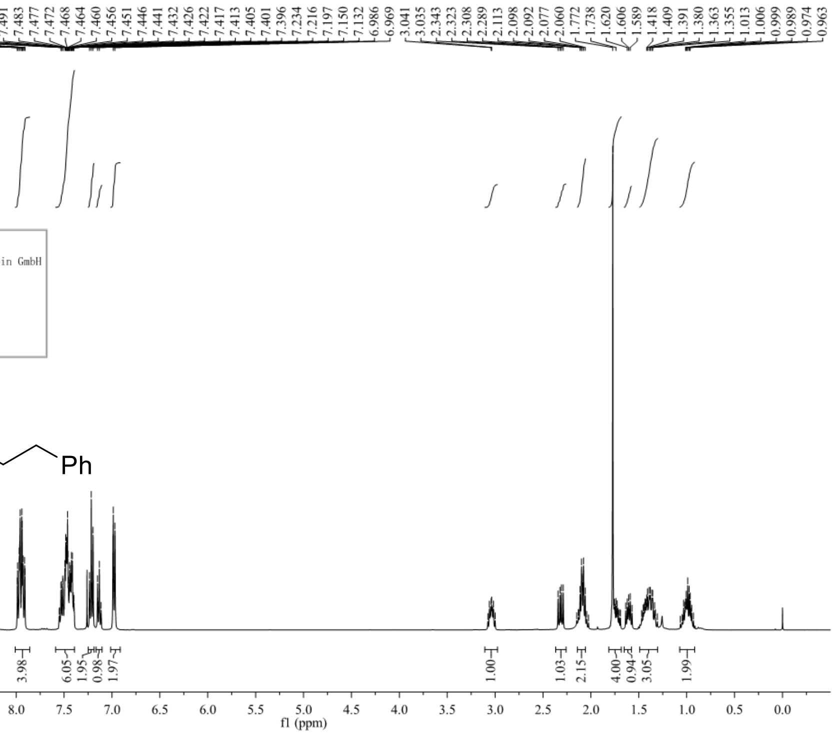

Parameter	Value
1 Origin	Bruker BioSpin GmbH
2 Solvent	CDCl3
3 Temperature	296.8
4 Number of Scans	2
5 Spectrometer Frequency	400.15
6 Nucleus	1H

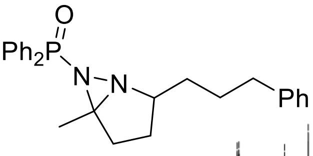

4b

Parameter	Value
1 Origin	Bruker BioSpin GmbH
2 Solvent	CDCl3
3 Temperature	296.9
4 Number of Scans	73
5 Spectrometer Frequency	100.62
6 Nucleus	13C

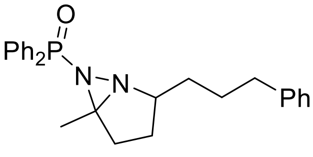

4b

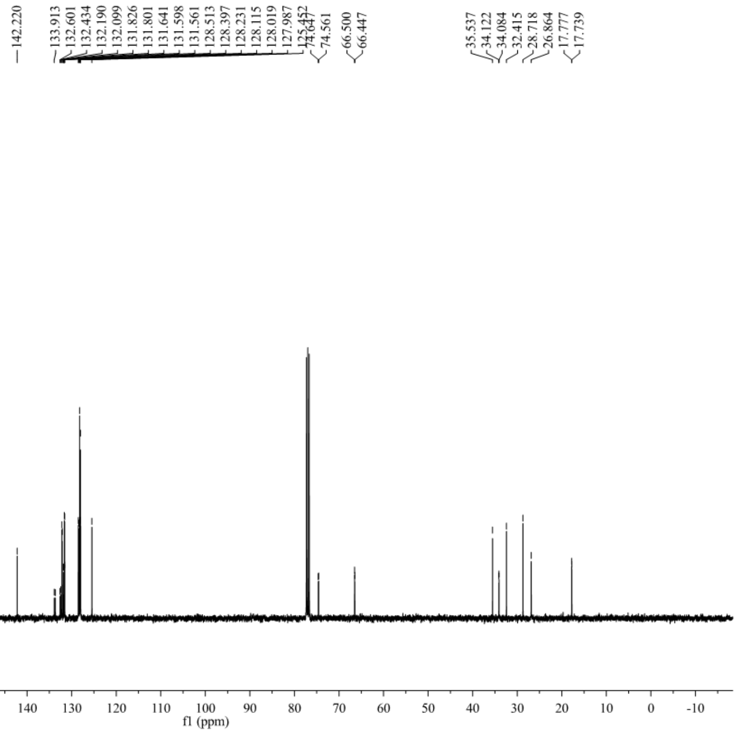

Parameter	Value
1 Origin	Bruker BioSpin GmbH
2 Solvent	CDCl3
3 Temperature	296.9
4 Number of Scans	2
5 Spectrometer Frequency	161.98
6 Nucleus	31P

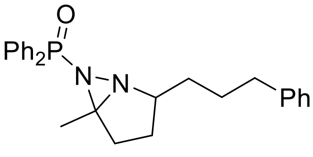

4b

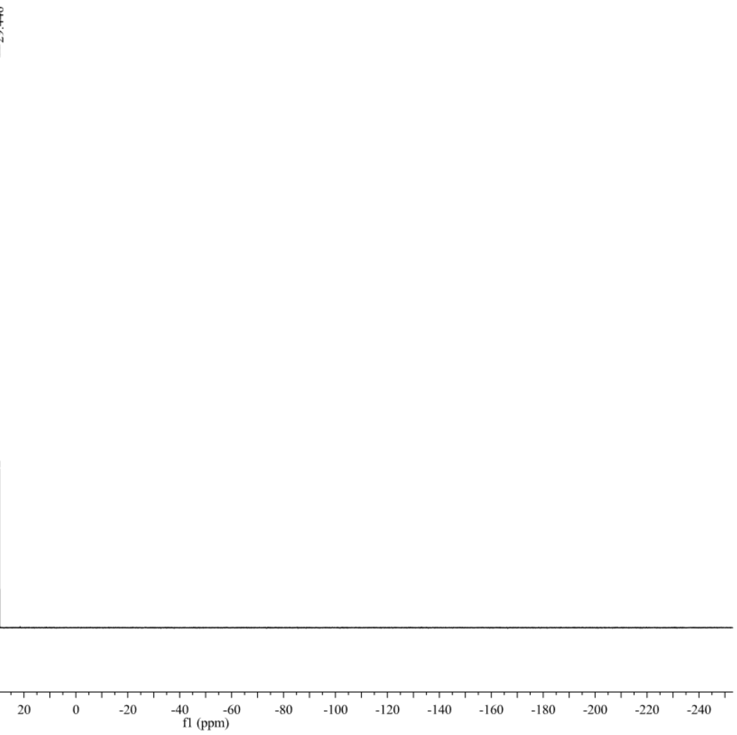

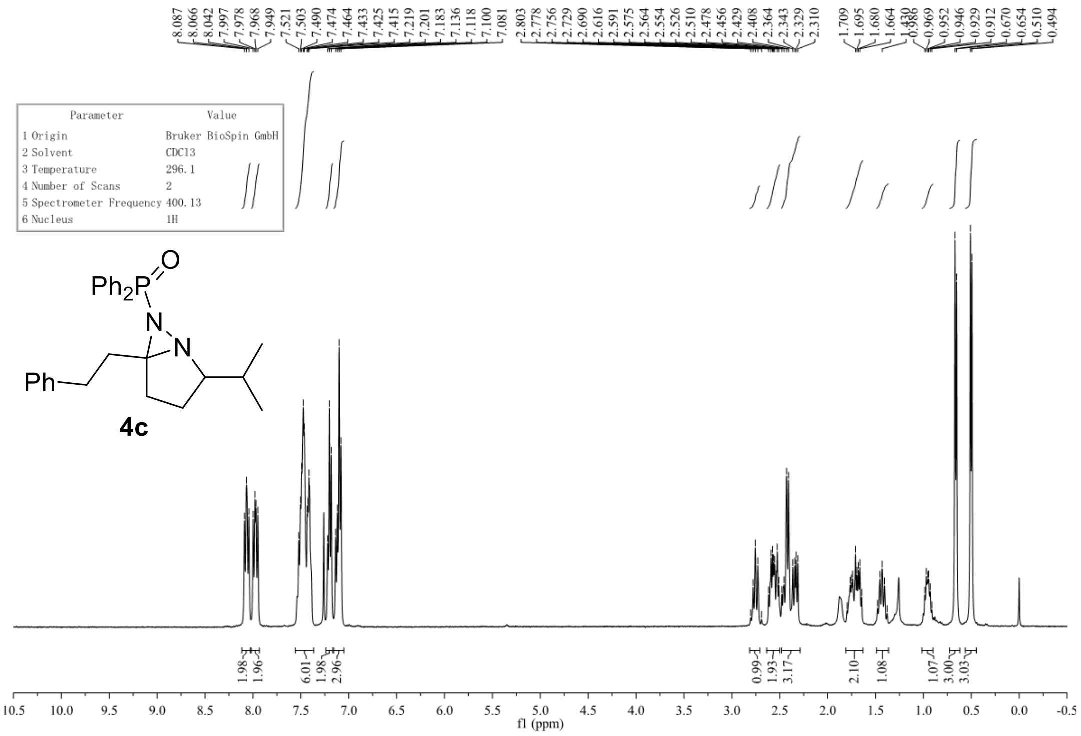

Parameter Value
1 Origin Bruker BioSpin GmbH
2 Solvent CDCl3
3 Temperature 296.1
4 Number of Scans 2
5 Spectrometer Frequency 400.13
6 Nucleus 1H
Ph2P O
N
N
Ph
4c
f1 (ppm)

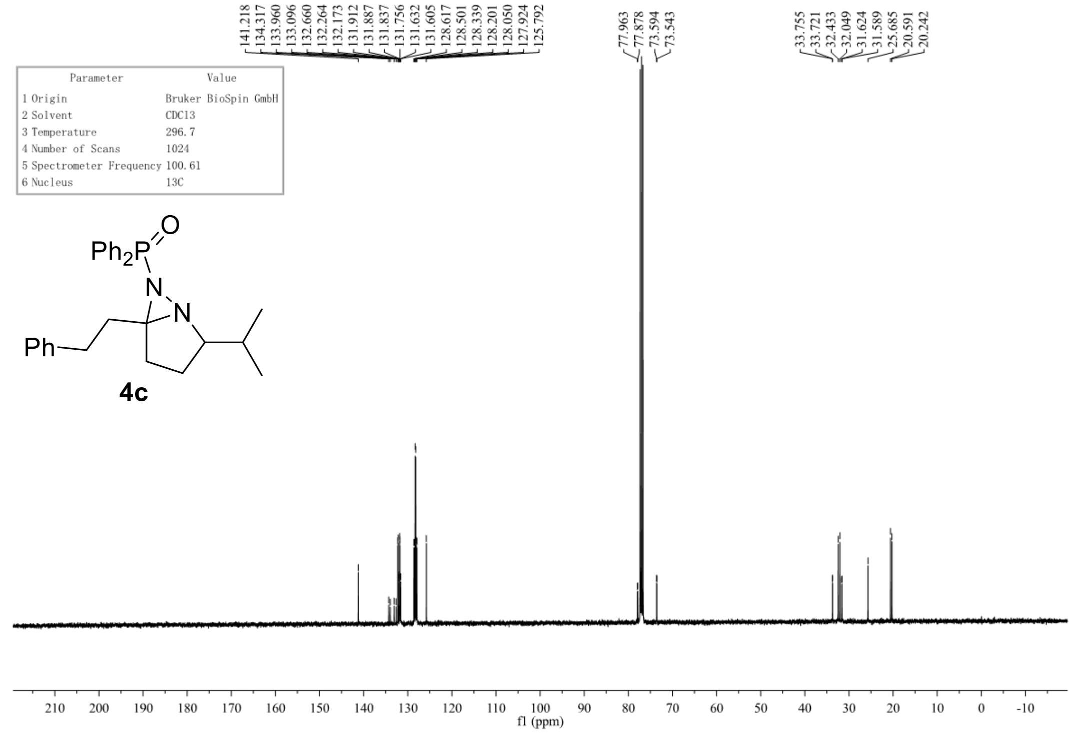

Parameter Value
1 Origin Bruker BioSpin GmbH
2 Solvent CDCl3
3 Temperature 296.7
4 Number of Scans 1024
5 Spectrometer Frequency 100.61
6 Nucleus 13C
Ph2P O
N
N
Ph
4c
f1 (ppm)

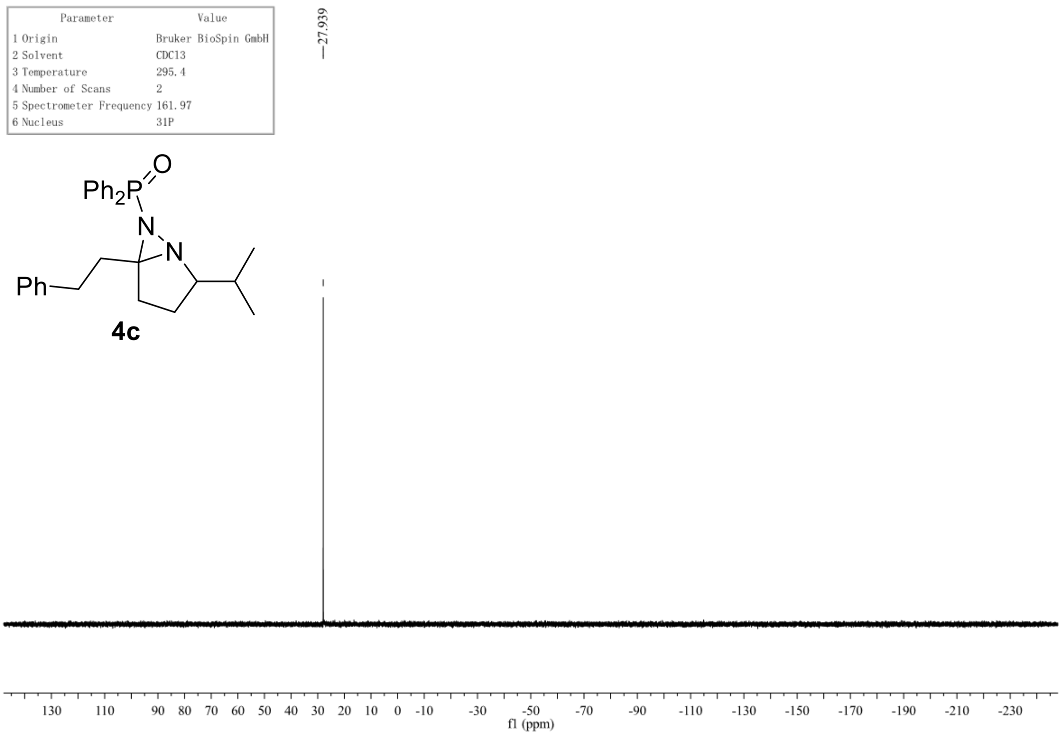

Parameter	Value
1 Origin	Bruker BioSpin GmbH
2 Solvent	CDCl3
3 Temperature	295.4
4 Number of Scans	2
5 Spectrometer Frequency	161.97
6 Nucleus	31P

4c

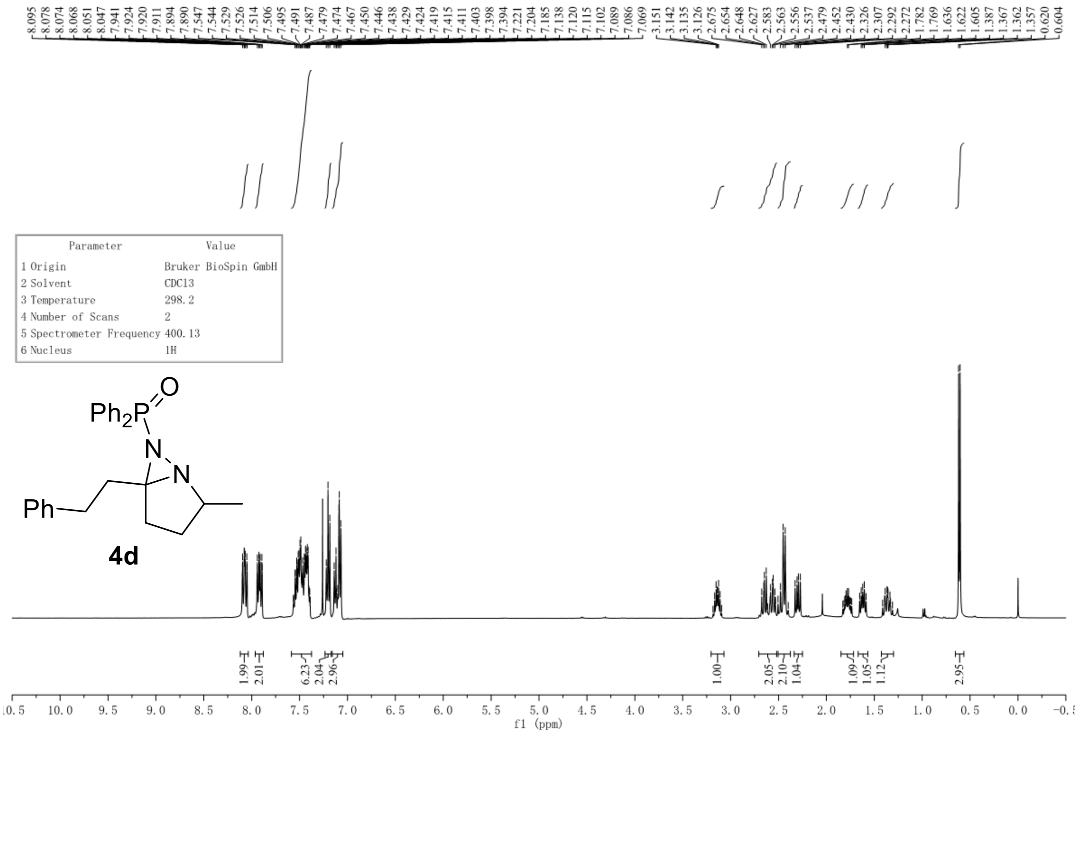

Parameter	Value
1 Origin	Bruker BioSpin GmbH
2 Solvent	CDCl3
3 Temperature	298.2
4 Number of Scans	2
5 Spectrometer Frequency	400.13
6 Nucleus	1H

4d

Parameter	Value
1 Origin	Bruker BioSpin GmbH
2 Solvent	CDCl3
3 Temperature	298.1
4 Number of Scans	1024
5 Spectrometer Frequency	100.61
6 Nucleus	13C

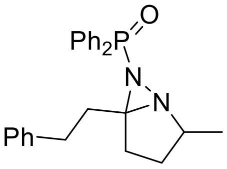

4d

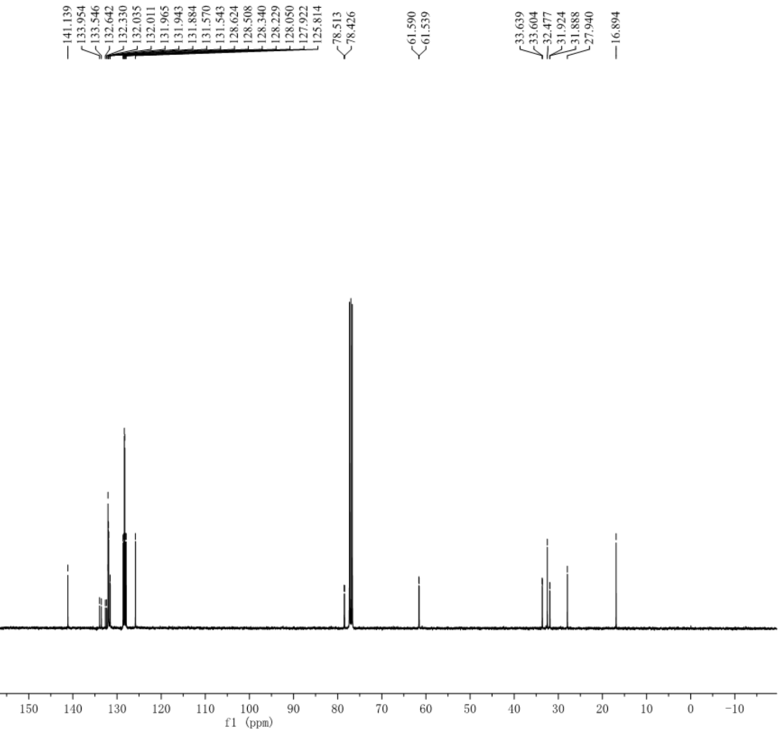

Parameter	Value
1 Origin	Bruker BioSpin GmbH
2 Solvent	CDCl3
3 Temperature	298.2
4 Number of Scans	2
5 Spectrometer Frequency	161.98
6 Nucleus	31P

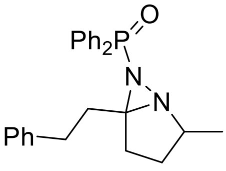

4d

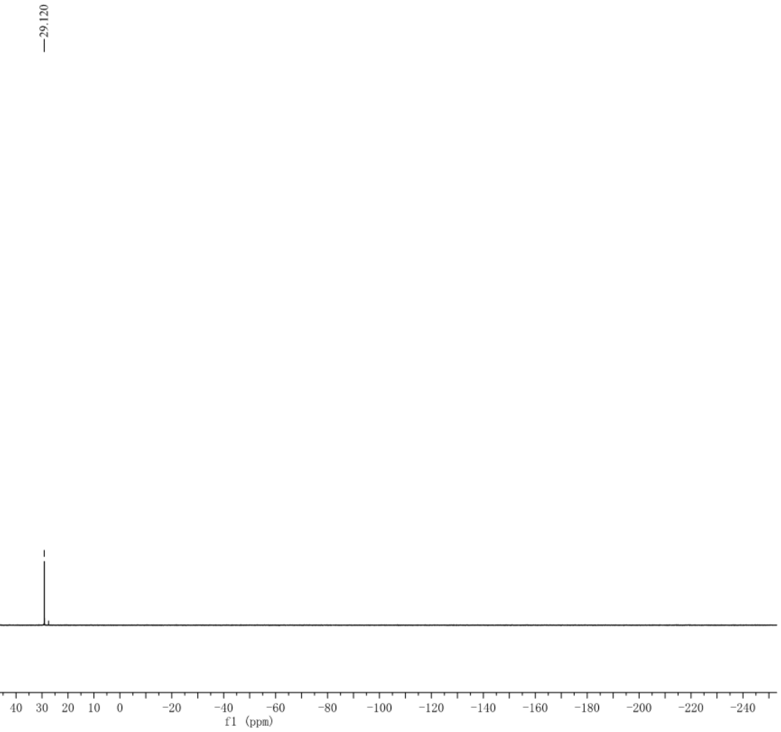

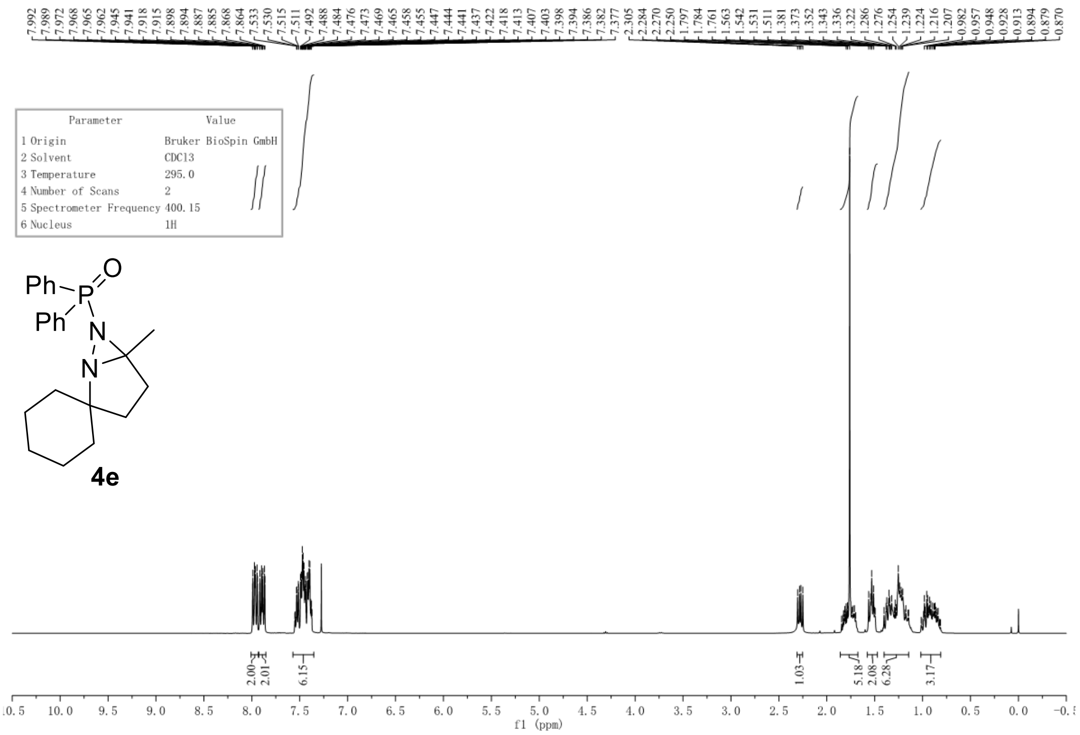
7.992 7.989 7.972 7.968 7.965 7.962 7.945 7.941 7.918 7.915 7.898 7.894 7.887 7.885 7.868 7.864 7.533 7.530 7.515 7.511 7.492 7.488 7.484 7.476 7.473 7.469 7.465 7.458 7.455 7.447 7.444 7.441 7.437 7.422 7.418 7.413 7.407 7.403 7.398 7.394 7.386 7.382 7.377 2.305 2.284 2.270 2.250 1.797 1.784 1.761 1.563 1.542 1.531 1.511 1.381 1.373 1.352 1.343 1.336 1.322 1.286 1.276 1.254 1.239 1.224 1.216 1.207 0.982 0.957 0.948 0.928 0.913 0.894 0.879 0.870
Parameter Value
1 Origin Bruker BioSpin GmbH
2 Solvent CDCl3
3 Temperature 295.0
4 Number of Scans 2
5 Spectrometer Frequency 400.15
6 Nucleus 1H
Ph P O Ph N N
4e
2.00 2.01 6.15 1.03 5.18 2.08 6.28 3.17
10.5 10.0 9.5 9.0 8.5 8.0 7.5 7.0 6.5 6.0 5.5 5.0 4.5 4.0 3.5 3.0 2.5 2.0 1.5 1.0 0.5 0.0 -0.5
f1 (ppm)

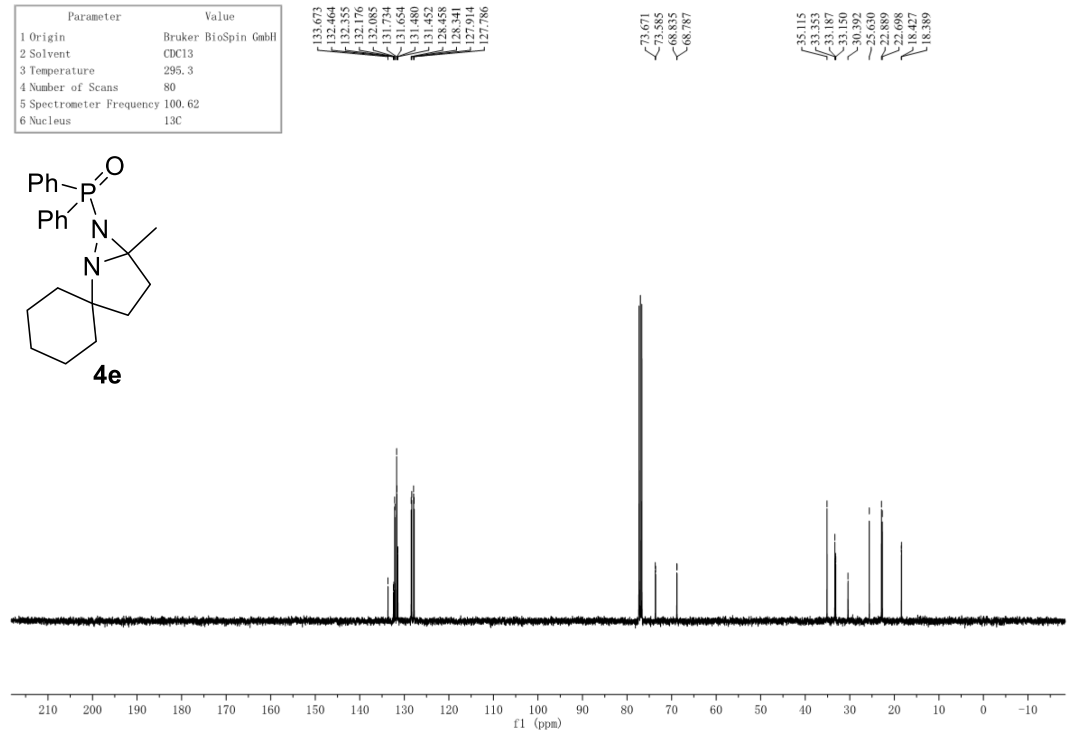
Parameter Value
1 Origin Bruker BioSpin GmbH
2 Solvent CDCl3
3 Temperature 295.3
4 Number of Scans 80
5 Spectrometer Frequency 100.62
6 Nucleus 13C
133.673 132.464 132.355 132.176 132.085 131.734 131.654 131.480 131.452 128.458 128.341 127.914 127.786
73.671 73.585 68.835 68.787
35.115 33.353 33.187 33.150 30.392 25.630 22.889 22.698 18.427 18.389
Ph P O Ph N N
4e
210 200 190 180 170 160 150 140 130 120 110 100 90 80 70 60 50 40 30 20 10 0 -10
f1 (ppm)

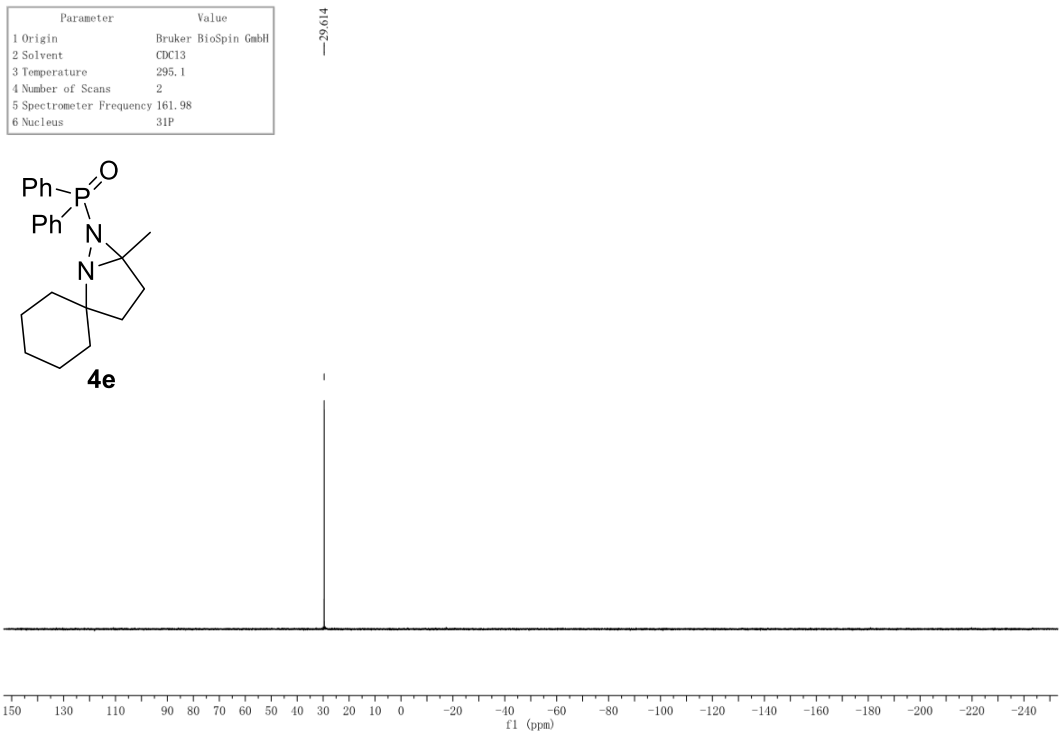
Parameter
Value
1 Origin
Bruker BioSpin GmbH
2 Solvent
CDCl3
3 Temperature
295.1
4 Number of Scans
2
5 Spectrometer Frequency
161.98
6 Nucleus
31P
29.614
4e
f1 (ppm)

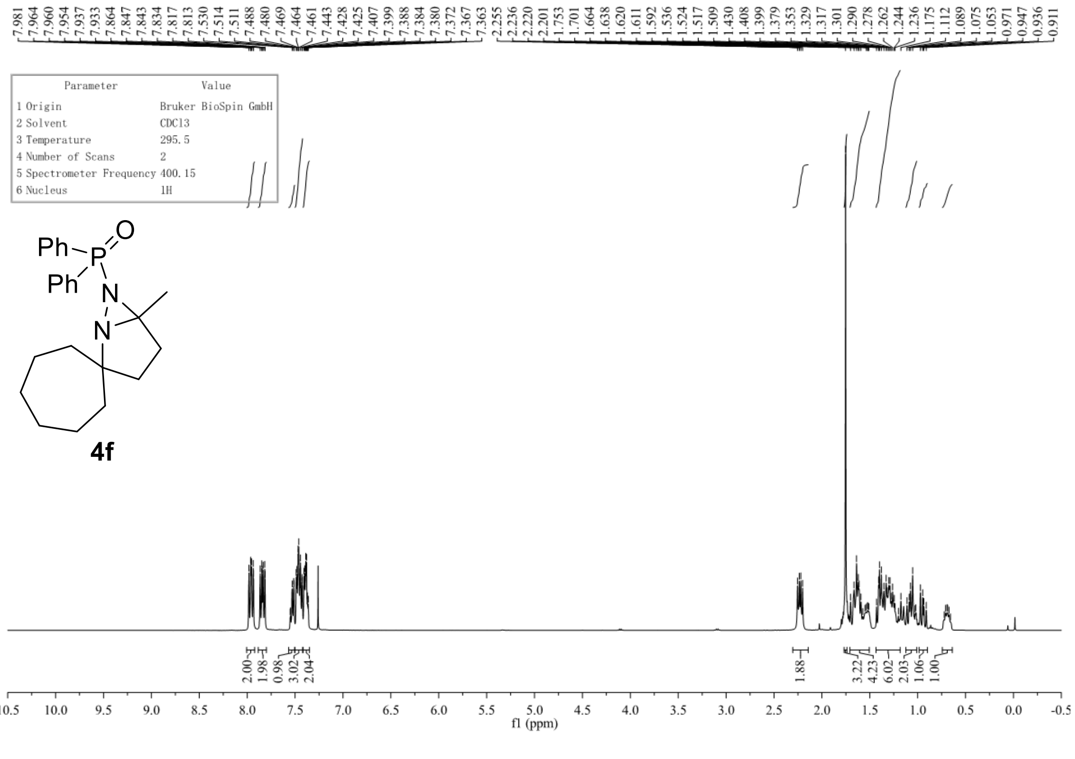
Parameter
Value
1 Origin
Bruker BioSpin GmbH
2 Solvent
CDCl3
3 Temperature
295.5
4 Number of Scans
2
5 Spectrometer Frequency
400.15
6 Nucleus
1H
4f
fl (ppm)

Parameter	Value
1 Origin	Bruker BioSpin GmbH
2 Solvent	CDCl3
3 Temperature	296.0
4 Number of Scans	80
5 Spectrometer Frequency	100.62
6 Nucleus	13C

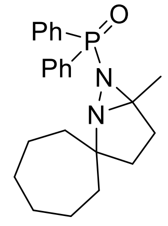

4f

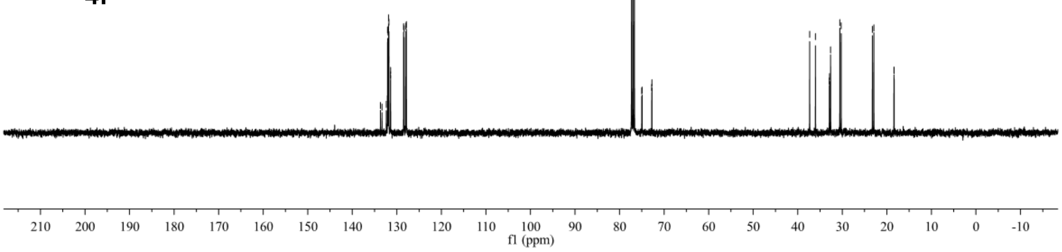

Parameter	Value
1 Origin	Bruker BioSpin GmbH
2 Solvent	CDCl3
3 Temperature	295.8
4 Number of Scans	2
5 Spectrometer Frequency	161.98
6 Nucleus	31P

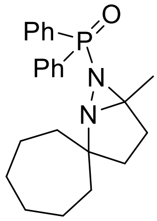

4f

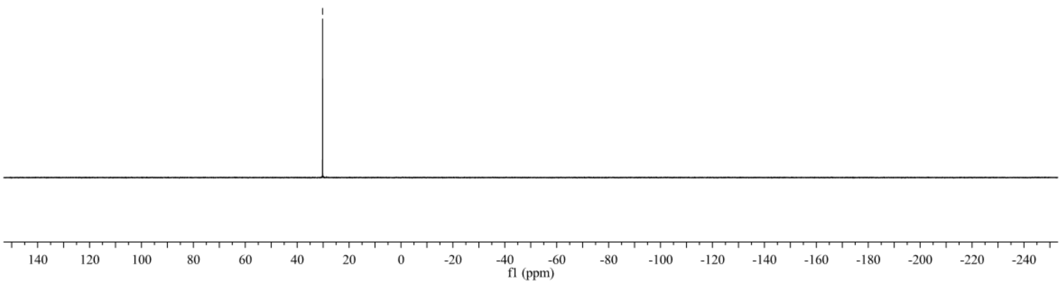

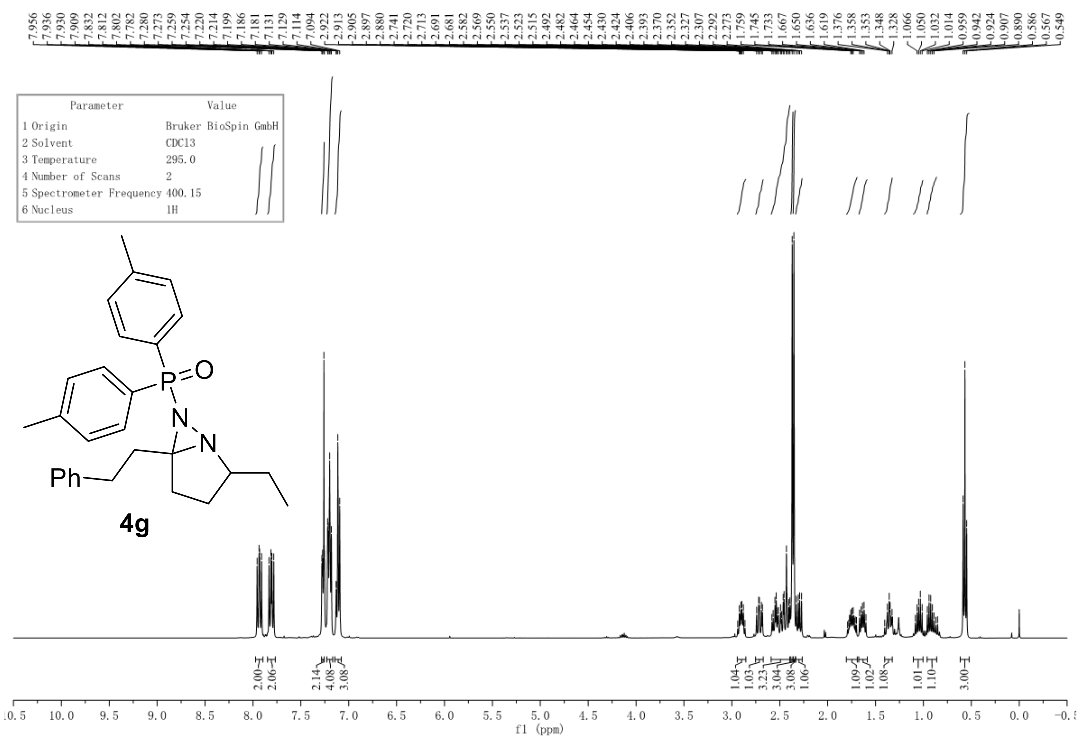

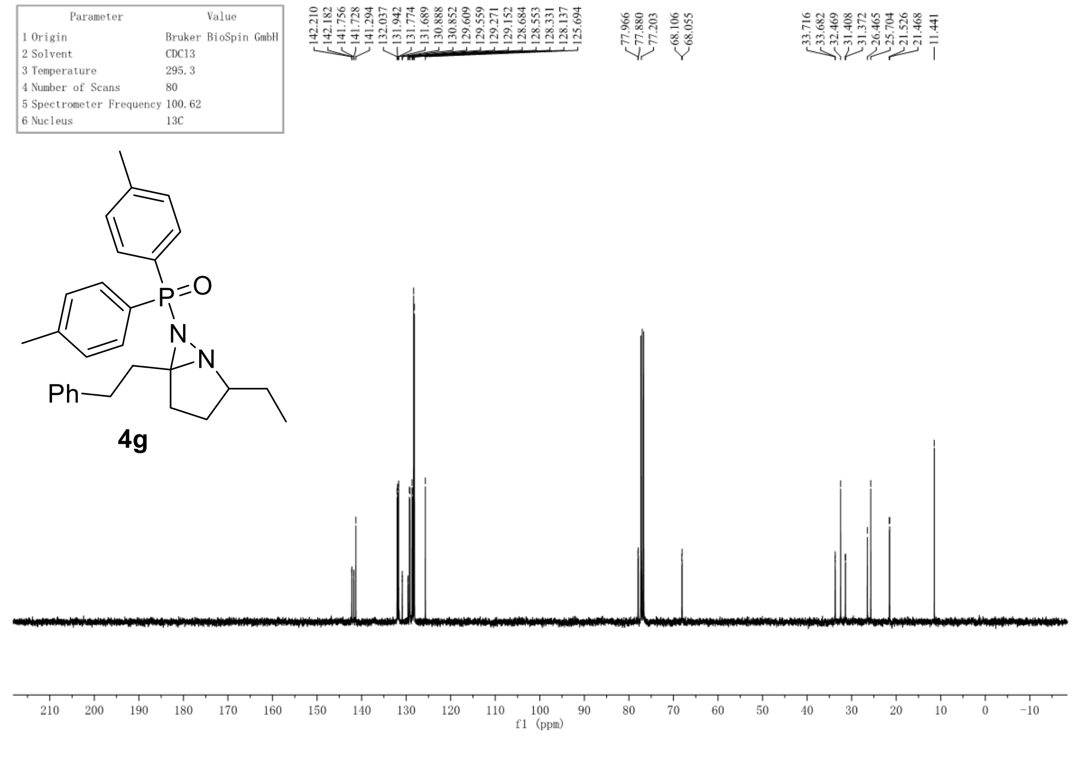

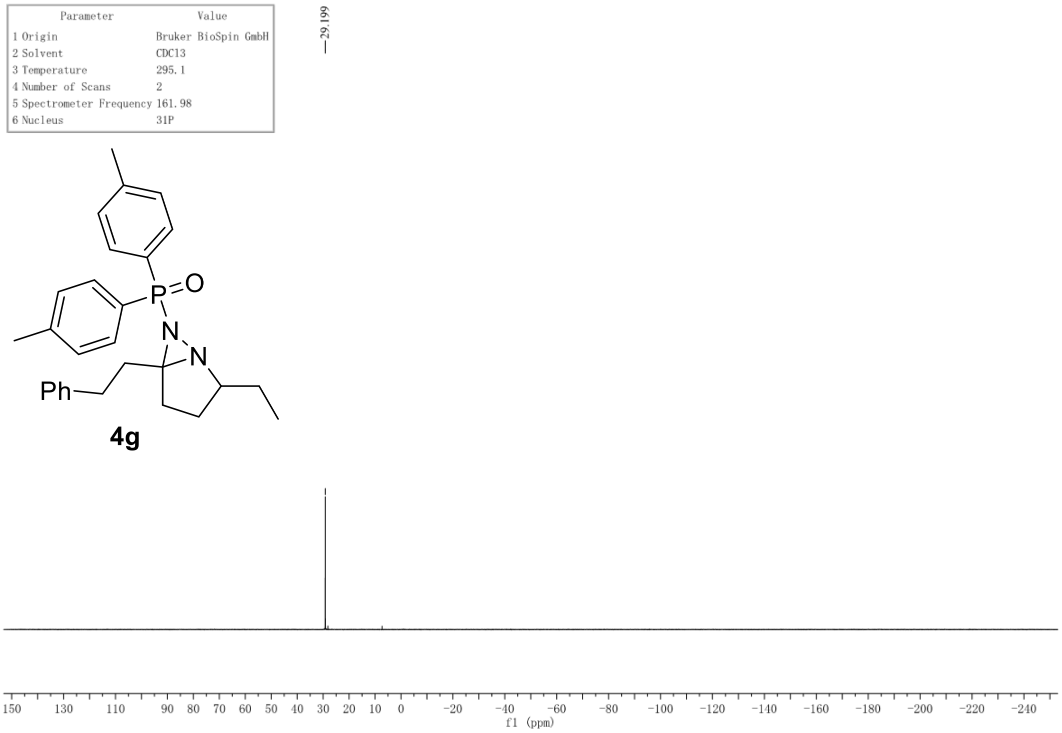
Parameter Value
1 Origin Bruker BioSpin GmbH
2 Solvent CDCl3
3 Temperature 295.1
4 Number of Scans 2
5 Spectrometer Frequency 161.98
6 Nucleus 31P
—29.199
P=O
N
N
Ph
4g
150 130 110 90 80 70 60 50 40 30 20 10 0 −20 −40 −60 −80 −100 −120 −140 −160 −180 −200 −220 −240
f1 (ppm)

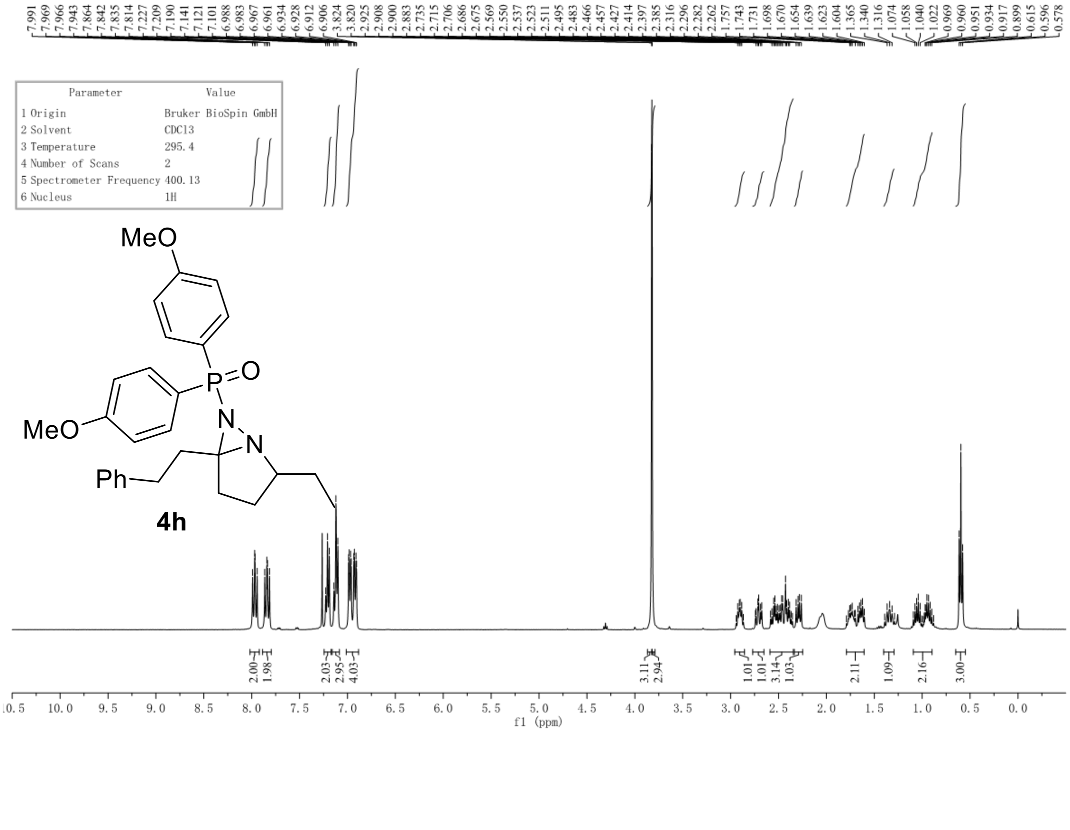
7.991 7.969 7.966 7.943 7.864 7.842 7.835 7.814 7.227 7.209 7.190 7.141 7.121 7.101 6.988 6.983 6.967 6.961 6.934 6.928 6.912 6.906 3.824 3.820 2.925 2.908 2.900 2.883 2.735 2.715 2.706 2.686 2.675 2.569 2.550 2.537 2.523 2.511 2.495 2.483 2.466 2.457 2.427 2.414 2.397 2.385 2.316 2.296 2.282 2.262 1.757 1.743 1.731 1.698 1.670 1.654 1.639 1.623 1.604 1.365 1.340 1.316 1.074 1.058 1.040 1.022 0.969 0.960 0.951 0.934 0.917 0.899 0.615 0.596 0.578
Parameter Value
1 Origin Bruker BioSpin GmbH
2 Solvent CDCl3
3 Temperature 295.4
4 Number of Scans 2
5 Spectrometer Frequency 400.13
6 Nucleus 1H
MeO
MeO
P=O
N
N
Ph
4h
2.00 1.98 2.03 2.95 4.03 3.11 2.94 1.01 1.01 3.14 1.03 2.11 1.09 2.16 3.00
10.5 10.0 9.5 9.0 8.5 8.0 7.5 7.0 6.5 6.0 5.5 5.0 4.5 4.0 3.5 3.0 2.5 2.0 1.5 1.0 0.5 0.0
f1 (ppm)

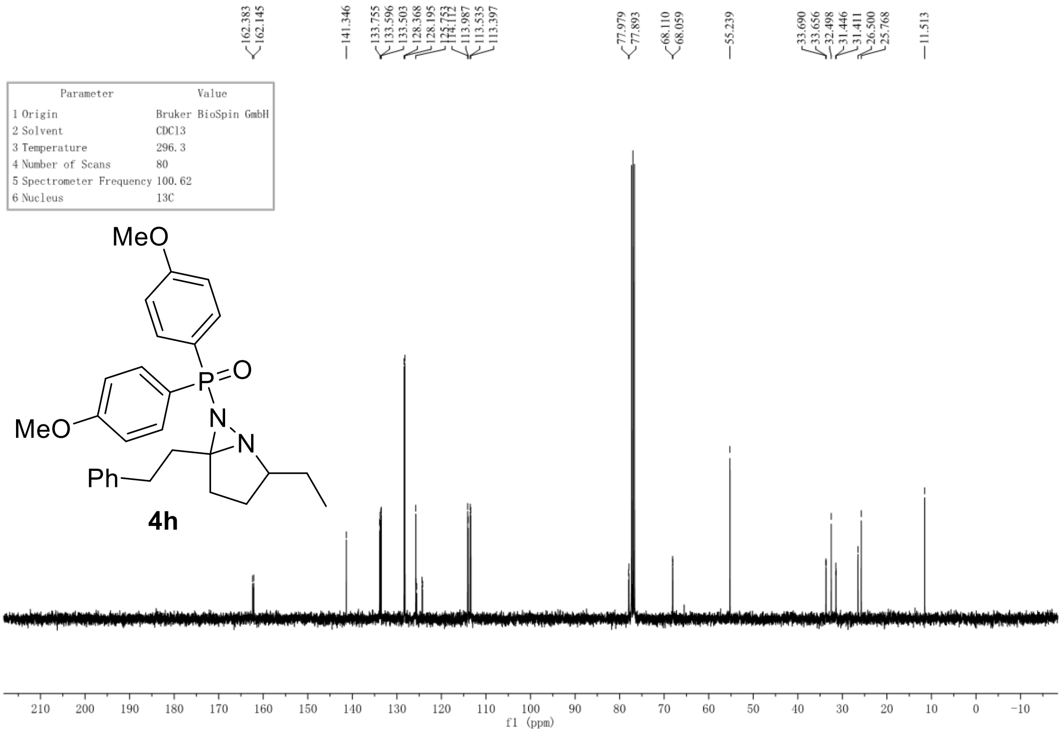
162.383
162.145
141.346
133.755
133.596
133.503
128.368
128.195
125.753
114.112
113.987
113.535
113.397
77.979
77.893
68.110
68.059
55.239
33.690
33.656
32.498
31.446
31.411
26.500
25.768
11.513
Parameter Value
1 Origin Bruker BioSpin GmbH
2 Solvent CDCl3
3 Temperature 296.3
4 Number of Scans 80
5 Spectrometer Frequency 100.62
6 Nucleus 13C
MeO
MeO
P
O
N
N
Ph
4h
f1 (ppm)

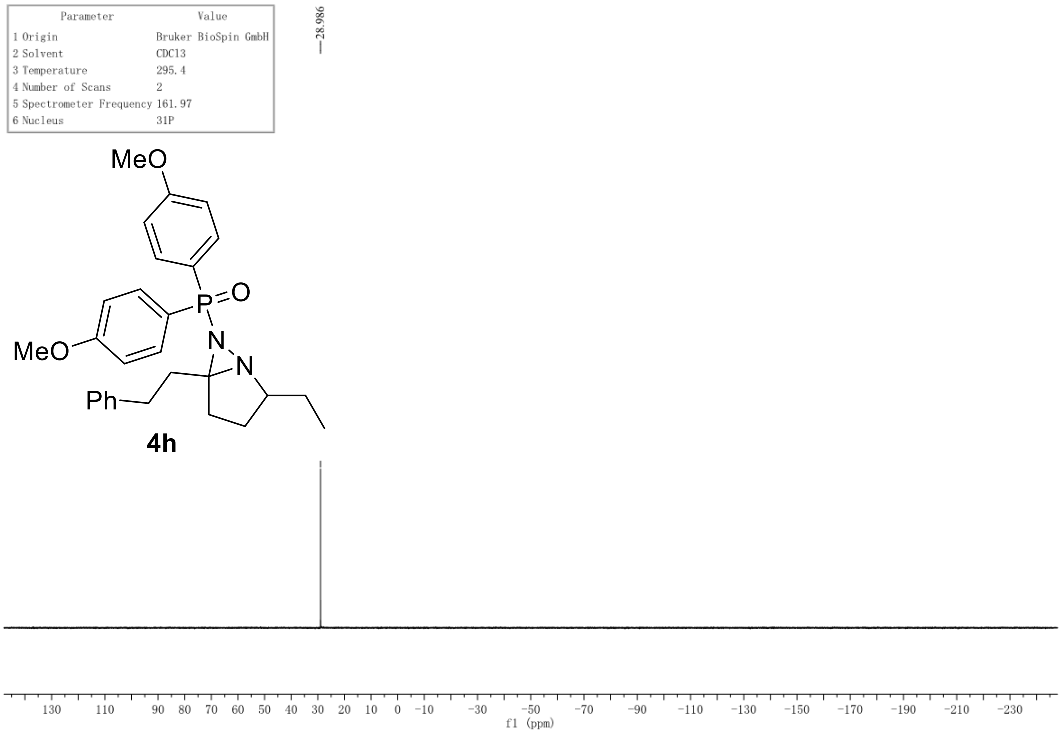
28.986
Parameter Value
1 Origin Bruker BioSpin GmbH
2 Solvent CDCl3
3 Temperature 295.4
4 Number of Scans 2
5 Spectrometer Frequency 161.97
6 Nucleus 31P
MeO
MeO
P
O
N
N
Ph
4h
f1 (ppm)

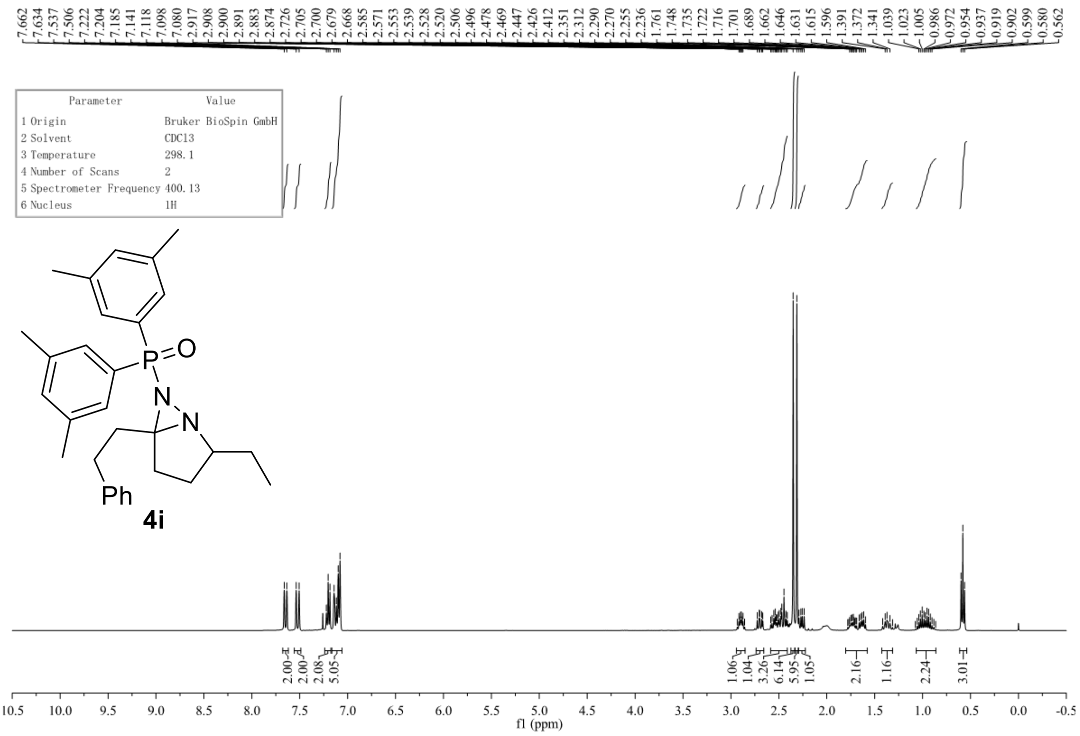
Parameter Value
1 Origin Bruker BioSpin GmbH
2 Solvent CDCl3
3 Temperature 298.1
4 Number of Scans 2
5 Spectrometer Frequency 400.13
6 Nucleus 1H
4i
fl (ppm)

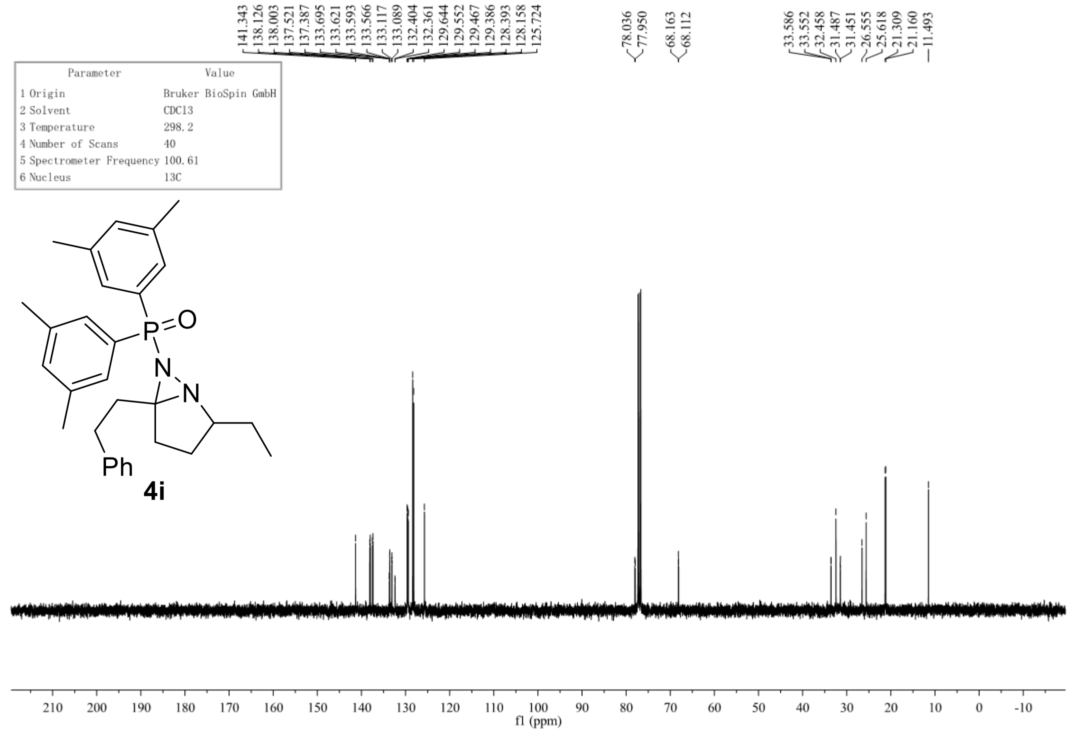
Parameter Value
1 Origin Bruker BioSpin GmbH
2 Solvent CDCl3
3 Temperature 298.2
4 Number of Scans 40
5 Spectrometer Frequency 100.61
6 Nucleus 13C
4i
fl (ppm)

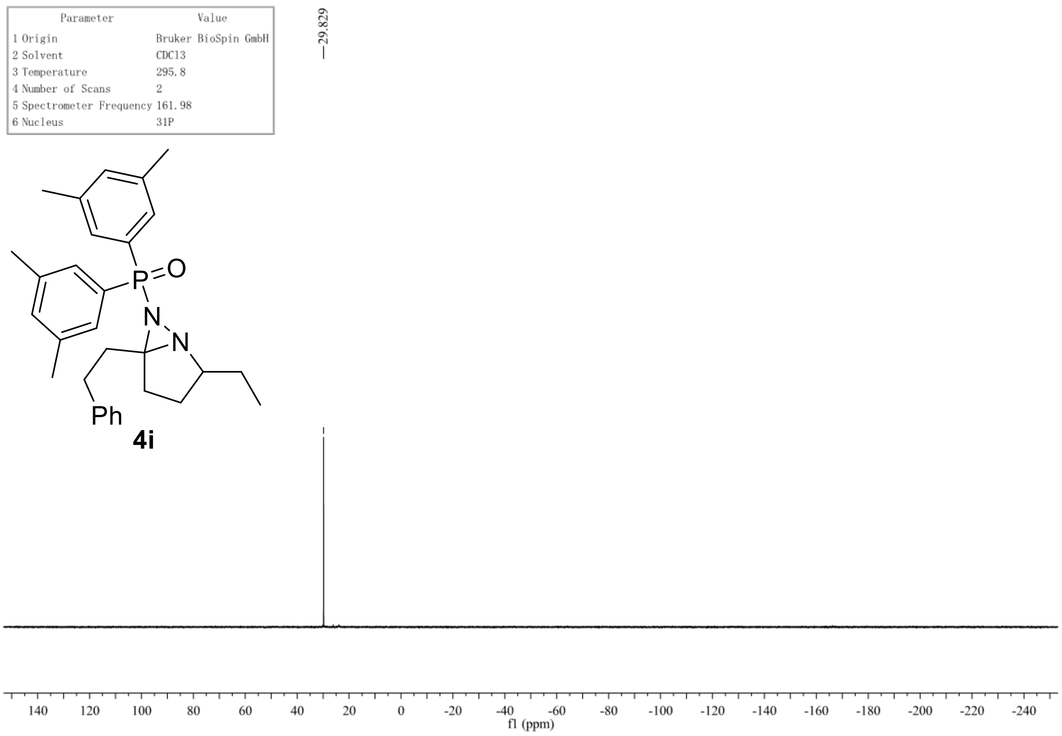
Parameter Value
1 Origin Bruker BioSpin GmbH
2 Solvent CDCl3
3 Temperature 295.8
4 Number of Scans 2
5 Spectrometer Frequency 161.98
6 Nucleus 31P
29.829
4i
f1 (ppm)

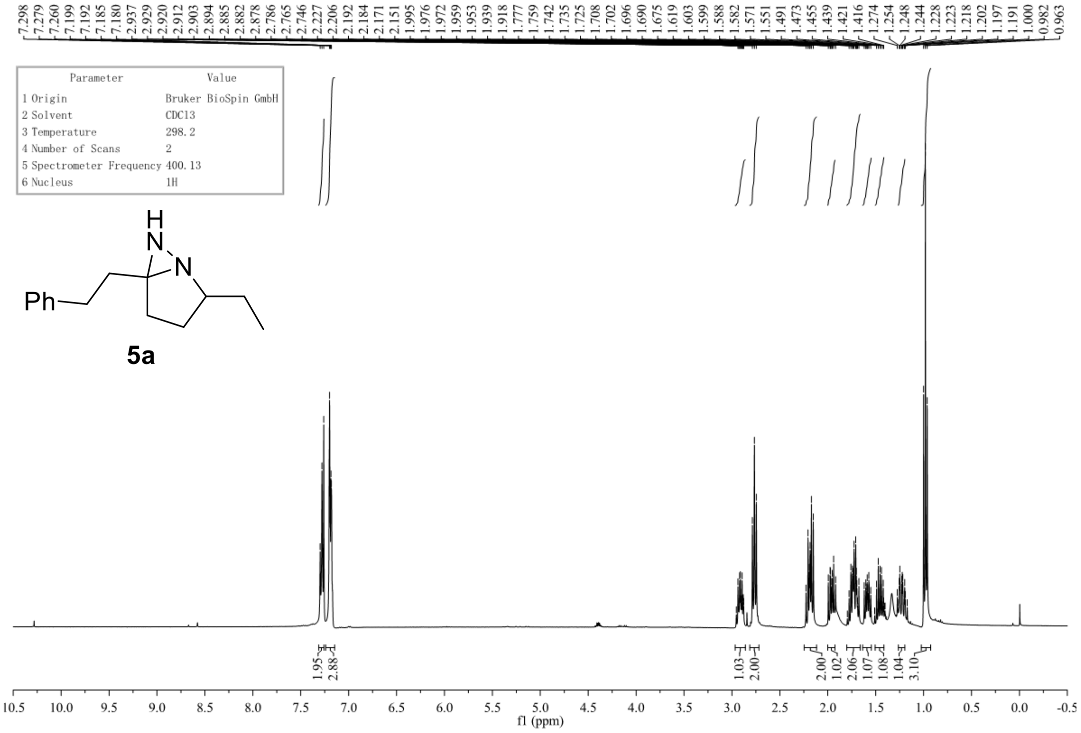
Parameter Value
1 Origin Bruker BioSpin GmbH
2 Solvent CDCl3
3 Temperature 298.2
4 Number of Scans 2
5 Spectrometer Frequency 400.13
6 Nucleus 1H
7.298 7.279 7.260 7.199 7.192 7.185 7.180 2.937 2.929 2.920 2.912 2.903 2.894 2.885 2.882 2.878 2.786 2.765 2.746 2.227 2.206 2.192 2.184 2.171 2.151 1.995 1.976 1.972 1.959 1.953 1.939 1.918 1.777 1.759 1.742 1.735 1.725 1.708 1.702 1.696 1.690 1.675 1.619 1.603 1.599 1.588 1.582 1.571 1.551 1.491 1.473 1.455 1.439 1.421 1.416 1.274 1.254 1.248 1.244 1.228 1.223 1.218 1.202 1.197 1.191 1.000 0.982 0.963
1.95 2.88 1.03 2.00 2.00 1.02 2.06 1.07 1.08 1.04 3.10
5a
f1 (ppm)

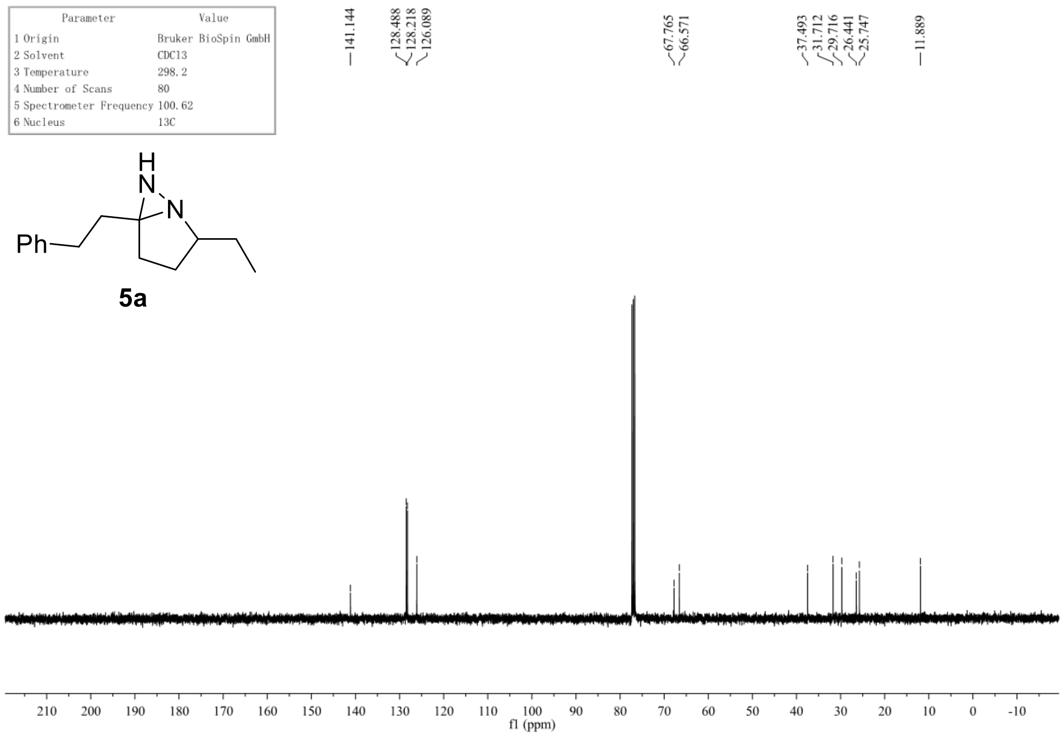
Parameter Value
1 Origin Bruker BioSpin GmbH
2 Solvent CDCl3
3 Temperature 298.2
4 Number of Scans 80
5 Spectrometer Frequency 100.62
6 Nucleus 13C
141.144
128.488
128.218
126.089
67.765
66.571
37.493
31.712
29.716
26.441
25.747
11.889
H
N
N
Ph
5a
210 200 190 180 170 160 150 140 130 120 110 100 90 80 70 60 50 40 30 20 10 0 -10
f1 (ppm)

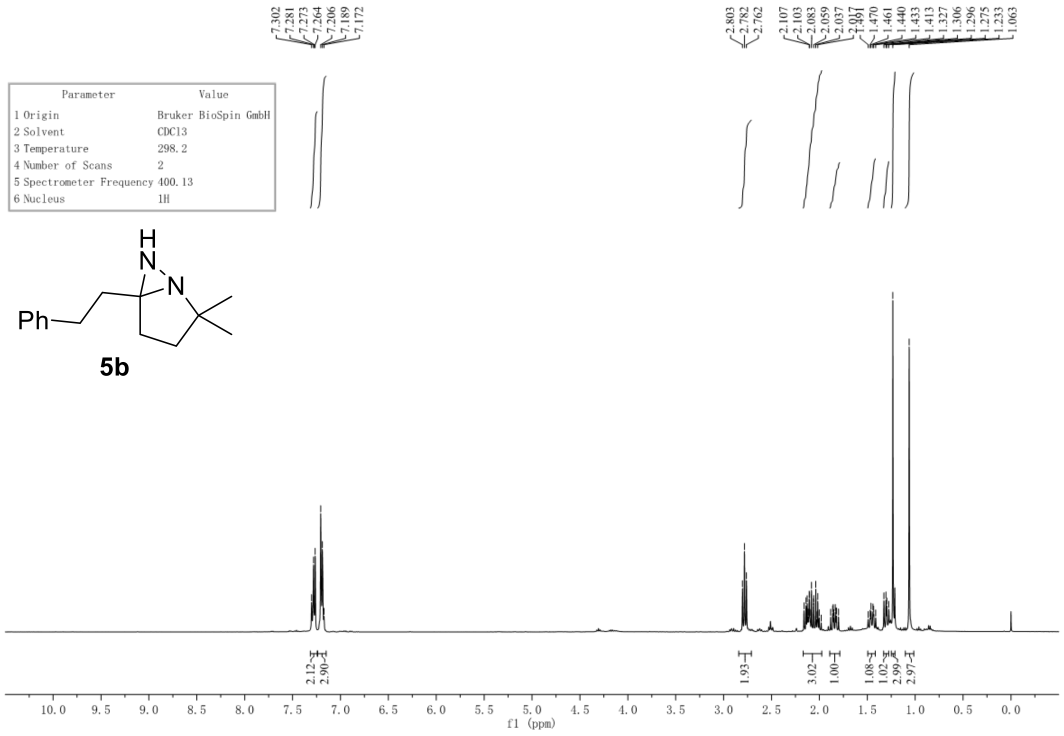
7.302
7.281
7.273
7.264
7.206
7.189
7.172
2.803
2.782
2.762
2.107
2.103
2.083
2.059
2.037
2.017
1.991
1.470
1.461
1.440
1.433
1.413
1.327
1.306
1.296
1.275
1.233
1.063
Parameter Value
1 Origin Bruker BioSpin GmbH
2 Solvent CDCl3
3 Temperature 298.2
4 Number of Scans 2
5 Spectrometer Frequency 400.13
6 Nucleus 1H
H
N
N
Ph
5b
2.12
2.90
1.93
3.02
1.00
1.08
1.02
2.99
2.97
10.0 9.5 9.0 8.5 8.0 7.5 7.0 6.5 6.0 5.5 5.0 4.5 4.0 3.5 3.0 2.5 2.0 1.5 1.0 0.5 0.0
f1 (ppm)

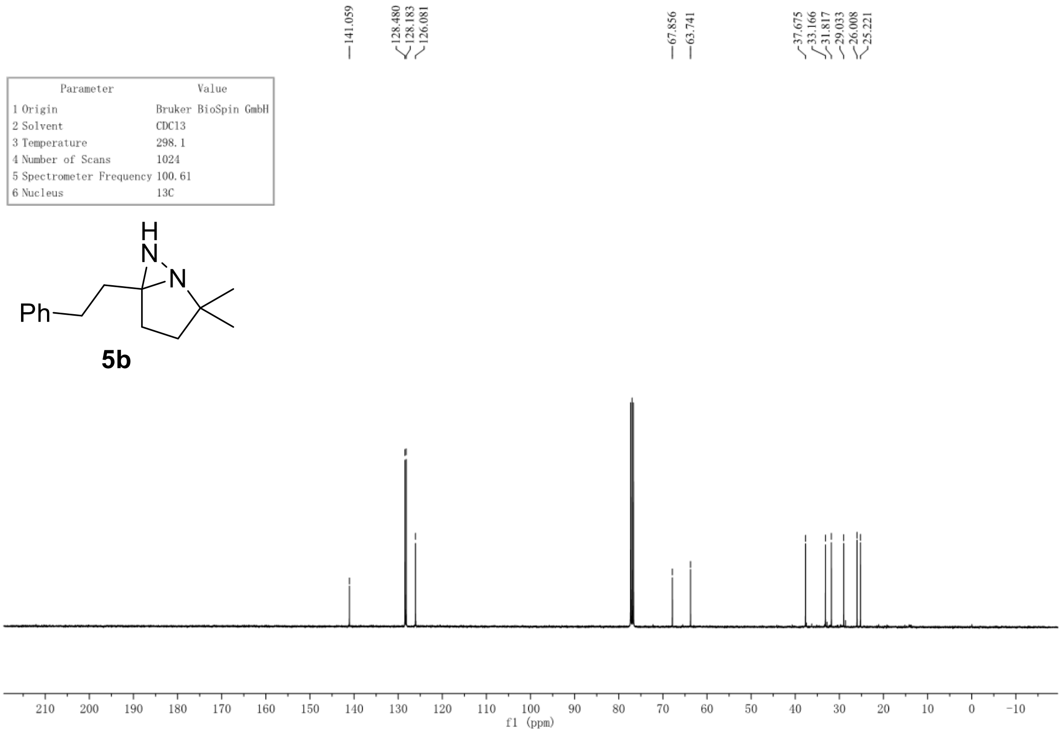
141.059
128.480
128.183
126.081
67.856
63.741
37.675
33.166
31.817
29.033
26.008
25.221
Parameter Value
1 Origin Bruker BioSpin GmbH
2 Solvent CDCl3
3 Temperature 298.1
4 Number of Scans 1024
5 Spectrometer Frequency 100.61
6 Nucleus 13C
H
N
N
Ph
f1 (ppm)

5b

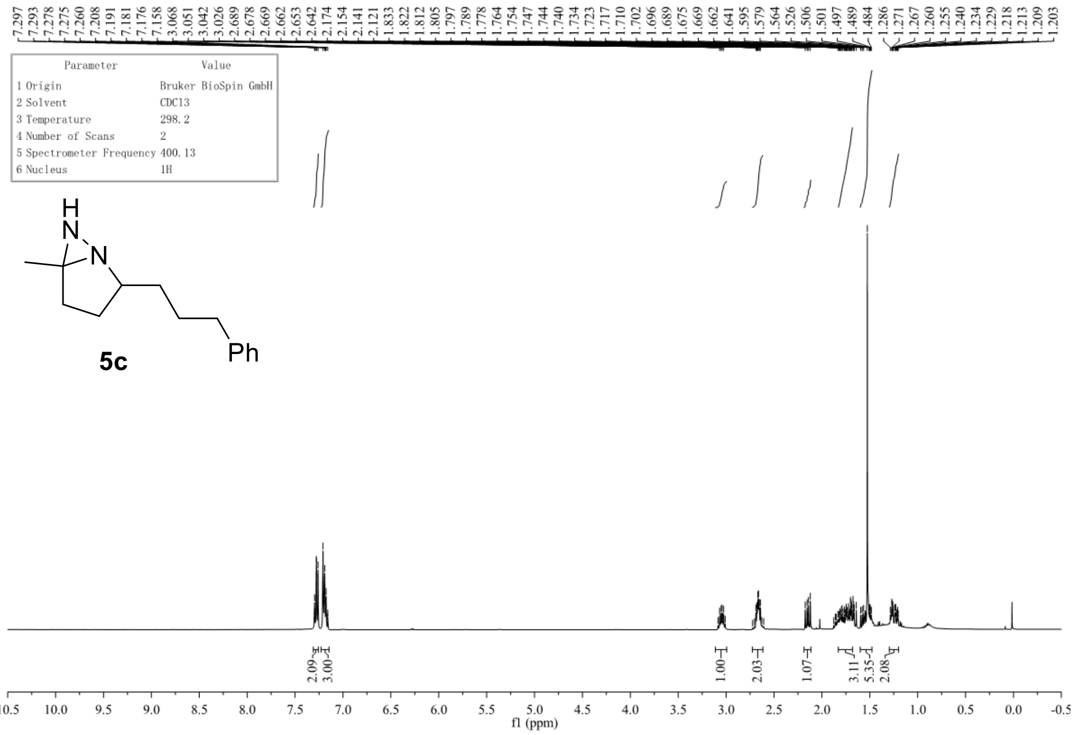
7.297
7.293
7.278
7.275
7.260
7.208
7.191
7.181
7.176
7.158
3.068
3.051
3.042
3.026
2.689
2.678
2.669
2.662
2.653
2.642
2.174
2.154
2.141
2.121
1.833
1.822
1.812
1.805
1.797
1.789
1.778
1.764
1.754
1.747
1.744
1.740
1.734
1.723
1.717
1.710
1.702
1.696
1.689
1.675
1.669
1.662
1.641
1.595
1.579
1.564
1.526
1.506
1.501
1.497
1.489
1.484
1.286
1.271
1.267
1.260
1.255
1.240
1.234
1.229
1.218
1.213
1.209
1.203
Parameter Value
1 Origin Bruker BioSpin GmbH
2 Solvent CDCl3
3 Temperature 298.2
4 Number of Scans 2
5 Spectrometer Frequency 400.13
6 Nucleus 1H
H
N
N
Ph
2.09
3.00
1.00
2.03
1.07
3.11
5.35
2.08
f1 (ppm)

5c

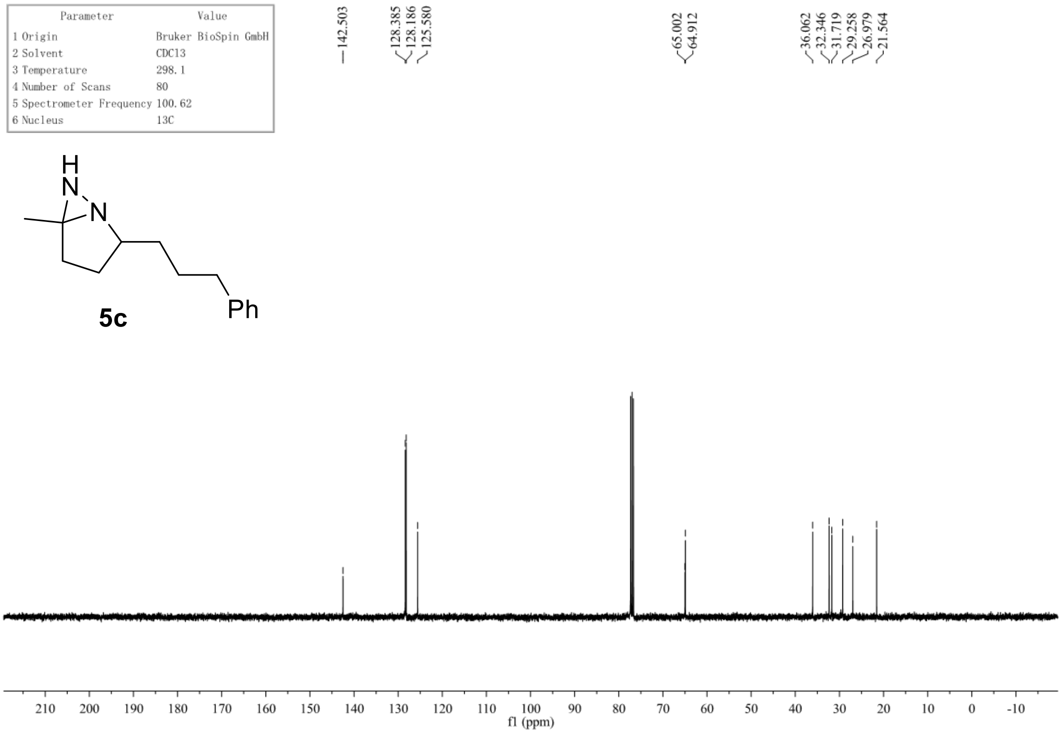
Parameter Value
1 Origin Bruker BioSpin GmbH
2 Solvent CDCl3
3 Temperature 298.1
4 Number of Scans 80
5 Spectrometer Frequency 100.62
6 Nucleus 13C
142.503
128.385
128.186
125.580
65.002
64.912
36.062
32.346
31.719
29.258
26.979
21.564
H
N
N
Ph
5c
f1 (ppm)

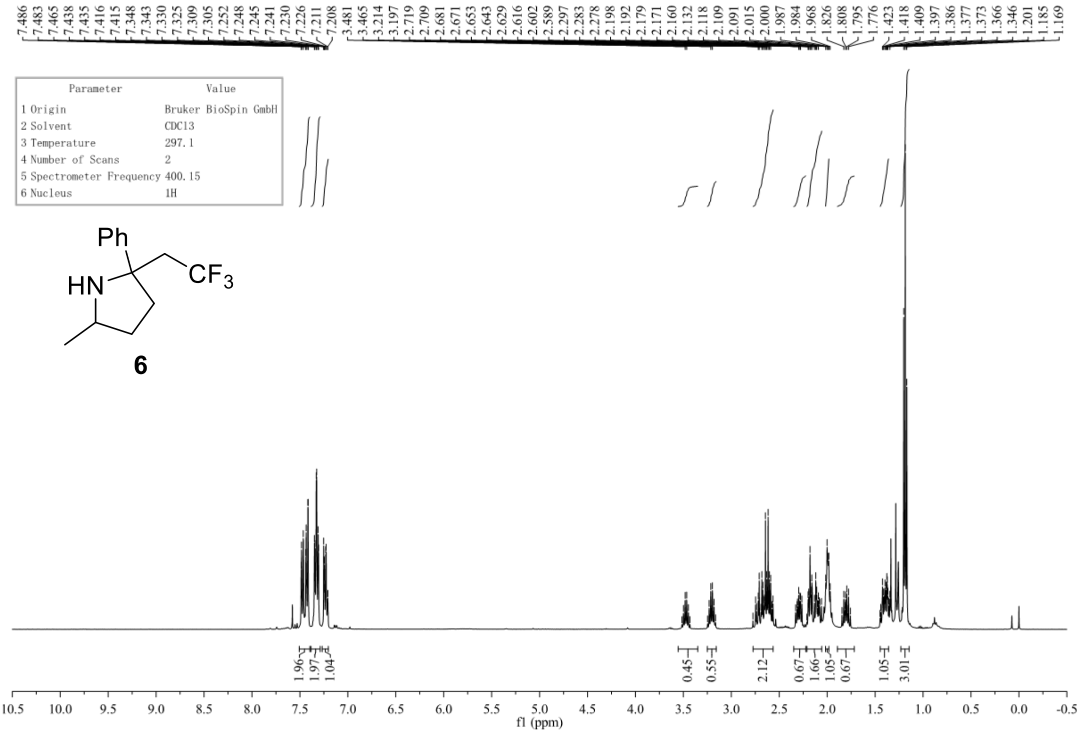
7.486
7.483
7.465
7.438
7.435
7.416
7.415
7.348
7.343
7.330
7.325
7.309
7.305
7.252
7.248
7.245
7.241
7.230
7.226
7.211
7.208
3.481
3.465
3.214
3.197
2.719
2.709
2.681
2.671
2.653
2.643
2.629
2.616
2.602
2.589
2.297
2.283
2.278
2.198
2.192
2.179
2.171
2.160
2.132
2.118
2.109
2.091
2.015
2.000
1.987
1.984
1.968
1.826
1.808
1.795
1.776
1.423
1.418
1.409
1.397
1.386
1.377
1.373
1.366
1.346
1.201
1.185
1.169
Parameter Value
1 Origin Bruker BioSpin GmbH
2 Solvent CDCl3
3 Temperature 297.1
4 Number of Scans 2
5 Spectrometer Frequency 400.15
6 Nucleus 1H
Ph
HN
CF3
6
1.96
1.97
1.04
0.45
0.55
2.12
0.67
1.66
1.05
0.67
1.05
3.01
f1 (ppm)

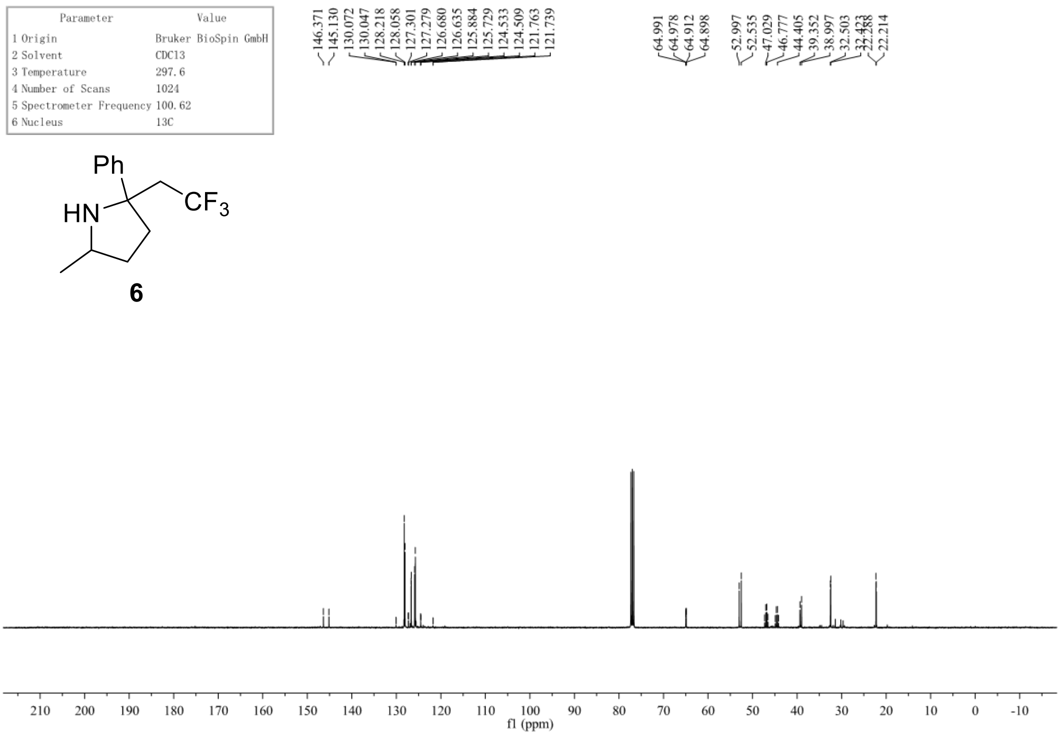
Parameter
Value
1 Origin
Bruker BioSpin GmbH
2 Solvent
CDCl3
3 Temperature
297.6
4 Number of Scans
1024
5 Spectrometer Frequency
100.62
6 Nucleus
13C
146.371
145.130
130.072
130.047
128.218
128.058
127.301
127.279
126.680
126.635
125.884
125.729
124.533
124.509
121.763
121.739
64.991
64.978
64.912
64.898
52.997
52.535
47.029
46.777
44.405
39.352
38.997
32.503
32.423
22.288
22.214
Ph
HN
CF3
6
f1 (ppm)

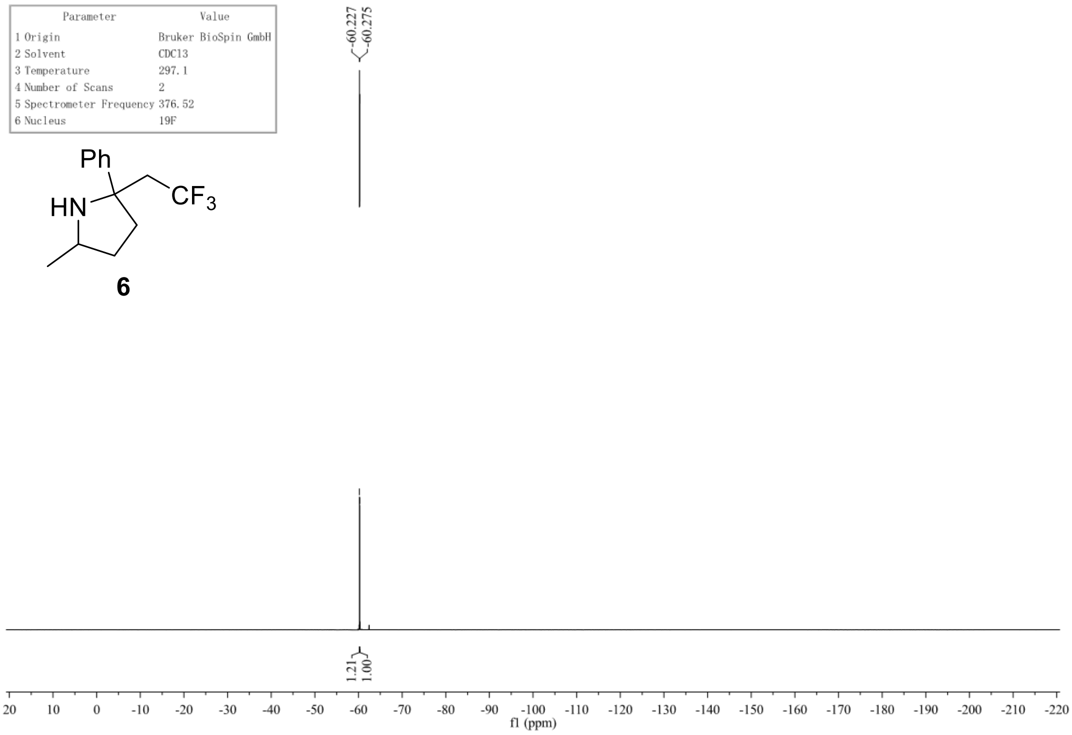
Parameter
Value
1 Origin
Bruker BioSpin GmbH
2 Solvent
CDCl3
3 Temperature
297.1
4 Number of Scans
2
5 Spectrometer Frequency
376.52
6 Nucleus
19F
-60.227
-60.275
Ph
HN
CF3
6
1.21
1.00
f1 (ppm)

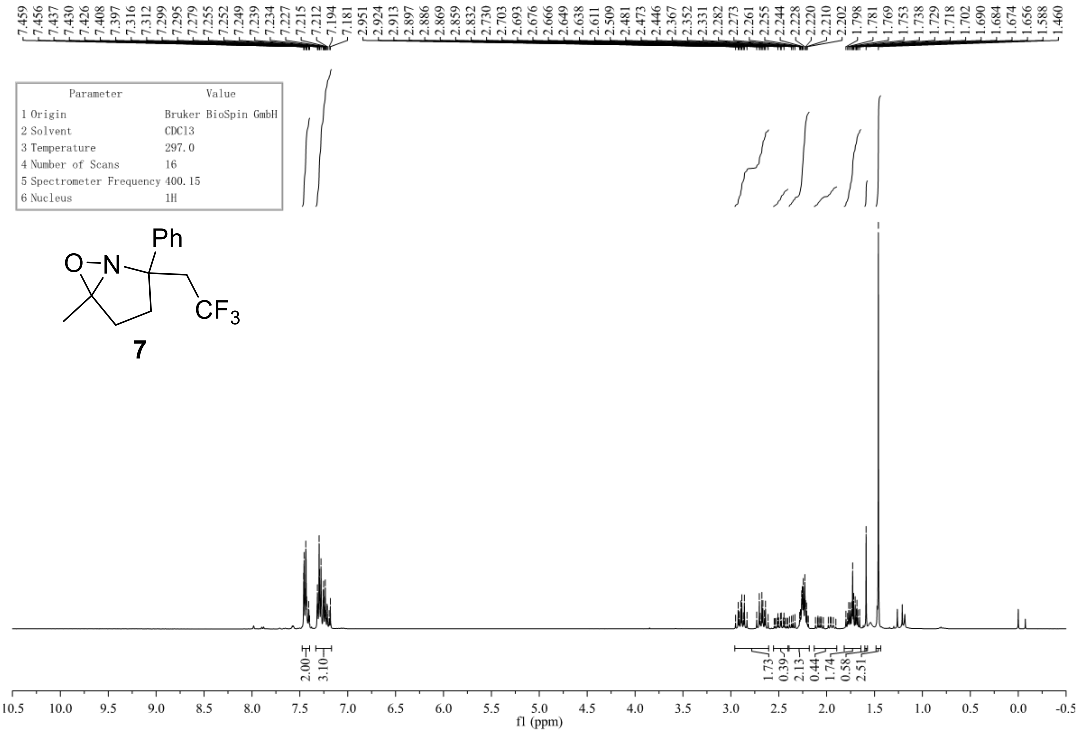
Parameter Value
1 Origin Bruker BioSpin GmbH
2 Solvent CDCl3
3 Temperature 297.0
4 Number of Scans 16
5 Spectrometer Frequency 400.15
6 Nucleus 1H
Ph
O–N
CF3
7
fl (ppm)

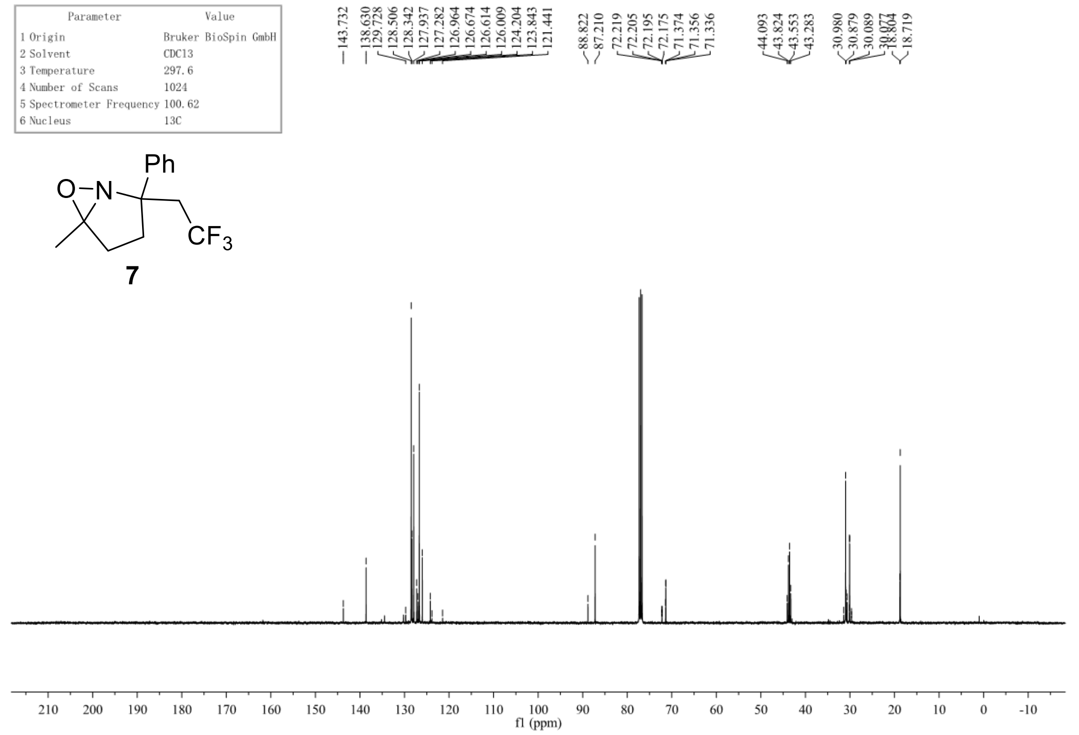
Parameter Value
1 Origin Bruker BioSpin GmbH
2 Solvent CDCl3
3 Temperature 297.6
4 Number of Scans 1024
5 Spectrometer Frequency 100.62
6 Nucleus 13C
Ph
O–N
CF3
7
fl (ppm)

Parameter	Value
1 Origin	Bruker BioSpin GmbH
2 Solvent	CDCl3
3 Temperature	297.1
4 Number of Scans	16
5 Spectrometer Frequency	376.52
6 Nucleus	19F

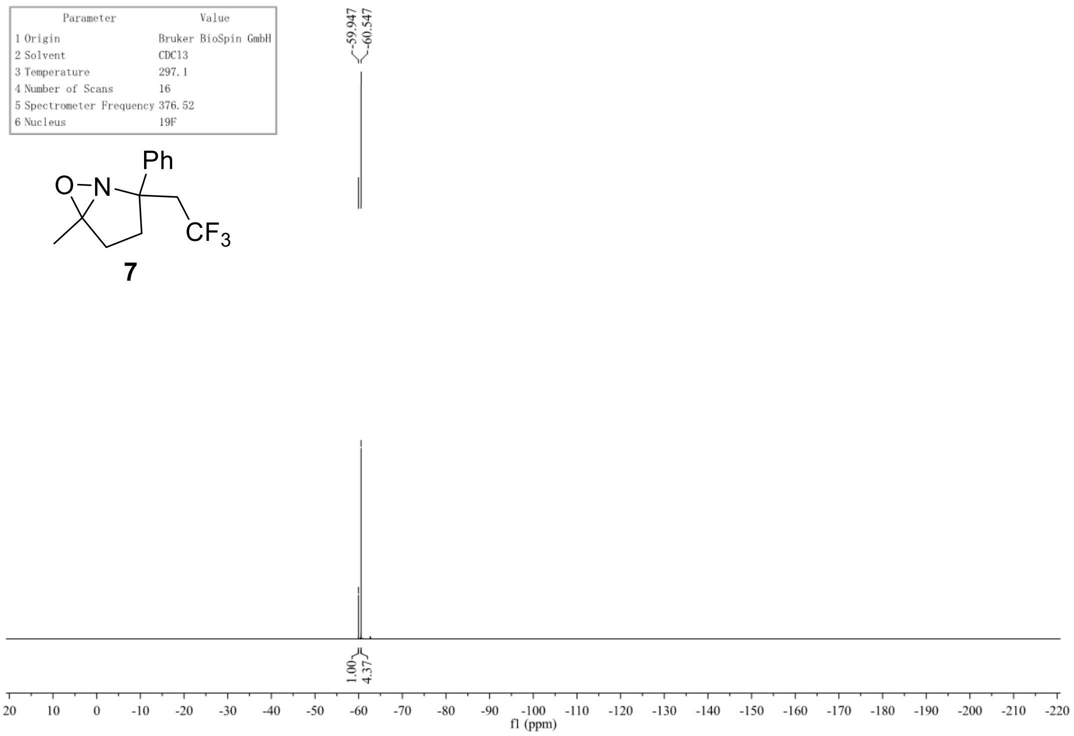

7

Parameter	Value
1 Origin	Bruker BioSpin GmbH
2 Solvent	CDCl3
3 Temperature	296.1
4 Number of Scans	2
5 Spectrometer Frequency	400.15
6 Nucleus	1H

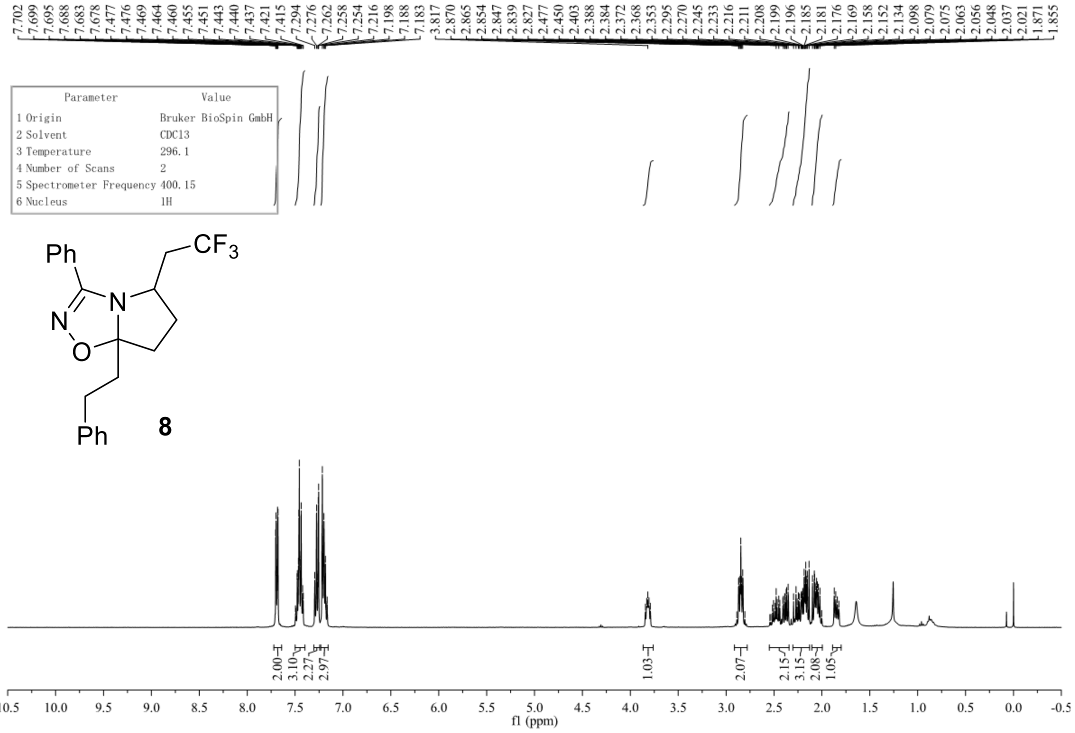

8

Parameter	Value
1 Origin	Bruker BioSpin GmbH
2 Solvent	CDCl3
3 Temperature	296.5
4 Number of Scans	1024
5 Spectrometer Frequency	100.62
6 Nucleus	13C

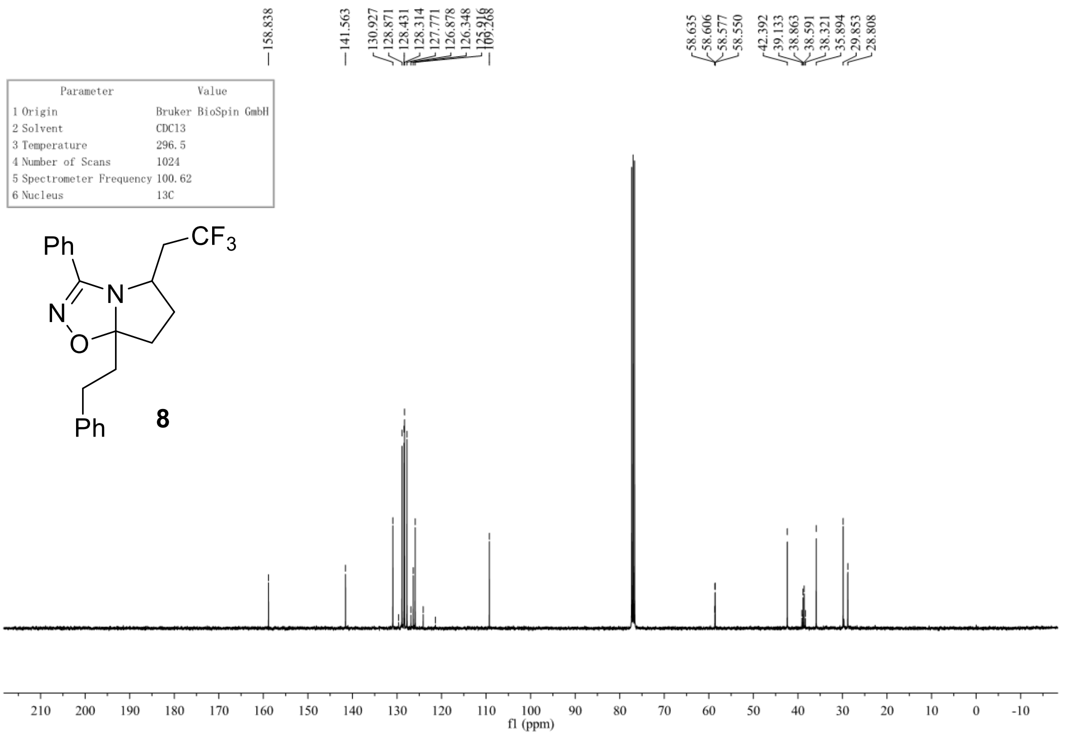

Parameter	Value
1 Origin	Bruker BioSpin GmbH
2 Solvent	CDCl3
3 Temperature	296.2
4 Number of Scans	2
5 Spectrometer Frequency	376.52
6 Nucleus	19F

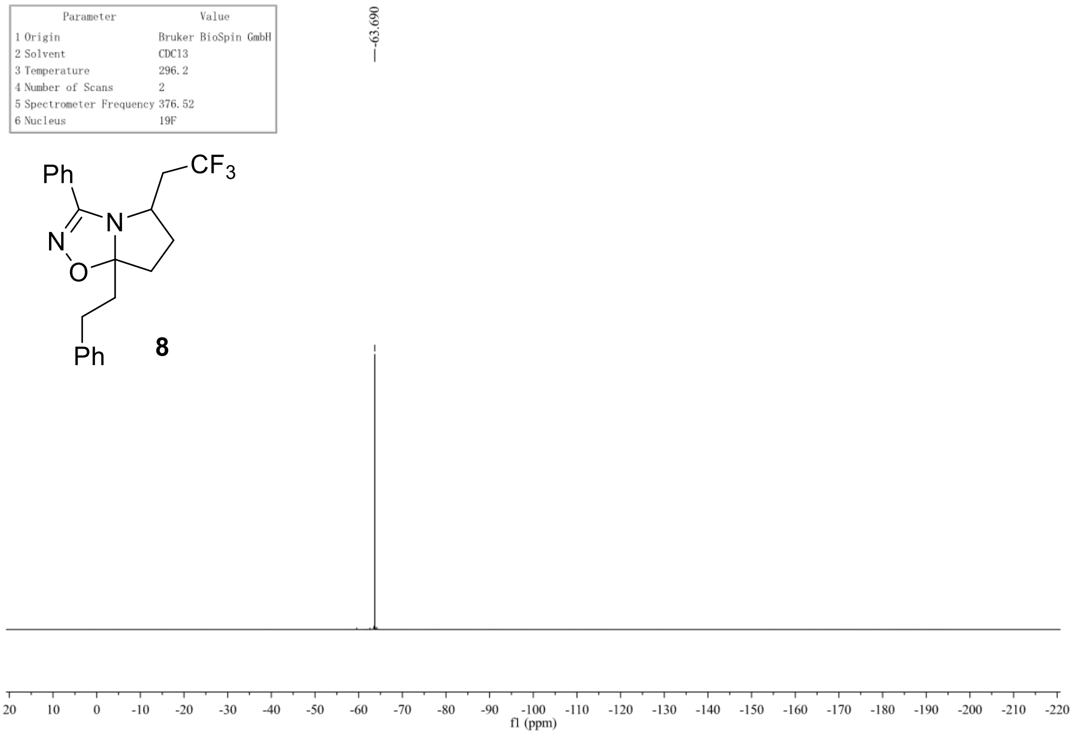

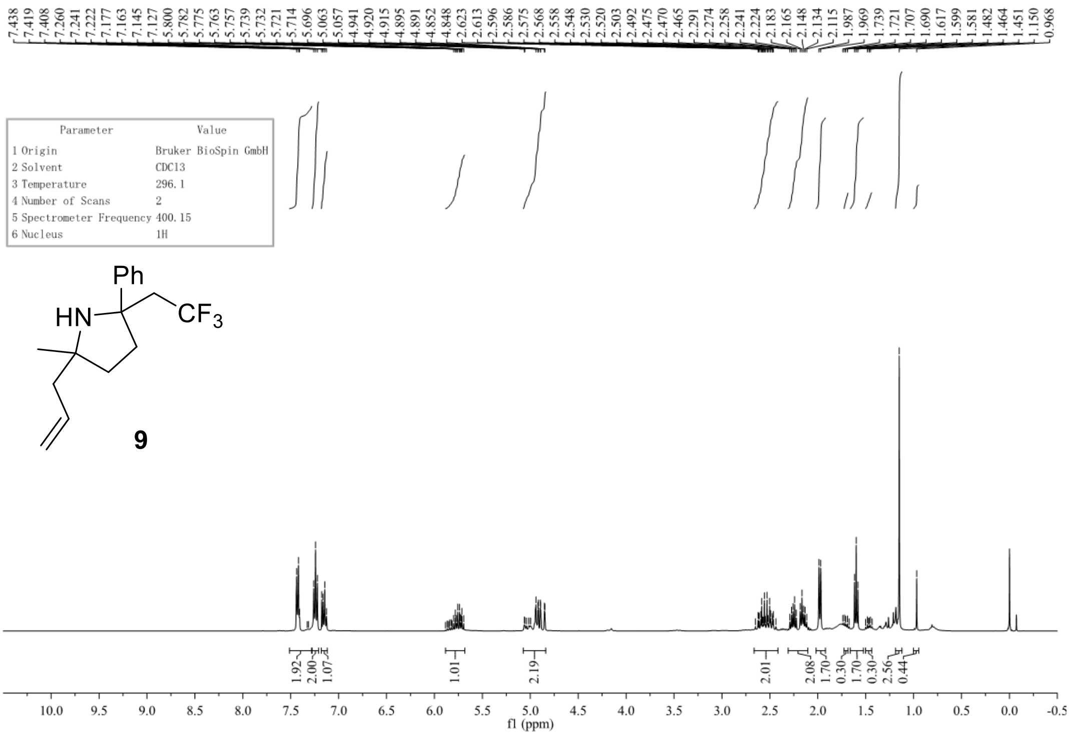

Parameter	Value
1 Origin	Bruker BioSpin GmbH
2 Solvent	CDCl3
3 Temperature	296.1
4 Number of Scans	2
5 Spectrometer Frequency	400.15
6 Nucleus	1H

9

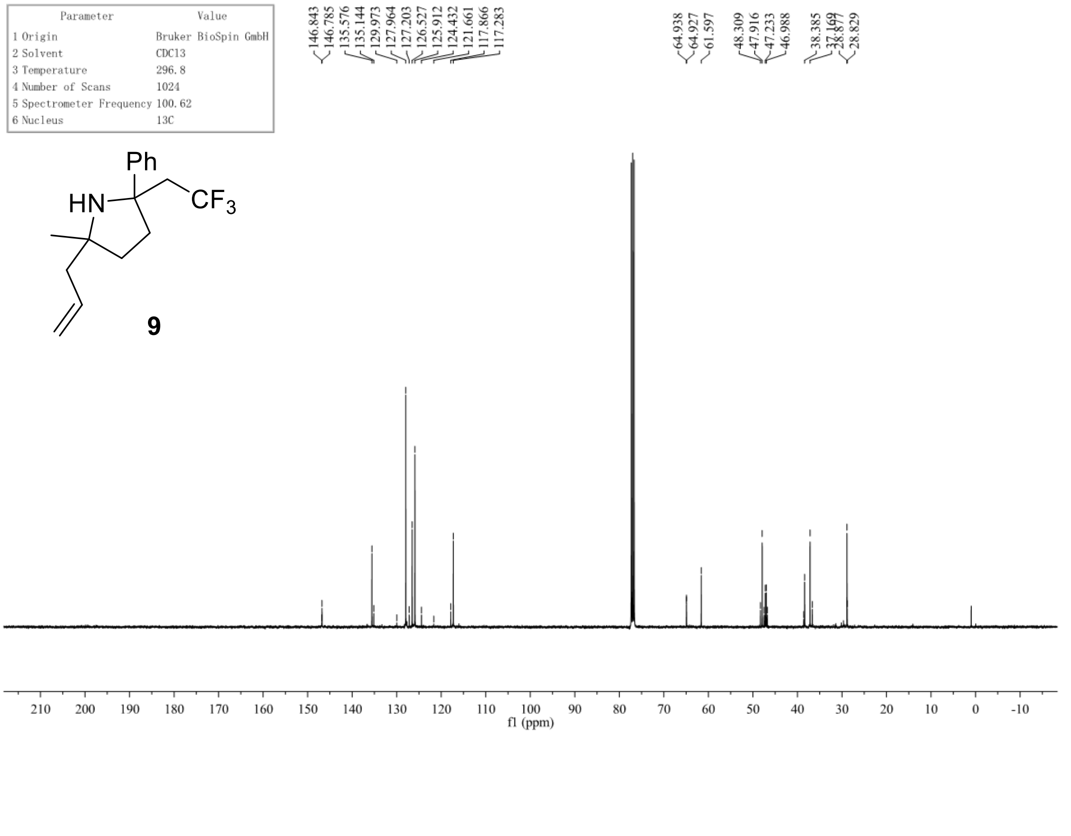

Parameter	Value
1 Origin	Bruker BioSpin GmbH
2 Solvent	CDCl3
3 Temperature	296.8
4 Number of Scans	1024
5 Spectrometer Frequency	100.62
6 Nucleus	13C

9

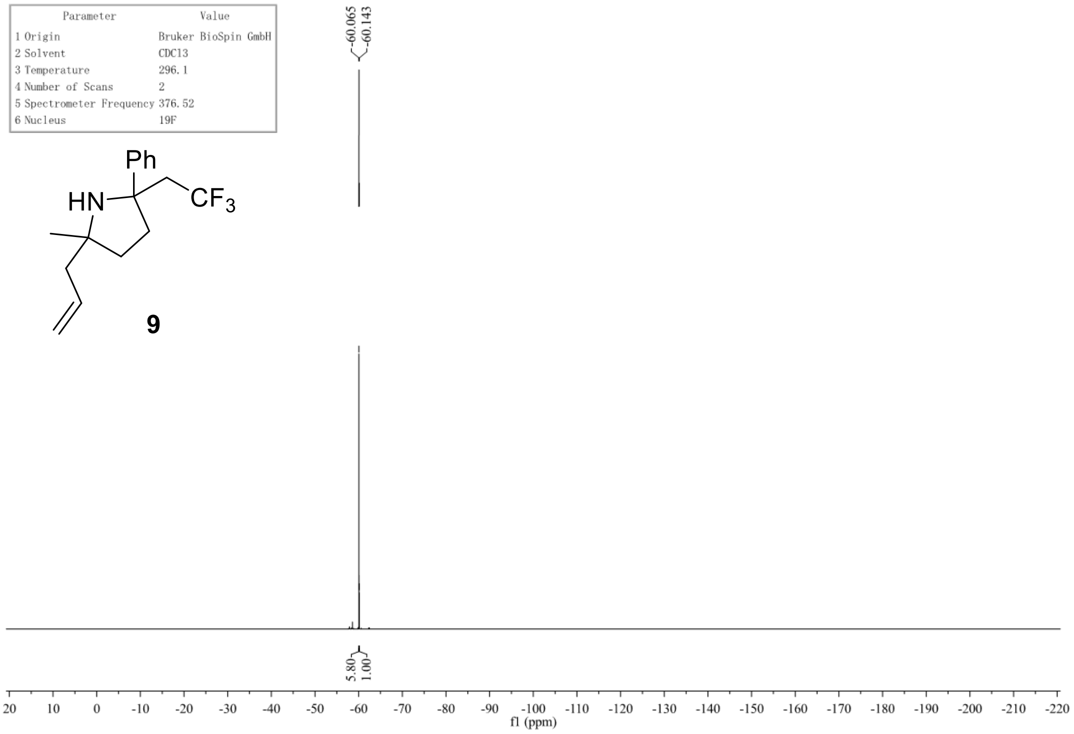
Parameter Value
1 Origin Bruker BioSpin GmbH
2 Solvent CDCl3
3 Temperature 296.1
4 Number of Scans 2
5 Spectrometer Frequency 376.52
6 Nucleus 19F
-60.065
-60.143
Ph
HN
CF3
9
5.80
1.00
fl (ppm)

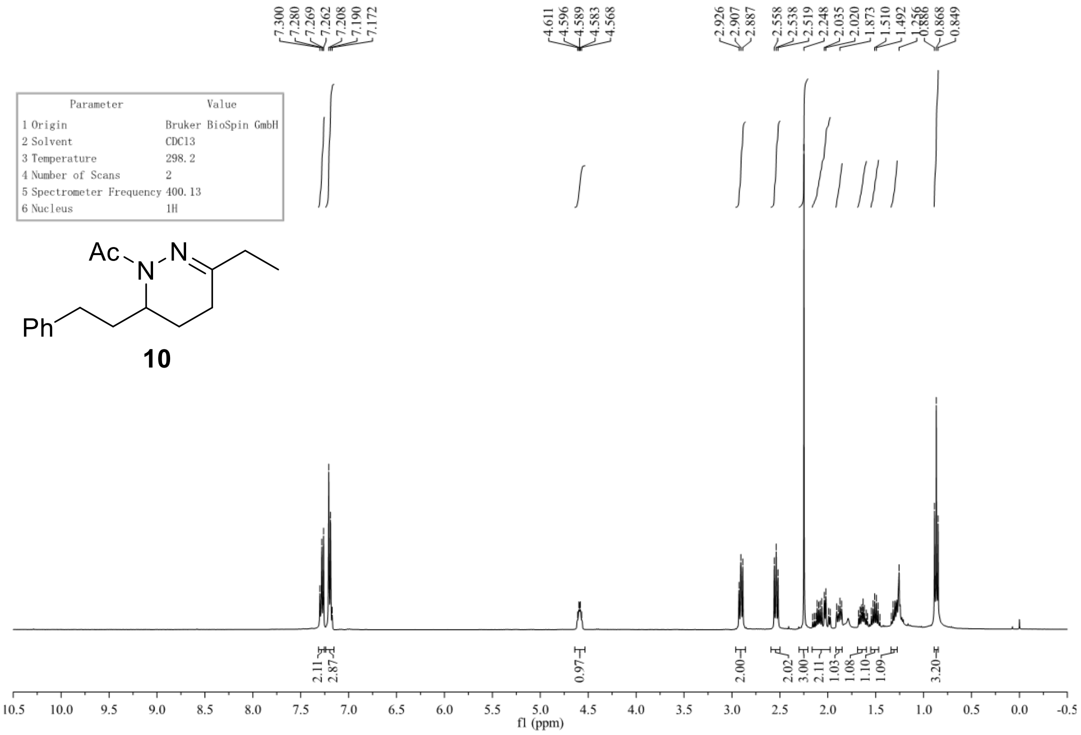
Parameter Value
1 Origin Bruker BioSpin GmbH
2 Solvent CDCl3
3 Temperature 298.2
4 Number of Scans 2
5 Spectrometer Frequency 400.13
6 Nucleus 1H
7.300
7.280
7.269
7.262
7.208
7.190
7.172
4.611
4.596
4.589
4.583
4.568
2.926
2.907
2.887
2.558
2.538
2.519
2.248
2.035
2.020
1.873
1.510
1.492
0.886
0.868
0.849
Ac
N
N
Ph
10
2.11
2.87
0.97
2.00
2.02
3.00
2.11
1.03
1.08
1.10
1.09
3.20
fl (ppm)

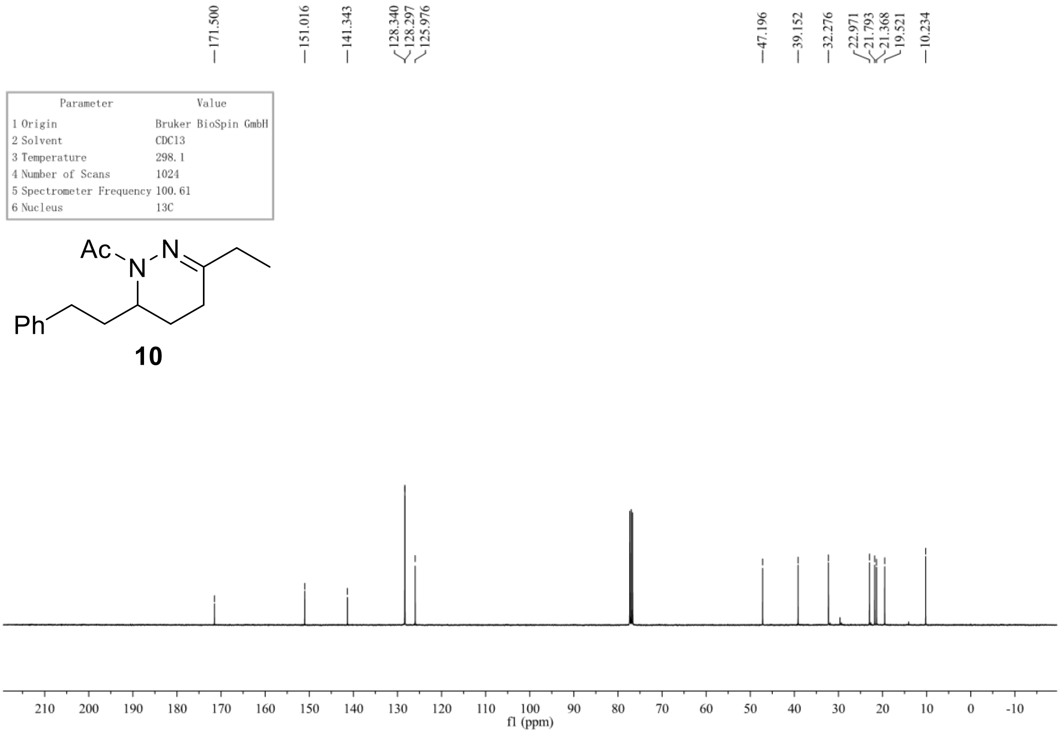
171.500
151.016
141.343
128.340
128.297
125.976
47.196
39.152
32.276
22.971
21.793
21.368
19.521
10.234
Parameter Value
1 Origin Bruker BioSpin GmbH
2 Solvent CDCl3
3 Temperature 298.1
4 Number of Scans 1024
5 Spectrometer Frequency 100.61
6 Nucleus 13C
Ac
N
N
Ph
f1 (ppm)

10

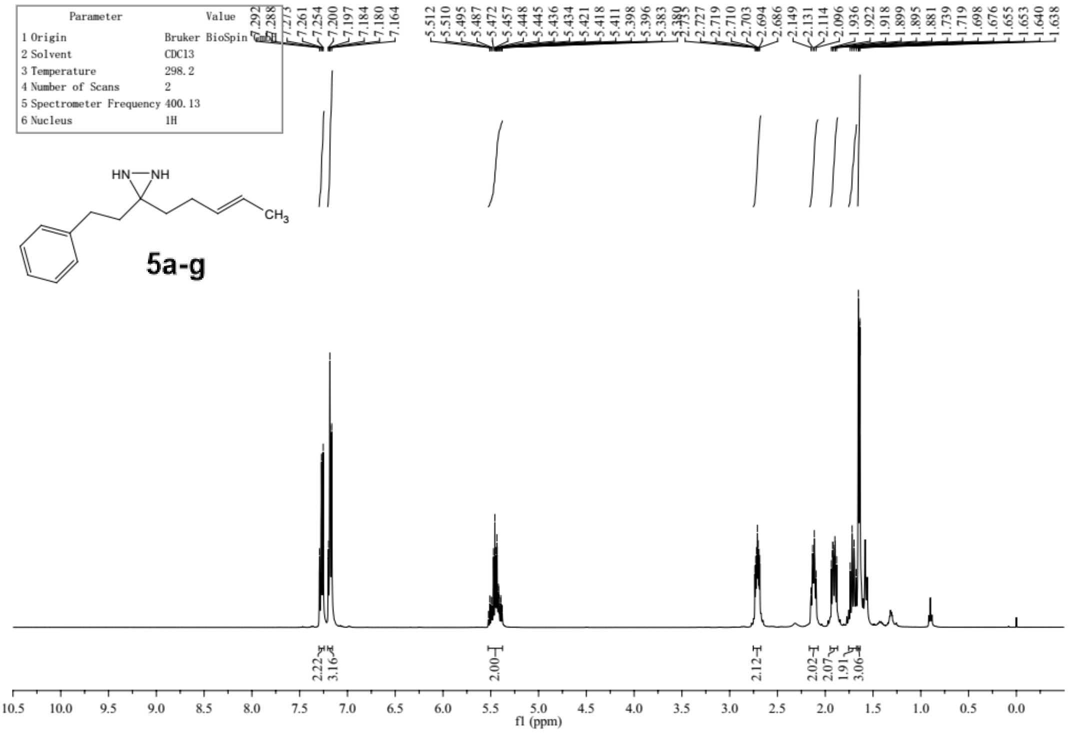
7.292
7.288
7.273
7.261
7.254
7.200
7.197
7.184
7.180
7.164
5.512
5.510
5.495
5.487
5.472
5.457
5.448
5.445
5.436
5.434
5.421
5.418
5.411
5.398
5.396
5.383
2.727
2.719
2.710
2.703
2.694
2.686
2.149
2.131
2.114
2.096
1.936
1.922
1.918
1.899
1.895
1.881
1.739
1.719
1.698
1.676
1.653
1.640
1.638
Parameter Value
1 Origin Bruker BioSpin GmbH
2 Solvent CDCl3
3 Temperature 298.2
4 Number of Scans 2
5 Spectrometer Frequency 400.13
6 Nucleus 1H
HN
NH
CH3
2.22
3.16
2.00
2.12
2.02
2.07
1.91
3.06
f1 (ppm)

5a-g

Parameter	Value
1 Origin	Bruker BioSpin GmbH
2 Solvent	CDCl3
3 Temperature	297.2
4 Number of Scans	80
5 Spectrometer Frequency	100.61
6 Nucleus	13C

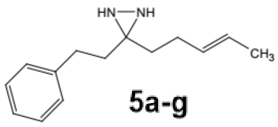

5a-g

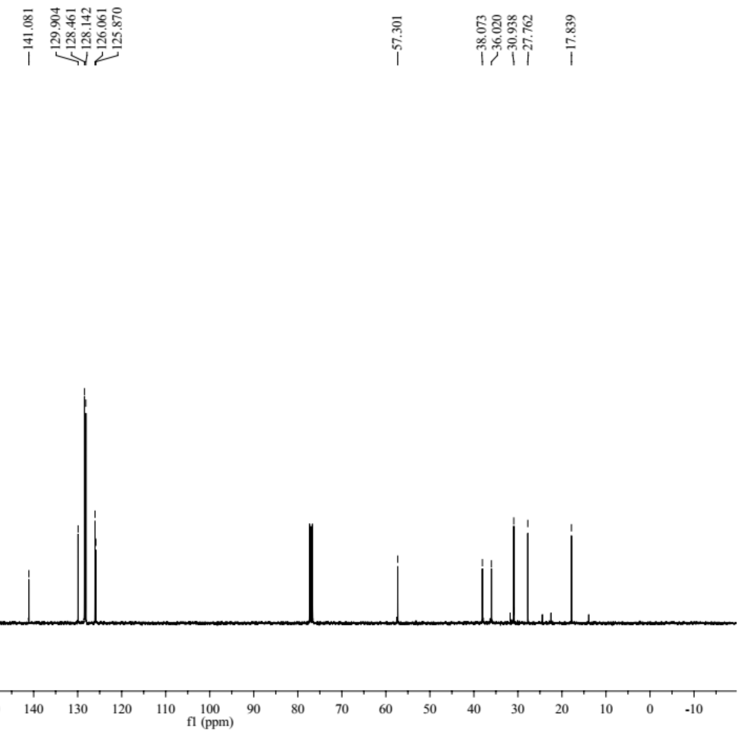

Supplement: SC-015-D3SC04886A-s001 [file SC-015-D3SC04886A-s001.pdf]
